# Risk factors and prognostic implications of diagnosis of cancer within 30 days after an emergency hospital admission (emergency presentation): an International Cancer Benchmarking Partnership (ICBP) population-based study

**DOI:** 10.1016/S1470-2045(22)00127-9

**Published:** 2022-05

**Authors:** Sean McPhail, Ruth Swann, Shane A Johnson, Matthew E Barclay, Hazem Abd Elkader, Riaz Alvi, Andriana Barisic, Oliver Bucher, Gavin R C Clark, Nicola Creighton, Bolette Danckert, Cheryl A Denny, David W Donnelly, Jeff J Dowden, Norah Finn, Colin R Fox, Sharon Fung, Anna T Gavin, Elba Gomez Navas, Steven Habbous, Jihee Han, Dyfed W Huws, Christopher G C A Jackson, Henry Jensen, Bethany Kaposhi, S Eshwar Kumar, Alana L Little, Shuang Lu, Carol A McClure, Bjørn Møller, Grace Musto, Yngvar Nilssen, Nathalie Saint-Jacques, Sabuj Sarker, Luc te Marvelde, Rebecca S Thomas, Robert J S Thomas, Catherine S Thomson, Ryan R Woods, Bin Zhang, Georgios Lyratzopoulos, ICBP Module 9 Emergency Presentations Working Group, Brooke Filsinger, Katharina Forster, Leon May, David S Morrison, A. Ffion Thomas, Janet L Warlow, Hui You

**Affiliations:** a National Disease Registration Service, NHS Digital, Leeds, UK; b Cancer Research UK, London, UK; c Epidemiology of Cancer Healthcare and Outcomes, Department of Behavioural Science and Health, Institute of Epidemiology and Health Care (IEHC), University College London, London, UK; d Cancer Society of New Zealand, Wellington, New Zealand; e Department of Epidemiology and Performance Measurement, Saskatchewan Cancer Agency, Saskatoon, SK, Canada; f Ontario Health (Cancer Care Ontario), Toronto, ON, Canada; g Department of Epidemiology and Cancer Registry, CancerCare Manitoba, Winnipeg, MB, Canada; h Public Health Scotland, Edinburgh, UK; i Cancer Institute NSW, St Leonards, NSW, Australia; j Danish Cancer Society Research Center, Danish Cancer Society, Copenhagen, Denmark; k Northern Ireland Cancer Registry, Queen's University Belfast, Belfast, UK; l Provincial Cancer Care Program, Eastern Health, St John's, NL, Canada; m Victorian Cancer Registry, Cancer Council Victoria, Melbourne, VIC, Australia; n Cancer Support, Treatment and Research, Department of Health, Melbourne, VIC, Australia; o Canadian Partnership against Cancer, Toronto, ON, Canada; p Welsh Cancer Intelligence and Surveillance Unit, Public Health Data, Knowledge and Research Directorate, Public Health Wales, Cardiff, UK; q Population Data Science, Swansea University Medical School, Swansea, UK; r Department of Medicine, Otago Medical School, University of Otago, Dunedin, New Zealand; s Research Unit for General Practice, Aarhus University, Aarhus, Denmark; t Surveillance and Reporting, Advanced Analytics, Cancer Care Alberta, Alberta Health Services, Edmonton; u Calgary, AB, Canada; v New Brunswick Cancer Network, Department of Health, New Brunswick, Fredericton, NB, Canada; w Prince Edward Island Cancer Registry, Queen Elizabeth Hospital, Charlottetown, PE, Canada; x Cancer Registry of Norway, Oslo, Norway; y Nova Scotia Health Cancer Care Program, Halifax, NS, Canada; z Department of the Dean, Medicine Dentistry and Health Sciences, University of Melbourne, Parkville, VIC, Australia; aa Cancer Control Research, BC Cancer, Vancouver, BC, Canada; ab Health Analytics, Department of Health, Fredericton, NB, Canada

## Abstract

**Background:**

Greater understanding of international cancer survival differences is needed. We aimed to identify predictors and consequences of cancer diagnosis through emergency presentation in different international jurisdictions in six high-income countries.

**Methods:**

Using a federated analysis model, in this cross-sectional population-based study, we analysed cancer registration and linked hospital admissions data from 14 jurisdictions in six countries (Australia, Canada, Denmark, New Zealand, Norway, and the UK), including patients with primary diagnosis of invasive oesophageal, stomach, colon, rectal, liver, pancreatic, lung, or ovarian cancer during study periods from Jan 1, 2012, to Dec 31, 2017. Data were collected on cancer site, age group, sex, year of diagnosis, and stage at diagnosis. Emergency presentation was defined as diagnosis of cancer within 30 days after an emergency hospital admission. Using logistic regression, we examined variables associated with emergency presentation and associations between emergency presentation and short-term mortality. We meta-analysed estimates across jurisdictions and explored jurisdiction-level associations between cancer survival and the percentage of patients diagnosed as emergencies.

**Findings:**

In 857 068 patients across 14 jurisdictions, considering all of the eight cancer sites together, the percentage of diagnoses through emergency presentation ranged from 24·0% (9165 of 38 212 patients) to 42·5% (12 238 of 28 794 patients). There was consistently large variation in the percentage of emergency presentations by cancer site across jurisdictions. Pancreatic cancer diagnoses had the highest percentage of emergency presentations on average overall (46·1% [30 972 of 67 173 patients]), with the jurisdictional range being 34·1% (1083 of 3172 patients) to 60·4% (1317 of 2182 patients). Rectal cancer had the lowest percentage of emergency presentations on average overall (12·1% [10 051 of 83 325 patients]), with a jurisdictional range of 9·1% (403 of 4438 patients) to 19·8% (643 of 3247 patients). Across the jurisdictions, older age (ie, 75–84 years and 85 years or older, compared with younger patients) and advanced stage at diagnosis compared with non-advanced stage were consistently associated with increased emergency presentation risk, with the percentage of emergency presentations being highest in the oldest age group (85 years or older) for 110 (98%) of 112 jurisdiction-cancer site strata, and in the most advanced (distant spread) stage category for 98 (97%) of 101 jurisdiction-cancer site strata with available information. Across the jurisdictions, and despite heterogeneity in association size (*I*^2^=93%), emergency presenters consistently had substantially greater risk of 12-month mortality than non-emergency presenters (odds ratio >1·9 for 112 [100%] of 112 jurisdiction-cancer site strata, with the minimum lower bound of the related 95% CIs being 1·26). There were negative associations between jurisdiction-level percentage of emergency presentations and jurisdiction-level 1-year survival for colon, stomach, lung, liver, pancreatic, and ovarian cancer, with a 10% increase in percentage of emergency presentations in a jurisdiction being associated with a decrease in 1-year net survival of between 2·5% (95% CI 0·28–4·7) and 7·0% (1·2–13·0).

**Interpretation:**

Internationally, notable proportions of patients with cancer are diagnosed through emergency presentation. Specific types of cancer, older age, and advanced stage at diagnosis are consistently associated with an increased risk of emergency presentation, which strongly predicts worse prognosis and probably contributes to international differences in cancer survival. Monitoring emergency presentations, and identifying and acting on contributing behavioural and health-care factors, is a global priority for cancer control.

**Funding:**

Canadian Partnership Against Cancer; Cancer Council Victoria; Cancer Institute New South Wales; Cancer Research UK; Danish Cancer Society; National Cancer Registry Ireland; The Cancer Society of New Zealand; National Health Service England; Norwegian Cancer Society; Public Health Agency Northern Ireland, on behalf of the Northern Ireland Cancer Registry; the Scottish Government; Western Australia Department of Health; and Wales Cancer Network.

## Introduction

International differences in cancer survival have been extensively documented although the underlying causes remain unclear and might include differences in diagnostic or treatment pathways.^1^, ^2^, ^3^ Evidence from several studies carried out in single countries indicates that some patients with cancer are diagnosed following an emergency presentation (eg, soon after an emergency hospital admission).^4^ This diagnostic route is associated with lower survival and worse patient-reported outcomes than patients with non-emergency diagnoses, even after adjustment for stage at diagnosis.^4^, ^5^ Emergency presentation, therefore, represents a non-stage prognosticator variable.^6^ Consequently, differences in proportions of patients with cancer who are diagnosed following an emergency presentation might partially explain survival differences between countries or jurisdictions (provinces, devolved administrations, or states) within countries. A crucial first step in examining this hypothesis is to measure emergency presentations in different jurisdictions worldwide.

Diagnosis of cancer as an emergency is a complex phenomenon with contributions from tumour, patient, and health-care system factors, often in combination.^4^ In some patients, emergency presentation results from rapidly advancing disease (at times with few or no prodromal symptoms) presenting with complications such as haemorrhage or gastrointestinal obstruction and requiring emergency hospital care; in such circumstances, emergency presentation might be deemed unpreventable, and indeed represents optimal care.^4^ However, in other patients, emergency presentation might reflect disease progression due to delayed help-seeking or prolonged diagnostic intervals after presentation; therefore, at least some emergency presentations might be preventable through public health or health-care system interventions (appendix p 3).^4^, ^7^ Further, for colorectal and lung cancer, increased participation in screening programmes can lead to a reduction in emergency presentations.^8^, ^9^, ^10^

**Research in context**

**Evidence before this study**

We searched PubMed without language or year of publication restrictions on Dec 3, 2021, for population-based studies examining the frequency, predictors, and consequences of diagnosis of cancer as an emergency presentation, and associations between jurisdiction-level percentages of emergency presentation and cancer survival, using the terms “emergency presentation”, “emergency diagnosis”, “emergency admission”, and “cancer”. Population-based evidence on the frequency, predictors, and consequences of emergency presentations relates to single-country studies with low risk of bias, mainly including patients with colorectal or lung cancer. Single-country evidence suggests that the risk of emergency presentation varies by cancer site, age, and stage at diagnosis, and that emergency presentation is associated with worse survival. We identified no studies examining the consistency of predictors and consequences of emergency presentation between different country populations, and no studies examining associations between the proportion of patients with cancer diagnosed as emergencies and cancer survival in different jurisdictional populations.

**Added value of this study**

To our knowledge, this is the first time that predictors and consequences of diagnosis of cancer as an emergency presentation have been examined in an international context, and that the probable contribution of emergency presentations in international differences in cancer survival has been assessed. As this diagnostic route has consistent predictors and is uniformly (across jurisdictions and the eight cancer sites studied) associated with worse survival, variation in the proportion of emergency presentations is a possible contributor to international variations in cancer survival. Defining emergency presentation through linked cancer registration and administrative hospital admissions data is feasible in different health systems. The findings support incorporating measures of emergency presentation both in international studies comparing cancer survival between jurisdictions, and population-based surveillance to support cancer control efforts within jurisdictions.

**Implications of all the available evidence**

Diagnosis of cancer as an emergency presentation is common and, given its adverse prognostic consequences, it represents a target for cancer control efforts. Future work should examine the tumour, patient, and health-care factors underlying emergency presentation in different countries and health systems, and potential mitigating strategies. These approaches might include interventions to increase participation in population-based screening (eg, for colorectal cancer); public health awareness campaigns to support prompt help-seeking for possible cancer (particularly alarm) symptoms; and health system interventions to increase the diagnostic service capacity to expedite the diagnosis of cancer (such as guidelines for fast-track referral of patients presenting with possible cancer symptoms in primary care, implementation of related care pathways in secondary care, and diagnostic care pathways and services for patients with non-specific symptoms). Incorporating measures of emergency presentation in both international comparative survival studies and in routine cancer surveillance is warranted to support efforts to improve cancer outcomes.

In England, substantial reductions in emergency presentations have been observed between 2006 and 2015.^11^ The size and speed of this decline exceeds what would have been expected by changes in incident cancer site case mix, and is unlikely to reflect changes in tumour biology.^11^ The decline has, therefore, been attributed both to increased public awareness of possible cancer symptoms and health system redesign enabling prompt (2-week wait) specialist referrals of patients with suspected cancer.^12^ Measures of emergency presentations have been introduced in cancer surveillance in England and examined in populations of patients with cancer in Ireland and Denmark.^13^, ^14^, ^15^

We aimed to identify the predictors and consequences of cancer diagnosis through an emergency presentation, and to examine the usefulness and consistency of this metric and its related operational definitions across different countries and data systems. Additionally, we aimed to explore jurisdiction-level associations between emergency presentations and cancer survival.

The study forms part of the International Cancer Benchmarking Partnership (ICBP), a global collaboration of clinicians, policy makers, researchers, and data experts in 21 jurisdictions in seven countries, seeking to explain cancer survival differences between high-income countries with comprehensive cancer registries, similar health system expenditure, and universal health care, to help improve cancer care and outcomes.^1^, ^16^, ^17^, ^18^

## Methods

### Study design and data collection

In this cross-sectional population-based study, we examined cancer registration data linked to hospital admissions data from 14 jurisdictions in six countries: Denmark, Norway, UK (England; Northern Ireland; Scotland; and Wales), Canada (Alberta; Atlantic Canada [comprising Newfoundland and Labrador, New Brunswick, Nova Scotia, and Prince Edward Island, considered jointly]; British Columbia; Ontario; and Saskatchewan-Manitoba [comprising Saskatchewan and Manitoba considered jointly], Australia (New South Wales and Victoria), and New Zealand. Data related to patients aged 15–99 years with a new primary diagnosis of eight cancers—oesophageal (International Classification of Diseases tenth revision [ICD-10] diagnosis code C15), stomach (ICD-10 C16), colon (ICD-10 C18–19), rectal (ICD-10 C20), liver (ICD-10 C22), pancreatic (ICD-10 C25), lung (ICD-10 C34), and ovarian (including cancers of the peritoneum and fallopian tube; ICD-10 C48·1–2, C56, and C57·0)—were extracted. Only invasive tumours (behaviour code 3) were included, and borderline ovarian cancers (third revision of ICD oncology codes 8442, 8451, 8462, 8472, and 8473) were excluded. These inclusion criteria were by-design consistent with those used in a population-based study of cancer survival, covering the jurisdictions included in the present study.^1^ Patients were diagnosed between Jan 1, 2012, and Dec 31, 2017, with small study period differences between jurisdictions due to the variable availability of linked data. Information was available on cancer site, age group, sex, year of diagnosis, and stage at diagnosis. Availability of data on stage at diagnosis reflects the standard collection scope of participating jurisdictional cancer registries during the study years.

Based on linked cancer registry and hospital inpatient admissions data (appendix p 2), emergency presentation was defined as diagnosis of cancer within 30 days after an emergency hospital admission. This definition requires an emergency hospital admission to have occurred, not simply an emergency department attendance. This broad definition of emergency presentation, as described, was used in Norway, Scotland, Wales, the Canadian jurisdictions, and New Zealand. In Denmark, England, Northern Ireland, New South Wales, and Victoria, a narrow version of this definition was used, in which it was additionally required that an emergency hospital admission (in the 30 days before a cancer diagnosis) occurred without an intervening elective hospital admission (appendix pp 4–5). Both definitions are contextual rather than clinical, making no assumptions about the clinical state of the emergency admitted patients, nor the coded reason (diagnosis) relating to the emergency hospital admission episode.^4^ A 30-day cutoff was chosen as a reasonable period to enable histologically verified diagnosis, and because 30-day cutoffs are established in other health-care measures (eg, 30-day postoperative mortality or 30-day hospital readmission). Further, alternative cutoff values at 60 and 90 days indicated only a small incremental yield (appendix p 6). The date of cancer diagnosis was defined as per standard procedures in the participating cancer registries.^1^ Data used in the study were collected under jurisdiction-specific regulations enabling cancer registration and the collection of administrative data on hospital admissions. No participant consent was applicable. No identifiable data were shared for this project. In England, Northern Ireland, Scotland, Wales, Alberta, Victoria, and New Zealand, no additional ethics approvals were necessary given the nature of the study and its alignment with the routine function of cancer registries. In Denmark, studies not involving medical interventions do not require ethics approval. In Ontario, Research Ethics Board approval was obtained by the University of Toronto Research Ethics Board. In Norway, the Norwegian Regional Ethics Committee concluded that no approval was needed for this study (reference 2017/428REK sør-øst A), thus giving the authors exemption from the statutory duty of confidentiality; approval for handling indirect identifiable data was obtained from the Data Protection Officer (reference 2017/6597). In New South Wales, approval for the data used in study was granted by the New South Wales Population and Health Services Research Ethics Committee (HREC/15/CIPHS/15) and the Australian Institute of Health and Welfare Ethics Committee (EO2016/1/224). For Atlantic Canada jurisdictions, British Columbia, and Saskatchewan-Manitoba, data were accessed through the Statistics Canada Research Data Centres, in which confidentiality is protected through stringent policies and procedures enabling public health research.

### Quality control

The overall production and quality control process of data collection involved several steps. First, the availability of high-quality cancer registry and hospital admission data sources for each jurisdiction was explored through meetings and subsequent correspondence between each jurisdiction and SMcP and GL (based in England). This step included collection of information on data availability by type and period on a template spreadsheet. Second, the properties of jurisdiction-specific analysis-ready datasets were specified and communicated back to jurisdictions for feedback. Any queries received helped to clarify operational definitions of different variables and study periods of available data. This step included initial checks on the structure of the available data and key variables and their distribution. Third, data quality analysis code in *R* Markdown (*R* version 4.0.2 and *R*Studio version 1.1.453) was developed in the England data and shared with co-authors in other jurisdictional teams, who ran it against their prespecified datasets and returned aggregate findings for checks. This process encompassed checks of: counts and proportions by variable of interest (age group [15–64 years, 65–74 years, 75–84 years, or ≥85 years]), sex (male or female), disease stage at diagnosis (using either a 3-level or 4-level classification as applicable to each jurisdictional cancer registry), year of diagnosis (2012–17, as applicable), and cancer type; counts of diagnoses and mortality over time; proportion of diagnoses associated with an emergency hospital admission; and, if available, summary statistics of the interval between diagnosis and treatment events (not reported in this manuscript). This process has helped to identify and correct occasional coding errors and misspecifications in jurisdictional datasets. This step was removed in some jurisdictions that joined after analysis scripts had already been quality assured. Fourth, once jurisdictional analyses were run, they were quality-assured locally as per standard procedures used by cancer registries or analysis teams. Anonymous jurisdiction-specific data outputs (aggregated at the level needed for tabulation or figure plotting) were shared with the central team and inspected for potential inconsistencies or gaps, with any resulting queries fed back and addressed, as applicable, by and with the jurisdictional teams. Data were checked further for internal consistency during collation, tabulation, and meta-analysis; any queries raised were discussed with jurisdictional teams and resolved.

The final datasets were received on the following dates: Aug 28, 2020 (Denmark); Sept 15, 2020 (Norway); May 10, 2021 (England); Dec 3, 2020 (Scotland); Sept 28, 2020 (Wales); Oct 19, 2020 (Alberta, Atlantic Canada, British Columbia, Ontario, and Saskatchewan-Manitoba); March 19, 2021 (New South Wales); June 17, 2020 (Victoria); and July 22, 2020 (New Zealand).

### Statistical analysis

A federated or distributed analysis model was used, whereby standardised patient-level datasets were first prespecified, and then created and quality-assured in participating analytical hubs in each jurisdiction. Data were subsequently analysed per-protocol within each participating hub, using centrally developed code scripted in *R* Markdown. Aggregate, group-level, data suitable for publication were subsequently shared with the central team, without any transfer of patient-level data. We examined the frequency of emergency presentation by jurisdiction and variable patient group, and summarised concordance of cancer site ordering using pairwise Pearson correlations (England, the jurisdictions with the largest sample, vs each other jurisdiction).

To account for potential confounding between exposure variables, in each jurisdiction-specific dataset, multivariable logistic regression was used to estimate adjusted odds ratios (ORs) for emergency presentation. To support interpretation, two incremental models were used. The first model included cancer site, age group, sex, and year of diagnosis, and the second additionally included stage at diagnosis. To examine consistency in adjusted associations between patient-level variables and emergency presentation, and to obtain pooled (cross-jurisdictional) estimates, we used random effect meta-analysis, additionally including definition type (broad or narrow) as an effect modifier. The *I*^2^ statistic, representing the proportion of total variation between jurisdictions not explained by sample variation, was used to measure statistical heterogeneity. Meta-analyses used estimates from the first model (ie, without adjustment for disease stage at diagnosis) for each of the 14 jurisdictions.

We used logistic regression to examine crude and adjusted associations between emergency presentation and short-term (all-cause, observed) mortality at 1, 3, and 12 months from diagnosis in each jurisdictional dataset. Analysing all-cause mortality obviates concerns about inaccuracies in ascertainment of causes of death. Mortality relates to ascertained death, with censored patients with follow-up of less than 12 months assumed to have survived for at least 12 months. This assumption was deemed appropriate, since loss to follow-up in participating jurisdictional cancer registries is very low (0·0–0·2% at 5 years).^2^ For example, in the England subcohort, the percentage of patients lost to follow-up before 12 months was 0·1%. Given that vital status ascertainment was highly complete, logistic instead of Cox proportional hazards regression was used for simplicity.^19^ To examine consistency in adjusted associations between emergency presentation and mortality, and pool estimates, we used random effect meta-analysis, additionally examining definition type (broad or narrow) as an effect modifier. We pooled estimates adjusted for all variables, including stage at diagnosis, restricting to the 12 jurisdictions with stage data for all eight cancer sites (ie, excluding British Columbia and Victoria). In an additional analysis we pooled estimates adjusted for all variables other than stage at diagnosis (across all 14 jurisdictions).

Lastly, we examined jurisdiction-level (ecological) associations between the percentage of patients diagnosed through an emergency presentation and the corresponding, previously reported, 1-year net survival estimates for 2010–14.^1^, ^18^ For each cancer site, we used a linear regression model treating jurisdiction-level net survival as the outcome and adjusting for jurisdiction-level percentage of emergency presentations and definition type (broad or narrow) used. To maximise statistical power in these analyses, jurisdiction-specific estimates were used for 17 jurisdictions (New Brunswick, Nova Scotia, Prince Edward Island, Manitoba, and Saskatchewan were considered individually, and not as part of a jurisdictional cluster). *R* versions 4.0.2 and 4.1.0 were used to create analysis scripts and for visualisation, with the metafor package (version 3.0-2) used for meta-analysis,^19^ and tidyverse (version 1.3.1), gt (version 0.3.0), gtsummary (version 1.4.2), and labelled (version 2.8.0) packages for data and results preparation (appendix p 7). Jurisdictional aggregate data were collated in Excel. We considered p values less than 0·05 to indicate statistical significance, and calculated 95% CIs around proportions using the Wilson score method. We did not adjust for multiple comparisons.

The reported analysis differed from that originally planned in three ways. First, through initial steps in the quality assurance process it became apparent that information on screening detection status was not uniformly available, therefore screening detection was not included in the analysis code, first shared with jurisdictions in July, 2019. Second, subsequent quality assurance steps showed that two different operational definitions of emergency presentation had been applied, prompting description of differences between broad and narrow definitions (including the percentage of patients identified as emergency presentations under both definitions for England and New South Wales), included in the manuscript in August, 2021. Third, coauthor feedback and discussions on earlier drafts led to the addition of meta-analyses, included in the manuscript in August, 2021. All three changes were approved by SMcP and GL, communicated in writing to all coauthors, and endorsed through feedback.

### Role of the funding source

The funder of the study had no role in the study design, data analysis, data interpretation, or the writing of the report, although they have facilitated resources for data collection and analysis for the project.

## Results

Of 964 619 patients with one of the eight studied cancers, 107 551 (11·1%) met exclusion criteria and 857 068 (88·9%) were included in the analysis (appendix p 8). Across jurisdictions, sample composition was similar by cancer site, age group, and sex (appendix pp 9–11). Among analysed cases, data were complete for all variables except stage at diagnosis (appendix p 12).

Across jurisdictions, considering all of the cancer sites together, the percentage of diagnoses through emergency presentations ranged from 24·0% (9165 of 38 212 patients) to 42·5% (12 238 of 28 794 patients); between jurisdictions, use of a broad definition was generally associated with higher percentages of emergency presentations than use of a narrow definition (table 1). Post-hoc exploratory analyses using data from England and New South Wales suggested that the broad definition gives around 5 absolute percentage points more emergency presentations in these jurisdictions than the narrow definition (appendix pp 4–5). There was consistently large variation in the percentage of emergency presentations by cancer site across jurisdictions. Pancreatic cancer diagnoses had the highest percentage of emergency presentations on average overall (46·1% [30 972 of 67 173 patients]), with the jurisdictional range being 34·1% (1083 of 3172 patients) to 60·4% (1317 of 2182 patients). Rectal cancer had the lowest percentage of emergency presentations on average overall (12·1% [10 051 of 83 325 patients]), with a jurisdictional range of 9·1% (403 of 4438 patients) to 19·8% (643 of 3247 patients; table 1). The ordering of cancer sites in terms of the percentage of emergency presentations was highly consistent across jurisdictions, with pairwise correlations between the percentage in England and each of the other jurisdictions being at minimum 0·88 (appendix p 13).

**Table 1:** Percentage of patients diagnosed through emergency presentation (defined as diagnosis of cancer within 30 days of an emergency hospital admission) by cancer site and jurisdiction

	**All cancer sites**	**Oesophageal cancer**	**Stomach cancer**	**Colon cancer**	**Rectal cancer**	**Liver cancer**	**Pancreatic cancer**	**Lung cancer**	**Ovarian cancer**
Narrow definition*									
Denmark	30·9%	24·3%	32·2%	28·3%	13·5%	43·3%	48·0%	36·4%	20·6%
England	31·3%	19·7%	31·4%	29·3%	10·7%	42·4%	46·9%	34·7%	34·8%
Northern Ireland	27·9%	19·9%	29·6%	23·8%	9·1%	36·9%	42·7%	32·0%	29·6%
New South Wales	30·9%	26·9%	30·5%	27·6%	12·1%	36·8%	45·3%	36·4%	28·4%
Victoria	24·0%	20·0%	23·7%	22·9%	9·1%	31·9%	34·1%	27·8%	20·9%
**Broad definition**†									
Norway	36·5%	32·7%	40·0%	35·8%	15·8%	50·6%	55·4%	39·4%	34·9%
Scotland	38·5%	25·2%	39·1%	35·1%	14·1%	47·5%	59·2%	42·1%	42·8%
Wales	37·4%	25·5%	39·1%	34·2%	13·8%	50·5%	56·7%	41·5%	40·8%
Alberta	30·0%	23·0%	34·6%	28·6%	12·9%	31·7%	40·9%	33·3%	25·7%
Atlantic Canada	26·9%	20·7%	30·4%	28·4%	12·6%	34·3%	38·8%	26·6%	28·1%
British Columbia	30·5%	28·3%	40·3%	27·5%	12·0%	38·1%	41·4%	33·2%	31·7%
Ontario	26·1%	20·3%	26·9%	27·2%	11·0%	28·3%	35·3%	27·5%	23·9%
Saskatchewan-Manitoba	28·3%	18·5%	31·8%	27·7%	13·7%	33·3%	37·3%	30·6%	30·1%
New Zealand	42·5%	36·8%	47·8%	36·6%	19·8%	49·7%	60·4%	51·1%	48·1%

For n/N and 95% CIs of presented estimates, see appendix pp 15–28.

* A narrow operational definition of emergency presentation was used.

† A broad operational definition of emergency presentation was used.

For 110 (98%) of 112 jurisdiction–cancer site strata, the percentage of emergency presentations was greatest in the oldest age group (ie, 85 years or older; appendix pp 14–28). Additionally, for colon and stomach cancer, a J-shaped pattern by age was apparent in most jurisdictions, whereby the youngest patient group (aged 15–64 years) had a higher percentage of emergency presentations than the one immediately older (patients aged 65–74 years). For 98 (97%) of 101 jurisdiction–cancer site strata with available information, the percentage of emergency presentations was greatest among patients with the most advanced stage of cancer at diagnosis (appendix pp 14–28). There was either weak and inconsistent or no evidence for variation in percentages of emergency presentation by diagnosis year during the applicable study periods (2012–17; appendix pp 15–28).

Adjusted ORs for emergency presentation were consistent with the univariable analysis findings, both from models including and models not including adjustment for stage (appendix pp 29–35 for stage-adjustment; appendix pp 36–42 for no-stage-adjustment). This finding indicates little confounding between the examined exposure variables, particularly between cancer site, age group, and stage at diagnosis (the three stronger predictors).

Meta-analyses indicated overall concordance across the jurisdictions in the direction and strength of adjusted associations with emergency presentation, particularly regarding age group and sex. Associations with cancer site (treating colon cancer as the reference group) were generally concordant across the jurisdictions, with occasional variability in direction (appendix pp 36–42). Pooled estimates for associations between exposure variables and emergency presentation risk were similar for jurisdictions using either broad or narrow definitions of emergency presentation (appendix pp 36–42).

In all 112 (100%) jurisdiction–cancer site strata, diagnosis through emergency presentation was strongly associated with a greater risk of 12-month mortality than non-emergency presentation diagnoses. The estimated odds ratio for 12-month mortality was 1·9 or greater for all jurisdiction-cancer site strata, with the minimum lower bound of the related 95% CIs being 1·26. The weakest observed association of emergency presentation with 12-month mortality was for oesophageal cancer in Atlantic Canada (OR 1·92; 95% CI 1·30–2·84; Table 2, Table 3; appendix pp 43–44). Although mortality differences by emergency presentation status were similar across cancer sites on the odds scale, absolute differences were generally greater for rectal cancer than for other cancers and lower for pancreatic cancer than for other cancers (Table 2, Table 3; appendix pp 43–44). Differences in the risk of mortality by emergency presentation status were greater at 1 and 3 months than at 12 months (appendix pp 43–44).

**Table 2:** Observed 12-month mortality by emergency presentation status and cancer site (oesophageal, stomach, colon, and rectal)

	**Oesophageal cancer**				**Stomach cancer**				**Colon cancer**				**Rectal cancer**			
	EP	Non-EP	Abs diff	OR (95% CI)	EP	Non-EP	Abs diff	OR (95% CI)	EP	Non-EP	Abs diff	OR (95% CI)	EP	Non-EP	Abs diff	OR (95% CI)
Denmark*	77%	46%	30%	3·77 (2·97–4·79)	64%	37%	27%	3·01 (2·50–3·62)	35%	12%	23%	4·03 (3·67–4·41)	35%	10%	25%	5·03 (4·24–5·97)
Norway†	74%	43%	31%	3·70 (2·88–4·76)	65%	34%	31%	3·63 (3·05–4·32)	36%	12%	24%	4·16 (3·83–4·52)	36%	9%	27%	5·77 (4·87–6·83)
England*	84%	50%	34%	5·29 (4·90–5·72)	79%	48%	31%	3·96 (3·70–4·24)	52%	20%	32%	4·27 (4·14–4·41)	58%	14%	44%	8·75 (8·13–9·42)
Northern Ireland*	82%	46%	36%	5·39 (3·62–8·01)	84%	48%	35%	5·42 (3·85–7·64)	50%	17%	33%	4·75 (4·05–5·58)	59%	12%	47%	10·57 (7·12–15·69)
Scotland†	82%	51%	31%	4·31 (3·56–5·22)	75%	50%	24%	2·92 (2·43–3·50)	51%	20%	31%	4·13 (3·77–4·53)	49%	13%	36%	6·23 (5·08–7·65)
Wales†	80%	51%	29%	3·90 (3·00–5·05)	79%	51%	28%	3·58 (2·85–4·48)	51%	21%	30%	3·89 (3·47–4·36)	57%	15%	42%	7·46 (5·86–9·48)
Alberta†	75%	51%	24%	2·87 (1·94–4·25)	64%	46%	18%	2·07 (1·59–2·70)	37%	15%	23%	3·47 (3·02–3·99)	40%	11%	30%	5·70 (4·25–7·64)
Atlantic Canada†	67%	51%	16%	1·92 (1·30–2·84)	69%	43%	26%	2·89 (2·21–3·77)	30%	12%	18%	3·05 (2·66–3·49)	52%	12%	40%	7·72 (5·85–10·20)
British Columbia†	77%	51%	26%	3·14 (2·35–4·19)	67%	42%	25%	2·81 (2·28–3·46)	28%	11%	17%	3·30 (2·94–3·72)	40%	11%	29%	5·51 (4·38–6·92)
Ontario†	78%	49%	29%	3·62 (2·95–4·44)	66%	35%	31%	3·57 (3·18–4·01)	33%	11%	22%	3·97 (3·71–4·26)	41%	10%	32%	6·63 (5·66–7·76)
Saskatchewan-Manitoba†	75%	58%	17%	2·14 (1·26–3·65)	64%	42%	22%	2·45 (1·83–3·27)	29%	12%	18%	3·13 (2·66–3·68)	44%	12%	32%	5·79 (4·32–7·76)
New South Wales*	74%	46%	29%	3·43 (2·75–4·28)	63%	32%	32%	3·76 (3·21–4·41)	33%	10%	22%	4·20 (3·85–4·57)	39%	8%	31%	7·32 (6·06–8·83)
Victoria*	76%	43%	32%	4·06 (3·07–5·37)	66%	35%	31%	3·58 (2·96–4·33)	32%	11%	21%	3·74 (3·37–4·16)	36%	8%	28%	6·37 (5·04–8·04)
New Zealand†	78%	51%	27%	3·38 (2·59–4·42)	68%	41%	27%	3·11 (2·52–3·84)	38%	14%	24%	3·86 (3·49–4·27)	35%	11%	25%	4·65 (3·79–5·71)

Numerator and denominator data and 95% CIs for proportions and differences are provided in the appendix (pp 61–69). EP=emergency presentation. Abs diff=absolute difference. OR=odds ratio.

* Narrow operational definition was used.

† Broad operational definition was used.

**Table 3:** Observed 12-month mortality by emergency presentation status and cancer site (liver, pancreatic, lung, and ovarian)

	**Liver cancer**				**Pancreatic cancer**				**Lung cancer**				**Ovarian cancer**			
	EP	Non-EP	Abs diff	OR (95% CI)	EP	Non-EP	Abs diff	OR (95% CI)	EP	Non-EP	Abs diff	OR (95% CI)	EP	Non-EP	Abs diff	OR (95% CI)
Denmark*	81%	48%	33%	4·59 (3·63–5·80)	81%	60%	21%	2·85 (2·44–3·33)	71%	43%	27%	3·15 (2·94–3·38)	47%	15%	32%	5·10 (4·00–6·50)
Norway†	75%	43%	32%	3·98 (3·10–5·10)	84%	57%	27%	4·03 (3·45–4·69)	77%	39%	38%	5·33 (4·95–5·75)	37%	11%	25%	4·56 (3·73–5·58)
England*	84%	52%	32%	4·85 (4·51–5·22)	89%	69%	20%	3·59 (3·37–3·81)	86%	52%	34%	5·70 (5·54–5·87)	54%	19%	35%	4·95 (4·65–5·28)
Northern Ireland*	87%	53%	34%	6·14 (3·91–9·66)	93%	68%	26%	6·69 (4·53–9·87)	90%	56%	34%	7·21 (6·11–8·50)	56%	26%	30%	3·59 (2·62–4·91)
Scotland†	80%	44%	36%	5·20 (4·32–6·26)	83%	69%	14%	2·20 (1·85–2·62)	81%	52%	29%	3·84 (3·59–4·11)	49%	21%	28%	3·58 (2·96–4·33)
Wales†	84%	53%	31%	4·65 (3·53–6·12)	83%	68%	15%	2·33 (1·88–2·88)	83%	53%	30%	4·31 (3·90–4·76)	40%	16%	25%	3·67 (2·89–4·65)
Alberta†	80%	38%	41%	6·32 (4·66–8·59)	85%	64%	21%	3·13 (2·45–4·01)	78%	45%	33%	4·24 (3·80–4·73)	47%	20%	27%	3·50 (2·56–4·80)
Atlantic Canada†	83%	52%	31%	4·57 (3·09–6·74)	88%	72%	15%	2·69 (2·01–3·60)	82%	47%	35%	5·10 (4·55–5·71)	63%	23%	39%	5·48 (3·93–7·63)
British Columbia†	85%	53%	32%	5·18 (4·05–6·61)	86%	68%	18%	2·89 (2·36–3·54)	82%	45%	37%	5·64 (5·15–6·18)	47%	14%	33%	5·52 (4·33–7·05)
Ontario†	72%	38%	34%	4·15 (3·58–4·81)	74%	58%	17%	2·11 (1·88–2·36)	76%	42%	35%	4·50 (4·27–4·75)	48%	15%	33%	5·33 (4·6–6·18)
Saskatchewan-Manitoba†	77%	55%	22%	2·75 (1·86–4·06)	84%	69%	16%	2·45 (1·84–3·26)	82%	46%	36%	5·43 (4·76–6·18)	45%	12%	33%	6·00 (4·12–8·74)
New South Wales*	75%	38%	38%	5·02 (4·27–5·90)	77%	57%	20%	2·58 (2·26–2·94)	74%	41%	32%	3·94 (3·68–4·23)	42%	14%	28%	4·46 (3·61–5·50)
Victoria*	72%	36%	36%	4·51 (3·73–5·46)	76%	54%	22%	2·73 (2·32–3·22)	74%	41%	33%	4·17 (3·80–4·59)	37%	12%	26%	4·43 (3·39–5·79)
New Zealand†	76%	41%	35%	4·52 (3·54–5·76)	83%	69%	14%	2·21 (1·81–2·72)	77%	45%	32%	4·13 (3·76–4·53)	42%	17%	25%	3·52 (2·75–4·50)

Numerator and denominator data and 95% CIs for proportions and differences are provided in the appendix (pp 61–69). EP=emergency presentation. Abs diff=absolute difference. OR=odds ratio.

* Narrow operational definition was used.

† Broad operational definition was used.

**Figure 1:**
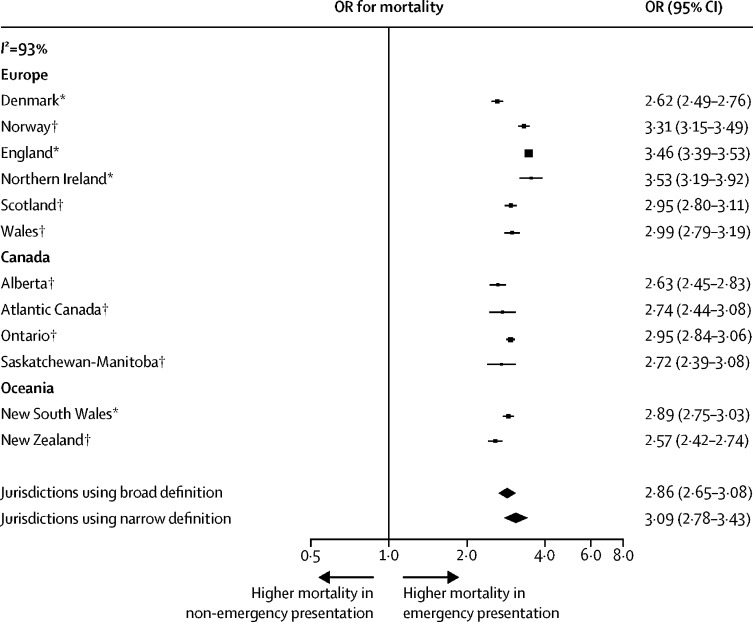
Meta-analysis of jurisdiction-specific associations between emergency presentation and 12-month mortality Emergency presentation is defined as diagnosis of cancer within 30 days after an emergency hospital admission. Results are restricted to the 12 jurisdictions with information on disease stage at diagnosis for all eight cancer sites and have been adjusted for cancer site, age group, sex, and year. OR=odds ratio. *Narrow operational definition was used. †Broad operational definition was used.

After adjustment for variables other than stage at diagnosis, ORs for 12-month mortality for patients diagnosed through emergency presentation compared with those diagnosed as non-emergency presentations (all cancer sites combined) were greater than 3·2 in all 14 jurisdictions, with the minimum lower bound of the 95% CIs being 3·13 (table 4). After additionally adjusting for disease stage at diagnosis for all eight cancer sites studied, the ORs for 12-month mortality were greater than 2·5 in all 12 jurisdictions contributing data on disease stage at diagnosis, with the minimum lower bound of the 95% CIs being 2·42 (figure 1; table 4; appendix pp 45–58).

**Table 4:** Frequency and ORs of 12-month mortality by jurisdiction and emergency presentation status across patients with any of the studied eight cancers

	**Number of patients**	Number of 12-month mortalities*	**Crude OR (95% CI)**†	**Adjusted (stage-unadjusted) OR (95% CI)**†	**Stage-adjusted OR (95% CI)**†
**Denmark**§					
Non-EP	31 804	9013 (28·3%)	(ref)	(ref)	(ref)
EP	14 199	8597 (60·5%)	3·88 (3·72–4·05)	3·28 (3·13–3·43)	2·62 (2·49–2·76)
**Norway**‡					
Non-EP	29 281	7222 (24·7%)	(ref)	(ref)	(ref)
EP	16 866	10 107 (59·9%)	4·57 (4·39–4·76)	4·37 (4·17–4·58)	3·31 (3·15–3·49)
**England**§					
Non-EP	256 260	99 437 (38·8%)	(ref)	(ref)	(ref)
EP	116 934	88 261 (75·5%)	4·85 (4·78–4·93)	4·54 (4·46–4·62)	3·46 (3·39–3·53)
**Northern Ireland**§					
Non-EP	11 179	4328 (38·7%)	(ref)	(ref)	(ref)
EP	4322	3380 (78·2%)	5·68 (5·24–6·17)	5·46 (4·98–5·99)	3·53 (3·19–3·92)
**Scotland**‡					
Non-EP	27 708	10 910 (39·4%)	(ref)	(ref)	(ref)
EP	17 321	12 507 (72·2%)	4·00 (3·84–4·17)	3·60 (3·44–3·77)	2·95 (2·80–3·11)
**Wales**‡					
Non-EP	16 376	6253 (38·2%)	(ref)	(ref)	(ref)
EP	9763	7017 (71·9%)	4·14 (3·92–4·37)	3·79 (3·57–4·03)	2·99 (2·79–3·19)
**Alberta**‡¶					
Non-EP	17 741	5599 (31·6%)	(ref)	(ref)	(ref)
EP	7441	4839 (65·0%)	4·03 (3·81–4·27)	3·86 (3·62–4·12)	2·63 (2·45–2·83)
**Atlantic Canada**‡¶					
Non-EP	8065	2745 (34·0%)	(ref)	(ref)	(ref)
EP	2905	1855 (63·9%)	3·42 (3·13–3·74)	3·79 (3·42–4·21)	2·74 (2·44–3·08)
**British Columbia**‡¶‖					
Non-EP	11 295	3555 (31·5%)	(ref)	(ref)	(ref)
EP	5195	3320 (63·9%)	3·84 (3·59–4·12)	4·01 (3·70–4·36)	3·11 (2·85–3·39)
**Ontario**‡					
Non-EP	68 316	20 197 (29·6%)	(ref)	(ref)	(ref)
EP	24 131	14 623 (60·6%)	3·66 (3·55–3·78)	3·87 (3·74–4·00)	2·95 (2·84–3·06)
**Saskatchewan-Manitoba**‡¶					
Non-EP	5985	1955 (32·7%)	(ref)	(ref)	(ref)
EP	2400	1520 (63·3%)	3·57 (3·24–3·95)	3·77 (3·36–4·24)	2·72 (2·39–3·09)
**New South Wales**§					
Non-EP	36 753	9663 (26·3%)	(ref)	(ref)	(ref)
EP	16 445	9836 (59·8%)	4·17 (4·01–4·34)	3·74 (3·58–3·91)	2·89 (2·75–3·03)
**Victoria**§‖					
Non-EP	29 047	7557 (26·0%)	(ref)	(ref)	(ref)
EP	9165	5328 (58·1%)	3·95 (3·76–4·15)	3·70 (3·50–3·92)	3·33 (3·15–3·53)
**New Zealand**‡					
Non-EP	16 556	4653 (28·1%)	(ref)	(ref)	(ref)
EP	12 238	7572 (61·9%)	4·15 (3·95–4·36)	3·73 (3·53–3·95)	2·57 (2·42–2·74)

Adjusted ORs for emergency presentation estimated after adjustment for other variables other than stage (ie, including cancer site, age group, and sex; but without adjustment for stage), and then including all variables and including stage at diagnosis (stage adjusted; appendix pp 45–58). EP=emergency presentation. OR=Odds ratio. ref=reference group.

* For 95% CIs for mortality proportions see appendix pp 45–58.

† p values for all OR values shown are p<0·0001.

‡ Broad operational definition was used.

§ Narrow operational definition was used.

¶ Data for these jurisdictions relate to cancer cases diagnosed in 2012 and 2013.

‖ Victoria and British Columbia did not contribute stage data across all cancer sites; see appendix (p 12) for missing stage data by jurisdiction.

Meta-analysis supported the finding that emergency presentation was strongly associated with increased 12-month mortality across all jurisdictions, with little evidence of difference between those using broad (OR 2·86 [95% CI 2·65–3·08]) and narrow (OR 3·09 [2·78–3·43]) definitions; figure 1). Although associations were qualitatively concordant, their size differed by jurisdiction (*I*^2^=93%). Similar findings were observed in a meta-analysis without adjustment for disease stage at diagnosis (appendix p 59).

**Figure 2:**
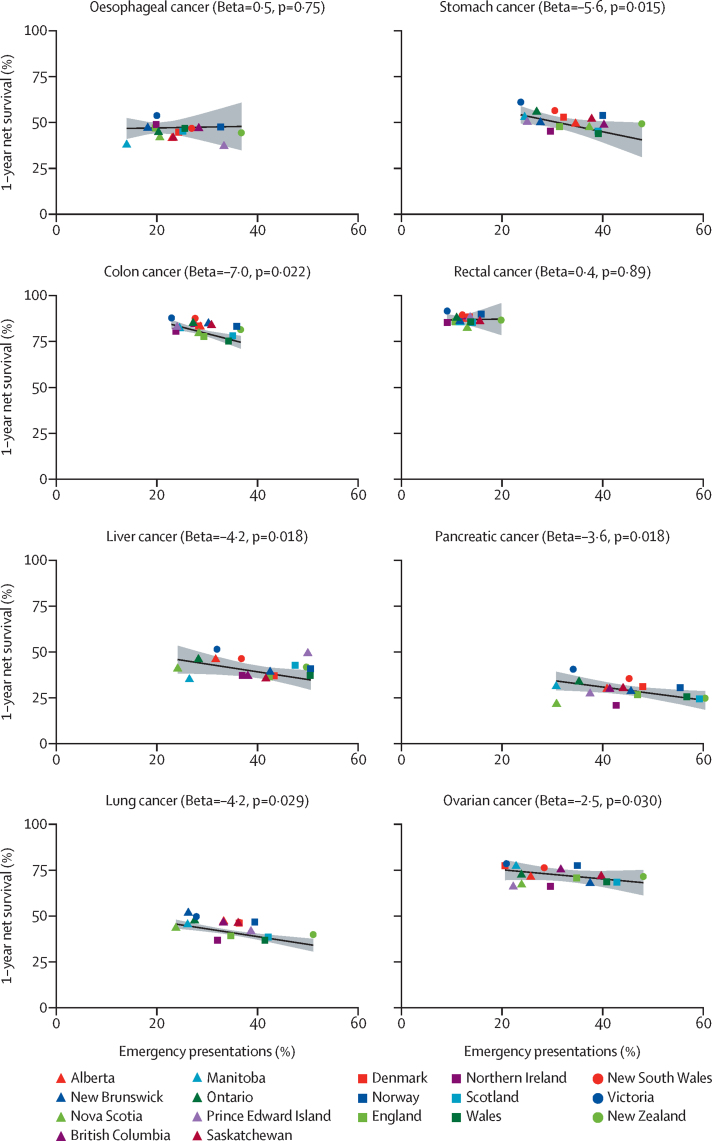
Ecological associations between jurisdiction-level percentage of emergency presentation and 1-year net survival, by cancer site, adjusted for operational definition used (narrow or broad) Emergency presentation is defined as diagnosis of cancer within 30 days after an emergency hospital admission. The grey surface surrounding the central estimates illustrates the 95% CIs. The lines are positioned for jurisdictions using the narrow definition. To maximise statistical power, jurisdiction-specific estimates were used in these models for New Brunswick, Nova Scotia, and Prince Edward Island (part of Atlantic Canada in other analyses), and Manitoba and Saskatchewan (considered jointly in other analyses), therefore including estimates for 17 jurisdictions (appendix p 60).

There was evidence for inverse associations between jurisdiction-level percentage of emergency presentations and corresponding 1-year net survival for stomach, colon, liver, pancreatic, lung, and ovarian cancers, with net survival decreasing by between 2·5% (95% CI 0·28–4·7; ovarian cancer) and 7·0% (1·2–13·0; colon cancer) for a 10 percentage point increase in emergency presentation (p value range: 0·015–0·030; table 5, figure 2). There was no evidence for jurisdiction-level associations between net survival and emergency presentation for oesophageal cancer (p=0·75) or rectal cancer (p=0·89; table 5, figure 2).

**Table 5:** Associations between jurisdiction-level percentage of emergency presentations and corresponding 1-year net survival

	**Oesophageal cancer**		**Stomach cancer**		**Colon cancer**		**Rectal cancer**		**Liver cancer**		**Pancreatic cancer**		**Lung cancer**		**Ovarian cancer**	
	Beta (95% CI)	p	Beta (95% CI)	p	Beta (95% CI)	p	Beta (95% CI)	p	Beta (95% CI)	p	Beta (95% CI)	p	Beta (95% CI)	p	Beta (95% CI)	p
10% increase in emergency presentations	0·5% (−2·7 to 3·6)	0·75	−5·6% (−9·9 to −1·3)	0·015	−7·0% (−13·0 to −1·2)	0·022	0·4% (−5·2 to 5·9)	0·89	−4·2% (−7·6 to −0·85)	0·018	−3·6% (−6·4 to −0·71)	0·018	−4·2% (−7·9 to −0·50)	0·029	−2·5% (−4·7 to −0·28)	0·030
*R*^2^	0·18	..	0·36	..	0·36	..	0·02	..	0·41	..	0·34	..	0·39	..	0·29	..

Beta values denote the percentage change in jurisdiction-level 1-year net survival associated with a 10% increase in jurisdiction-level percentage of emergency presentations. To maximise statistical power, jurisdiction-specific estimates were used in these models for New Brunswick, Nova Scotia, Prince Edward Island (part of Atlantic Canada in other analyses), and Manitoba and Saskatchewan (considered jointly in other analyses), therefore including estimates for 17 jurisdictions.

## Discussion

Emergency presentation, defined as diagnosis of cancer within 30 days of an emergency hospital admission, affected large proportions of patients with cancer across the studied jurisdictions. Specific cancer sites, older patients (ie, patients in the 75–84 years and ≥85 years), and patients diagnosed at an advanced stage are over-represented in this diagnostic route, which is strongly associated with higher mortality than non-emergency presentation diagnosis. Ecological analyses support the hypothesis that variation in proportions of emergency presentations between jurisdictions contributes to international differences in cancer survival.

The findings substantially amplify previous, single-country, evidence on the predictors and outcomes of emergency presentation.^4^, ^20^, ^21^ The observed large variation in risk of emergency presentation by cancer site might reflect the so-called symptom signature and diagnostic difficulty of the studied cancers (ie, the proportion of patients presenting with symptoms of relatively high specificity for cancer).^22^ Pancreatic cancer, which had the highest percentage of emergency presentations consistently across jurisdictions, often presents with symptoms of low predictive value for cancer, such as abdominal or back pain.^22^ Similarly, many patients with lung, colon, and ovarian cancer present with non-specific symptoms, for example, cough, abdominal pain, or bloating, and these cancers are associated with high proportions of multiple prediagnostic primary care consultations.^23^ By contrast, oesophageal and rectal cancer, which had lower proportions of emergency presentations consistently across jurisdictions, typically have clearer symptom signatures, including cancer alarm symptoms such as dysphagia and rectal bleeding.^22^ Including additional cancer sites in future international studies could help to examine this hypothesis further. Similar to most other studies in this field, we could not examine health-care use before the relevant emergency hospital admission.^4^ Other research indicates three major subtypes of emergency presenters: some have no history of primary care consultations, some have sought help and were being investigated electively when the emergency presentation occurred, and others have sought help (at times repeatedly) but no investigations were instigated (appendix p 3).^24^, ^25^ Future international studies should address this gap by incorporating links to primary care electronic health records.

Previous population-based evidence relates to single-country studies, mainly examining emergency presentations for specific cancer sites (most often colorectal or lung cancer). To our knowledge, this is the first time that predictors and consequences of emergency presentation have been studied in multiple jurisdictions in patients with eight cancer sites using linked population-based cancer registry and hospital admissions data. Beyond the role of differences in definition type used, differences between jurisdictions in the percentage of emergency presenters could reflect artefactual differences in hospital admission data sources, or differences in emergency care organisation or clinical severity thresholds, prompting emergency hospital admission in different health systems (panel). Although some emergency department attendances could reflect low urgency care, emergency hospital admissions (as used in our definition of emergency presentation) require a medical decision to admit the patient, typically on grounds of clinical urgency, minimising concerns about variability introduced by different patterns of emergency department attendance. Further, our measure of emergency presentation had consistent predictors and associations with mortality across jurisdictions, indicating a highly similar underlying construct. We nonetheless advise against formal ranking of jurisdictions according to their emergency presentation percentage, particularly when different definition types are used.

Panel **Factors possibly contributing to differences in the percentage of patients diagnosed through emergency presentation and in the strength of its association with mortality risk between jurisdictions**

**Factors possibly contributing to differences in the percentage of patients diagnosed through emergency presentation between jurisdictions**

• Actual differences in:
  • Public perceptions and understanding of possible cancer symptoms
  • Presence or absence and content of clinical guidelines for patients with new-onset symptoms that might be due to cancer
  • Health service organisation (eg, poor access to primary care or specialist referrals or investigations that can lead to an increased risk of emergency presentation)
  • Existence and uptake of population-based cancer screening programmes (particularly for colorectal cancer)
• Artefactual differences due to:
  • Definition type used (broad or narrow)
  • Unmeasured differences in emergency care organisation and in the administrative data sources and operational definitions used (eg, how emergency hospital admissions are semantically defined)
  • Unmeasured confounding (eg, by socioeconomic status, comorbidity, or rurality)

**Factors possibly contributing to differences in strength of associations between emergency presentation and mortality risk between jurisdictions**

• Both actual and artefactual differences in the percentage of patients diagnosed through emergency presentation (in the earlier part of this panel) affecting the clinical severity case mix of emergency presenters, and therefore their prognosis, resulting in between-jurisdiction differences in the observed risk of mortality associated with emergency presentation
• Actual differences in the quality of clinical management of emergency presenters (eg, regarding the provision of emergency surgery when required)

Consistent with previous studies, our definition is agnostic to the actual clinical circumstances of presenting patients and to the coded reasons (diagnoses) assigned to the emergency hospital admissions; this approach is particularly suitable for population-based studies because it is robust to inaccuracies in coded diagnoses in hospital administration data.^4^ Enriching operational definitions of emergency presentation by clinical information captured in routine data sources (eg, emergency laparotomy contributing to defining emergency presentations in colorectal cancer) can be useful, but would require a large range of clinical scenarios specific to each cancer site to be appropriately defined in administrative data sources in different jurisdictions—a non-trivial task. Additionally, patients presenting as genuine emergencies but managed non-surgically (palliatively) because of poor operative risk will not be captured by emergency presentation definitions relying on surgical management. Therefore, some patients identified as emergency presenters in our study would have had no critical symptomatology, or their emergency presentations might have been triggered by conditions other than their underlying cancer. Consequently, if it were possible to accurately assign the cause of the relevant emergency hospital admission to the patient's subsequently diagnosed cancer, we would expect lower percentages of emergency presentations than percentages calculated according to our definition.^4^ However, the observed large and highly consistent associations (across jurisdictions and studied cancers) between our measure of emergency presentation and increased mortality strongly support its validity as a marker of clinical severity.

Additional variables that were not examined in our study are probably associated with risk of emergency presentation. Single-country studies indicate that lower socioeconomic status is associated with higher emergency presentation risk, although strong associations by age group and stage at diagnosis prevail even after adjustment for socioeconomic status.^4^, ^20^ Validated cross-country socioeconomic status indicators across all jurisdictions examined do not exist currently. Comparable comorbidity data across most jurisdictions examined are also sparse.^26^ Associations between emergency presentation and increased risk of 12-month mortality might partly reflect patients' underlying comorbidities. However, strong associations between emergency presentation and mortality prevail even after adjustment for comorbidity.^15^, ^21^, ^27^, ^28^ Rural or urban patient residence might also be associated with differential risk of emergency presentation.^29^ Another possible confounder (eg, of associations with age) is screening detection status, particularly in colorectal cancer, although there were no suitable data to enable such an analysis to be done.^10^ Focusing on the studied variables maximised feasibility given data availability.

Information on stage at diagnosis was missing for some patients, particularly in the two jurisdictions (British Columbia and Victoria) in which such data were only available for some cancer sites (appendix p 12). This missing data might have led to either overestimation or underestimation of associations between stage at diagnosis and emergency presentation risk, or of mortality risk estimates by emergency presentation status. However, because the observed associations are large, it is unlikely that such bias would have affected their direction. Further, there are probable associations between emergency presentation and non-examined tumour factors (eg, histological type, tumour grade, or other biomarker status).

Bias in our study could arise from several sources. Potential bias from differential completeness and accuracy of the primary jurisdictional data was mitigated by using information from high-quality data sources and the quality assurance processes employed when deriving analysis datasets (as described in the Methods). Variable completeness of information on disease stage at diagnosis between the jurisdictions might bias some of the studied associations, but we did analyses both with and without adjustment for stage at diagnosis. Incomplete follow-up might bias associations with mortality, but in all participating registries, follow-up to 1 year was highly complete. Potential biases that could have arisen from recording inaccuracies (eg, if using cause-specific mortality, or if restricting to emergency hospital admissions with coded diagnosis of cancer) have been obviated by considering all-cause mortality and emergency hospital admission for any reason.

By their nature, ecological analyses, such as jurisdiction-level associations between survival and percentage of emergency presentation, do not encompass patient-level variables, and effective sample size reflects the number of jurisdiction-level observations (n=17 in our context).^30^ Consequently, CIs around our observed central estimates are wide and associations might be stronger or weaker than observed. Further, the absence of evidence of an association for oesophageal and rectal cancers might reflect genuinely null effects or an underpowered study. Nonetheless, overall, the findings support the hypothesis that varying emergency presentation percentages are a source of substantial variation in international cancer survival differences for at least six cancers. This hypothesis should be examined further in comparative survival studies incorporating emergency presentation status among other patient-level variables.

The findings suggest that operationally defining emergency presentation as diagnosis of cancer within 30 days after an emergency hospital admission has construct validity, given its consistent cross-jurisdictional associations with cancer site, age, and stage at diagnosis; and its consistent associations with higher mortality. How patient and health-care factors associated with the risk of emergency presentation might vary between different jurisdictions needs to be quantified; examining emergency presentation subtypes can reveal patient or health-care factors amenable to improvement efforts.^17^ Improvements in survival that can result from earlier diagnosis require accompanying health system investment to ensure effective treatment.

This study documents the feasibility of enriching cancer registration with hospital admissions data to enhance cancer surveillance both within jurisdictions and internationally, at least in countries served by both population-based cancer registries and hospital admissions data sources. Although population-based hospital record datasets exist in most high-income countries, they are rarely used to support routine monitoring of cancer diagnoses and treatment. Given organisational differences in diagnostic services and pathways across countries, operational definitions to capture diagnostic routes other than emergency presentation can additionally be developed in different jurisdictions.^9^, ^15^, ^20^ Diagnosis of cancer through an emergency presentation, however, is probably a global phenomenon; approaches to enable its study in countries without developed population-based hospital admissions data sources need to be developed.

Emergency presentation status can be incorporated into routine cancer surveillance as an important non-stage prognosticator to help monitor cancer control progress. We advocate the need for understanding inequalities in the risk of emergency presentation within different country populations, and research to quantify how tumour, patient, and health-care factors associated with emergency presentation and its adverse prognostic implications might vary between jurisdictions. Routine monitoring of emergency presentations can underpin public health and health system improvement efforts to reduce cancer outcome inequalities, not only between, but also within, jurisdictions.

In conclusion, we show that emergency presentations are frequent, have adverse prognostic implications, and probably contribute to international differences in cancer survival; these observations emphasise the need to reduce the proportion of patients diagnosed through this route, to support cancer control efforts globally.

## Data sharing

The following analytical resources are available on request from the authors: an Excel spreadsheet describing the analysis dataset structure (data items and properties)–exemplified for a jurisdiction; a Word document write-out of SQL code that can be used and adapted to generate analysis-ready datasets; *R* Markdown quality assurance code for analysis datasets; *R* Markdown output of quality assurance of analysis dataset for an exemplar jurisdiction; R Markdown analysis code designed to be run against analysis datasets and produce aggregate data outputs in CSV files; and *R* code used to do the meta-analyses. Most of the aggregate data are shared in the main text or the appendix. Primary data used in this study can be requested following the data access policies of data owner organisations listed in the Contributors section. Additionally, for data from Ontario, Ontario Health is prohibited from making the data used in this research publicly accessible if they include potentially identifiable personal health information or personal information as defined in Ontario law, specifically the Personal Health Information Protection Act and the Freedom of Information and Protection of Privacy Act. Upon request, data de-identified to a level suitable for public release might be provided. For data from Denmark, the data supporting the findings of this study are stored and maintained electronically at Statistics Denmark. The data are not publicly available due to the Danish legislation on data privacy.

## Declarations of interests

OB and GM declare salary compensation for analysis of trial data in preparation for review by the Data Safety Monitoring Board for the POWERRANGER trial (NCT01404156), unrelated to this project. BD declares project funding from the Velux Foundation (Velux Fonden) and the Nordic Collaboration Cancer Care Pathways, for the Danish Cancer Society. ATG reports support to her employer (Queen's University Belfast) from the Public Health Agency for Northern Ireland. DWD declares support to his employer (Northern Ireland Cancer Registry) from the Public Health Agency for Northern Ireland. DWH declares project support from Public Health Wales NHS Trust and Macmillan Cancer Support, and personal payment for research consultancy fees from Pfizer for work done by Swansea University concerning value-based health care, unrelated to this work. CGCAJ declares past part-employment by the Cancer Society of New Zealand, who co-sponsors the project. HJ declares research grant funding from the Velux Foundation (Velux Fonden). BM and YN declare research grant funding to the Cancer Registry of Norway from the Norwegian Cancer Society. RST declares project support to Public Health Wales NHS Trust from the NHS Wales Cancer Network and Macmillan Cancer Support. RRW reports research grant funding from the Michael Smith Foundation for Health Research, and the First Nations Health Authority and Canadian Partnership Against Cancer. GL declares research grant funding from the study sponsors to his employer (University College London).
