# Supplementary material for: Risk factors and prognostic implications of diagnosis of cancer within 30 days after an emergency hospital admission (emergency presentation): an International Cancer Benchmarking Partnership (ICBP) population-based study
Source: Lancet Oncol. 2022 May;23(5):587–600. doi: 10.1016/S1470-2045(22)00127-9 (PMC9046095; doi:10.1016/S1470-2045(22)00127-9)
Supplement: Supplementary appendix [file mmc1.pdf]

THE LANCET
Oncology

Supplementary appendix

This appendix formed part of the original submission and has been peer reviewed. We post it as supplied by the authors.

Supplement to: McPhail S, Swann R, Johnson SA, et al. Risk factors and prognostic implications of diagnosis of cancer within 30 days after an emergency hospital admission (emergency presentation): an International Cancer Benchmarking Partnership (ICBP) population-based study. *Lancet Oncol* 2022; published online April 6. https://doi.org/10.1016/S1470-2045(22)00127-9.

Supplementary Materials for "Risk factors and prognostic implications of diagnosis of cancer within 30 days after an emergency hospital admission (emergency presentation): An International Cancer Benchmarking Partnership (ICBP) population-based study"

Supplementary Material 1.

A. Data sources and operational definitions used to identify emergency presentation (defined as diagnosis of cancer within 30 days after an emergency hospital admission) in participating jurisdictions. Superscript indicators ‘b’ and ‘n’ denote whether ‘broad’ / ‘narrow’ definitions used (see main paper-Methods, and ‘B’ below).

Jurisdiction	Source of hospitalisation data upon which cancer registration data were linked	Hospital inpatient admission codes used to emergency hospitalisation
**Denmark**[n]	Danish National Patient Registry (NPR) dataset	Inpatient admission coded with admission type: “1”
**Norway**[b]	Norwegian Patient Registry (NPR)	Episodes registered with an urgency code “Acute”
**England**[n]	Hospital Episodes Statistics (HES)	Inpatient admission coded with admission type: “21; 22; 23; 24; 28; 2A; 2B; 2D”
**Northern Ireland**[n]	Patient Administration System (PAS)	Inpatient method of admission category coded as “Emergency”
**Scotland**[b]	Scottish Morbidity Record 01 (SMR01)	Any record of inpatient admission coded with admission type “30-36, 38, 39” within the same Continuous Inpatient Stay (CIS)
**Wales**[b]	Patient Episode Database for Wales (PEDW)	Admission method codes "21-25, 27, 28”
**Canadian Provinces**[b] (Alberta, Atlantic Canada, British Columbia, Ontario, Saskatchewan-Manitoba)	Canadian Institutes for Health Information Discharge Abstract Database (DAD)	Inpatient admissions coded with an emergency entry code (“E”)
**New South Wales**[n]	NSW Admitted Patient Data Collection (APDC)	Inpatient admission coded with admission type: “Emergency”
**Victoria**[n]	Victorian Admitted Episodes Dataset (VAED)	Admission type: "C" or "O"
**New Zealand**[b]	New Zealand National Minimum Data Set (NDMS) (hospital events)	Inpatient admission coded with admission type: “AC”

B. Figure. Factors likely to be implicated in emergency presentations

Reproduced from Zhou Y, Abel GA, Hamilton W, Pritchard-Jones K, Gross CP, Walter FM, Renzi C, Johnson S, McPhail S, Elliss-Brookes L, Lyratzopoulos G. Diagnosis of cancer as an emergency: a critical review of current evidence. Nat Rev Clin Oncol. 2017;14(1):45-56. doi: 10.1038/nrclinonc.2016.155. Epub 2016 Oct 11. PMID: 27725680. https://www.nature.com/articles/nrclinonc.2016.155 (accessed Feb 17, 2022) – main text reference 4.

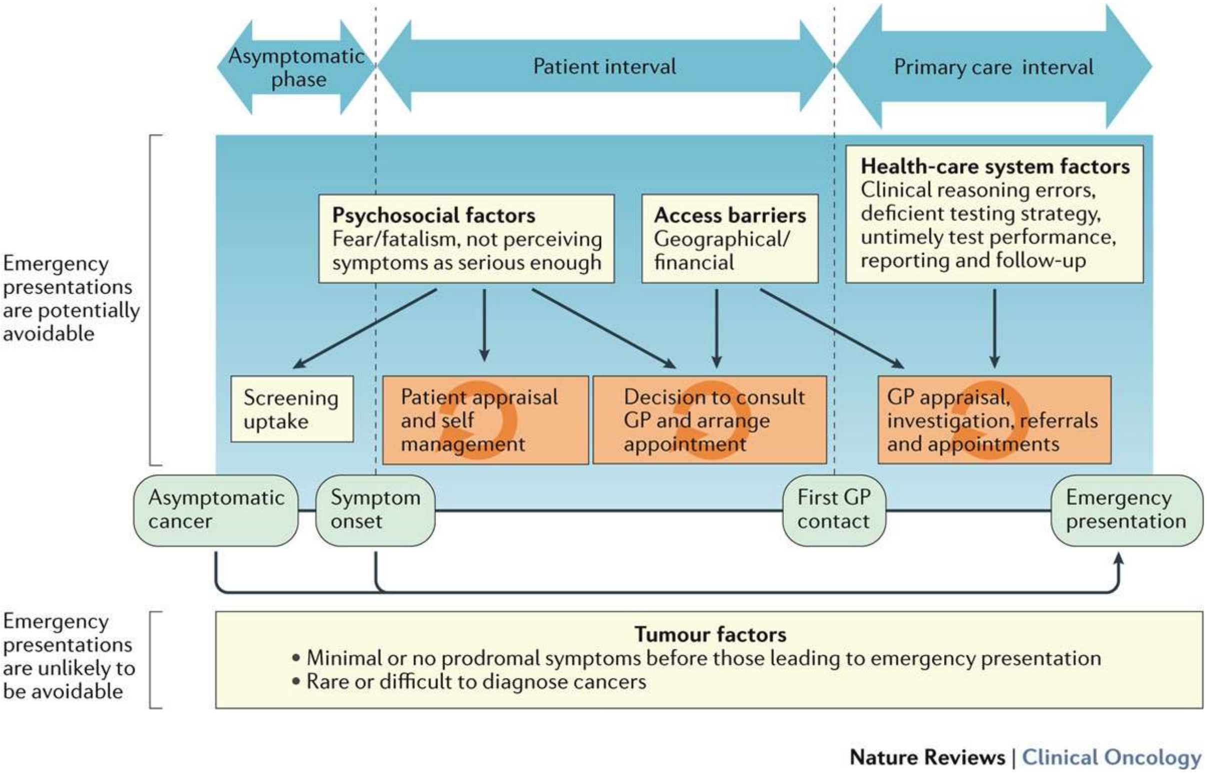

Tumour factors: Likely to chiefly contribute in the context of inherently aggressive tumours with minimal or no prodromal symptoms. For certain cancer sites (e.g. colorectal) the incidence of inherently aggressive tumours may be prevented *ab initio* through participation to screening, through the detection and resection of adenomatous polyps which would otherwise evolve into invasive tumours. However, for most other cancer sites such events may be deemed largely unavoidable with currently available diagnostic technologies and existing screening programmes.

Patient factors: Likely to chiefly contribute in patients with prolonged intervals from symptom onset to help-seeking, or regarding participation to screening. Public health educational interventions raising awareness of possible cancer symptoms, or aimed at reducing cancer fear or fatalism, and increasing participation to screening programmes where available, may help to reduce such occurrences.

Healthcare factors: Likely to chiefly contribute in patients who experience prolonged intervals post-presentation, either because their disease progressed making an emergency presentation necessary while awaiting planned investigations, or because the diagnosis of cancer was not suspected and no investigations instigated. Clinical guidelines for specialist assessment and timely access to specialist investigations may help to minimise such risks, though both tumour (e.g. atypical presenting symptoms and/or inherent tumour aggressiveness) and patient factors (e.g. inability to attend for investigations) remain important.

C. Schematic representation of definition of emergency presentation (including 'broad' and 'narrow' definition subtypes)

The construct underlying the operational definition of Emergency Presentation (EP) is the occurrence of emergency hospital admission (i.e. emergency inpatient care) during the 30-day period before the diagnosis of cancer. No information about the diagnostic coding relating to the relevant admission episode is required.

This definition is by nature agnostic to actual clinical circumstances that prompted the emergency hospitalisation. To increase the likely specificity of our operational definition as a marker of clinical emergency, it can be required that the relevant emergency hospitalisation event has occurred without an intervening elective hospital admission until the diagnosis date (in the subsequent 30-day period; see Diagram below – particularly 'Scenario 3'). Where this additional requirement was applied we refer to it as the 'narrow' definition, if it is not we refer to it as the 'broad' definition. For example, consider that a hypothetical cancer patient has an emergency hospital admission at -20 days pre-diagnosis, and a subsequent elective admission at -10 days. This patient will not be classified as an EP if the 'narrow' definition is used (requiring the emergency hospitalisation to be proximal) but will be classed as EP if the 'broad' definition is used (not placing any condition on whether emergency hospitalisation during the 30-days pre-diagnosis was or not proximal).

The two definitions are expressions of the same underlying construct and, in our study, we have found both to have similar associations between age group, sex and cancer site and risk of EP (see Table below summarising pooled estimates derived from meta-regression); and also between EP and 12-month mortality (odds ratio 2·86 (2·65-3·08) for jurisdictions using the broad definition, and 3·09 (2·78-3·43) for those using the narrow definition; see also main paper - Figure 1, and appendix p.61.

Table. Comparison of adjusted associations between age group, sex and cancer site and risk of diagnosis through an emergency presentation, by emergency presentation definition type.
See also appendix pp.36-42

	**Variable**	**Pooled estimate across jurisdictions using a broad definition***	**Pooled estimate across jurisdictions using a narrow definition***
**Age group**	15-64	0·99 (0·94-1·03)	1·04 (0·98-1·10)
	65-74	*Reference*	*Reference*
	75-84	1·35 (1·30-1·40)	1·43 (1·36-1·49)
	85-99	2·40 (2·22-2·59)	2·65 (2·39-2·94)
**Sex**	Men	*Reference*	*Reference*
	Women	0·99 (0·96-1·03)	1·02 (0·98-1·06)
**Cancer site**	Oesophageal	0·78 (0·69-0·88)	0·81 (0·69-0·95)
	Stomach	1·27 (1·14-1·41)	1·17 (1·02-1·34)
	Colon	*Reference*	*Reference*
	Rectal	0·37 (0·34-0·40)	0·36 (0·32-0·39)
	Liver	1·58 (1·40-1·78)	1·83 (1·56-2·15)
	Pancreatic	2·02 (1·76-2·32)	2·18 (1·81-2·63)
	Lung	1·28 (1·15-1·43)	1·46 (1·27-1·70)
	Ovarian	1·22 (1·05-1·42)	1·08 (0·88-1·33)

*Estimates shown are adjusted for variables shown in the table, and year of diagnosis.

Applying the narrow definition changes the EP status classification of a relatively small group of patients who had both elective and emergency hospital admissions during the 30-day period pre-diagnosis. Jurisdictions using a 'broad' definition tend to have a higher % of EP in general, because some patients would not have been classified as EPs had the narrow definition been used (see Diagram, 'Scenario 3'). Similarly, jurisdictions using a 'narrow' definition tend to have a lower % of EP – see example Table for two jurisdictions below. As above, associations between EP and patient-level variables, and between EP and mortality, are similar, independently of the definition type used.

Table. Impact of using 'broad' or 'narrow' definition in the same jurisdiction. 'Broad' definitions assign a greater number of cases as having been diagnosed as EPs, compared to 'narrow' definitions.

	**Oesophageal**	**Stomach**	**Colon**	**Rectal**	**Liver**	**Pancreatic**	**Lung**	**Ovarian**	**All sites**
**England–narrow** (as used in main paper)	19·7%	31·4%	29·3%	10·7%	42·4%	47·0%	34·7%	34·8%	31·3%
**England–broad**	23·0%	37·0%	33·9%	13·1%	47·0%	55·5%	41·6%	41·7%	37·0%

	Oesophageal	Stomach	Colon	Rectal	Liver	Pancreatic	Lung	Ovarian	All sites
**New South Wales–narrow** (as used in main paper)	26·9%	30·5%	27·6%	12·1%	36·8%	45·3%	36·4%	28·4%	30·9%
**New South Wales–broad**	31·2%	34·8%	32·4%	14·6%	40·1%	50·4%	40·2%	34·8%	35·2%

A further illustration is provided in the Table below, to help appreciate the above presented data in the context of all jurisdictions.

Table: Impact of using ‘broad’ or ‘narrow’ definition in the same jurisdiction (England and New South Wales) in the context of all jurisdiction estimates. ‘Broad’ definitions’ assign a greater number of cases as having been diagnosed as EPs, compared to ‘narrow’ definitions.

	Oesophageal	Stomach	Colon	Rectal	Liver	Pancreatic	Lung	Ovarian	All sites
**Denmark–narrow**	24·3%	32·2%	28·3%	13·5%	43·3%	48·0%	36·4%	20·6%	30·9%
**Norway–broad**	32·7%	40·0%	35·8%	15·8%	50·6%	55·4%	39·4%	34·9%	36·5%
**England–narrow**	19·7%	31·4%	29·3%	10·7%	42·4%	47·0%	34·7%	34·8%	31·3%
**England–broad**	23·0%	37·0%	33·9%	13·1%	47·0%	55·5%	41·6%	41·7%	37·0%
**Northern Ireland–narrow**	19·9%	29·6%	23·8%	9·1%	36·9%	42·7%	32·0%	29·6%	27·9%
**Scotland–broad**	25·2%	39·1%	35·1%	14·1%	47·5%	59·2%	42·1%	42·8%	38·5%
**Wales–broad**	25·5%	39·1%	34·2%	13·8%	50·5%	56·7%	41·5%	40·8%	37·4%
**Alberta–broad**	23·0%	34·6%	28·6%	12·9%	31·7%	40·9%	33·3%	25·7%	30·0%
**Atlantic Canada–broad**	20·7%	30·4%	28·4%	12·6%	34·3%	38·8%	26·6%	28·1%	26·9%
**British Columbia–broad**	28·3%	40·3%	27·5%	12·0%	38·1%	41·4%	33·2%	31·7%	30·5%
**Ontario–broad**	20·3%	26·9%	27·2%	11·0%	28·3%	35·3%	27·5%	23·9%	26·1%
**Saskatchewan-Manitoba–broad**	18·5%	31·8%	27·7%	13·7%	33·3%	37·3%	30·6%	30·1%	28·3%
**New South Wales–narrow**	26·9%	30·5%	27·6%	12·1%	36·8%	45·3%	36·4%	28·4%	30·9%
**New South Wales–broad**	31·2%	34·8%	32·4%	14·6%	40·1%	50·4%	40·2%	34·8%	35·2%
**Victoria–narrow**	20·0%	23·7%	22·9%	9·1%	31·9%	34·1%	27·8%	20·9%	24·0%
**New Zealand–broad**	36·8%	47·8%	36·6%	19·8%	49·7%	60·4%	51·1%	48·1%	42·5%

Diagram:

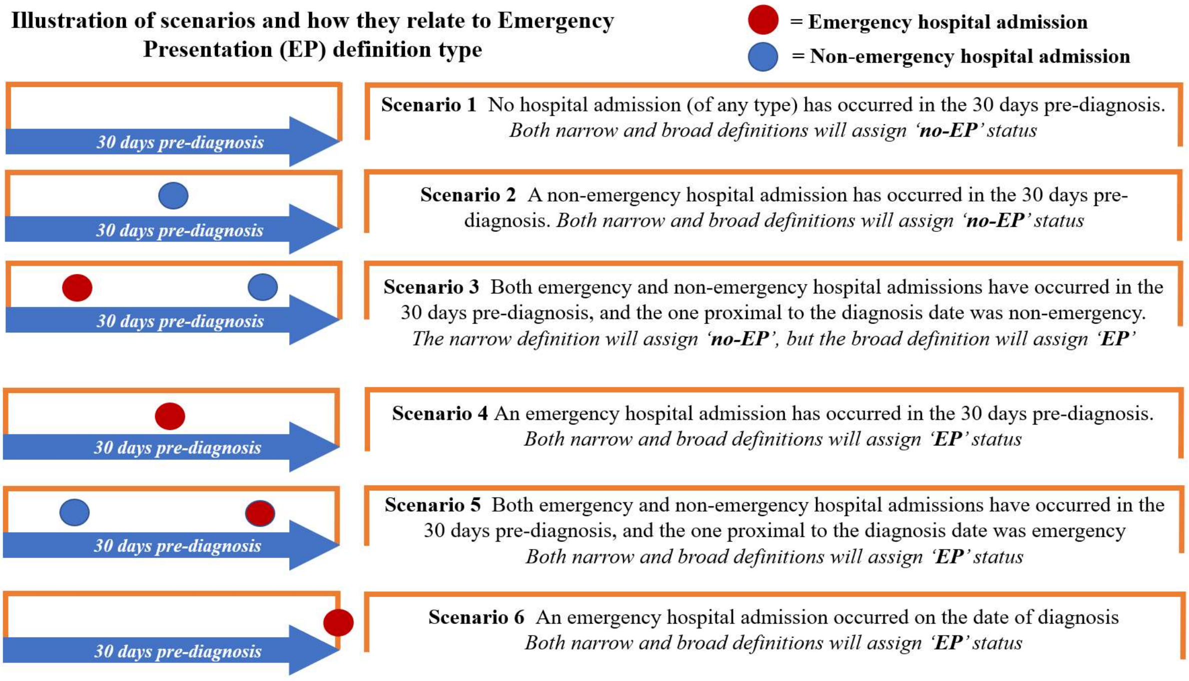

D. Exploration of yield of alternative cut-off periods in the English data

A 30-day cut-off was chosen for the period preceding the diagnosis of cancer during which the emergency hospital admission defining the emergency presentation ought to have occurred. This was deemed an appropriately short period during which histological diagnosis of cancer, which typically requires an interval of few weeks' duration until a biopsy or excision sample are obtained and processed by the pathology lab, could take place. Additionally, 30-day intervals are used in other established healthcare statistics (e.g. as in '30-day postoperative mortality' or '30-day hospital readmission'). However, we have additionally explored cut-offs at 60 and 90 days in the English data, which indicated that only few cancer patients would have been additionally classified as having been diagnosed through an emergency presentation if such cut-off values were used (see Table). This suggests that in cancer patients who experience an emergency hospital admission shortly before their diagnosis, the emergency hospitalisation event typically leads to prompt subsequent diagnostic resolution (i.e. diagnosis of cancer within 30 days), which is to be expected.

Table: % of cancer patients with an emergency hospital admission within the 30-days (as used in main analysis) or within 60- and 90-days pre-diagnosis in the England analysis sample; and comparison with English Routes-to-Diagnosis emergency presentation % on the same analysis sample.

	30 days cut-off (as used in main analysis)	60-day cut-off	90-day cut-off	RTD-defined EP
**Oesophageal**	19.7%	19.9%	20.0%	19.2%
**Stomach**	31.4%	31.9%	32.0%	30.6%
**Colon**	29.3%	29.8%	30.0%	28.9%
**Rectal**	10.7%	11.0%	11.1%	10.8%
**Liver**	42.4%	43.9%	44.6%	41.0%
**Pancreatic**	46.9%	47.8%	48.1%	44.7%
**Lung**	34.7%	35.9%	36.6%	34.7%
**Ovarian**	34.8%	35.2%	35.4%	31.0%
**All**	31.3%	32.1%	32.5%	30.7%

EP: Emergency Presentation; RTD: Routes-to-Diagnosis.

In the England sub-cohort, our 30-day cut-off definition also provides similar percentage of emergency presentations to that derived by the algorithmically produced English Routes to Diagnosis definition. No attempt was made to use the latter definition as it requires several additional data linkages only applicable to the English health system.

Supplementary Material 2.

A. Statistical analysis additional references:

The following references relate to software used in analysis (see main text-Methods).

- Wickham et al., (2019). Welcome to the tidyverse. Journal of Open Source Software, 4(43), 1686, https://doi.org/10.21105/joss.01686
- Richard Iannone, Joe Cheng and Barret Schloerke (2021). gt: Easily Create Presentation-Ready Display Tables. R package version 0.3.0. https://CRAN.R-project.org/package=gt
- Daniel D. Sjoberg, Michael Curry, Margie Hannum, Joseph Larmarange, Karissa Whiting and Emily C. Zabor (2021). gtsummary: Presentation-Ready Data Summary and Analytic Result Tables. R package version 1.4.2. https://CRAN.R-project.org/package=gtsummary
- Joseph Larmarange (2021). labelled: Manipulating Labelled Data. R package version 2.8.0. https://CRAN.R-project.org/package=labelled

B. Analysis sample description and derivation (across the 8 cancer sites studied)

	Study years	Cases identified (15-99 years of age)	Sex not male/ female	Discordant sex-site combination	Death Certificate Only (DCO)	Diagnosis of other primary cancer*	Cases in analysis	% excluded
**Denmark** [n]	2014-17	53,697	383	13	437	6,861	46,003	14·3%
**Norway** [b]	2012-16	49,237	0	85	625	2,380	46,147	6·3%
**England** [n]	2013-16	419,643	1	123	4,916	41,409	373,194	11·1%
**Northern Ireland** [n]	2012-16	17,302	0	0	103	1,698	15,501	10·4%
**Scotland** [b]	2014-17	50,886	0	13	135	5,709	45,029	11·5%
**Wales** [b]	2013-16	27,875	0	10	636	1,090	26,139	6·2%
**Alberta^** [b]	2012-15	23,012	0	21	…	…	19,969	13·2%
**Atlantic Canada^** [b $]	2012-15	22,445	…	…	…	…	22,125	1·4%
**British Columbia^** [b $]	2012-15	34,110	…	…	…	…	33,655	1·3%
**Ontario** [b]	2012-16	116,498	0	376	10,326	13,337	92,447	20·6%
**Saskatchewan-Manitoba^** [b $]	2012-15	16,840	…	…	…	…	16,655	1·1%
**New South Wales** [n]	2012-16	59,998	0	0	1,204	5,596	53,198	11·3%
**Victoria** [n]	2012-16	43,713	0	6	1,423	4,072	38,212	12·6%
**New Zealand** [b]	2012-15	29,363	6	51	512	0	28,794	1·9%
**Total**		964,619	390	698	20,317	82,152	857,068	11·1%

Superscript indicators ‘n’ and ‘b’ describe whether a narrow or broad operational definition of emergency presentation was used – please also see main paper-Methods, and appendix pp.4-5.
* During 5 years prior to 1 year after index cancer diagnosis, or during the study period (the latter was applied in Atlantic Canada, British Columbia, Ontario, Saskatchewan-Manitoba).
"…": Denotes data suppression due to small counts.
^ A different analysis sample was used to examining adjusted associations with mortality, specifically, 25,182 for Alberta (cases diagnosed 2012-16), 10,970 for Atlantic Canada (2012-13), 16,490 for British Columbia (2012-13), and 8,385 for Saskatchewan-Manitoba (2012-13), reflecting different study years for which mortality follow-up was available.
$ To comply with requirements for minimising residual disclosure risk, all actual counts for these jurisdictions were randomly rounded to a higher or lower multiple of 5 within cells. Sample sizes used in different aspects of the analysis may vary minimally for this reason.

	Analysed cases sample size (for predictors)	Study years (for predictors)	Analysed cases sample size / crude mortality	Study years (for crude mortality)	Analysed cases (mortality regression)	Study years (for mortality regression)
**Denmark** [n]	46,003	2014-2017	46,003	As column 3	46,003	As column 3
**Norway** [b]	46,147	2012-2016	46,147	As column 3	46,147	As column 3
**England** [n]	373,194	2013-2016	373,194	As column 3	373,194	As column 3
**Northern Ireland** [n]	15,501	2012-2016	15,501	As column 3	15,501	As column 3
**Scotland** [b]	45,029	2014-2017	45,029	As column 3	45,029	As column 3
**Wales** [b]	26,139	2013-2016	26,139	As column 3	26,139	As column 3
**Alberta^** [b]	19,969	2012-2015	9,440	2012-2013	25,182	2012-2016
**Atlantic Canada^** [b]	22,125	2012-2015	10,970	2012-2013	10,970	As column 5
**British Columbia^** [b]	33,655	2012-2015	16,490	2012-2013	16,490	As column 5
**Ontario** [b]	92,447	2012-2016	92,447	As column 3	92,447	As column 3
**Saskatchewan-Manitoba^** [b]	16,655	2012-2015	8,385	2012-2013	8,385	As column 5
**New South Wales** [n]	53,198	2012-2016	53,198	As column 3	53,198	As column 3
**Victoria** [n]	38,212	2012-2016	38,212	As column 3	38,212	As column 3
**New Zealand** [b]	28,794	2012-2015	28,794	As column 3	28,794	As column 3

Superscript indicators ‘n’ and ‘b’ describe whether a narrow or broad operational definition of emergency presentation was used – please also see main paper-Methods, and appendix pp.4-5.

Supplementary Material 3. Analysis sample composition, by cancer site, age group, sex, within each jurisdiction

The composition of analysis samples in each jurisdiction was generally similar regarding cancer site, age group and sex. For example, considering cancer site, the percentage of patients with pancreatic cancer ranged between 6.5% (Atlantic Canada) to 8.5% (New South Wales), and with rectal cancer from 7.9% (Scotland) to 13.1% (Denmark). Regarding age groups, the percentage of patients aged 85 years and over varied from 7.7% (Denmark) to 13.7% (England). The sex composition of analysed patients was highly similar between jurisdictions (with/without inclusion of ovarian cancer from such calculations).

A. Analysis sample composition by cancer site

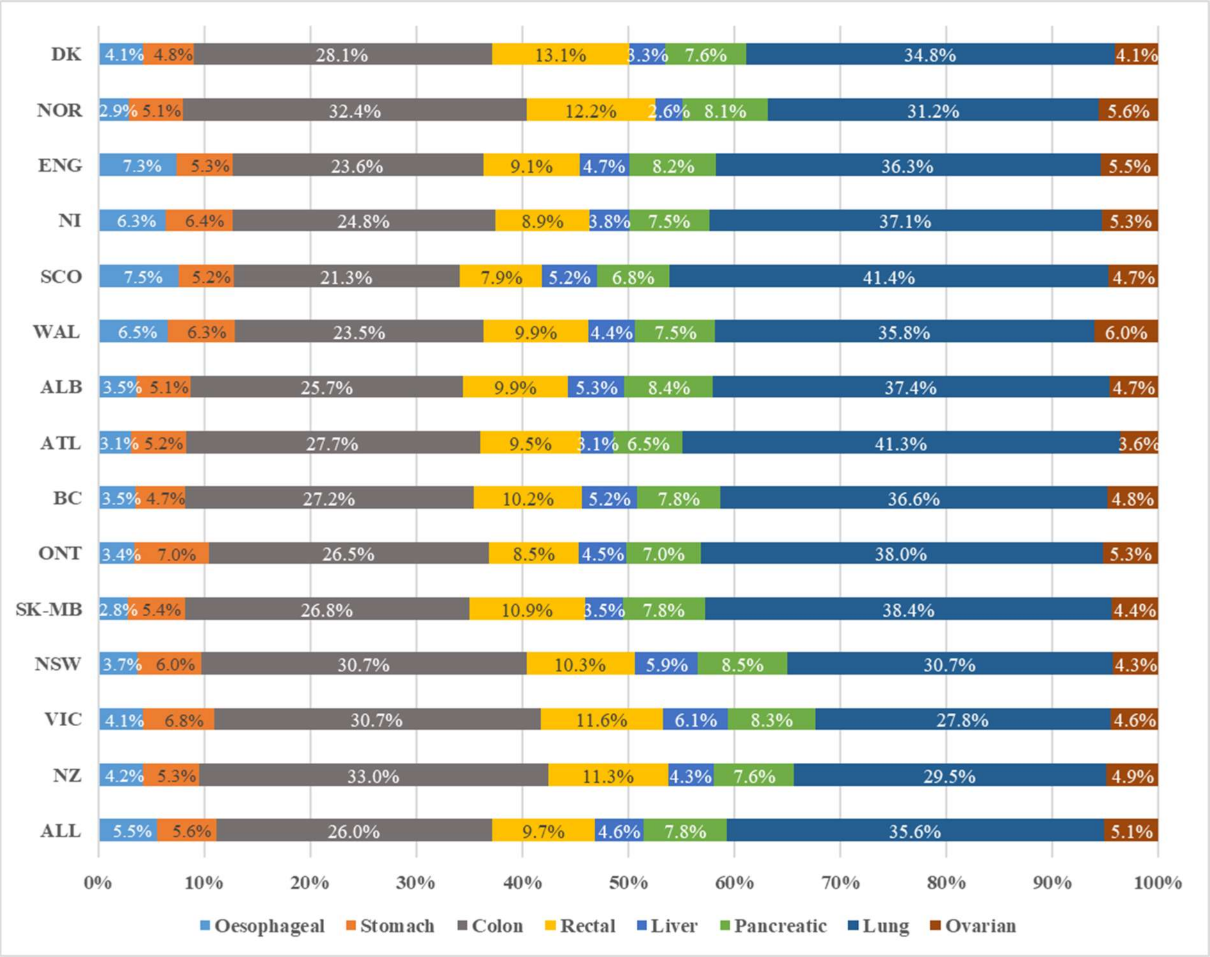

DK: Denmark; NOR: Norway; ENG; England; NI: Northern Ireland; SCO: Scotland; WAL: Wales; ALB: Alberta; ATL: Atlantic; BC: British Columbia; ONT: Ontario; SK-MB: Saskatchewan-Manitoba; NSW: New South Wales; VIC: Victoria; NZ: New Zealand; ALL: All jurisdictions.

B. Analysis sample composition by age group

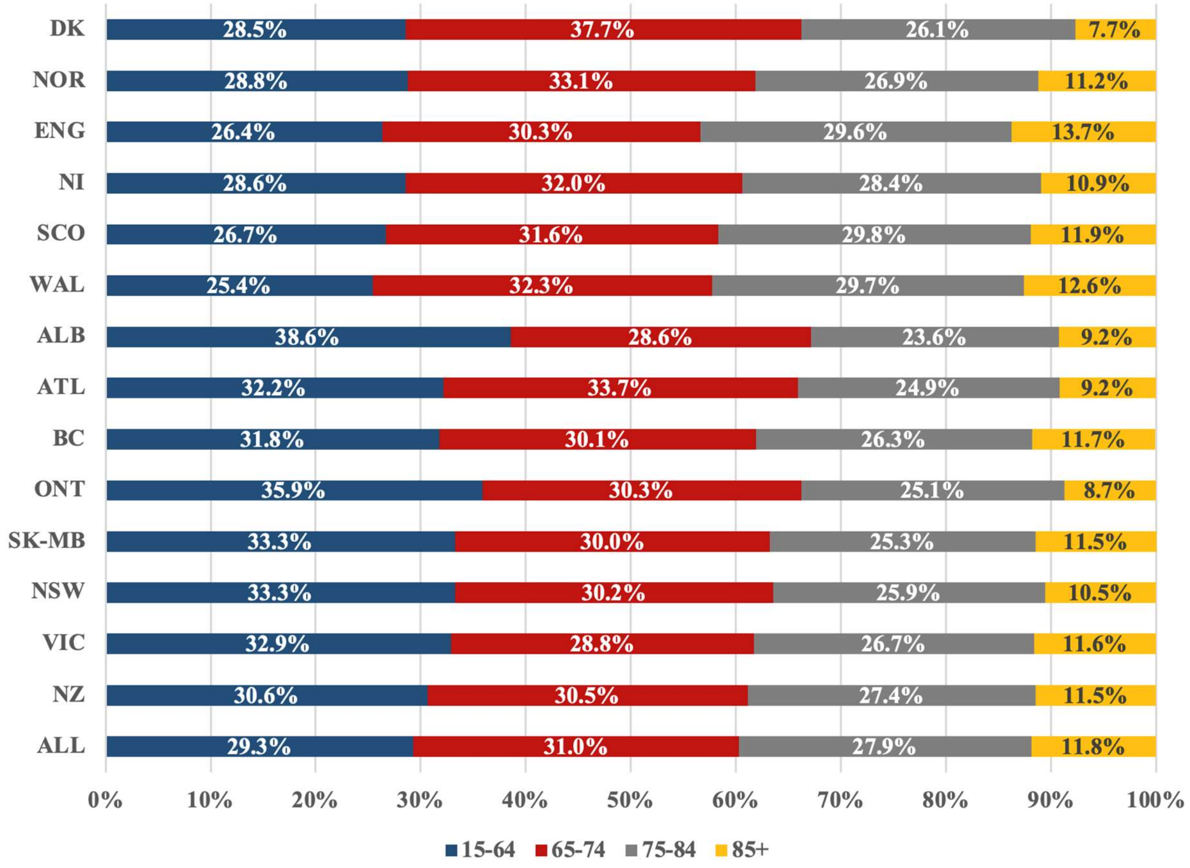

DK: Denmark; NOR: Norway; ENG; England; NI: Northern Ireland; SCO: Scotland; WAL: Wales; ALB: Alberta; ATL: Atlantic; BC: British Columbia; ONT: Ontario; SK-MB: Saskatchewan-Manitoba; NSW: New South Wales; VIC: Victoria; NZ: New Zealand; ALL: All jurisdictions.

C. Analysis sample composition by sex

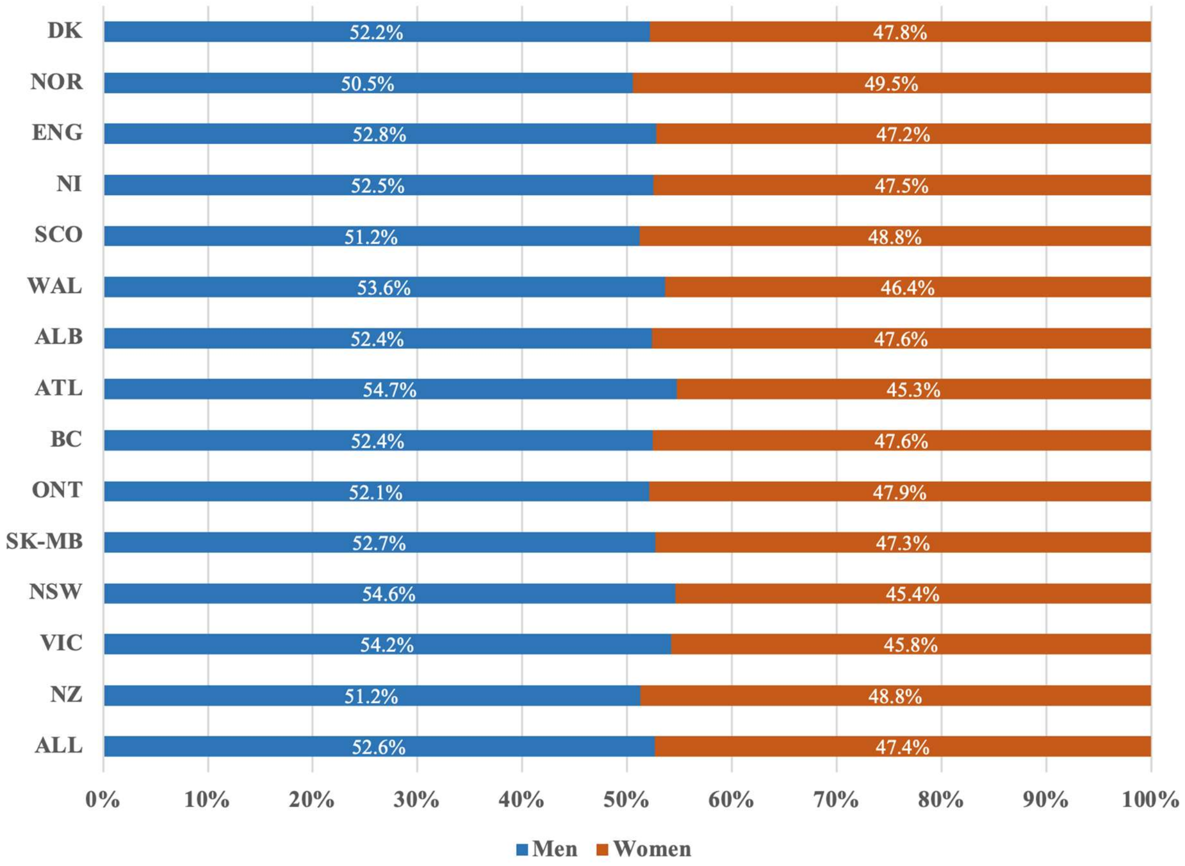

DK: Denmark; NOR: Norway; ENG; England; NI: Northern Ireland; SCO: Scotland; WAL: Wales; ALB: Alberta; ATL: Atlantic; BC: British Columbia; ONT: Ontario; SK-MB: Saskatchewan-Manitoba; NSW: New South Wales; VIC: Victoria; NZ: New Zealand; ALL: All jurisdictions.

Supplementary Material 4. Description of stage completeness

Stage completeness (% included cases) by jurisdiction and cancer site

	All cancers	Oesophagus	Stomach	Colon	Rectal	Liver	Pancreatic	Lung	Ovarian
**Denmark [n]**	75%	64%	68%	67%	61%	57%	72%	94%	68%
**Norway [b]**	88%	65%	71%	94%	89%	65%	80%	89%	94%
**England [n]**	84%	81%	75%	88%	90%	42%	73%	91%	83%
**Northern Ireland [n]**	85%	70%	68%	90%	94%	54%	76%	91%	88%
**Scotland [b]**	85%	77%	71%	85%	85%	57%	81%	92%	87%
**Wales [b]**	83%	83%	80%	88%	90%	31%	69%	91%	72%
**Alberta [b]**	95%	85%	87%	95%	93%	89%	94%	99%	97%
**Atlantic Canada [b]**	89%	66%	54%	96%	94%	51%	58%	98%	65%
**British Columbia [b]**	…	...	...	90%	83%	...	...	94%	...
**Ontario [b]**	83%	53%	41%	93%	91%	48%	58%	96%	67%
**Saskatchewan-Manitoba [b]**	95%	89%	87%	95%	93%	70%	95%	99%	97%
**New South Wales [n]**	88%	82%	82%	93%	86%	78%	86%	86%	93%
**Victoria [n]**	...	...	...	90%	79%	...	...	...	...
**New Zealand [b]**	70%	36%	55%	87%	56%	38%	66%	67%	92%
**Average across jurisdictions**		76%	69%	89%	78%	50%	73%	92%	78%

Superscript indicators 'n' and 'b' describe whether a narrow or broad operational definition of emergency presentation was used – please also see main paper-Methods and appendix pp.4-5.
"…": Denotes that no data were submitted for stage

Supplementary Material 5. Correlations of % of emergency presentations (defined as diagnosis of cancer within 30 days after an emergency hospital admission) by cancer site across jurisdictions. 13 pair-wise correlation values are shown (against England, chosen as the jurisdiction with the largest sample size).

Two-country comparison (pairwise correlations of cancer site-specific percentages of emergency presentation)	Pearson *r* *
England - Denmark	0·88
England - Norway	0·95
England - Northern Ireland	0·99
England - Scotland	0·99
England - Wales	1·00
England - Alberta	0·90
England - Atlantic Canada	0·97
England - British Columbia	0·88
England - Ontario	0·93
England - Saskatchewan-Manitoba	0·97
England - New South Wales	0·93
England - Victoria	0·94
England - New Zealand	0·94

* For all comparisons shown, $p<0.002$

Whether differences in emergency presentation frequency between the studied cancer sites were consistent across the jurisdictions was examined by correlating the cancer-site specific percentage of emergency presentations in England, the participating jurisdiction with the largest sample size, with the corresponding percentages in each other jurisdiction, resulting in 13 pair-wise correlations each containing eight pairs of observations.

Supplementary Material 6.

A. **Figure. Percentage of patients diagnosed through emergency presentation (defined as diagnosis of cancer within 30 days after an emergency hospital admission) by age group, sex, and stage at diagnosis. Data visualised in this figure are included in appendix pp.15-28.** For jurisdictions using four-group stage classification, the middle groups were aggregated into a single category.

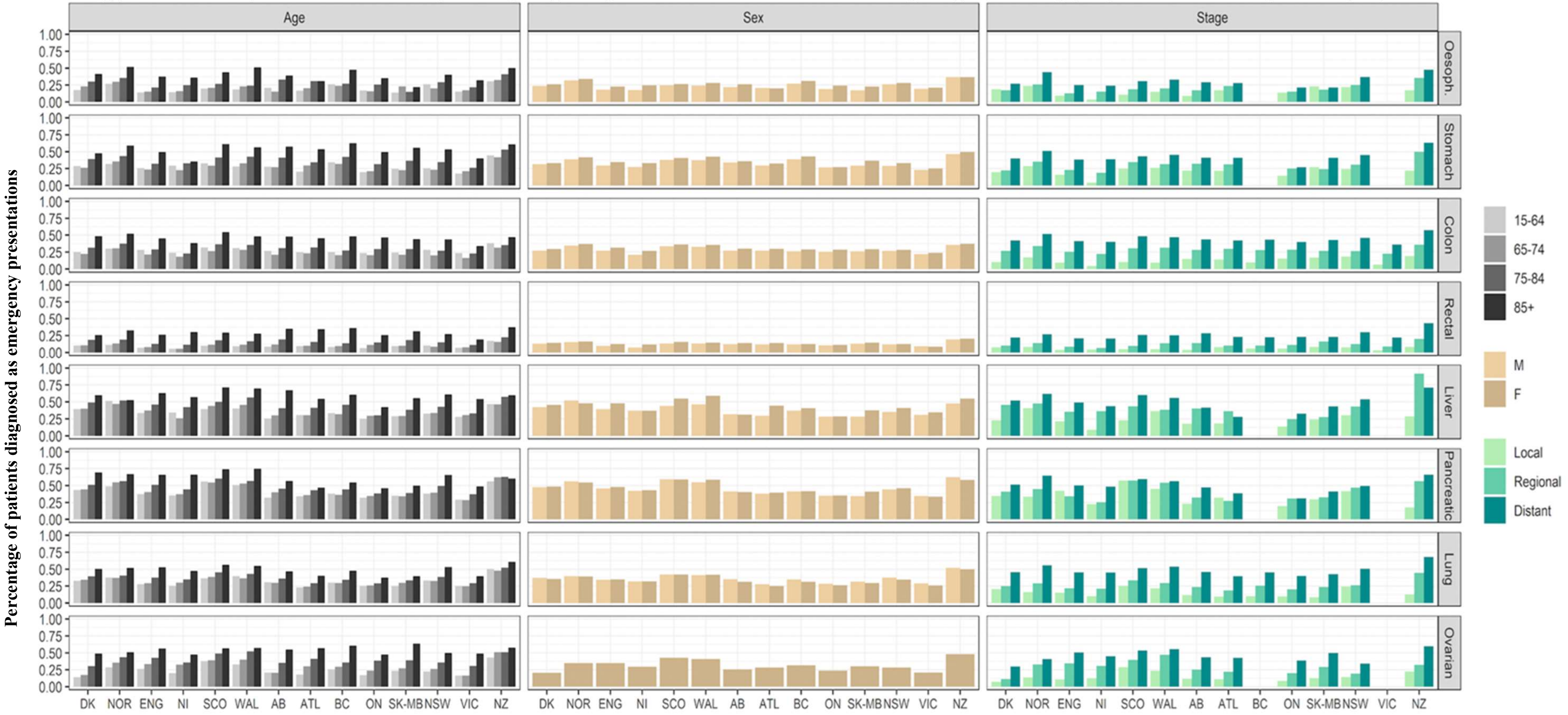

DK: Denmark; NOR: Norway; ENG; England; NI: Northern Ireland; SCO: Scotland; WAL: Wales; ALB: Alberta; ATL: Atlantic; BC: British Columbia; ONT: Ontario; SK-MB: Saskatchewan-Manitoba; NSW: New South Wales; VIC: Victoria; NZ: New Zealand; Oesoph: Oesophageal; M: Male; F: Female.

B. Included patients, and patients who presented through an emergency presentation (defined as diagnosis of cancer within 30 days after an emergency hospital admission), with accompanying % value and 95% confidence intervals, by jurisdiction, cancer site, sex, diagnosis year, age group and stage at diagnosis

Denmark		Oesophageal				Stomach				Colon				Rectal			
		All	EP	%EP	95% CI	All	EP	%EP	95% CI	All	EP	%EP	95% CI	All	EP	%EP	95% CI
	**Total**	1,896	461	24·3%	(22·4%, 26·3%)	2,231	719	32·2%	(30·3%, 34·2%)	12,928	3,655	28·3%	(27·5%, 29·1%)	6,005	811	13·5%	(12·7%, 14·4%)
**Sex**	**Male**	1,373	323	23·5%	(21·4%, 25·8%)	1,477	468	31·7%	(29·4%, 34·1%)	6,643	1,801	27·1%	(26·1%, 28·2%)	3,677	481	13·1%	(12·0%, 14·2%)
	**Female**	523	138	26·4%	(22·8%, 30·3%)	754	251	33·3%	(30·0%, 36·7%)	6,285	1,854	29·5%	(28·4%, 30·6%)	2,328	330	14·2%	(12·8%, 15·7%)
**Diagnosis year**	**2012**	…	…	…	…	…	…	…	…	…	…	…	…	…	…	…	…
	**2013**	…	…	…	…	…	…	…	…	…	…	…	…	…	…	…	…
	**2014**	439	111	25·3%	(21·4%, 29·6%)	547	196	35·8%	(31·9%, 39·9%)	3,098	958	30·9%	(29·3%, 32·6%)	1,570	224	14·3%	(12·6%, 16·1%)
	**2015**	474	113	23·8%	(20·2%, 27·9%)	539	192	35·6%	(31·7%, 39·8%)	3,248	900	27·7%	(26·2%, 29·3%)	1,553	207	13·3%	(11·7%, 15·1%)
	**2016**	477	128	26·8%	(23·1%, 31·0%)	569	179	31·5%	(27·8%, 35·4%)	3,257	910	27·9%	(26·4%, 29·5%)	1,405	189	13·5%	(11·8%, 15·3%)
	**2017**	506	109	21·5%	(18·2%, 25·3%)	576	152	26·4%	(23·0%, 30·1%)	3,325	887	26·7%	(25·2%, 28·2%)	1,477	191	12·9%	(11·3%, 14·7%)
**Age group**	**15-64**	610	107	17·5%	(14·7%, 20·8%)	729	210	28·8%	(25·6%, 32·2%)	3,257	815	25.0%	(23·6%, 26·5%)	2,038	211	10·4%	(9·1%, 11·8%)
	**65-74**	724	168	23·2%	(20·3%, 26·4%)	730	191	26·2%	(23·1%, 29·5%)	4,783	1,065	22·3%	(21·1%, 23·5%)	2,160	230	10·6%	(9·4%, 12·0%)
	**75-84**	425	129	30·4%	(26·2%, 34·9%)	577	225	39.0%	(35·1%, 43·0%)	3,565	1,132	31·8%	(30·2%, 33·3%)	1,348	251	18·6%	(16·6%, 20·8%)
	**85+**	137	57	41·6%	(33·7%, 50%)	195	93	47·7%	(40·8%, 54·7%)	1,323	643	48·6%	(45·9%, 51·3%)	459	119	25·9%	(22·1%, 30·1%)
**Stage**	**1**	106	20	18·9%	(12·6%, 27·4%)	240	47	19·6%	(15·1%, 25·1%)	1,820	188	10·3%	(9·0%, 11·8%)	1,067	83	7·8%	(6·3%, 9·5%)
	**2**	145	25	17·2%	(12·0%, 24·2%)	359	70	19·5%	(15·7%, 23·9%)	2,587	631	24·4%	(22·8%, 26·1%)	822	85	10·3%	(8·4%, 12·6%)
	**3**	448	70	15·6%	(12·6%, 19·3%)	218	49	22·5%	(17·4%, 28·5%)	1,756	472	26·9%	(24·9%, 29·0%)	843	86	10·2%	(8·3%, 12·4%)
	**4**	518	141	27·2%	(23·6%, 31·2%)	707	283	40.0%	(36·5%, 43·7%)	2,435	1,035	42·5%	(40·6%, 44·5%)	906	203	22·4%	(19·8%, 25·2%)
	**Unknown**	679	205	30·2%	(26·9%, 33·7%)	707	270	38·2%	(34·7%, 41·8%)	4,330	1,329	30·7%	(29·3%, 32·1%)	2,367	354	15.0%	(13·6%, 16·4%)

		Liver				Pancreatic				Lung				Ovarian				All cancers			
		All	EP	%EP	95% CI	All	EP	%EP	95% CI	All	EP	%EP	95% CI	All	EP	%EP	95% CI	All	EP	%EP	95% CI
	**Total**	1,541	668	43·3%	(40·9%, 45·8%)	3,491	1,674	48.0%	(46·3%, 49·6%)	16,003	5,818	36·4%	(35·6%, 37·1%)	1,908	393	20·6%	(18·8%, 22·5%)	46,003	14,199	30·9%	(30·4%, 31·3%)
**Sex**	**Male**	1,081	458	42·4%	(39·5%, 45·3%)	1,795	852	47·5%	(45·2%, 49·8%)	7,970	2,951	37·0%	(36·0%, 38·1%)					24,016	7,334	30·5%	(30·0%, 31·1%)
	**Female**	460	210	45·7%	(41·2%, 50·2%)	1,696	822	48·5%	(46·1%, 50·8%)	8,033	2,867	35·7%	(34·6%, 36·7%)	1,908	393	20·6%	(18·8%, 22·5%)	21,987	6,865	31·2%	(30·6%, 31·8%)
**Diagnosis year**	**2012**	…	…	…	…	…	…	…	…	…	…	…	…	…	…	…	…	…	…	…	…
	**2013**	…	…	…	…	…	…	…	…	…	…	…	…	…	…	…	…	…	…	…	…
	**2014**	384	178	46·4%	(41·4%, 51·4%)	859	423	49·2%	(45·9%, 52·6%)	4,011	1,563	39.0%	(37·5%, 40·5%)	446	97	21·7%	(18·2%, 25·8%)	11,354	3,750	33·0%	(32·2%, 33·9%)
	**2015**	370	163	44.1%	(39·1%, 49·1%)	835	398	47·7%	(44·3%, 51·1%)	3,916	1,456	37·2%	(35·7%, 38·7%)	488	106	21·7%	(18·3%, 25·6%)	11,423	3,535	30·9%	(30·1%, 31·8%)
	**2016**	415	192	46·3%	(41·5%, 51·1%)	835	405	48·5%	(45·1%, 51·9%)	4,023	1,454	36·1%	(34·7%, 37·6%)	485	92	19.0%	(15·7%, 22·7%)	11,466	3,549	31.0%	(30·1%, 31·8%)
	**2017**	372	135	36·3%	(31·6%, 41·3%)	962	448	46·6%	(43·4%, 49·7%)	4,053	1,345	33·2%	(31·8%, 34·7%)	489	98	20.0%	(16·7%, 23·8%)	11,760	3,365	28·6%	(27·8%, 29·4%)
**Age group**	**15-64**	483	190	39·3%	(35·1%, 43·8%)	933	404	43·3%	(40·2%, 46·5%)	4,320	1,425	33.0%	(31·6%, 34·4%)	761	106	13·9%	(11·6%, 16·6%)	13,131	3,468	26·4%	(25·7%, 27·2%)
	**65-74**	582	233	40.0%	(36·1%, 44·1%)	1,325	588	44·4%	(41·7%, 47·1%)	6,397	2,200	34·4%	(33·2%, 35·6%)	635	110	17·3%	(14·6%, 20·5%)	17,336	4,785	27·6%	(26·9%, 28·3%)
	**75-84**	376	185	49·2%	(44·2%, 54·2%)	948	484	51·1%	(47·9%, 54·2%)	4,354	1,723	39·6%	(38·1%, 41%)	402	123	30·6%	(26·3%, 35·3%)	11,995	4,252	35·4%	(34·6%, 36·3%)
	**85+**	100	60	60·0%	(50·2%, 69·1%)	285	198	69·5%	(63·9%, 74·5%)	932	470	50·4%	(47·2%, 53·6%)	110	54	49·1%	(39·9%, 58·3%)	3,541	1,694	47·8%	(46·2%, 49·5%)
**Stage**	**1**	140	32	22·9%	(16·7%, 30·5%)	112	39	34·8%	(26·6%, 44%)	2,236	464	20·8%	(19·1%, 22·5%)	256	18	7.0%	(4·5%, 10·8%)	5,977	891	14·9%	(14·0%, 15·8%)
	**2**	323	92	28·5%	(23·8%, 33·6%)	493	155	31·4%	(27·5%, 35·7%)	1,258	264	21.0%	(18·8%, 23·3%)	77	7	9·1%	(4·5%, 17·6%)	6,064	1,329	21·9%	(20·9%, 23%)
	**3**	70	32	45·7%	(34·6%, 57·3%)	244	100	41.0%	(35·0%, 47·2%)	3,309	830	25·1%	(23·6%, 26·6%)	343	39	11·4%	(8·4%, 15·2%)	7,231	1,678	23·2%	(22·2%, 24·2%)
	**4**	351	183	52·1%	(46·9%, 57·3%)	1,651	850	51·5%	(49·1%, 53·9%)	8,165	3,748	45·9%	(44·8%, 47%)	626	187	29·9%	(26·4%, 33·6%)	15,359	6,630	43·2%	(42·4%, 44%)
	**Unknown**	657	329	50·1%	(46·3%, 53·9%)	991	530	53·5%	(50·4%, 56·6%)	1,035	512	49·5%	(46·4%, 52·5%)	606	142	23·4%	(20·2%, 27%)	11,372	3,671	32·3%	(31·4%, 33·1%)

Norway		Oesophageal				Stomach				Colon				Rectal			
		All	EP	%EP	95% CI	All	EP	%EP	95% CI	All	EP	%EP	95% CI	All	EP	%EP	95% CI
	**Total**	1,332	435	32·7%	(30·2%, 35·2%)	2,357	943	40.0%	(38·0%, 42·0%)	14,941	5,354	35·8%	(35·1%, 36·6%)	5,612	888	15·8%	(14·9%, 16·8%)
**Sex**	**Male**	999	321	32·1%	(29·3%, 35·1%)	1,489	579	38·9%	(36·4%, 41·4%)	7,216	2,507	34·7%	(33·7%, 35·8%)	3,323	515	15·5%	(14·3%, 16·8%)
	**Female**	333	114	34·2%	(29·3%, 39·5%)	868	364	41·9%	(38·7%, 45·2%)	7,725	2,847	36·9%	(35·8%, 37·9%)	2,289	373	16·3%	(14·8%, 17·9%)
**Diagnosis year**	**2012**	243	85	35.0%	(29·3%, 41·2%)	473	186	39·3%	(35%, 43·8%)	2,850	1,116	39·2%	(37·4%, 41·0%)	1,056	185	17·5%	(15·3%, 19·9%)
	**2013**	254	85	33·5%	(27·9%, 39·5%)	488	193	39·5%	(35·3%, 44·0%)	2,922	1,040	35·6%	(33·9%, 37·3%)	1,157	190	16·4%	(14·4%, 18·7%)
	**2014**	277	91	32·9%	(27·6%, 38·6%)	491	215	43·8%	(39·5%, 48·2%)	2,982	1,084	36·4%	(34·6%, 38·1%)	1,144	182	15·9%	(13·9%, 18·1%)
	**2015**	284	96	33·8%	(28·5%, 39·5%)	461	192	41·6%	(37·2%, 46·2%)	3,097	1,087	35·1%	(33·4%, 36·8%)	1,129	161	14·3%	(12·3%, 16·4%)
	**2016**	274	78	28·5%	(23·5%, 34·1%)	444	157	35·4%	(31·1%, 39·9%)	3,090	1,027	33·2%	(31·6%, 34·9%)	1,126	170	15·1%	(13·1%, 17·3%)
**Age group**	**15-64**	411	111	27.0%	(22·9%, 31·5%)	704	225	32.0%	(28·6%, 35·5%)	3,721	1,116	30.0%	(28·5%, 31·5%)	1,992	222	11·1%	(9·8%, 12·6%)
	**65-74**	468	140	29·9%	(25·9%, 34·2%)	683	243	35·6%	(32·1%, 39·2%)	4,477	1,378	30·8%	(29·4%, 32·1%)	1,742	235	13·5%	(12·0%, 15·2%)
	**75-84**	313	111	35·5%	(30·4%, 40·9%)	638	279	43·7%	(39·9%, 47·6%)	4,576	1,725	37·7%	(36·3%, 39·1%)	1,337	254	19.0%	(17·0%, 21·2%)
	**85+**	140	73	52·1%	(43·9%, 60·2%)	332	196	59.0%	(53·7%, 64·2%)	2,167	1,135	52·4%	(50·3%, 54·5%)	541	177	32·7%	(28·9%, 36·8%)
**Stage**	**Local**	203	48	23·6%	(18·3%, 29·9%)	390	112	28·7%	(24·5%, 33·4%)	2,726	467	17·1%	(15·8%, 18·6%)	1,425	118	8·3%	(7·0%, 9·8%)
	**Regional**	341	89	26·1%	(21·7%, 31·0%)	615	216	35·1%	(31·5%, 39·0%)	7,810	2,648	33·9%	(32·9%, 35·0%)	2,623	364	13·9%	(12·6%, 15·3%)
	**Distant**	328	145	44·2%	(38·9%, 49·6%)	669	342	51·1%	(47·3%, 54·9%)	3,539	1,842	52·0%	(50·4%, 53·7%)	962	262	27·2%	(24·5%, 30·1%)
	**Unknown**	460	153	33·3%	(29·1%, 37·7%)	683	273	40.0%	(36·4%, 43·7%)	866	397	45·8%	(42·5%, 49·2%)	602	144	23·9%	(20·7%, 27·5%)

		Liver				Pancreatic				Lung				Ovarian				All cancers			
		All	EP	%EP	95% CI	All	EP	%EP	95% CI	All	EP	%EP	95% CI	All	EP	%EP	95% CI	All	EP	%EP	95% CI
	**Total**	1,181	597	50·6%	(47·7%, 53·4%)	3,715	2,059	55·4%	(53·8%, 57·0%)	14,402	5,679	39·4%	(38·6%, 40·2%)	2,607	911	34·9%	(33·1%, 36·8%)	46,147	16,866	36·5%	(36·1%, 37·0%)
**Sex**	**Male**	763	397	52.0%	(48·5%, 55·6%)	1,851	1,041	56·2%	(54·0%, 58·5%)	7,678	3,050	39·7%	(38·6%, 40·8%)					23,319	8,410	36·1%	(35·5%, 36·7%)
	**Female**	418	200	47·8%	(43·1%, 52·6%)	1,864	1,018	54·6%	(52·3%, 56·9%)	6,724	2,629	39·1%	(37·9%, 40·3%)	2,607	911	34·9%	(33·1%, 36·8%)	22,828	8,456	37·0%	(36·4%, 37·7%)
**Diagnosis year**	**2012**	209	114	54·5%	(47·8%, 61·2%)	770	432	56·1%	(52·6%, 59·6%)	2,855	1,205	42·2%	(40·4%, 44·0%)	506	185	36·6%	(32·5%, 40·8%)	8,962	3,508	39·1%	(38·1%, 40·2%)
	**2013**	246	127	51·6%	(45·4%, 57·8%)	726	395	54·4%	(50·8%, 58·0%)	2,773	1,139	41·1%	(39·3%, 42·9%)	494	181	36·6%	(32·5%, 41·0%)	9,060	3,350	37.0%	(36·0%, 38·0%)
	**2014**	221	122	55·2%	(48·6%, 61·6%)	742	416	56·1%	(52·5%, 59·6%)	2,906	1,126	38·7%	(37·0%, 40·5%)	530	199	37·5%	(33·5%, 41·7%)	9,293	3,435	37.0%	(36·0%, 37·9%)
	**2015**	265	122	46.0%	(40·1%, 52·1%)	810	448	55·3%	(51·9%, 58·7%)	2,967	1,165	39·3%	(37·5%, 41·0%)	548	174	31·8%	(28·0%, 35·8%)	9,561	3,445	36.0%	(35·1%, 37·0%)
	**2016**	240	112	46·7%	(40·5%, 53·0%)	667	368	55·2%	(51·4%, 58·9%)	2,901	1,044	36.0%	(34·3%, 37·8%)	529	172	32·5%	(28·7%, 36·6%)	9,271	3,128	33·7%	(32·8%, 34·7%)
**Age group**	**15-64**	445	229	51·5%	(46·8%, 56·1%)	976	477	48·9%	(45·7%, 52·0%)	3,807	1,435	37·7%	(36·2%, 39·2%)	1,221	350	28·7%	(26·2%, 31·3%)	13,277	4,165	31·4%	(30·6%, 32·2%)
	**65-74**	321	151	47.0%	(41·6%, 52·5%)	1,237	678	54·8%	(52·0%, 57·6%)	5,619	2,088	37·2%	(35·9%, 38·4%)	731	259	35·4%	(32·0%, 39·0%)	15,278	5,172	33·9%	(33·1%, 34·6%)
	**75-84**	305	159	52·1%	(46·5%, 57·7%)	983	556	56·6%	(53·4%, 59·6%)	3,830	1,561	40·8%	(39·2%, 42·3%)	442	193	43·7%	(39·1%, 48·3%)	12,424	4,838	38·9%	(38·1%, 39·8%)
	**85+**	110	58	52·7%	(43·5%, 61·8%)	519	348	67·1%	(62·9%, 71·0%)	1,146	595	51·9%	(49·0%, 54·8%)	213	109	51·2%	(44·5%, 57·8%)	5,168	2,691	52·1%	(50·7%, 53·4%)
**Stage**	**Local**	359	146	40·7%	(35·7%, 45·8%)	311	104	33·4%	(28·4%, 38·9%)	2,572	420	16·3%	(15·0%, 17·8%)	529	72	13·6%	(10·9%, 16·8%)	8,515	1,487	17·5%	(16·7%, 18·3%)
	**Regional**	141	67	47·5%	(39·5%, 55·7%)	810	364	44·9%	(41·5%, 48·4%)	4,038	1,167	28·9%	(27·5%, 30·3%)	82	27	32·9%	(23·7%, 43·7%)	16,460	4,942	30·0%	(29·3%, 30·7%)
	**Distant**	264	164	62·1%	(56·1%, 67·8%)	1,842	1,190	64·6%	(62·4%, 66·8%)	6,173	3,446	55·8%	(54·6%, 57·1%)	1,836	749	40·8%	(38·6%, 43·1%)	15,613	8,140	52·1%	(51·4%, 52·9%)
	**Unknown**	417	220	52·8%	(48·0%, 57·5%)	752	401	53·3%	(49·8%, 56·9%)	1,619	646	39·9%	(37·5%, 42·3%)	160	63	39·4%	(32·1%, 47·1%)	5,559	2,297	41·3%	(40·0%, 42·6%)

England		Oesophageal				Stomach				Colon				Rectal			
		All	EP	%EP	95% CI	All	EP	%EP	95% CI	All	EP	%EP	95% CI	All	EP	%EP	95% CI
	**Total**	27,274	5,364	19·7%	(19·2%, 20·1%)	19,813	6,224	31·4%	(30·8%, 32·1%)	88,219	25,859	29·3%	(29·0%, 29·6%)	33,905	3,628	10·7%	(10·4%, 11·0%)
**Sex**	**Male**	18,551	3,369	18·2%	(17·6%, 18·7%)	12,877	3,826	29·7%	(28·9%, 30·5%)	45,853	12,463	27·2%	(26·8%, 27·6%)	21,251	2,057	9·7%	(9·3%, 10·1%)
	**Female**	8,723	1,995	22·9%	(22·0%, 23·8%)	6,936	2,398	34·6%	(33·5%, 35·7%)	42,366	13,396	31·6%	(31·2%, 32·1%)	12,654	1,571	12·4%	(11·9%, 13·0%)
**Diagnosis**	**2012**	…	…	…	…	…	…	…	…	…	…	…	…	…	…	…	…
**year**	**2013**	6,668	1,301	19·5%	(18·6%, 20·5%)	5,080	1,694	33·3%	(32·1%, 34·7%)	21,531	6,506	30·2%	(29·6%, 30·8%)	8,363	907	10·8%	(10·2%, 11·5%)
	**2014**	6,676	1,327	19·9%	(18·9%, 20·9%)	4,876	1,524	31·3%	(30·0%, 32·6%)	21,819	6,435	29·5%	(28·9%, 30·1%)	8,415	884	10·5%	(9·9%, 11·2%)
	**2015**	6,973	1,353	19·4%	(18·5%, 20·3%)	5,013	1,551	30·9%	(29·7%, 32·2%)	22,472	6,524	29·0%	(28·4%, 29·6%)	8,524	956	11·2%	(10·6%, 11·9%)
	**2016**	6,957	1,383	19·9%	(19·0%, 20·8%)	4,844	1,455	30·0%	(28·8%, 31·3%)	22,397	6,394	28·5%	(28·0%, 29·1%)	8,603	881	10·2%	(9·6%, 10·9%)
**Age group**	**15-64**	7,567	1,075	14·2%	(13·4%, 15·0%)	4,712	1,213	25·7%	(24·5%, 27·0%)	23,531	6,678	28·4%	(27·8%, 29·0%)	11,769	819	7·0%	(6·5%, 7·4%)
	**65-74**	8,663	1,312	15·1%	(14·4%, 15·9%)	5,070	1,203	23·7%	(22·6%, 24·9%)	24,057	5,146	21·4%	(20·9%, 21·9%)	10,103	793	7·8%	(7·3%, 8·4%)
	**75-84**	7,369	1,588	21·5%	(20·6%, 22·5%)	6,689	2,155	32·2%	(31·1%, 33·3%)	26,651	7,706	28·9%	(28·4%, 29·5%)	8,428	1,066	12·6%	(12%, 13·4%)
	**85+**	3,675	1,389	37·8%	(36·2%, 39·4%)	3,342	1,653	49·5%	(47·8%, 51·2%)	13,980	6,329	45·3%	(44·4%, 46·1%)	3,605	950	26·4%	(24·9%, 27·8%)
**Stage**	**1**	2,550	237	9·3%	(8·2%, 10·5%)	1,763	281	15·9%	(14·3%, 17·7%)	11,397	1,093	9·6%	(9·1%, 10·1%)	7,396	273	3·7%	(3·3%, 4·1%)
	**2**	3,396	429	12·6%	(11·6%, 13·8%)	2,666	612	23·0%	(21·4%, 24·6%)	22,408	5,289	23·6%	(23·1%, 24·2%)	5,753	494	8·6%	(7·9%, 9·3%)
	**3**	8,038	1,048	13·0%	(12·3%, 13·8%)	3,257	665	20·4%	(19·1%, 21·8%)	21,088	5,424	25·7%	(25·1%, 26·3%)	11,091	756	6·8%	(6·4%, 7·3%)
	**4**	8,111	2,044	25·2%	(24·3%, 26·2%)	7,236	2,792	38·6%	(37·5%, 39·7%)	22,492	9,314	41·4%	(40·8%, 42·1%)	6,241	1,313	21·0%	(20%, 22·1%)
	**Unknown**	5,179	1,606	31·0%	(29·8%, 32·3%)	4,891	1,874	38·3%	(37·0%, 39·7%)	10,834	4,739	43·7%	(42·8%, 44·7%)	3,424	792	23·1%	(21·7%, 24·6%)

		Liver				Pancreatic				Lung				Ovarian				All cancers			
		All	EP	%EP	95% CI	All	EP	%EP	95% CI	All	EP	%EP	95% CI	All	EP	%EP	95% CI	All	EP	%EP	95% CI
	**Total**	17,699	7,497	42·4%	(41·6%, 43·1%)	30,442	14,291	46·9%	(46·4%, 47·5%)	135,396	46,951	34·7%	(34·4%, 34·9%)	20,446	7,120	34·8%	(34·2%, 35·5%)	373,194	116,934	31·3%	(31·2%, 31·5%)
**Sex**	**Male**	11,409	4,482	39·3%	(38·4%, 40·2%)	15,243	6,988	45·8%	(45·1%, 46·6%)	71,819	24,654	34·3%	(34·0%, 34·7%)					197,003	57,839	29·4%	(29·2%, 29·6%)
	**Female**	6,290	3,015	47·9%	(46·7%, 49·2%)	15,199	7,303	48·0%	(47·3%, 48·8%)	63,577	22,297	35·1%	(34·7%, 35·4%)	20,446	7,120	34·8%	(34·2%, 35·5%)	176,191	59,095	33·5%	(33·3%, 33·8%)
**Diagnosis**	**2012**	…	…	…	…	…	…	…	…	…	…	…	…	…	…	…	…	…	…	…	…
**year**	**2013**	4,155	1,830	44·0%	(42·5%, 45·6%)	7,235	3,426	47·4%	(46·2%, 48·5%)	32,950	11,842	35·9%	(35·4%, 36·5%)	5,011	1,765	35·2%	(33·9%, 36·6%)	90,993	29,271	32·2%	(31·9%, 32·5%)
	**2014**	4,377	1,865	42·6%	(41·2%, 44·1%)	7,504	3,543	47·2%	(46·1%, 48·3%)	33,800	11,948	35·3%	(34·8%, 35·9%)	5,142	1,789	34·8%	(33·5%, 36·1%)	92,609	29,315	31·7%	(31·4%, 32·0%)
	**2015**	4,500	1,894	42·1%	(40·7%, 43·5%)	7,789	3,664	47·0%	(45·9%, 48·2%)	33,968	11,586	34·1%	(33·6%, 34·6%)	5,057	1,769	35·0%	(33·7%, 36·3%)	94,296	29,297	31·1%	(30·8%, 31·4%)
	**2016**	4,667	1,908	40·9%	(39·5%, 42·3%)	7,914	3,658	46·2%	(45·1%, 47·3%)	34,678	11,575	33·4%	(32·9%, 33·9%)	5,236	1,797	34·3%	(33·0%, 35·6%)	95,296	29,051	30·5%	(30·2%, 30·8%)
**Age group**	**15-64**	4,909	1,646	33·5%	(32·2%, 34·9%)	7,282	2,709	37·2%	(36·1%, 38·3%)	30,543	8,521	27·9%	(27·4%, 28·4%)	8,106	2,133	26·3%	(25·4%, 27·3%)	98,419	24,794	25·2%	(24·9%, 25·5%)
	**65-74**	5,138	1,911	37·2%	(35·9%, 38·5%)	8,964	3,627	40·5%	(39·5%, 41·5%)	45,107	13,237	29·3%	(28·9%, 29·8%)	5,868	1,963	33·5%	(32·3%, 34·7%)	112,970	29,192	25·8%	(25·6%, 26·1%)
	**75-84**	5,234	2,415	46·1%	(44·8%, 47·5%)	9,429	4,815	51·1%	(50·1%, 52·1%)	42,119	15,838	37·6%	(37·1%, 38·1%)	4,602	1,969	42·8%	(41·4%, 44·2%)	110,521	37,552	34·0%	(33·7%, 34·3%)
	**85+**	2,418	1,525	63·1%	(61·1%, 65·0%)	4,767	3,140	65·9%	(64·5%, 67·2%)	17,627	9,355	53·1%	(52·3%, 53·8%)	1,870	1,055	56·4%	(54·2%, 58·6%)	51,284	25,396	49·5%	(49·1%, 50·0%)
**Stage**	**1**	911	196	21·5%	(19·0%, 24·3%)	1,464	620	42·3%	(39·8%, 44·9%)	19,368	2,941	15·2%	(14·7%, 15·7%)	3,596	384	10·7%	(9·7%, 11·7%)	48,445	6,025	12·4%	(12·1%, 12·7%)
	**2**	1,092	235	21·5%	(19·2%, 24·1%)	3,168	1,085	34·2%	(32·6%, 35·9%)	9,742	1,647	16·9%	(16·2%, 17·7%)	1,150	137	11·9%	(10·2%, 13·9%)	49,375	9,928	20·1%	(19·8%, 20·5%)
	**3**	1,014	358	35·3%	(32·4%, 38·3%)	2,382	816	34·3%	(32·4%, 36·2%)	26,334	5,719	21·7%	(21·2%, 22·2%)	7,649	2,646	34·6%	(33·5%, 35·7%)	80,853	17,432	21·6%	(21·3%, 21·8%)
	**4**	4,484	2,198	49·0%	(47·6%, 50·5%)	15,059	7,590	50·4%	(49·6%, 51·2%)	68,141	30,926	45·4%	(45·0%, 45·8%)	4,618	2,348	50·8%	(49·4%, 52·3%)	136,382	58,525	42·9%	(42·7%, 43·2%)
	**Unknown**	10,198	4,510	44·2%	(43·3%, 45·2%)	8,369	4,180	49·9%	(48·9%, 51·0%)	11,811	5,718	48·4%	(47·5%, 49·3%)	3,433	1,605	46·8%	(45·1%, 48·4%)	58,139	25,024	43·0%	(42·6%, 43·4%)

Northern Ireland		Oesophageal				Stomach				Colon				Rectal			
		All	EP	%EP	95% CI	All	EP	%EP	95% CI	All	EP	%EP	95% CI	All	EP	%EP	95% CI
	**Total**	972	193	19·9%	(17·5%, 22·5%)	990	293	29·6%	(26·8%, 32·5%)	3,846	914	23·8%	(22·4%, 25·1%)	1,376	125	9·1%	(7·7%, 10·7%)
**Sex**	**Male**	660	116	17·6%	(14·9%, 20·7%)	620	170	27·4%	(24·1%, 31·1%)	2,026	427	21·1%	(19·4%, 22·9%)	851	62	7·3%	(5·7%, 9·2%)
	**Female**	312	77	24·7%	(20·2%, 29·8%)	370	123	33·2%	(28·6%, 38·2%)	1,820	487	26·8%	(24·8%, 28·8%)	525	63	12·0%	(9·5%, 15·1%)
**Diagnosis year**	**2012**	196	43	21·9%	(16·7%, 28·2%)	180	65	36·1%	(29·5%, 43·4%)	782	193	24·7%	(21·8%, 27·8%)	315	25	7·9%	(5·4%, 11·5%)
	**2013**	184	38	20·7%	(15·4%, 27·1%)	212	57	26·9%	(21·4%, 33·2%)	823	198	24·1%	(21·3%, 27·1%)	278	28	10·1%	(7·1%, 14·2%)
	**2014**	199	40	20·1%	(15·1%, 26·2%)	205	57	27·8%	(22·1%, 34·3%)	766	169	22·1%	(19·3%, 25·1%)	251	26	10·4%	(7·2%, 14·7%)
	**2015**	186	38	20·4%	(15·3%, 26·8%)	185	55	29·7%	(23·6%, 36·7%)	724	162	22·4%	(19·5%, 25·6%)	269	19	7·1%	(4·6%, 10·8%)
	**2016**	207	34	16·4%	(12·0%, 22·1%)	208	59	28·4%	(22·7%, 34·8%)	751	192	25·6%	(22·6%, 28·8%)	263	27	10·3%	(7·2%, 14·5%)
**Age group**	**15-64**	335	48	14·3%	(11·0%, 18·5%)	263	77	29·3%	(24·1%, 35·0%)	1,085	260	24·0%	(21·5%, 26·6%)	522	29	5·6%	(3·9%, 7·9%)
	**65-74**	303	49	16·2%	(12·5%, 20·7%)	267	61	22·9%	(18·2%, 28·2%)	1,156	209	18·1%	(16·0%, 20·4%)	420	24	5·7%	(3·9%, 8·4%)
	**75-84**	221	55	24·9%	(19·6%, 31·0%)	317	104	32·8%	(27·9%, 38·2%)	1,122	259	23·1%	(20·7%, 25·6%)	319	37	11·6%	(8·5%, 15·6%)
	**85+**	113	41	36·3%	(28·0%, 45·5%)	143	51	35·7%	(28·3%, 43·8%)	483	186	38·5%	(34·3%, 42·9%)	115	35	30·4%	(22·8%, 39·4%)
**Stage**	**1**	81	3	3·7%	(1·3%, 10·3%)	96	4	4·2%	(1·6%, 10·2%)	601	27	4·5%	(3·1%, 6·5%)	297	13	4·4%	(2·6%, 7·3%)
	**2**	121	12	9·9%	(5·8%, 16·5%)	74	14	18·9%	(11·6%, 29·3%)	1,083	189	17·5%	(15·3%, 19·8%)	268	7	2·6%	(1·3%, 5·3%)
	**3**	183	28	15·3%	(10·8%, 21·2%)	117	22	18·8%	(12·8%, 26·8%)	896	199	22·2%	(19·6%, 25·0%)	508	35	6·9%	(5·0%, 9·4%)
	**4**	298	72	24·2%	(19·6%, 29·3%)	385	149	38·7%	(34·0%, 43·7%)	874	352	40·3%	(37·1%, 43·6%)	217	45	20·7%	(15·9%, 26·6%)
	**Unknown**	289	78	27.0%	(22·2%, 32·4%)	318	104	32·7%	(27·8%, 38·0%)	392	147	37·5%	(32·9%, 42·4%)	86	25	29·1%	(20·5%, 39·4%)

		Liver				Pancreatic				Lung				Ovarian				All cancers			
		All	EP	%EP	95% CI	All	EP	%EP	95% CI	All	EP	%EP	95% CI	All	EP	%EP	95% CI	All	EP	%EP	95% CI
	**Total**	582	215	36·9%	(33·1%, 40·9%)	1,162	496	42·7%	(39·9%, 45·5%)	5,752	1,843	32·0%	(30·8%, 33·3%)	821	243	29·6%	(26·6%, 32·8%)	15,501	4,322	27·9%	(27·2%, 28·6%)
**Sex**	**Male**	384	142	37·0%	(32·3%, 41·9%)	572	242	42·3%	(38·3%, 46·4%)	3,021	965	31·9%	(30·3%, 33·6%)					8,134	2,124	26·1%	(25·2%, 27·1%)
	**Female**	198	73	36·9%	(30·5%, 43·8%)	590	254	43·1%	(39·1%, 47·1%)	2,731	878	32·1%	(30·4%, 33·9%)	821	243	29·6%	(26·6%, 32·8%)	7,367	2,198	29·8%	(28·8%, 30·9%)
**Diagnosis year**	**2012**	98	43	43·9%	(34·5%, 53·7%)	230	109	47·4%	(41·0%, 53·8%)	1,099	374	34.0%	(31·3%, 36·9%)	171	57	33·3%	(26·7%, 40·7%)	3,071	909	29·6%	(28·0%, 31·2%)
	**2013**	116	52	44·8%	(36·1%, 53·9%)	222	93	41·9%	(35·6%, 48·5%)	1,121	354	31·6%	(28·9%, 34·4%)	169	47	27·8%	(21·6%, 35·0%)	3,125	867	27·7%	(26·2%, 29·3%)
	**2014**	120	40	33·3%	(25·5%, 42·2%)	222	99	44·6%	(38·2%, 51·2%)	1,124	364	32·4%	(29·7%, 35·2%)	164	61	37·2%	(30·2%, 44·8%)	3,051	856	28·1%	(26·5%, 29·7%)
	**2015**	132	47	35·6%	(28·0%, 44·1%)	258	108	41·9%	(36·0%, 48·0%)	1,158	363	31·3%	(28·7%, 34·1%)	160	43	26·9%	(20·6%, 34·2%)	3,072	835	27·2%	(25·6%, 28·8%)
	**2016**	116	33	28·4%	(21·0%, 37·2%)	230	87	37·8%	(31·8%, 44·2%)	1,250	388	31·0%	(28·5%, 33·7%)	157	35	22·3%	(16·5%, 29·4%)	3,182	855	26·9%	(25·4%, 28·4%)
**Age group**	**15-64**	149	51	34·2%	(27·1%, 42·2%)	278	98	35·3%	(29·9%, 41·0%)	1,466	373	25·4%	(23·3%, 27·7%)	334	67	20·1%	(16·1%, 24·7%)	4,432	1,003	22·6%	(21·4%, 23·9%)
	**65-74**	181	47	26·0%	(20·1%, 32·8%)	389	145	37·3%	(32·6%, 42·2%)	2,022	611	30·2%	(28·3%, 32·3%)	227	74	32·6%	(26·8%, 38·9%)	4,965	1,220	24·6%	(23·4%, 25·8%)
	**75-84**	184	78	42·4%	(35·5%, 49·6%)	339	150	44·2%	(39·1%, 49·6%)	1,724	603	35·0%	(32·8%, 37·3%)	182	65	35·7%	(29·1%, 42·9%)	4,408	1,351	30·6%	(29·3%, 32·0%)
	**85+**	68	39	57·4%	(45·5%, 68·4%)	156	103	66·0%	(58·3%, 73·0%)	540	256	47·4%	(43·2%, 51·6%)	78	37	47·4%	(36·7%, 58·4%)	1,696	748	44·1%	(41·8%, 46·5%)
**Stage**	**1**	69	6	8·7%	(4·0%, 17·7%)	53	12	22·6%	(13·5%, 35·5%)	860	87	10·1%	(8·3%, 12·3%)	164	20	12·2%	(8·0%, 18·1%)	2,221	172	7·7%	(6·7%, 8·9%)
	**2**	59	8	13·6%	(7·0%, 24·5%)	131	18	13·7%	(8·9%, 20·7%)	437	44	10·1%	(7·6%, 13·2%)	61	3	4·9%	(1·7%, 13·5%)	2,234	295	13·2%	(11·9%, 14·7%)
	**3**	58	21	36·2%	(25·1%, 49·1%)	133	34	25·6%	(18·9%, 33·6%)	1,320	281	21·3%	(19·2%, 23·6%)	369	114	30·9%	(26·4%, 35·8%)	3,584	734	20·5%	(19·2%, 21·8%)
	**4**	128	56	43·8%	(35·5%, 52·4%)	562	273	48·6%	(44·5%, 52·7%)	2,594	1,171	45·1%	(43·2%, 47·1%)	126	57	45·2%	(36·8%, 53·9%)	5,184	2,175	42·0%	(40·6%, 43·3%)
	**Unknown**	268	124	46·3%	(40·4%, 52·2%)	283	159	56·2%	(50·4%, 61·8%)	541	260	48·1%	(43·9%, 52·3%)	101	49	48·5%	(39·0%, 58·1%)	2,278	946	41·5%	(39·5%, 43·6%)

Scotland		Oesophageal				Stomach				Colon				Rectal			
		**All**	**EP**	**%EP**	**95% CI**	**All**	**EP**	**%EP**	**95% CI**	**All**	**EP**	**%EP**	**95% CI**	**All**	**EP**	**%EP**	**95% CI**
	**Total**	3,378	851	25·2%	(23·8%, 26·7%)	2,337	913	39·1%	(37·1%, 41·1%)	9,600	3366	35·1%	(34·1%, 36·0%)	3,535	499	14·1%	(13·0%, 15·3%)
**Sex**	**Male**	2,231	545	24·4%	(22·7%, 26·3%)	1,463	557	38·1%	(35·6%, 40·6%)	4,890	1655	33·8%	(32·5%, 35·2%)	2,196	290	13·2%	(11·9%, 14·7%)
	**Female**	1,147	306	26·7%	(24·2%, 29·3%)	874	356	40·7%	(37·5%, 44·0%)	4,710	1711	36·3%	(35·0%, 37·7%)	1,339	209	15·6%	(13·8%, 17·7%)
**Diagnosis year**	**2014**	854	222	26·0%	(23·2%, 29.0%)	622	248	39·9%	(36·1%, 43·8%)	2,444	868	35·5%	(33·6%, 37·4%)	876	126	14·4%	(12·2%, 16·9%)
	**2015**	856	214	25·0%	(22·2%, 28.0%)	564	219	38·8%	(34·9%, 42·9%)	2,409	853	35·4%	(33·5%, 37·3%)	838	121	14·4%	(12·2%, 17·0%)
	**2016**	787	193	24·5%	(21·6%, 27·6%)	576	223	38·7%	(34·8%, 42·8%)	2,365	813	34·4%	(32·5%, 36·3%)	914	127	13·9%	(11·8%, 16·3%)
	**2017**	881	222	25·2%	(22·4%, 28·2%)	575	223	38·8%	(34·9%, 42·8%)	2,382	832	34·9%	(33·0%, 36·9%)	907	125	13·8%	(11·7%, 16·2%)
**Age group**	**15-64**	935	186	19·9%	(17·5%, 22·6%)	626	205	32·7%	(29·2%, 36·5%)	2,568	822	32·0%	(30·2%, 33·8%)	1,311	133	10·1%	(8·6%, 11·9%)
	**65-74**	1,087	229	21·1%	(18·7%, 23·6%)	560	164	29·3%	(25·7%, 33·2%)	2,782	740	26·6%	(25·0%, 28·3%)	1,072	123	11·5%	(9·7%, 13·5%)
	**75-84**	942	254	27·0%	(24·2%, 29·9%)	814	338	41·5%	(38·2%, 44·9%)	2,904	1068	36·8%	(35·0%, 38·5%)	844	152	18·0%	(15·6%, 20·7%)
	**85+**	414	182	44·0%	(39·3%, 48·8%)	337	206	61·1%	(55·8%, 66·2%)	1,346	736	54·7%	(52·0%, 57·3%)	308	91	29·5%	(24·7%, 34·9%)
**Stage**	**1**	270	29	10·7%	(7·6%, 15·0%)	149	37	24·8%	(18·6%, 32·3%)	1,017	106	10·4%	(8·7%, 12·5%)	766	39	5·1%	(3·7%, 6·9%)
	**2**	317	54	17·0%	(13·3%, 21·6%)	283	98	34·6%	(29·3%, 40·3%)	2,532	703	27·8%	(26·1%, 29·5%)	742	72	9·7%	(7·8%, 12·0%)
	**3**	909	172	18·9%	(16·5%, 21·6%)	227	41	18·1%	(13·6%, 23·6%)	2,270	698	30·7%	(28·9%, 32·7%)	876	84	9·6%	(7·8%, 11·7%)
	**4**	1,112	342	30·8%	(28·1%, 33·5%)	990	429	43·3%	(40·3%, 46·4%)	2,345	1142	48·7%	(46·7%, 50·7%)	628	164	26·1%	(22·8%, 29·7%)
	**Unknown**	770	254	33·0%	(29·8%, 36·4%)	688	308	44·8%	(41·1%, 48·5%)	1,436	717	49·9%	(47·3%, 52·5%)	523	140	26·8%	(23·2%, 30·7%)

		Liver				Pancreatic				Lung				Ovarian				All cancers			
		**All**	**EP**	**%EP**	**95% CI**	**All**	**EP**	**%EP**	**95% CI**	**All**	**EP**	**%EP**	**95% CI**	**All**	**EP**	**%EP**	**95% CI**	**All**	**EP**	**%EP**	**95% CI**
	**Total**	2,340	1,111	47·5%	(45·5%, 49·5%)	3,054	1,809	59·2%	(57·5%, 61·0%)	18,662	7,863	42·1%	(41·4%, 42·8%)	2123	909	42·8%	(40·7%, 44·9%)	45,029	17,321	38·5%	(38%, 38·9%)
**Sex**	**Male**	1,573	691	43·9%	(41·5%, 46·4%)	1,500	891	59·4%	(56·9%, 61·9%)	9,189	3,877	42·2%	(41·2%, 43·2%)					23,042	8,506	36·9%	(36·3%, 37·5%)
	**Female**	767	420	54·8%	(51·2%, 58·2%)	1,554	918	59·1%	(56·6%, 61·5%)	9,473	3,986	42·1%	(41·1%, 43·1%)	2123	909	42·8%	(40·7%, 44·9%)	21,987	8,815	40·1%	(39·4%, 40·7%)
**Diagnosis year**	**2014**	574	265	46·2%	(42·1%, 50·3%)	766	475	62·0%	(58·5%, 65·4%)	4,901	2,076	42·4%	(41·0%, 43·7%)	562	231	41·1%	(37·1%, 45·2%)	11,599	4,511	38·9%	(38·0%, 39·8%)
	**2015**	599	286	47·7%	(43·8%, 51·7%)	768	468	60·9%	(57·4%, 64·3%)	4,531	1,936	42·7%	(41·3%, 44·2%)	526	249	47·3%	(43·1%, 51·6%)	11,091	4,346	39·2%	(38·3%, 40·1%)
	**2016**	597	295	49·4%	(45·4%, 53·4%)	766	430	56·1%	(52·6%, 59·6%)	4,517	1,922	42·6%	(41·1%, 44·0%)	542	233	43·0%	(38·9%, 47·2%)	11,064	4,236	38·3%	(37·4%, 39·2%)
	**2017**	570	265	46·5%	(42·4%, 50·6%)	754	436	57·8%	(54·3%, 61·3%)	4,713	1,929	40·9%	(39·5%, 42·3%)	493	196	39·8%	(35·5%, 44·1%)	11,275	4,228	37·5%	(36·6%, 38·4%)
**Age group**	**15-64**	679	266	39·2%	(35·6%, 42·9%)	756	423	56·0%	(52·4%, 59·5%)	4,319	1,573	36·4%	(35·0%, 37·9%)	830	312	37·6%	(34·4%, 40·9%)	12,024	3,920	32·6%	(31·8%, 33·4%)
	**65-74**	736	324	44·0%	(40·5%, 47·6%)	977	532	54·5%	(51·3%, 57·6%)	6,461	2,486	38·5%	(37·3%, 39·7%)	558	219	39·2%	(35·3%, 43·4%)	14,233	4,817	33·8%	(33·1%, 34·6%)
	**75-84**	655	328	50·1%	(46·3%, 53·9%)	912	550	60·3%	(57·1%, 63·4%)	5,818	2,640	45·4%	(44·1%, 46·7%)	510	250	49·0%	(44·7%, 53·3%)	13,399	5,580	41·6%	(40·8%, 42·5%)
	**85+**	270	193	71·5%	(65·8%, 76·5%)	409	304	74·3%	(69·9%, 78·3%)	2,064	1,164	56·4%	(54·2%, 58·5%)	225	128	56·9%	(50·4%, 63·2%)	5,373	3,004	55·9%	(54·6%, 57·2%)
**Stage**	**1**	232	53	22·8%	(17·9%, 28·7%)	207	119	57·5%	(50·7%, 64·0%)	2,781	706	25·4%	(23·8%, 27·0%)	436	127	29·1%	(25·1%, 33·6%)	5,858	1,216	20·8%	(19·7%, 21·8%)
	**2**	290	84	29·0%	(24·0%, 34·4%)	300	172	57·3%	(51·7%, 62·8%)	1,249	312	25·0%	(22·7%, 27·5%)	144	39	27·1%	(20·5%, 34·9%)	5,857	1,534	26·2%	(25·1%, 27·3%)
	**3**	133	58	43·6%	(35·5%, 52·1%)	278	137	49·3%	(43·5%, 55·1%)	3,809	1,276	33·5%	(32·0%, 35·0%)	678	270	39·8%	(36·2%, 43·6%)	9,180	2,736	29·8%	(28·9%, 30·7%)
	**4**	669	403	60·2%	(56·5%, 63·9%)	1,676	1,000	59·7%	(57·3%, 62·0%)	9,245	4,792	51·8%	(50·8%, 52·9%)	585	313	53·5%	(49·5%, 57·5%)	17,250	8,585	49·8%	(49·0%, 50·5%)
	**Unknown**	1,016	513	50·5%	(47·4%, 53·6%)	593	381	64·2%	(60·3%, 68·0%)	1,578	777	49·2%	(46·8%, 51·7%)	280	160	57·1%	(51·3%, 62·8%)	6,884	3,250	47·2%	(46·0%, 48·4%)

Wales		Oesophageal				Stomach				Colon				Rectal			
		All	EP	%EP	95% CI	All	EP	%EP	95% CI	All	EP	%EP	95% CI	All	EP	%EP	95% CI
	**Total**	1,703	435	25·5%	(23·5%, 27·7%)	1,656	648	39·1%	(36·8%, 41·5%)	6,142	2,102	34·2%	(33·0%, 35·4%)	2,577	355	13·8%	(12·5%, 15·2%)
**Sex**	**Male**	1,128	273	24·2%	(21·8%, 26·8%)	1,131	423	37·4%	(34·6%, 40·3%)	3,349	1,103	32·9%	(31·4%, 34·5%)	1,658	221	13·3%	(11·8%, 15·1%)
	**Female**	575	162	28·2%	(24·7%, 32·0%)	525	225	42·9%	(38·7%, 47·1%)	2,793	999	35·8%	(34·0%, 37·6%)	919	134	14·6%	(12·4%, 17·0%)
**Diagnosis**	**2012**	…	…	…	…	…	…	…	…	…	…	…	…	…	…	…	…
**year**	**2013**	395	109	27·6%	(23·4%, 32·2%)	436	176	40·4%	(35·9%, 45·0%)	1,501	535	35·6%	(33·3%, 38·1%)	650	72	11·1%	(8·9%, 13·7%)
	**2014**	438	109	24·9%	(21·1%, 29·1%)	423	171	40·4%	(35·9%, 45·2%)	1,598	539	33·7%	(31·5%, 36·1%)	665	95	14·3%	(11·8%, 17·2%)
	**2015**	459	116	25·3%	(21·5%, 29·4%)	399	155	38·8%	(34·2%, 43·7%)	1,536	515	33·5%	(31·2%, 35·9%)	613	95	15·5%	(12·8%, 18·6%)
	**2016**	411	101	24·6%	(20·7%, 29·0%)	398	146	36·7%	(32·1%, 41·5%)	1,507	513	34·0%	(31·7%, 36·5%)	649	93	14·3%	(11·8%, 17·2%)
**Age group**	**15-64**	466	87	18·7%	(15·4%, 22·5%)	350	98	28·0%	(23·6%, 32·9%)	1,475	457	31·0%	(28·7%, 33·4%)	811	74	9·1%	(7·3%, 11·3%)
	**65-74**	554	128	23·1%	(19·8%, 26·8%)	477	157	32·9%	(28·8%, 37·3%)	1,829	517	28·3%	(26·3%, 30·4%)	824	94	11·4%	(9·4%, 13·8%)
	**75-84**	481	117	24·3%	(20·7%, 28·4%)	546	233	42·7%	(38·6%, 46·9%)	1,940	693	35·7%	(33·6%, 37·9%)	682	114	16·7%	(14·1%, 19·7%)
	**85+**	202	103	51·0%	(44·1%, 57·8%)	283	160	56·5%	(50·7%, 62·2%)	898	435	48·4%	(45·2%, 51·7%)	260	73	28·1%	(23·0%, 33·8%)
**Stage**	**1**	100	15	15·0%	(9·3%, 23·3%)	141	37	26·2%	(19·7%, 34·1%)	774	74	9·6%	(7·7%, 11·8%)	462	23	5·0%	(3·3%, 7·4%)
	**2**	319	64	20·1%	(16·0%, 24·8%)	198	56	28·3%	(22·5%, 34·9%)	1,517	480	31·6%	(29·3%, 34%)	415	57	13·7%	(10·8%, 17·4%)
	**3**	491	85	17·3%	(14·2%, 20·9%)	287	91	31·7%	(26·6%, 37·3%)	1,597	503	31·5%	(29·3%, 33·8%)	991	99	10·0%	(8·3%, 12·0%)
	**4**	495	164	33·1%	(29·1%, 37·4%)	699	317	45·4%	(41·7%, 49·1%)	1,500	708	47·2%	(44·7%, 49·7%)	463	118	25·5%	(21·7%, 29·6%)
	**Unknown**	298	107	35·9%	(30·7%, 41·5%)	331	147	44·4%	(39·2%, 49·8%)	754	337	44·7%	(41·2%, 48·3%)	246	58	23·6%	(18·7%, 29·3%)

		Liver				Pancreatic				Lung				Ovarian				All cancers			
		All	EP	%EP	95% CI	All	EP	%EP	95% CI	All	EP	%EP	95% CI	All	EP	%EP	95% CI	All	EP	%EP	95% CI
	**Total**	1,155	583	50·5%	(47·6%, 53·4%)	1,965	1,115	56·7%	(54·5%, 58·9%)	9,365	3,882	41·5%	(40·5%, 42·5%)	1,576	643	40·8%	(38·4%, 43·2%)	26,139	9,763	37·4%	(36·8%, 37·9%)
**Sex**	**Male**	788	366	46·5%	(43%, 49·9%)	988	542	54·9%	(51·7%, 57·9%)	4,975	2,049	41·2%	(39·8%, 42·6%)					14,017	4,977	35·5%	(34·7%, 36·3%)
	**Female**	367	217	59·1%	(54·0%, 64·0%)	977	573	58·6%	(55·5%, 61·7%)	4,390	1,833	41·8%	(40·3%, 43·2%)	1,576	643	40·8%	(38·4%, 43·2%)	12,122	4,786	39·5%	(38·6%, 40·4%)
**Diagnosis**	**2012**	…	…	…	…	…	…	…	…	…	…	…	…	…	…	…	…	…	…	…	…
**year**	**2013**	303	161	53·1%	(47·5%, 58·7%)	490	289	59·0%	(54·6%, 63·2%)	2,339	983	42·0%	(40·0%, 44·0%)	423	165	39·0%	(34·5%, 43·7%)	6,537	2,490	38·1%	(36·9%, 39·3%)
	**2014**	256	132	51·6%	(45·5%, 57·6%)	497	294	59·2%	(54·8%, 63·4%)	2,317	978	42·2%	(40·2%, 44·2%)	392	164	41·8%	(37·1%, 46·8%)	6,586	2,482	37·7%	(36·5%, 38·9%)
	**2015**	322	172	53·4%	(48·0%, 58·8%)	504	279	55·4%	(51·0%, 59·6%)	2,346	926	39·5%	(37·5%, 41·5%)	366	150	41·0%	(36·1%, 46·1%)	6,545	2,408	36·8%	(35·6%, 38·0%)
	**2016**	274	118	43·1%	(37·3%, 49%)	474	253	53·4%	(48·9%, 57·8%)	2,363	995	42·1%	(40·1%, 44·1%)	395	164	41·5%	(36·8%, 46·4%)	6,471	2,383	36·8%	(35·7%, 38·0%)
**Age group**	**15-64**	276	111	40·2%	(34·6%, 46·1%)	497	253	50·9%	(46·5%, 55·3%)	2,082	826	39·7%	(37·6%, 41·8%)	688	227	33·0%	(29·6%, 36·6%)	6,645	2,133	32·1%	(31%, 33·2%)
	**65-74**	393	179	45·5%	(40·7%, 50·5%)	599	317	52·9%	(48·9%, 56·9%)	3,335	1,222	36·6%	(35·0%, 38·3%)	442	177	40·1%	(35·6%, 44·7%)	8,453	2,791	33.0%	(32·0%, 34·0%)
	**75-84**	347	196	56·5%	(51·2%, 61·6%)	587	334	56·9%	(52·9%, 60·8%)	2,853	1,233	43·2%	(41·4%, 45·0%)	322	168	52·2%	(46·7%, 57·6%)	7,758	3,088	39·8%	(38·7%, 40·9%)
	**85+**	139	97	69·8%	(61·7%, 76·8%)	282	211	74·8%	(69·4%, 79·5%)	1,095	601	54·9%	(51·9%, 57·8%)	124	71	57·3%	(48·5%, 65·6%)	3,283	1,751	53·3%	(51·6%, 55·0%)
**Stage**	**1**	22	8	36·4%	(19·7%, 57·0%)	73	33	45·2%	(34·3%, 56·6%)	1,326	288	21·7%	(19·6%, 24·0%)	412	97	23·5%	(19·7%, 27·9%)	3,310	575	17·4%	(16·1%, 18·7%)
	**2**	40	10	25·0%	(14·2%, 40·2%)	172	93	54·1%	(46·6%, 61·3%)	714	165	23·1%	(20·2%, 26·3%)	76	16	21·1%	(13·4%, 31·5%)	3,451	941	27·3%	(25·8%, 28·8%)
	**3**	47	18	38·3%	(25·8%, 52·6%)	153	81	52·9%	(45·1%, 60·7%)	2,074	615	29·7%	(27·7%, 31·7%)	398	188	47·2%	(42·4%, 52·1%)	6,038	1,680	27·8%	(26·7%, 29·0%)
	**4**	246	138	56·1%	(49·8%, 62·2%)	955	539	56·4%	(53·3%, 59·6%)	4,400	2,363	53·7%	(52·2%, 55·2%)	248	138	55·6%	(49·4%, 61·7%)	9,006	4,485	49·8%	(48·8%, 50·8%)
	**Unknown**	800	409	51·1%	(47·7%, 54·6%)	612	369	60·3%	(56·4%, 64·1%)	851	451	53·0%	(49·6%, 56·3%)	442	204	46·2%	(41·6%, 50·8%)	4,334	2,082	48·0%	(46·6%, 49·5%)

Alberta		Oesophageal				Stomach				Colon				Rectal			
		All	EP	%EP	95% CI	All	EP	%EP	95% CI	All	EP	%EP	95% CI	All	EP	%EP	95% CI
	**Total**	708	163	23.0%	(20·1%, 26·3%)	1,020	353	34·6%	(31·8%, 37·6%)	5,142	1,471	28·6%	(27·4%, 29·9%)	1,976	254	12·9%	(11·5%, 14·4%)
**Sex**	**Male**	567	126	22·2%	(19·0%, 25·8%)	656	223	34.0%	(30·5%, 37·7%)	2,738	744	27·2%	(25·5%, 28·9%)	1,258	154	12·2%	(10·5%, 14·2%)
	**Female**	141	37	26·2%	(19·7%, 34·1%)	364	130	35·7%	(31·0%, 40·8%)	2,404	727	30·2%	(28·4%, 32·1%)	718	100	13·9%	(11·6%, 16·7%)
**Diagnosis year**	**2012**	168	29	17·3%	(12·3%, 23·7%)	253	79	31·2%	(25·8%, 37·2%)	1,166	355	30·4%	(27·9%, 33·1%)	499	74	14·8%	(12·0%, 18·2%)
	**2013**	184	47	25·5%	(19·8%, 32·3%)	231	82	35·5%	(29·6%, 41·9%)	1,214	392	32·3%	(29·7%, 35·0%)	454	62	13·7%	(10·8%, 17·1%)
	**2014**	179	37	20·7%	(15·4%, 27·2%)	248	86	34·7%	(29·0%, 40·8%)	1,416	374	26·4%	(24·2%, 28·8%)	506	60	11·9%	(9·3%, 15·0%)
	**2015**	177	50	28·2%	(22·1%, 35·3%)	288	106	36·8%	(31·4%, 42·5%)	1,346	350	26.0%	(23·7%, 28·4%)	517	58	11·2%	(8·8%, 14·2%)
	**2016**	…	…	…	…	…	…	…	…	…	…	…	…	…	…	…	…
**Age group**	**15-64**	341	71	20·8%	(16·8%, 25·4%)	406	113	27·8%	(23·7%, 32·4%)	2,007	539	26·9%	(25·0%, 28·8%)	1,002	85	8·5%	(6·9%, 10·4%)
	**65-74**	184	28	15·2%	(10·7%, 21·1%)	243	66	27·2%	(22·0%, 33·1%)	1,355	288	21·3%	(19·2%, 23·5%)	503	60	11·9%	(9·4%, 15·1%)
	**75-84**	127	42	33·1%	(25·5%, 41·6%)	241	99	41·1%	(35·1%, 47·4%)	1,240	384	31.0%	(28·5%, 33·6%)	357	69	19·3%	(15·6%, 23·7%)
	**85+**	56	22	39·3%	(27·6%, 52·4%)	130	75	57·7%	(49·1%, 65·8%)	540	260	48·1%	(44·0%, 52·4%)	114	40	35·1%	(26·9%, 44·2%)
**Stage**	**1**	66	6	9·1%	(4·2%, 18·4%)	144	32	22·2%	(16·2%, 29·7%)	1,214	181	14·9%	(13·0%, 17·0%)	488	19	3·9%	(2·5%, 6·0%)
	**2**	87	15	17·2%	(10·7%, 26·5%)	112	36	32·1%	(24·2%, 41·3%)	1,251	354	28·3%	(25·9%, 30·9%)	314	44	14.0%	(10·6%, 18·3%)
	**3**	150	25	16·7%	(11·6%, 23·4%)	183	49	26·8%	(20·9%, 33·6%)	1,277	341	26·7%	(24·3%, 29·2%)	667	64	9·6%	(7·6%, 12·1%)
	**4**	302	89	29·5%	(24·6%, 34·8%)	450	186	41·3%	(36·9%, 45·9%)	1,167	511	43·8%	(41·0%, 46·6%)	369	105	28·5%	(24·1%, 33·3%)
	**Unknown**	103	28	27·2%	(19·5%, 36·5%)	131	50	38·2%	(30·3%, 46·7%)	233	84	36·1%	(30·2%, 42·4%)	138	22	15·9%	(10·8%, 23·0%)

		Liver				Pancreatic				Lung				Ovarian				All cancers			
		All	EP	%EP	95% CI	All	EP	%EP	95% CI	All	EP	%EP	95% CI	All	EP	%EP	95% CI	All	EP	%EP	95% CI
	**Total**	1,054	334	31·7%	(29·0%, 34·6%)	1,671	683	40·9%	(38·5%, 43·2%)	7,468	2,484	33·3%	(32·2%, 34·3%)	930	239	25·7%	(23·0%, 28·6%)	19,969	5,981	30·0%	(29·3%, 30·6%)
**Sex**	**Male**	736	235	31·9%	(28·7%, 35·4%)	817	338	41·4%	(38·0%, 44·8%)	3,683	1,296	35·2%	(33·7%, 36·7%)					10,455	3,116	29·8%	(28·9%, 30·7%)
	**Female**	318	99	31·1%	(26·3%, 36·4%)	854	345	40·4%	(37·2%, 43·7%)	3,785	1,188	31·4%	(29·9%, 32·9%)	930	239	25·7%	(23·0%, 28·6%)	9,514	2,865	30·1%	(29·2%, 31·0%)
**Diagnosis year**	**2012**	239	79	33·1%	(27·4%, 39·2%)	362	151	41·7%	(36·7%, 46·9%)	1,721	578	33·6%	(31·4%, 35·9%)	220	47	21·4%	(16·5%, 27·2%)	4,628	1,392	30·1%	(28·8%, 31·4%)
	**2013**	251	73	29·1%	(23·8%, 35·0%)	454	182	40·1%	(35·7%, 44·7%)	1,795	591	32·9%	(30·8%, 35·1%)	229	56	24·5%	(19·3%, 30·4%)	4,812	1,485	30·9%	(29·6%, 32·2%)
	**2014**	262	84	32·1%	(26·7%, 37·9%)	434	182	41·9%	(37·4%, 46·6%)	2,021	677	33·5%	(31·5%, 35·6%)	241	70	29·0%	(23·7%, 35·1%)	5,307	1,570	29·6%	(28·4%, 30·8%)
	**2015**	302	98	32·5%	(27·4%, 37·9%)	421	168	39·9%	(35·3%, 44·7%)	1,931	638	33.0%	(31·0%, 35·2%)	240	66	27·5%	(22·2%, 33·5%)	5,222	1,534	29·4%	(28·2%, 30·6%)
	**2016**	…	…	…	…	…	…	…	…	…	…	…	…	…	…	…	…	…	…	…	…
**Age group**	**15-64**	535	136	25·4%	(21·9%, 29·3%)	582	186	32.0%	(28·3%, 35·9%)	2,301	706	30·7%	(28·8%, 32·6%)	527	111	21·1%	(17·8%, 24·7%)	7,701	1,947	25·3%	(24·3%, 26·3%)
	**65-74**	267	80	30.0%	(24·8%, 35·7%)	435	174	40.0%	(35·5%, 44·7%)	2,538	757	29·8%	(28·1%, 31·6%)	189	39	20·6%	(15·5%, 27·0%)	5,714	1,492	26·1%	(25·0%, 27·3%)
	**75-84**	194	79	40·7%	(34·1%, 47·8%)	425	193	45·4%	(40·7%, 50·2%)	1,982	718	36·2%	(34·1%, 38·4%)	145	51	35·2%	(27·9%, 43·2%)	4,711	1,635	34·7%	(33·4%, 36·1%)
	**85+**	58	39	67·2%	(54·4%, 77·9%)	229	130	56·8%	(50·3%, 63·0%)	647	303	46·8%	(43·0%, 50·7%)	69	38	55·1%	(43·4%, 66·2%)	1,843	907	49·2%	(46·9%, 51·5%)
**Stage**	**1**	277	49	17·7%	(13·6%, 22·6%)	112	26	23·2%	(16·4%, 31·8%)	1,361	159	11·7%	(10·1%, 13·5%)	165	19	11·5%	(7·5%, 17·3%)	3,827	491	12·8%	(11·8%, 13·9%)
	**2**	176	37	21.0%	(15·7%, 27·6%)	414	134	32·4%	(28·0%, 37·0%)	599	94	15·7%	(13·0%, 18·8%)	89	11	12·4%	(7·0%, 20·8%)	3,042	725	23·8%	(22·4%, 25·4%)
	**3**	217	87	40·1%	(33·8%, 46·7%)	127	39	30·7%	(23·4%, 39·2%)	1,329	313	23·6%	(21·3%, 25·9%)	470	120	25·5%	(21·8%, 29·7%)	4,420	1,038	23·5%	(22·3%, 24·8%)
	**4**	272	113	41·5%	(35·8%, 47·5%)	919	436	47·4%	(44·2%, 50·7%)	4,080	1,883	46·2%	(44·6%, 47·7%)	174	76	43·7%	(36·5%, 51·1%)	7,733	3,399	44.0%	(42·9%, 45·1%)
	**Unknown**	112	48	42·9%	(34·1%, 52·1%)	99	48	48·5%	(38·9%, 58·2%)	99	35	35·4%	(26·6%, 45·2%)	32	13	40·6%	(25·5%, 57·7%)	947	328	34·6%	(31·7%, 37·7%)

Atlantic Canada $		Oesophageal				Stomach				Colon				Rectal			
		All	EP	%EP	95% CI	All	EP	%EP	95% CI	All	EP	%EP	95% CI	All	EP	%EP	95% CI
	**Total**	675	140	20·7%	(17·9%, 24%)	1,150	350	30·4%	(27·8%, 33·2%)	6,135	1,745	28·4%	(27·3%, 29·6%)	2,100	265	12·6%	(11·3%, 14·1%)
**Sex**	**Male**	535	110	20·6%	(17·4%, 24·2%)	775	230	29·7%	(26·6%, 33·0%)	3,250	885	27·2%	(25·7%, 28·8%)	1,395	165	11·8%	(10·2%, 13·6%)
	**Female**	150	30	20·0%	(14·4%, 27·1%)	370	120	32·4%	(27·9%, 37·4%)	2,890	860	29·8%	(28·1%, 31·5%)	710	100	14·1%	(11·7%, 16·8%)
**Diagnosis year**	**2012**	150	45	30·0%	(23·2%, 37·8%)	280	95	33·9%	(28·6%, 39·7%)	1,515	420	27·7%	(25·5%, 30·0%)	520	60	11·5%	(9·1%, 14·6%)
	**2013**	175	30	17·1%	(12·3%, 23·4%)	295	90	30·5%	(25·5%, 36·0%)	1,540	430	27·9%	(25·7%, 30·2%)	535	70	13·1%	(10·5%, 16·2%)
	**2014**	180	35	19·4%	(14·3%, 25·8%)	295	90	30·5%	(25·5%, 36·0%)	1,525	455	29·8%	(27·6%, 32·2%)	510	70	13·7%	(11·0%, 17·0%)
	**2015**	180	40	22·2%	(16·8%, 28·8%)	275	90	32·7%	(27·5%, 38·5%)	1,550	430	27·7%	(25·6%, 30·0%)	535	70	13·1%	(10·5%, 16·2%)
	**2016**	…	…	…	…	…	…	…	…	…	…	…	…	…	…	…	…
**Age group**	**15-64**	270	45	16·7%	(12·7%, 21·6%)	370	75	20·3%	(16·5%, 24·7%)	1,890	465	24·6%	(22·7%, 26·6%)	890	85	9·6%	(7·8%, 11·7%)
	**65-74**	220	45	20·5%	(15·7%, 26·3%)	355	105	29·6%	(25·1%, 34·5%)	1,945	455	23·4%	(21·6%, 25·3%)	700	70	10·0%	(8·0%, 12·4%)
	**75-84**	130	40	30·8%	(23·5%, 39·2%)	290	100	34·5%	(29·2%, 40·1%)	1,635	520	31·8%	(29·6%, 34·1%)	380	60	15·8%	(12·5%, 19·8%)
	**85+**	65	20	30·8%	(20·9%, 42·8%)	130	70	53·8%	(45·3%, 62·2%)	670	305	45·5%	(41·8%, 49·3%)	130	45	34·6%	(27·0%, 43·1%)
**Stage**	**1**	115	20	17·4%	(11·5%, 25·3%)	160	35	21·9%	(16·2%, 28·9%)	1,345	190	14·1%	(12·4%, 16·1%)	505	40	7·9%	(5·9%, 10·6%)
	**2**	85	20	23·5%	(15·8%, 33·6%)	80	25	31·3%	(22·2%, 42·1%)	1,655	420	25·4%	(23·3%, 27·5%)	385	40	10·4%	(7·7%, 13·8%)
	**3**	90	15	16·7%	(10·4%, 25·7%)	100	30	30·0%	(21·9%, 39·6%)	1,550	460	29·7%	(27·5%, 32·0%)	680	55	8·1%	(6·3%, 10·4%)
	**4**	160	45	28·1%	(21·7%, 35·5%)	280	115	41·1%	(35·5%, 46·9%)	1,320	560	42·4%	(39·8%, 45·1%)	410	95	23·2%	(19·3%, 27·5%)
	**Unknown**	230	50	21·7%	(16·9%, 27·5%)	525	155	29·5%	(25·8%, 33·6%)	265	110	41·5%	(35·7%, 47·5%)	125	30	24·0%	(17·4%, 32·2%)

		Liver				Pancreatic				Lung				Ovarian				All cancers			
		All	EP	%EP	95% CI	All	EP	%EP	95% CI	All	EP	%EP	95% CI	All	EP	%EP	95% CI	All	EP	%EP	95% CI
	**Total**	685	235	34·3%	(30·8%, 37·9%)	1,445	560	38·8%	(36·3%, 41·3%)	9,135	2,430	26·6%	(25·7%, 27·5%)	800	225	28·1%	(25·1%, 31·3%)	22,125	5,950	26·9%	(26·3%, 27·5%)
**Sex**	**Male**	460	135	29·3%	(25·4%, 33·7%)	765	290	37·9%	(34·5%, 41·4%)	4,940	1,375	27·8%	(26·6%, 29·1%)					12,120	3,190	26·3%	(25·5%, 27·1%)
	**Female**	225	100	44·4%	(38·1%, 51·0%)	685	270	39·4%	(35·8%, 43·1%)	4,195	1,055	25·1%	(23·9%, 26·5%)	795	225	28·3%	(25·3%, 31·5%)	10,020	2,760	27·5%	(26·7%, 28·4%)
**Diagnosis year**	**2012**	180	70	38·9%	(32·1%, 46·2%)	335	120	35·8%	(30·9%, 41·1%)	2,220	590	26·6%	(24·8%, 28·5%)	205	50	24·4%	(19·0%, 30·7%)	5,405	1,450	26·8%	(25·7%, 28·0%)
	**2013**	160	55	34·4%	(27·5%, 42·0%)	395	160	40·5%	(35·8%, 45·4%)	2,240	565	25·2%	(23·5%, 27·1%)	215	65	30·2%	(24·5%, 36·7%)	5,555	1,465	26·4%	(25·2%, 27·5%)
	**2014**	155	45	29·0%	(22·5%, 36·6%)	360	130	36·1%	(31·3%, 41·2%)	2,425	680	28·0%	(26·3%, 29·9%)	210	60	28·6%	(22·9%, 35·0%)	5,660	1,565	27·7%	(26·5%, 28·8%)
	**2015**	190	65	34·2%	(27·8%, 41·2%)	350	145	41·4%	(36·4%, 46·7%)	2,250	580	25·8%	(24·0%, 27·6%)	170	50	29·4%	(23·1%, 36·7%)	5,500	1,470	26·7%	(25·6%, 27·9%)
	**2016**	…	…	…	…	…	…	…	…	…	…	…	…	…	…	…	…	…	…	…	…
**Age group**	**15-64**	245	75	30·6%	(25·2%, 36·6%)	455	155	34·1%	(29·9%, 38·5%)	2,630	605	23·0%	(21·4%, 24·7%)	360	65	18·1%	(14·4%, 22·4%)	7,110	1,570	22·1%	(21·1%, 23·1%)
	**65-74**	230	70	30·4%	(24·8%, 36·7%)	460	165	35·9%	(31·6%, 40·4%)	3,335	810	24·3%	(22·9%, 25·8%)	215	65	30·2%	(24·5%, 36·7%)	7,460	1,785	23·9%	(23·0%, 24·9%)
	**75-84**	145	60	41·4%	(33·7%, 49·5%)	360	155	43·1%	(38·0%, 48·2%)	2,425	715	29·5%	(27·7%, 31·3%)	145	60	41·4%	(33·7%, 49·5%)	5,510	1,710	31·0%	(29·8%, 32·3%)
	**85+**	55	30	54·5%	(41·5%, 67·0%)	170	80	47·1%	(39·7%, 54·5%)	745	300	40·3%	(36·8%, 43·8%)	70	40	57·1%	(45·5%, 68·1%)	2,035	890	43·7%	(41·6%, 45·9%)
**Stage**	**1**	110	20	18·2%	(12·1%, 26·4%)	125	40	32.0%	(24·5%, 40·6%)	2,055	200	9·7%	(8·5%, 11·1%)	135	15	11·1%	(6·8%, 17·5%)	4,550	560	12·3%	(11·4%, 13·3%)
	**2**	55	5	9·1%	(3·9%, 19·6%)	180	45	25·0%	(19·2%, 31·8%)	765	95	12·4%	(10·3%, 14·9%)	45	10	22·2%	(12·5%, 36·3%)	3,250	660	20·3%	(19·0%, 21·7%)
	**3**	55	20	36·4%	(24·9%, 49·6%)	55	15	27·3%	(17·3%, 40·2%)	1,745	325	18·6%	(16·9%, 20·5%)	240	50	20·8%	(16·2%, 26·4%)	4,515	970	21·5%	(20·3%, 22·7%)
	**4**	125	35	28·0%	(20·9%, 36·4%)	480	185	38·5%	(34·3%, 43·0%)	4,395	1,750	39·8%	(38·4%, 41·3%)	105	45	42·9%	(33·8%, 52·4%)	7,275	2,830	38·9%	(37·8%, 40·0%)
	**Unknown**	335	145	43·3%	(38·1%, 48·6%)	600	270	45·0%	(41·1%, 49·0%)	165	60	36·4%	(29·4%, 43·9%)	285	105	36·8%	(31·5%, 42·6%)	2,530	925	36·6%	(34·7%, 38·5%)

British Columbia $		Oesophageal				Stomach				Colon				Rectal			
		All	EP	%EP	95% CI	All	EP	%EP	95% CI	All	EP	%EP	95% CI	All	EP	%EP	95% CI
	**Total**	1,165	330	28·3%	(25·8%, 31·0%)	1,590	640	40·3%	(37·9%, 42·7%)	9,160	2,520	27·5%	(26·6%, 28·4%)	3,430	410	12·0%	(10·9%, 13·1%)
**Sex**	**Male**	865	235	27·2%	(24·3%, 30·2%)	1,055	410	38·9%	(36·0%, 41·8%)	4,855	1,275	26·3%	(25·0%, 27·5%)	2,170	255	11·8%	(10·5%, 13·2%)
	**Female**	305	95	31·2%	(26·2%, 36·6%)	535	230	43·0%	(38·9%, 47·2%)	4,305	1,245	28·9%	(27·6%, 30·3%)	1,260	155	12·3%	(10·6%, 14·2%)
**Diagnosis year**	**2012**	305	75	24·6%	(20·1%, 29·7%)	400	165	41·3%	(36·5%, 46·1%)	2,205	660	29·9%	(28·1%, 31·9%)	825	100	12·1%	(10·1%, 14·5%)
	**2013**	265	65	24·5%	(19·7%, 30·0%)	370	155	41·9%	(37·0%, 47·0%)	2,345	700	29·9%	(28·0%, 31·7%)	825	125	15·2%	(12·9%, 17·8%)
	**2014**	295	90	30·5%	(25·5%, 36·0%)	415	160	38·6%	(34·0%, 43·3%)	2,390	630	26·4%	(24·6%, 28·2%)	855	90	10·5%	(8·6%, 12·8%)
	**2015**	300	90	30·0%	(25·1%, 35·4%)	410	160	39.0%	(34·4%, 43·8%)	2,225	525	23·6%	(21·9%, 25·4%)	920	95	10·3%	(8·5%, 12·5%)
	**2016**	…	…	…	…	…	…	…	…	…	…	…	…	…	…	…	…
**Age group**	**15-64**	400	105	26·3%	(22·2%, 30·8%)	510	175	34·3%	(30·3%, 38·5%)	2,790	700	25·1%	(23·5%, 26·7%)	1,450	120	8·3%	(7·0%, 9·8%)
	**65-74**	355	85	23·9%	(19·8%, 28·6%)	440	140	31·8%	(27·6%, 36·3%)	2,660	545	20·5%	(19·0%, 22·1%)	990	95	9·6%	(7·9%, 11·6%)
	**75-84**	295	80	27·1%	(22·4%, 32·5%)	425	180	42·4%	(37·7%, 47·1%)	2,475	680	27·5%	(25·8%, 29·3%)	690	95	13·8%	(11·4%, 16·5%)
	**85+**	115	55	47·8%	(38·9%, 56·9%)	215	135	62·8%	(56·2%, 69·0%)	1,240	600	48·4%	(45·6%, 51·2%)	290	105	36·2%	(30·9%, 41·9%)
**Stage**	**1**	…	…	…	…	…	…	…	…	1,855	180	9·7%	(8·4%, 11·1%)	610	35	5·7%	(4·2%, 7·9%)
	**2**	…	…	…	…	…	…	…	…	2,240	625	27·9%	(26·1%, 29·8%)	630	65	10·3%	(8·2%, 12·9%)
	**3**	…	…	…	…	…	…	…	…	2,330	635	27·3%	(25·5%, 29·1%)	1,050	100	9·5%	(7·9%, 11·4%)
	**4**	…	…	…	…	…	…	…	…	1,785	780	43·7%	(41·4%, 46·0%)	555	125	22·5%	(19·2%, 26·2%)
	**Unknown**	1,165	325	27·9%	(25·4%, 30·5%)	1,590	640	40·3%	(37·9%, 42·7%)	950	295	31·1%	(28·2%, 34·1%)	590	80	13·6%	(11·0%, 16·6%)

		Liver				Pancreatic				Lung				Ovarian				All cancers			
		All	EP	%EP	95% CI	All	EP	%EP	95% CI	All	EP	%EP	95% CI	All	EP	%EP	95% CI	All	EP	%EP	95% CI
	**Total**	1,760	670	38·1%	(35·8%, 40·4%)	2,620	1,085	41·4%	(39·5%, 43·3%)	12,320	4,090	33·2%	(32·4%, 34·0%)	1,610	510	31·7%	(29·5%, 34·0%)	33,655	10,255	30·5%	(30·0%, 31·0%)
**Sex**	**Male**	1,245	460	36·9%	(34·3%, 39·7%)	1,350	560	41·5%	(38·9%, 44·1%)	6,105	2,135	35.0%	(33·8%, 36·2%)					17,645	5,330	30·2%	(29·5%, 30·9%)
	**Female**	515	210	40·8%	(36·6%, 45·1%)	1,265	525	41·5%	(38·8%, 44·2%)	6,210	1,955	31·5%	(30·3%, 32·6%)	1,615	510	31·6%	(29·4%, 33·9%)	16,010	4,925	30·8%	(30·1%, 31·5%)
**Diagnosis year**	**2012**	410	150	36·6%	(32·1%, 41·4%)	605	245	40·5%	(36·7%, 44·5%)	3,005	985	32·8%	(31·1%, 34·5%)	355	120	33·8%	(29·1%, 38·9%)	8,110	2,500	30·8%	(29·8%, 31·8%)
	**2013**	465	190	40·9%	(36·5%, 45·4%)	700	300	42·9%	(39·2%, 46·6%)	3,005	1,025	34·1%	(32·4%, 35·8%)	395	130	32·9%	(28·5%, 37·7%)	8,370	2,690	32·1%	(31·1%, 33·1%)
	**2014**	450	160	35·6%	(31·3%, 40·1%)	685	290	42·3%	(38·7%, 46·1%)	3,165	1,055	33·3%	(31·7%, 35·0%)	455	135	29·7%	(25·7%, 34·0%)	8,710	2,610	30·0%	(29%, 30·9%)
	**2015**	435	170	39·1%	(34·6%, 43·7%)	630	255	40·5%	(36·7%, 44·4%)	3,135	1,025	32·7%	(31·1%, 34·4%)	405	120	29·6%	(25·4%, 34·3%)	8,460	2,440	28·8%	(27·9%, 29·8%)
	**2016**	…	…	…	…	…	…	…	…	…	…	…	…	…	…	…	…	…	…	…	…
**Age group**	**15-64**	720	240	33·3%	(30·0%, 36·9%)	750	285	38.0%	(34·6%, 41·5%)	3,285	990	30·1%	(28·6%, 31·7%)	795	205	25·8%	(22·9%, 28·9%)	10,700	2,820	26·4%	(25·5%, 27·2%)
	**65-74**	520	165	31·7%	(27·9%, 35·9%)	785	285	36·3%	(33·0%, 39·7%)	3,985	1,175	29·5%	(28·1%, 30·9%)	405	120	29·6%	(25·4%, 34·3%)	10,140	2,610	25·7%	(24·9%, 26·6%)
	**75-84**	360	165	45·8%	(40·8%, 51·0%)	690	305	44·2%	(40·5%, 47·9%)	3,645	1,255	34·4%	(32·9%, 36·0%)	265	95	35·8%	(30·3%, 41·8%)	8,845	2,855	32·3%	(31·3%, 33·3%)
	**85+**	165	100	60·6%	(53·0%, 67·7%)	385	210	54·5%	(49·6%, 59·4%)	1,400	670	47·9%	(45·2%, 50·5%)	140	85	60·7%	(52·4%, 68·4%)	3,950	1,960	49·6%	(48·1%, 51·2%)
**Stage**	**1**	…	…	…	…	…	…	…	…	2,035	210	10·3%	(9·1%, 11·7%)	…	…	…	…	4,500	425	9·4%	(8·6%, 10·3%)
	**2**	…	…	…	…	…	…	…	…	940	145	15·4%	(13·3%, 17·9%)	…	…	…	…	3,810	835	21·9%	(20·6%, 23·3%)
	**3**	…	…	…	…	…	…	…	…	2,500	645	25·8%	(24·1%, 27·6%)	…	…	…	…	5,880	1,380	23·5%	(22·4%, 24·6%)
	**4**	…	…	…	…	…	…	…	…	6,165	2,810	45·6%	(44·3%, 46·8%)	…	…	…	…	8,505	3,715	43·7%	(42·6%, 44·7%)
	**Unknown**	1,760	665	37·8%	(35·5%, 40·1%)	2,615	1,085	41·5%	(39·6%, 43·4%)	680	270	39·7%	(36·1%, 43·4%)	1,615	510	31·6%	(29·4%, 33·9%)	10,965	3,870	35·3%	(34·4%, 36·2%)

Ontario		Oesophageal				Stomach				Colon				Rectal			
		All	EP	%EP	95% CI	All	EP	%EP	95% CI	All	EP	%EP	95% CI	All	EP	%EP	95% CI
	**Total**	3,099	630	20·3%	(18·9%, 21·8%)	6,465	1,738	26·9%	(25·8%, 28·0%)	24,481	6,653	27·2%	(26·6%, 27·7%)	7,845	860	11·0%	(10·3%, 11·7%)
**Sex**	**Male**	2,355	450	19·1%	(17·6%, 20·7%)	4,065	1,089	26·8%	(25·5%, 28·2%)	12,795	3,298	25·8%	(25·0%, 26·5%)	4,819	517	10·7%	(9·9%, 11·6%)
	**Female**	744	180	24·2%	(21·3%, 27·4%)	2,400	649	27·0%	(25·3%, 28·9%)	11,686	3,355	28·7%	(27·9%, 29·5%)	3,026	343	11·3%	(10·3%, 12·5%)
**Diagnosis year**	**2012**	624	121	19·4%	(16·5%, 22·7%)	1,199	316	26·4%	(23·9%, 28·9%)	4,984	1,271	25·5%	(24·3%, 26·7%)	1,549	193	12·5%	(10·9%, 14·2%)
	**2013**	616	130	21·1%	(18·1%, 24·5%)	1,299	339	26·1%	(23·8%, 28·6%)	4,809	1,281	26·6%	(25·4%, 27·9%)	1,542	175	11·3%	(9·9%, 13%)
	**2014**	570	117	20·5%	(17·4%, 24·0%)	1,260	362	28·7%	(26·3%, 31·3%)	4,913	1,336	27·2%	(26·0%, 28·5%)	1,578	170	10·8%	(9·3%, 12·4%)
	**2015**	650	135	20·8%	(17·8%, 24·1%)	1,348	354	26·3%	(24·0%, 28·7%)	4,985	1,444	29·0%	(27·7%, 30·2%)	1,547	148	9·6%	(8·2%, 11·1%)
	**2016**	639	127	19·9%	(17·0%, 23·1%)	1,359	367	27·0%	(24·7%, 29·4%)	4,790	1,321	27·6%	(26·3%, 28·9%)	1,629	174	10·7%	(9·3%, 12·3%)
**Age group**	**15-64**	1,222	210	17·2%	(15·2%, 19·4%)	2,301	456	19·8%	(18·2%, 21·5%)	8,441	1,993	23·6%	(22·7%, 24·5%)	3,664	241	6·6%	(5·8%, 7·4%)
	**65-74**	920	146	15·9%	(13·7%, 18·4%)	1,714	366	21·4%	(19·5%, 23·4%)	6,558	1,347	20·5%	(19·6%, 21·5%)	2,061	232	11·3%	(10%, 12·7%)
	**75-84**	677	175	25·8%	(22·7%, 29·3%)	1,662	527	31·7%	(29·5%, 34·0%)	6,490	1,912	29·5%	(28·4%, 30·6%)	1,496	224	15·0%	(13·3%, 16·9%)
	**85+**	280	99	35·4%	(30·0%, 41·1%)	788	389	49·4%	(45·9%, 52·9%)	2,992	1,401	46·8%	(45·0%, 48·6%)	624	163	26·1%	(22·8%, 29·7%)
**Stage**	**1**	144	20	13·9%	(9·2%, 20·5%)	484	70	14·5%	(11·6%, 17·9%)	5,272	815	15·5%	(14·5%, 16·5%)	2,115	122	5·8%	(4·9%, 6·8%)
	**2**	274	42	15·3%	(11·5%, 20·1%)	498	79	15·9%	(12·9%, 19·3%)	6,561	1,877	28·6%	(27·5%, 29·7%)	1,369	153	11·2%	(9·6%, 13·0%)
	**3**	476	58	12·2%	(9·5%, 15·4%)	543	137	25·2%	(21·8%, 29·0%)	6,590	1,827	27·7%	(26·7%, 28·8%)	2,489	233	9·4%	(8·3%, 10·6%)
	**4**	748	161	21·5%	(18·7%, 24·6%)	1,135	310	27·3%	(24·8%, 30·0%)	4,373	1,745	39·9%	(38·5%, 41·4%)	1,130	261	23·1%	(20·7%, 25·6%)
	**Unknown**	1,457	349	24.0%	(21·8%, 26·2%)	3,805	1,142	30·0%	(28·6%, 31·5%)	1,685	389	23·1%	(21·1%, 25·2%)	742	91	12·3%	(10·1%, 14·8%)

		Liver				Pancreatic				Lung				Ovarian				All cancers			
		All	EP	%EP	95% CI	All	EP	%EP	95% CI	All	EP	%EP	95% CI	All	EP	%EP	95% CI	All	EP	%EP	95% CI
	**Total**	4,129	1,167	28·3%	(26·9%, 29·7%)	6,456	2,279	35·3%	(34·1%, 36·5%)	35,092	9,640	27·5%	(27·0%, 27·9%)	4,880	1,164	23·9%	(22·7%, 25·1%)	92,447	24,131	26·1%	(25·8%, 26·4%)
**Sex**	**Male**	2,946	828	28·1%	(26·5%, 29·8%)	3,473	1,221	35·2%	(33·6%, 36·8%)	17,693	5,031	28·4%	(27·8%, 29·1%)					48,146	12,434	25·8%	(25·4%, 26·2%)
	**Female**	1,183	339	28·7%	(26·2%, 31·3%)	2,983	1,058	35·5%	(33·8%, 37·2%)	17,399	4,609	26·5%	(25·8%, 27·2%)	4,880	1,164	23·9%	(22·7%, 25·1%)	44,301	11,697	26·4%	(26·0%, 26·8%)
**Diagnosis year**	**2012**	710	196	27·6%	(24·4%, 31·0%)	1,242	437	35·2%	(32·6%, 37·9%)	6,849	1,813	26·5%	(25·4%, 27·5%)	933	234	25·1%	(22·4%, 28·0%)	18,090	4,581	25·3%	(24·7%, 26·0%)
	**2013**	761	200	26·3%	(23·3%, 29·5%)	1,227	387	31·5%	(29·0%, 34·2%)	6,728	1,823	27·1%	(26·0%, 28·2%)	996	222	22·3%	(19·8%, 25·0%)	17,978	4,557	25·3%	(24·7%, 26·0%)
	**2014**	900	251	27·9%	(25·1%, 30·9%)	1,241	420	33·8%	(31·3%, 36·5%)	7,071	2,019	28·6%	(27·5%, 29·6%)	980	240	24·5%	(21·9%, 27·3%)	18,513	4,915	26·5%	(25·9%, 27·2%)
	**2015**	898	269	30·0%	(27·1%, 33·0%)	1,340	529	39·5%	(36·9%, 42·1%)	7,099	1,967	27·7%	(26·7%, 28·8%)	992	247	24·9%	(22·3%, 27·7%)	18,859	5,093	27·0%	(26·4%, 27·6%)
	**2016**	860	251	29·2%	(26·2%, 32·3%)	1,406	506	36.0%	(33·5%, 38·5%)	7,345	2,018	27·5%	(26·5%, 28·5%)	979	221	22·6%	(20·1%, 25·3%)	19,007	4,985	26·2%	(25·6%, 26·9%)
**Age group**	**15-64**	1,804	449	24·9%	(22·9%, 26·9%)	2,394	769	32·1%	(30·3%, 34·0%)	10,741	2,733	25·4%	(24·6%, 26·3%)	2,610	442	16·9%	(15·5%, 18·4%)	33,177	7,293	22·0%	(21·5%, 22·4%)
	**65-74**	1,204	356	29·6%	(27·1%, 32·2%)	2,091	725	34·7%	(32·7%, 36·7%)	12,271	3,183	25·9%	(25·2%, 26·7%)	1,208	288	23·8%	(21·5%, 26·3%)	28,027	6,643	23·7%	(23·2%, 24·2%)
	**75-84**	907	271	29·9%	(27·0%, 32·9%)	1,591	610	38·3%	(36·0%, 40·8%)	9,569	2,780	29·1%	(28·2%, 30·0%)	792	306	38·6%	(35·3%, 42·1%)	23,184	6,805	29·4%	(28·8%, 29·9%)
	**85+**	214	91	42·5%	(36·1%, 49·2%)	380	175	46·1%	(41·1%, 51·1%)	2,511	944	37·6%	(35·7%, 39·5%)	270	128	47·4%	(41·5%, 53·4%)	8,059	3,390	42·1%	(41·0%, 43·1%)
**Stage**	**1**	421	57	13·5%	(10·6%, 17·1%)	337	67	19·9%	(16·0%, 24·5%)	7,035	687	9·8%	(9·1%, 10·5%)	812	71	8·7%	(7·0%, 10·9%)	16,620	1,909	11·5%	(11·0%, 12·0%)
	**2**	452	53	11·7%	(9·1%, 15·0%)	874	270	30·9%	(27·9%, 34·0%)	2,780	339	12·2%	(11%, 13·5%)	369	45	12·2%	(9·2%, 15·9%)	13,177	2,858	21·7%	(21·0%, 22·4%)
	**3**	529	131	24·8%	(21·3%, 28·6%)	532	164	30·8%	(27·1%, 34·9%)	6,801	1,381	20·3%	(19·4%, 21·3%)	1,516	302	19·9%	(18·0%, 22·0%)	19,476	4,233	21·7%	(21·2%, 22·3%)
	**4**	572	188	32·9%	(29·1%, 36·8%)	2,021	628	31·1%	(29·1%, 33·1%)	17,017	6,812	40·0%	(39·3%, 40·8%)	583	226	38·8%	(34·9%, 42·8%)	27,579	10,331	37·5%	(36·9%, 38·0%)
	**Unknown**	2,155	738	34·2%	(32·3%, 36·3%)	2,692	1,150	42·7%	(40·9%, 44·6%)	1,459	421	28·9%	(26·6%, 31·2%)	1,600	520	32·5%	(30·2%, 34·8%)	15,595	4,800	30·8%	(30·1%, 31·5%)

Saskatchewan-Manitoba $		Oesophageal				Stomach				Colon				Rectal			
		All	EP	%EP	95% CI	All	EP	%EP	95% CI	All	EP	%EP	95% CI	All	EP	%EP	95% CI
	**Total**	460	85	18·5%	(15·2%, 22·3%)	895	285	31·8%	(28·9%, 35·0%)	4,470	1,240	27·7%	(26·4%, 29·1%)	1,820	250	13·7%	(12·2%, 15·4%)
**Sex**	**Male**	345	60	17·4%	(13·8%, 21·7%)	595	175	29·4%	(25·9%, 33·2%)	2,450	645	26·3%	(24·6%, 28·1%)	1,160	150	12·9%	(11·1%, 15·0%)
	**Female**	110	25	22·7%	(15·9%, 31·4%)	300	110	36·7%	(31·4%, 42·3%)	2,030	595	29·3%	(27·4%, 31·3%)	650	95	14·6%	(12·1%, 17·5%)
**Diagnosis year**	**2012**	110	15	13·6%	(8·4%, 21·3%)	230	75	32·6%	(26·9%, 38·9%)	1,095	300	27·4%	(24·8%, 30·1%)	465	65	14·0%	(11·1%, 17·4%)
	**2013**	110	20	18·2%	(12·1%, 26·4%)	205	70	34·1%	(28·0%, 40·9%)	1,190	345	29·0%	(26·5%, 31·6%)	455	60	13·2%	(10·4%, 16·6%)
	**2014**	135	30	22·2%	(16·0%, 29·9%)	230	75	32·6%	(26·9%, 38·9%)	1,155	310	26·8%	(24·4%, 29·5%)	420	55	13·1%	(10·2%, 16·7%)
	**2015**	105	25	23·8%	(16·7%, 32·8%)	230	60	26·1%	(20·8%, 32·1%)	1,040	295	28·4%	(25·7%, 31·2%)	475	65	13·7%	(10·9%, 17·1%)
	**2016**	…	…	…	…	…	…	…	…	…	…	…	…	…	…	…	…
**Age group**	**15-64**	180	25	13·9%	(9·6%, 19·7%)	305	75	24·6%	(20·1%, 29·7%)	1,515	365	24·1%	(22·0%, 26·3%)	790	75	9·5%	(7·6%, 11·7%)
	**65-74**	130	30	23·1%	(16·7%, 31·0%)	220	50	22·7%	(17·7%, 28·7%)	1,175	250	21·3%	(19·0%, 23·7%)	500	50	10·0%	(7·7%, 12·9%)
	**75-84**	100	15	15·0%	(9·3%, 23·3%)	245	90	36·7%	(30·9%, 42·9%)	1,150	340	29·6%	(27·0%, 32·3%)	355	65	18·3%	(14·6%, 22·7%)
	**85+**	45	10	22·2%	(12·5%, 36·3%)	125	70	56·0%	(47·2%, 64·4%)	635	280	44·1%	(40·3%, 48·0%)	175	55	31·4%	(25·0%, 38·6%)
**Stage**	**1**	65	15	23·1%	(14·5%, 34·6%)	165	45	27·3%	(21·1%, 34·5%)	1,065	180	16·9%	(14·8%, 19·3%)	425	35	8·2%	(6·0%, 11·2%)
	**2**	55	10	18·2%	(10·2%, 30·3%)	130	30	23·1%	(16·7%, 31·0%)	1,215	325	26·8%	(24·3%, 29·3%)	275	45	16·4%	(12·5%, 21·2%)
	**3**	100	15	15·0%	(9·3%, 23·3%)	145	35	24·1%	(17·9%, 31·7%)	1,055	280	26·5%	(24·0%, 29·3%)	635	60	9·4%	(7·4%, 12·0%)
	**4**	185	40	21·6%	(16·3%, 28·1%)	330	135	40·9%	(35·7%, 46·3%)	905	390	43·1%	(39·9%, 46·3%)	365	85	23·3%	(19·2%, 27·9%)
	**Unknown**	50	5	10·0%	(4·3%, 21·4%)	115	45	39·1%	(30·7%, 48·3%)	235	70	29·8%	(24·3%, 35·9%)	120	20	16·7%	(11·1%, 24·3%)

		Liver				Pancreatic				Lung				Ovarian				All cancers			
		All	EP	%EP	95% CI	All	EP	%EP	95% CI	All	EP	%EP	95% CI	All	EP	%EP	95% CI	All	EP	%EP	95% CI
	**Total**	585	195	33·3%	(29·6%, 37·3%)	1,300	485	37·3%	(34·7%, 40·0%)	6,395	1,955	30·6%	(29·5%, 31·7%)	730	220	30·1%	(26·9%, 33·6%)	16,655	4,715	28·3%	(27·6%, 29·0%)
**Sex**	**Male**	390	110	28·2%	(24·0%, 32·9%)	670	230	34·3%	(30·8%, 38·0%)	3,170	1,005	31·7%	(30·1%, 33·3%)					8,780	2,375	27·1%	(26·1%, 28%)
	**Female**	200	75	37·5%	(31·1%, 44·4%)	635	260	40·9%	(37·2%, 44·8%)	3,220	950	29·5%	(28·0%, 31·1%)	730	220	30·1%	(26·9%, 33·6%)	7,875	2,330	29·6%	(28·6%, 30·6%)
**Diagnosis year**	**2012**	155	60	38·7%	(31·4%, 46·6%)	300	115	38·3%	(33·0%, 43·9%)	1,560	485	31·1%	(28·8%, 33·4%)	165	35	21·2%	(15·7%, 28·1%)	4,080	1,150	28·2%	(26·8%, 29·6%)
	**2013**	140	50	35·7%	(28·3%, 43·9%)	355	135	38·0%	(33·1%, 43·2%)	1,635	505	30·9%	(28·7%, 33·2%)	195	70	35·9%	(29·5%, 42·8%)	4,285	1,255	29·3%	(27·9%, 30·7%)
	**2014**	135	45	33·3%	(25·9%, 41·6%)	330	115	34·8%	(29·9%, 40·1%)	1,655	530	32·0%	(29·8%, 34·3%)	190	60	31·6%	(25·4%, 38·5%)	4,250	1,220	28·7%	(27·4%, 30·1%)
	**2015**	155	40	25·8%	(19·6%, 33·2%)	310	130	41·9%	(36·6%, 47·5%)	1,545	440	28·5%	(26·3%, 30·8%)	185	50	27·0%	(21·1%, 33·8%)	4,045	1,105	27·3%	(26·0%, 28·7%)
	**2016**	…	…	…	…	…	…	…	…	…	…	…	…	…	…	…	…	…	…	…	…
**Age group**	**15-64**	260	75	28·8%	(23·7%, 34·6%)	385	135	35·1%	(30·5%, 40·0%)	1,710	430	25·2%	(23·1%, 27·3%)	400	95	23·8%	(19·8%, 28·2%)	5,545	1,275	23·0%	(21·9%, 24·1%)
	**65-74**	155	45	29·0%	(22·5%, 36·6%)	380	130	34·2%	(29·6%, 39·1%)	2,265	675	29·8%	(28·0%, 31·7%)	165	45	27·3%	(21·1%, 34·5%)	4,990	1,275	25·6%	(24·4%, 26·8%)
	**75-84**	130	50	38·5%	(30·5%, 47·0%)	335	130	38·8%	(33·7%, 44·1%)	1,785	600	33·6%	(31·5%, 35·8%)	115	45	39·1%	(30·7%, 48·3%)	4,215	1,335	31·7%	(30·3%, 33·1%)
	**85+**	45	25	55·6%	(41·2%, 69·1%)	200	100	50·0%	(43·1%, 56·9%)	630	250	39·7%	(35·9%, 43·6%)	55	35	63·6%	(50·4%, 75·1%)	1,910	825	43·2%	(41·0%, 45·4%)
**Stage**	**1**	125	30	24·0%	(17·4%, 32·2%)	135	40	29·6%	(22·6%, 37·8%)	1,200	105	8·8%	(7·3%, 10·5%)	160	20	12·5%	(8·2%, 18·5%)	3,340	470	14·1%	(12·9%, 15·3%)
	**2**	85	15	17·6%	(11·0%, 27·1%)	305	100	32·8%	(27·8%, 38·2%)	500	70	14·0%	(11·2%, 17·3%)	65	15	23·1%	(14·5%, 34·6%)	2,630	610	23·2%	(21·6%, 24·8%)
	**3**	90	25	27·8%	(19·6%, 37·8%)	95	20	21·1%	(14·1%, 30·3%)	1,220	290	23·8%	(21·5%, 26·2%)	340	100	29·4%	(24·8%, 34·5%)	3,680	825	22·4%	(21·1%, 23·8%)
	**4**	115	50	43·5%	(34·8%, 52·6%)	715	295	41·3%	(37·7%, 44·9%)	3,390	1,455	42·9%	(41·3%, 44·6%)	140	70	50·0%	(41·8%, 58·2%)	6,145	2,520	41.0%	(39·8%, 42·2%)
	**Unknown**	180	75	41·7%	(34·7%, 49·0%)	60	20	33·3%	(22·7%, 45·9%)	85	40	47·1%	(36·8%, 57·6%)	25	15	60·0%	(40·7%, 76·6%)	870	290	33·3%	(30·3%, 36·5%)

New South Wales		Oesophageal				Stomach				Colon				Rectal			
		All	EP	%EP	95% CI	All	EP	%EP	95% CI	All	EP	%EP	95% CI	All	EP	%EP	95% CI
	**Total**	1,970	530	26·9%	(25·0%, 28·9%)	3,179	969	30·5%	(28·9%, 32·1%)	16,316	4499	27·6%	(26·9%, 28·3%)	5,459	660	12·1%	(11·3%, 13·0%)
**Sex**	**Male**	1,368	360	26·3%	(24·1%, 28·7%)	2,083	605	29·0%	(27·1%, 31·0%)	8,207	2204	26·9%	(25·9%, 27·8%)	3,421	406	11·9%	(10·8%, 13·0%)
	**Female**	602	170	28·2%	(24·8%, 32·0%)	1,096	364	33·2%	(30·5%, 36·1%)	8,109	2295	28·3%	(27·3%, 29·3%)	2,038	254	12·5%	(11·1%, 14·0%)
**Diagnosis year**	**2012**	369	97	26·3%	(22·1%, 31·0%)	640	210	32·8%	(29·3%, 36·5%)	3,278	911	27·8%	(26·3%, 29·4%)	1,050	139	13·2%	(11·3%, 15·4%)
	**2013**	391	101	25·8%	(21·7%, 30·4%)	624	179	28·7%	(25·3%, 32·4%)	3,121	862	27·6%	(26·1%, 29·2%)	1,052	131	12·5%	(10·6%, 14·6%)
	**2014**	438	96	21·9%	(18·3%, 26·0%)	634	181	28·5%	(25·2%, 32·2%)	3,159	878	27·8%	(26·3%, 29·4%)	1,072	123	11·5%	(9·7%, 13·5%)
	**2015**	394	118	29·9%	(25·6%, 34·6%)	599	175	29·2%	(25·7%, 33·0%)	3,390	931	27·5%	(26·0%, 29·0%)	1,084	129	11·9%	(10·1%, 14%)
	**2016**	378	118	31·2%	(26·8%, 36·1%)	682	224	32·8%	(29·4%, 36·5%)	3,368	917	27·2%	(25·8%, 28·8%)	1,201	138	11·5%	(9·8%, 13·4%)
**Age group**	**15-64**	626	164	26·2%	(22·9%, 29·8%)	1,112	283	25·4%	(23·0%, 28·1%)	4,932	1394	28·3%	(27·0%, 29·5%)	2,384	247	10·4%	(9·2%, 11·6%)
	**65-74**	598	120	20·1%	(17·1%, 23·5%)	897	209	23·3%	(20·7%, 26·2%)	4,748	966	20·3%	(19·2%, 21·5%)	1,570	134	8·5%	(7·3%, 10·0%)
	**75-84**	500	147	29·4%	(25·6%, 33·5%)	790	274	34·7%	(31·4%, 38·1%)	4,582	1238	27·0%	(25·8%, 28·3%)	1,097	167	15·2%	(13·2%, 17·5%)
	**85+**	246	99	40·2%	(34·3%, 46·5%)	380	203	53·4%	(48·4%, 58·4%)	2,054	901	43·9%	(41·7%, 46·0%)	408	112	27·5%	(23·3%, 32·0%)
**Stage**	**Localised**	645	143	22·2%	(19·1%, 25·5%)	893	216	24·2%	(21·5%, 27·1%)	4,905	907	18·5%	(17·4%, 19·6%)	1,961	152	7·8%	(6·6%, 9·0%)
	**Inv. adjc. tissue/organ**	131	32	24·4%	(17·9%, 32·4%)	217	67	30·9%	(25·1%, 37·3%)	3,379	894	26·5%	(25·0%, 28·0%)	682	85	12·5%	(10·2%, 15·2%)
	**Inv. regional LNs**	284	71	25.0%	(20·3%, 30·3%)	498	113	22·7%	(19·2%, 26·6%)	3,690	978	26·5%	(25·1%, 28·0%)	1,268	129	10·2%	(8·6%, 12·0%)
	**Distant metastasis**	551	205	37·2%	(33·3%, 41·3%)	1,004	453	45·1%	(42·1%, 48·2%)	3,246	1497	46·1%	(44·4%, 47·8%)	790	237	30.0%	(26·9%, 33·3%)
	**Unknown**	359	79	22·0%	(18·0%, 26·6%)	567	120	21·2%	(18·0%, 24·7%)	1,096	223	20·4%	(18·1%, 22·8%)	758	57	7·5%	(5·8%, 9·6%)

		Liver				Pancreatic				Lung				Ovarian				All cancers			
		All	EP	%EP	95% CI	All	EP	%EP	95% CI	All	EP	%EP	95% CI	All	EP	%EP	95% CI	All	EP	%EP	95% CI
	**Total**	3,155	1,161	36·8%	(35·1%, 38·5%)	4,498	2,036	45·3%	(43·8%, 46·7%)	16,334	5,941	36·4%	(35·6%, 37·1%)	2287	649	28·4%	(26·6%, 30·3%)	53,198	16,445	30·9%	(30·5%, 31·3%)
**Sex**	**Male**	2,329	821	35·3%	(33·3%, 37·2%)	2,315	1,029	44·5%	(42·4%, 46·5%)	9,314	3,500	37·6%	(36·6%, 38·6%)					29,037	8,925	30·7%	(30·2%, 31·3%)
	**Female**	826	340	41·2%	(37·9%, 44·6%)	2,183	1,007	46·1%	(44·0%, 48·2%)	7,020	2,441	34·8%	(33·7%, 35·9%)	2287	649	28·4%	(26·6%, 30·3%)	24,161	7,520	31·1%	(30·5%, 31·7%)
**Diagnosis year**	**2012**	543	187	34·4%	(30·6%, 38·5%)	847	373	44·0%	(40·7%, 47·4%)	3,189	1,240	38·9%	(37·2%, 40·6%)	445	137	30·8%	(26·7%, 35·2%)	10,361	3,294	31·8%	(30·9%, 32·7%)
	**2013**	626	235	37·5%	(33·8%, 41·4%)	846	357	42·2%	(38·9%, 45·6%)	3,252	1,205	37·1%	(35·4%, 38·7%)	456	127	27·9%	(23·9%, 32·1%)	10,368	3,197	30·8%	(30·0%, 31·7%)
	**2014**	634	234	36·9%	(33·2%, 40·7%)	901	391	43·4%	(40·2%, 46·7%)	3,138	1,079	34·4%	(32·7%, 36·1%)	456	130	28·5%	(24·6%, 32·8%)	10,432	3,112	29·8%	(29·0%, 30·7%)
	**2015**	664	248	37·3%	(33·8%, 41·1%)	942	446	47·3%	(44·2%, 50·5%)	3,357	1,222	36·4%	(34·8%, 38·0%)	473	121	25·6%	(21·9%, 29·7%)	10,903	3,390	31·1%	(30·2%, 32·0%)
	**2016**	688	257	37·4%	(33·8%, 41%)	962	469	48·8%	(45·6%, 51·9%)	3,398	1,195	35·2%	(33·6%, 36·8%)	457	134	29·3%	(25·3%, 33·7%)	11,134	3,452	31·0%	(30·2%, 31·9%)
**Age group**	**15-64**	1,492	486	32·6%	(30·2%, 35%)	1,355	515	38·0%	(35·5%, 40·6%)	4,769	1,591	33·4%	(32·0%, 34·7%)	1038	233	22·4%	(20·0%, 25·1%)	17,708	4,913	27·7%	(27·1%, 28·4%)
	**65-74**	827	277	33·5%	(30·4%, 36·8%)	1,310	518	39·5%	(36·9%, 42·2%)	5,528	1,807	32·7%	(31·5%, 33·9%)	607	159	26·2%	(22·9%, 29·8%)	16,085	4,190	26·0%	(25·4%, 26·7%)
	**75-84**	617	264	42·8%	(38·9%, 46·7%)	1,235	611	49·5%	(46·7%, 52·3%)	4,536	1,745	38·5%	(37·1%, 39·9%)	445	159	35·7%	(31·4%, 40·3%)	13,802	4,605	33·4%	(32·6%, 34·2%)
	**85+**	219	134	61·2%	(54·6%, 67·4%)	598	392	65·6%	(61·7%, 69·3%)	1,501	798	53·2%	(50·6%, 55·7%)	197	98	49·7%	(42·8%, 56·7%)	5,603	2,737	48·8%	(47·5%, 50·2%)
**Stage**	**Localised**	1,553	470	30·3%	(28·0%, 32·6%)	768	319	41·5%	(38·1%, 45·1%)	3,209	775	24·2%	(22·7%, 25·7%)	391	56	14·3%	(11·2%, 18·1%)	14,325	3,038	21·2%	(20·5%, 21·9%)
	**Inv. adjc. tissue/organ**	238	103	43·3%	(37·1%, 49·6%)	448	211	47·1%	(42·5%, 51·7%)	939	247	26·3%	(23·6%, 29·2%)	228	44	19·3%	(14·7%, 24·9%)	6,262	1,683	26·9%	(25·8%, 28·0%)
	**Inv. regional LNs**	100	34	34·0%	(25·5%, 43·7%)	480	185	38·5%	(34·3%, 43·0%)	2,472	571	23·1%	(21·5%, 24·8%)	62	7	11·3%	(5·6%, 21·5%)	8,854	2,088	23·6%	(22·7%, 24·5%)
	**Distant metastasis**	556	299	53·8%	(49·6%, 57·9%)	2,150	1,062	49·4%	(47·3%, 51·5%)	7,435	3,772	50·7%	(49·6%, 51·9%)	1449	498	34·4%	(32·0%, 36·9%)	17,181	8,023	46·7%	(46·0%, 47·4%)
	**Unknown**	708	255	36·0%	(32·6%, 39·6%)	652	259	39·7%	(36·0%, 43·5%)	2,279	576	25·3%	(23·5%, 27·1%)	157	44	28·0%	(21·6%, 35·5%)	6,576	1,613	24·5%	(23·5%, 25·6%)

Victoria		Oesophageal				Stomach				Colon				Rectal			
		All	EP	%EP	95% CI	All	EP	%EP	95% CI	All	EP	%EP	95% CI	All	EP	%EP	95% CI
	**Total**	1,576	315	20·0%	(18·1%, 22·0%)	2,597	616	23·7%	(22·1%, 25·4%)	11,747	2,689	22·9%	(22·1%, 23·7%)	4,438	403	9·1%	(8·3%, 10·0%)
**Sex**	**Male**	1,080	210	19·4%	(17·2%, 21·9%)	1,701	391	23·0%	(21·0%, 25·0%)	5,924	1,305	22·0%	(21·0%, 23·1%)	2,760	257	9·3%	(8·3%, 10·5%)
	**Female**	496	105	21·2%	(17·8%, 25·0%)	896	225	25·1%	(22·4%, 28·1%)	5,823	1,384	23·8%	(22·7%, 24·9%)	1,678	146	8·7%	(7·4%, 10·1%)
**Diagnosis**	**2012**	298	61	20·5%	(16·3%, 25·4%)	522	126	24·1%	(20·7%, 28·0%)	2,249	486	21·6%	(20·0%, 23·4%)	876	87	9·9%	(8·1%, 12·1%)
**year**	**2013**	336	60	17·9%	(14·1%, 22·3%)	508	126	24·8%	(21·2%, 28·7%)	2,279	496	21·8%	(20·1%, 23·5%)	886	86	9·7%	(7·9%, 11·8%)
	**2014**	312	54	17·3%	(13·5%, 21·9%)	504	110	21·8%	(18·4%, 25·6%)	2,349	561	23·9%	(22·2%, 25·6%)	837	68	8·1%	(6·5%, 10·2%)
	**2015**	311	65	20·9%	(16·8%, 25·8%)	528	127	24·1%	(20·6%, 27·9%)	2,449	599	24·5%	(22·8%, 26·2%)	900	74	8·2%	(6·6%, 10·2%)
	**2016**	319	75	23·5%	(19·2%, 28·5%)	535	127	23·7%	(20·3%, 27·5%)	2,421	547	22·6%	(21·0%, 24·3%)	939	88	9·4%	(7·7%, 11·4%)
**Age group**	**15-64**	465	71	15·3%	(12·3%, 18·8%)	866	152	17·6%	(15·2%, 20·2%)	3,647	855	23·4%	(22·1%, 24·8%)	1,962	137	7·0%	(5·9%, 8·2%)
	**65-74**	477	83	17·4%	(14·3%, 21·1%)	702	148	21·1%	(18·2%, 24·3%)	3,152	517	16·4%	(15·2%, 17·7%)	1,207	94	7·8%	(6·4%, 9·4%)
	**75-84**	421	92	21·9%	(18·2%, 26·0%)	705	186	26·4%	(23·3%, 29·8%)	3,346	770	23·0%	(21·6%, 24·5%)	895	100	11·2%	(9·3%, 13·4%)
	**85+**	213	69	32·4%	(26·5%, 38·9%)	324	130	40·1%	(34·9%, 45·5%)	1,602	547	34·1%	(31·9%, 36·5%)	374	72	19·3%	(15·6%, 23·6%)
**Stage***	**Localised**	…	…	…	…	…	…	…	…	2,368	156	6·6%	(5·7%, 7·7%)	1,219	37	3·0%	(2·2%, 4·2%)
	**Inv. adjc. tissue/organ**	…	…	…	…	…	…	…	…	3,254	644	19·8%	(18·5%, 21·2%)	757	66	8·7%	(6·9%, 10·9%)
	**Inv. regional LNs**	…	…	…	…	…	…	…	…	2,811	637	22·7%	(21·2%, 24·2%)	897	67	7·5%	(5·9%, 9·4%)
	**Distant metastasis**	…	…	…	…	…	…	…	…	2,111	766	36·3%	(34·3%, 38·4%)	616	137	22·2%	(19·1%, 25·7%)
	**Unknown**	1,576	315	20·0%	(18·1%, 22·0%)	2,597	616	23·7%	(22·1%, 25·4%)	1,185	484	40·8%	(38·1%, 43·7%)	934	94	10·1%	(8·3%, 12·2%)

		Liver				Pancreatic				Lung				Ovarian				All cancers			
		All	EP	%EP	95% CI	All	EP	%EP	95% CI	All	EP	%EP	95% CI	All	EP	%EP	95% CI	All	EP	%EP	95% CI
	**Total**	2,323	742	31·9%	(30·1%, 33·9%)	3,172	1,083	34·1%	(32·5%, 35·8%)	10,620	2,954	27·8%	(27·0%, 28·7%)	1,739	363	20·9%	(19·0%, 22·8%)	38,212	9,165	24·0%	(23·6%, 24·4%)
**Sex**	**Male**	1,669	517	31·0%	(28·8%, 33·2%)	1,619	564	34·8%	(32·6%, 37·2%)	5,967	1,736	29·1%	(28·0%, 30·3%)					20,720	4,980	24·0%	(23·5%, 24·6%)
	**Female**	654	225	34·4%	(30·9%, 38·1%)	1,553	519	33·4%	(31·1%, 35·8%)	4,653	1,218	26·2%	(24·9%, 27·5%)	1,739	363	20·9%	(19·0%, 22·8%)	17,492	4,185	23·9%	(23·3%, 24·6%)
**Diagnosis**	**2012**	363	130	35·8%	(31·1%, 40·9%)	530	167	31·5%	(27·7%, 35·6%)	1,872	490	26·2%	(24·2%, 28·2%)	335	68	20·3%	(16·3%, 24·9%)	7,045	1,615	22·9%	(22·0%, 23·9%)
**year**	**2013**	453	127	28.0%	(24·1%, 32·3%)	539	177	32·8%	(29·0%, 36·9%)	1,895	491	25·9%	(24·0%, 27·9%)	336	75	22·3%	(18·2%, 27·1%)	7,232	1,638	22·6%	(21·7%, 23·6%)
	**2014**	483	149	30·8%	(26·9%, 35·1%)	644	213	33·1%	(29·6%, 36·8%)	2,170	605	27·9%	(26·0%, 29·8%)	347	72	20·7%	(16·8%, 25·3%)	7,646	1,832	24·0%	(23·0%, 24·9%)
	**2015**	506	163	32·2%	(28·3%, 36·4%)	706	238	33·7%	(30·3%, 37·3%)	2,229	669	30.0%	(28·1%, 31·9%)	332	63	19.0%	(15·1%, 23·5%)	7,961	1,998	25·1%	(24·2%, 26·1%)
	**2016**	518	173	33·4%	(29·5%, 37·6%)	753	288	38·2%	(34·8%, 41·8%)	2,454	699	28·5%	(26·7%, 30·3%)	389	85	21·9%	(18·0%, 26·2%)	8,328	2,082	25·0%	(24·1%, 25·9%)
**Age group**	**15-64**	1,003	280	27·9%	(25·2%, 30·8%)	882	256	29·0%	(26·1%, 32·1%)	2,851	721	25·3%	(23·7%, 26·9%)	892	146	16·4%	(14·1%, 18·9%)	12,568	2,618	20·8%	(20·1%, 21·5%)
	**65-74**	582	178	30·6%	(27·0%, 34·4%)	927	263	28·4%	(25·6%, 31·4%)	3,527	871	24·7%	(23·3%, 26·1%)	442	72	16·3%	(13·1%, 20·0%)	11,016	2,226	20·2%	(19·5%, 21·0%)
	**75-84**	550	182	33·1%	(29·3%, 37·1%)	865	321	37·1%	(34·0%, 40·4%)	3,111	911	29·3%	(27·7%, 30·9%)	295	91	30·8%	(25·9%, 36·3%)	10,188	2,653	26·0%	(25·2%, 26·9%)
	**85+**	188	102	54·3%	(47·1%, 61·2%)	498	243	48·8%	(44·4%, 53·2%)	1,131	451	39·9%	(37·1%, 42·8%)	110	54	49·1%	(39·9%, 58·3%)	4,440	1,668	37·6%	(36·2%, 39·0%)
**Stage***	**Localised**	…	…	…	…	…	…	…	…	…	…	…	…	…	…	…	…	3,587	193	5·4%	(4·7%, 6·2%)
	**Inv. adjc. tissue/organ**	…	…	…	…	…	…	…	…	…	…	…	…	…	…	…	…	4,011	710	17·7%	(16·6%, 18·9%)
	**Inv. regional LNs**	…	…	…	…	…	…	…	…	…	…	…	…	…	…	…	…	3,708	704	19·0%	(17·8%, 20·3%)
	**Distant metastasis**	…	…	…	…	…	…	…	…	…	…	…	…	…	…	…	…	2,727	903	33·1%	(31·4%, 34·9%)
	**Unknown**	2,323	742	31·9%	(30·1%, 33·9%)	3,172	1,083	34·1%	(32·5%, 35·8%)	10,620	2,954	27·8%	(27·0%, 28·7%)	1,739	363	20·9%	(19%, 22·8%)	24,146	6,651	27·5%	(27·0%, 28·1%)

New Zealand		Oesophageal				Stomach				Colon				Rectal			
		All	EP	%EP	95% CI	All	EP	%EP	95% CI	All	EP	%EP	95% CI	All	EP	%EP	95% CI
	Total	1,196	440	36·8%	(34·1%, 39·6%)	1,520	726	47·8%	(45·3%, 50·3%)	9,502	3,480	36·6%	(35·7%, 37·6%)	3,247	643	19·8%	(18·5%, 21·2%)
Sex	Male	834	307	36·8%	(33·6%, 40·1%)	949	442	46·6%	(43·4%, 49·8%)	4,629	1,662	35·9%	(34·5%, 37·3%)	2,034	394	19·4%	(17·7%, 21·1%)
	Female	362	133	36·7%	(31·9%, 41·8%)	571	284	49·7%	(45·7%, 53·8%)	4,873	1,818	37·3%	(36·0%, 38·7%)	1,213	249	20·5%	(18·3%, 22·9%)
Diagnosis year	2012	305	107	35·1%	(29·9%, 40·6%)	377	170	45·1%	(40·1%, 50·1%)	2,312	832	36·0%	(34·1%, 38·0%)	786	159	20·2%	(17·6%, 23·2%)
	2013	312	120	38·5%	(33·2%, 44·0%)	370	184	49·7%	(44·7%, 54·8%)	2,363	853	36·1%	(34·2%, 38·1%)	786	167	21·2%	(18·5%, 24·2%)
	2014	273	100	36·6%	(31·1%, 42·5%)	391	193	49·4%	(44·4%, 54·3%)	2,452	889	36·3%	(34·4%, 38·2%)	876	155	17·7%	(15·3%, 20·4%)
	2015	306	113	36·9%	(31·7%, 42·5%)	382	179	46·9%	(41·9%, 51·9%)	2,375	906	38·1%	(36·2%, 40·1%)	799	162	20·3%	(17·6%, 23·2%)
	2016																
Age group	15-64	328	100	30·5%	(25·8%, 35·7%)	562	250	44·5%	(40·4%, 48·6%)	2,314	882	38·1%	(36·2%, 40·1%)	1,229	212	17·3%	(15·2%, 19·5%)
	65-74	372	121	32·5%	(28·0%, 37·4%)	413	173	41·9%	(37·2%, 46·7%)	2,776	878	31·6%	(29·9%, 33·4%)	996	158	15·9%	(13·7%, 18·3%)
	75-84	325	133	40·9%	(35·7%, 46·3%)	381	203	53·3%	(48·3%, 58·2%)	3,089	1,095	35·5%	(33·8%, 37·2%)	734	165	22·5%	(19·6%, 25·6%)
	85+	171	86	50·3%	(42·9%, 57·7%)	164	100	61·0%	(53·3%, 68·1%)	1,323	625	47·2%	(44·6%, 49·9%)	288	108	37·5%	(32·1%, 43·2%)
Stage	Localised	29	5	17·2%	(7·6%, 34·5%)	127	28	22·0%	(15·7%, 30·0%)	2,193	417	19.0%	(17·4%, 20·7%)	588	48	8·2%	(6·2%, 10·7%)
	Inv. adjc. tissue/organ	6	1	16·7%	(3·0%, 56·4%)	30	15	50·0%	(33·2%, 66·8%)	1,565	540	34·5%	(32·2%, 36·9%)	208	42	20·2%	(15·3%, 26·2%)
	Inv. regional LNs	84	30	35·7%	(26·3%, 46·4%)	141	60	42·6%	(34·7%, 50·8%)	2,348	846	36.0%	(34·1%, 38·0%)	526	89	16·9%	(14·0%, 20·4%)
	Distant metastasis	313	150	47·9%	(42·4%, 53·4%)	533	338	63·4%	(59·2%, 67·4%)	2,199	1,269	57·7%	(55·6%, 59·8%)	484	210	43·4%	(39·0%, 47·8%)
	Unknown	764	254	33·2%	(30·0%, 36·7%)	689	285	41·4%	(37·7%, 45·1%)	1,197	408	34·1%	(31·5%, 36·8%)	1,441	254	17·6%	(15·7%, 19·7%)

		Liver				Pancreatic				Lung				Ovarian				All cancers			
		All	EP	%EP	95% CI	All	EP	%EP	95% CI	All	EP	%EP	95% CI	All	EP	%EP	95% CI	All	EP	%EP	95% CI
	Total	1,243	618	49·7%	(46·9%, 52·5%)	2,182	1,317	60·4%	(58·3%, 62·4%)	8,481	4,330	51·1%	(50·0%, 52·1%)	1,423	684	48·1%	(45·5%, 50·7%)	28,794	12,238	42·5%	(41·9%, 43·1%)
Sex	Male	876	417	47·6%	(44·3%, 50·9%)	1,091	679	62·2%	(59·3%, 65·1%)	4,338	2,263	52·2%	(50·7%, 53·7%)					14,751	6,164	41·8%	(41·0%, 42·6%)
	Female	367	201	54·8%	(49·7%, 59·8%)	1,091	638	58·5%	(55·5%, 61·4%)	4,143	2,067	49·9%	(48·4%, 51·4%)	1,423	684	48·1%	(45·5%, 50·7%)	14,043	6,074	43·3%	(42·4%, 44·1%)
Diagnosis year	2012	293	130	44·4%	(38·8%, 50·1%)	538	327	60·8%	(56·6%, 64·8%)	2,033	1,035	50·9%	(48·7%, 53·1%)	317	157	49·5%	(44·1%, 55·0%)	6,961	2,917	41·9%	(40·8%, 43·1%)
	2013	273	140	51·3%	(45·4%, 57·2%)	505	305	60·4%	(56·1%, 64·6%)	2,025	1,066	52·6%	(50·5%, 54·8%)	360	174	48·3%	(43·2%, 53·5%)	6,994	3,009	43·0%	(41·9%, 44·2%)
	2014	332	163	49·1%	(43·8%, 54·5%)	555	333	60·0%	(55·9%, 64·0%)	2,216	1,123	50·7%	(48·6%, 52·8%)	352	166	47·2%	(42·0%, 52·4%)	7,447	3,122	41·9%	(40·8%, 43·0%)
	2015	345	185	53·6%	(48·3%, 58·8%)	584	352	60·3%	(56·3%, 64·2%)	2,207	1,106	50·1%	(48·0%, 52·2%)	394	187	47·5%	(42·6%, 52·4%)	7,392	3,190	43·2%	(42·0%, 44·3%)
	2016																				
Age group	15-64	575	268	46·6%	(42·6%, 50·7%)	625	351	56·2%	(52·2%, 60·0%)	2,528	1,275	50·4%	(48·5%, 52·4%)	658	284	43·2%	(39·4%, 47·0%)	8,819	3,622	41·1%	(40·0%, 42·1%)
	65-74	331	154	46·5%	(41·2%, 51·9%)	637	396	62·2%	(58·3%, 65·8%)	2,890	1,383	47·9%	(46·0%, 49·7%)	370	189	51·1%	(46·0%, 56·1%)	8,785	3,452	39·3%	(38·3%, 40·3%)
	75-84	242	139	57·4%	(51·1%, 63·5%)	595	374	62·9%	(58·9%, 66·6%)	2,265	1,186	52·4%	(50·3%, 54·4%)	256	131	51·2%	(45·1%, 57·2%)	7,887	3,426	43·4%	(42·3%, 44·5%)
	85+	95	57	60·0%	(49·9%, 69·3%)	325	196	60·3%	(54·9%, 65·5%)	798	486	60·9%	(57·5%, 64·2%)	139	80	57·6%	(49·2%, 65·5%)	3,303	1,738	52·6%	(50·9%, 54·3%)
Stage	Localised	133	38	28·6%	(21·6%, 36·8%)	62	11	17·7%	(10·2%, 29·0%)	577	73	12·7%	(10·2%, 15·6%)	184	41	22·3%	(16·9%, 28·8%)	3,893	661	17·0%	(15·8%, 18·2%)
	Inv. adjc. tissue/organ	22	13	59·1%	(38·7%, 76·7%)	45	24	53·3%	(39·1%, 67·1%)	337	91	27·0%	(22·5%, 32·0%)	208	67	32·2%	(26·2%, 38·8%)	2,421	793	32·8%	(30·9%, 34·7%)
	Inv. regional LNs	25	23	92·0%	(75·0%, 97·8%)	138	78	56·5%	(48·2%, 64·5%)	922	411	44·6%	(41·4%, 47·8%)	29	8	27·6%	(14·7%, 45·7%)	4,213	1,545	36·7%	(35·2%, 38·1%)
	Distant metastasis	298	212	71·1%	(65·8%, 76·0%)	1,188	786	66·2%	(63·4%, 68·8%)	3,857	2,631	68·2%	(66·7%, 69·7%)	894	534	59·7%	(56·5%, 62·9%)	9,766	6,130	62·8%	(61·8%, 63·7%)
	Unknown	765	332	43·4%	(39·9%, 46·9%)	749	418	55·8%	(52·2%, 59·3%)	2,788	1,124	40·3%	(38·5%, 42·1%)	108	34	31·5%	(23·5%, 40·7%)	8,501	3,109	36·6%	(35·6%, 37·6%)

All: All patients; EP: Emegency presentations; CI: Confidence Interval; Inv: Invading; Adjc: Adjacent; LNs: Lymph Nodes.

* Data shown for stage categories in Victoria does not include 33 cases in groups other than shown.

$ For Atlantic Canada, British Columbia, and Saskatchewan-Manitoba, random rounding within cells to a multiple of 5 was used for the tabulated variable groups, to adhere to rules for minimising residual disclosure risk.

Supplementary Material 7. Associations between emergency presentation (defined as diagnosis of cancer within 30 days after an emergency hospital admission) and sex, age, diagnosis year, stage at diagnosis, and cancer site, by jurisdiction, and odds ratios from crude and adjusted models (including adjustment for stage at diagnosis).

Odds ratios from adjusted models not including stage at diagnosis shown in Supplementary Material 8.

Denmark		All	EP	%EP	95% CI	Crude OR	Crude OR (LCI)	Crude OR (UCI)	p(COR)	Adjusted OR	Adj OR (LCI)	Adj OR (UCI)	p(AOR)
	**Total**	46,003	14,199	30·9%	(30·4%, 31·3%)								
**Sex**	**Male**	24,016	7,334	30·5%	(30·0%, 31·1%)	(ref)	(ref)	(ref)	0·11	(ref)	(ref)	(ref)	0·98
	**Female**	21,987	6,865	31·2%	(30·6%, 31·8%)	1·03	0·99	1·07		1·01	0·97	1·05	
**Diagnosis year**	**2014**	11,354	3,750	33·0%	(32·2%, 33·9%)	(ref)	(ref)	(ref)	<0·0001	(ref)	(ref)	(ref)	<0·0001
	**2015**	11,423	3,535	30·9%	(30·1%, 31·8%)	0·91	0·86	0·96		0·92	0·87	0·98	
	**2016**	11,466	3,549	31·0%	(30·1%, 31·8%)	0·91	0·86	0·96		0·92	0·86	0·97	
	**2017**	11,760	3,365	28·6%	(27·8%, 29·4%)	0·81	0·77	0·86		0·82	0·77	0·87	
**Age group**	**15-64**	13,131	3,468	26·4%	(25·7%, 27·2%)	0·94	0·89	0·99	<0·0001	0·97	0·92	1·02	<0·0001
	**65-74**	17,336	4,785	27·6%	(26·9%, 28·3%)	(ref)	(ref)	(ref)		(ref)	(ref)	(ref)	
	**75-84**	11,995	4,252	35·4%	(34·6%, 36·3%)	1·44	1·37	1·51		1·44	1·37	1·52	
	**85+**	3,541	1,694	47·8%	(46·2%, 49·5%)	2·41	2·23	2·59		2·46	2·27	2·66	
**Stage**	**1**	5,977	891	14·9%	(14·0%, 15·8%)	(ref)	(ref)	(ref)	<0·0001	(ref)	(ref)	(ref)	<0·0001
	**2**	6,064	1,329	21·9%	(20·9%, 23·0%)	1·60	1·46	1·76		1·46	1·33	1·61	
	**3**	7,231	1,678	23·2%	(22·2%, 24·2%)	1·72	1·58	1·89		1·64	1·50	1·80	
	**4**	15,359	6,630	43·2%	(42·4%, 44·0%)	4·34	4·01	4·69		3·65	3·37	3·96	
	**Unknown**	11,372	3,671	32·3%	(31·4%, 33·1%)	2·72	2·51	2·95		2·72	2·50	2·96	
**Cancer site**	**Oesophageal**	1,896	461	24·3%	(22·4%, 26·3%)	0·82	0·73	0·91	<0·0001	0·75	0·67	0·84	<0·0001
	**Stomach**	2,231	719	32·2%	(30·3%, 34·2%)	1·21	1·09	1·33		1·11	1·01	1·23	
	**Colon**	12,928	3,655	28·3%	(27·5%, 29·1%)	(ref)	(ref)	(ref)		(ref)	(ref)	(ref)	
	**Rectal**	6,005	811	13·5%	(12·7%, 14·4%)	0·40	0·36	0·43		0·40	0·37	0·44	
	**Liver**	1,541	668	43·3%	(40·9%, 45·8%)	1·94	1·74	2·16		1·92	1·72	2·15	
	**Pancreatic**	3,491	1,674	48·0%	(46·3%, 49·6%)	2·34	2·16	2·52		1·94	1·79	2·10	
	**Lung**	16,003	5,818	36·4%	(35·6%, 37·1%)	1·45	1·38	1·52		1·34	1·27	1·42	
	**Ovarian**	1,908	393	20·6%	(18·8%, 22·5%)	0·66	0·58	0·74		0·61	0·54	0·68	

Norway		All	EP	%EP	95% CI	Crude OR	Crude OR (LCI)	Crude OR (UCI)	p(COR)	Adjusted OR	Adj OR (LCI)	Adj OR (UCI)	p(AOR)
	**Total**	46,147	16,866	36·5%	(36·1%, 37·0%)								
**Sex**	**Male**	23,319	8,410	36·1%	(35·5%, 36·7%)	(ref)	(ref)	(ref)	0·029	(ref)	(ref)	(ref)	0·51
	**Female**	22,828	8,456	37·0%	(36·4%, 37·7%)	1·04	1.00	1·08		1·01	0·97	1·06	
**Diagnosis year**	**2012**	8,962	3,508	39·1%	(38·1%, 40·2%)	(ref)	(ref)	(ref)	<0·0001	(ref)	(ref)	(ref)	<0·0001
	**2013**	9,060	3,350	37·0%	(36·0%, 38·0%)	0·91	0·86	0·97		0·92	0·86	0·98	
	**2014**	9,293	3,435	37·0%	(36·0%, 37·9%)	0·91	0·86	0·97		0·93	0·87	0·99	
	**2015**	9,561	3,445	36·0%	(35·1%, 37·0%)	0·88	0·83	0·93		0·88	0·83	0·94	
	**2016**	9,271	3,128	33·7%	(32·8%, 34·7%)	0·79	0·75	0·84		0·80	0·75	0·86	
**Age group**	**15-64**	13,277	4,165	31·4%	(30·6%, 32·2%)	0·89	0·85	0·94	<0·0001	0·91	0·86	0·96	<0·0001
	**65-74**	15,278	5,172	33·9%	(33·1%, 34·6%)	(ref)	(ref)	(ref)		(ref)	(ref)	(ref)	
	**75-84**	12,424	4,838	38·9%	(38·1%, 39·8%)	1·25	1·19	1·31		1·30	1·23	1·37	
	**85+**	5,168	2,691	52·1%	(50·7%, 53·4%)	2·12	1·99	2·26		2·28	2·13	2·44	
**Stage**	**Local**	8,515	1,487	17·5%	(16·7%, 18·3%)	(ref)	(ref)	(ref)	<0·0001	(ref)	(ref)	(ref)	<0·0001
	**Regional**	16,460	4,942	30·0%	(29·3%, 30·7%)	2·03	1·90	2·16		2·04	1·91	2·18	
	**Distant**	15,613	8,140	52·1%	(51·4%, 52·9%)	5·15	4·83	5·49		5·04	4·72	5·39	
	**Unknown**	5,559	2,297	41·3%	(40·0%, 42·6%)	3·33	3·08	3·60		2·67	2·46	2·90	
**Cancer site**	**Oesophageal**	1,332	435	32·7%	(30·2%, 35·2%)	0·87	0·77	0·98	<0·0001	0·82	0·73	0·93	<0·0001
	**Stomach**	2,357	943	40·0%	(38·0%, 42·0%)	1·19	1·09	1·31		1·09	0·99	1·20	
	**Colon**	14,941	5,354	35·8%	(35·1%, 36·6%)	(ref)	(ref)	(ref)		(ref)	(ref)	(ref)	
	**Rectal**	5,612	888	15·8%	(14·9%, 16·8%)	0·34	0·31	0·36		0·37	0·34	0·40	
	**Liver**	1,181	597	50·6%	(47·7%, 53·4%)	1·83	1·63	2·06		2·11	1·85	2·39	
	**Pancreatic**	3,715	2,059	55·4%	(53·8%, 57·0%)	2·23	2·07	2·39		1·70	1·57	1·83	
	**Lung**	14,402	5,679	39·4%	(38·6%, 40·2%)	1·17	1·11	1·22		1·03	0·98	1·08	
	**Ovarian**	2,607	911	34·9%	(33·1%, 36·8%)	0·96	0·88	1·05		0·68	0·61	0·75	

England		All	EP	%EP	95% CI	Crude OR	Crude OR (LCI)	Crude OR (UCI)	p(COR)	Adjusted OR	Adj OR (LCI)	Adj OR (UCI)	p(AOR)
	**Total**	373,194	116,934	31·3%	(31·2%, 31·5%)								
**Sex**	**Male**	197,003	57,839	29·4%	(29·2%, 29·6%)	(ref)	(ref)	(ref)	<0·0001	(ref)	(ref)	(ref)	<0·0001
	**Female**	176,191	59,095	33·5%	(33·3%, 33·8%)	1·21	1·20	1·23		1·10	1·09	1·12	
**Diagnosis year**	**2012**	-	-										
	**2013**	90,993	29,271	32·2%	(31·9%, 32·5%)	(ref)	(ref)	(ref)	<0·0001	(ref)	(ref)	(ref)	0.0021
	**2014**	92,609	29,315	31·7%	(31·4%, 32·0%)	0·98	0·96	1.00		0·99	0·97	1·01	
	**2015**	94,296	29,297	31·1%	(30·8%, 31·4%)	0·95	0·93	0·97		0·98	0·96	1.00	
	**2016**	95,296	29,051	30·5%	(30·2%, 30·8%)	0·92	0·91	0·94		0·96	0·94	0·98	
**Age group**	**15-64**	98,419	24,794	25·2%	(24·9%, 25·5%)	0·97	0·95	0·99	<0·0001	1.00	0·98	1·02	<0·0001
	**65-74**	112,970	29,192	25·8%	(25·6%, 26·1%)	(ref)	(ref)	(ref)		(ref)	(ref)	(ref)	
	**75-84**	110,521	37,552	34·0%	(33·7%, 34·3%)	1·48	1·45	1·50		1·48	1·45	1·51	
	**85+**	51,284	25,396	49·5%	(49·1%, 50·0%)	2·82	2·75	2·88		2·72	2·66	2·79	
**Stage**	**1**	48,445	6,025	12·4%	(12·1%, 12·7%)	(ref)	(ref)	(ref)	<0·0001	(ref)	(ref)	(ref)	<0·0001
	**2**	49,375	9,928	20·1%	(19·8%, 20·5%)	1·77	1·71	1·83		1·75	1·69	1·82	
	**3**	80,853	17,432	21·6%	(21·3%, 21·8%)	1·94	1·87	2.00		2·02	1·96	2·09	
	**4**	136,382	58,525	42·9%	(42·7%, 43·2%)	5·29	5·14	5·45		4·94	4·80	5·09	
	**Unknown**	58,139	25,024	43·0%	(42·6%, 43·4%)	5·32	5·16	5·49		4·22	4·08	4·36	
**Cancer site**	**Oesophageal**	27,274	5,364	19·7%	(19·2%, 20·1%)	0·59	0·57	0·61	<0·0001	0·53	0·51	0·55	<0·0001
	**Stomach**	19,813	6,224	31·4%	(30·8%, 32·1%)	1·10	1·07	1·14		0·87	0·84	0·90	
	**Colon**	88,219	25,859	29·3%	(29·0%, 29·6%)	(ref)	(ref)	(ref)		(ref)	(ref)	(ref)	
	**Rectal**	33,905	3,628	10·7%	(10·4%, 11·0%)	0·29	0·28	0·30		0·33	0·32	0·34	
	**Liver**	17,699	7,497	42·4%	(41·6%, 43·1%)	1·77	1·71	1·83		1·29	1·25	1·34	
	**Pancreatic**	30,442	14,291	46·9%	(46·4%, 47·5%)	2·13	2·08	2·19		1·53	1·49	1·57	
	**Lung**	135,396	46,951	34·7%	(34·4%, 34·9%)	1·28	1·26	1·30		1·08	1·06	1·10	
	**Ovarian**	20,446	7,120	34·8%	(34·2%, 35·5%)	1·29	1·25	1·33		1·38	1·33	1·43	

Northern Ireland		All	EP	%EP	95% CI	Crude OR	Crude OR (LCI)	Crude OR (UCI)	p(COR)	Adjusted OR	Adj OR (LCI)	Adj OR (UCI)	p(AOR)
	**Total**	15,501	4,322	27·9%	(27·2%, 28·6%)								
**Sex**	**Male**	8,134	2,124	26·1%	(25·2%, 27·1%)	(ref)	(ref)	(ref)	<0·0001	(ref)	(ref)	(ref)	0·0030
	**Female**	7,367	2,198	29·8%	(28·8%, 30·9%)	1·20	1·12	1·29		1·13	1·04	1·22	
**Diagnosis year**	**2012**	3,071	909	29·6%	(28·0%, 31·2%)	1·09	0·98	1·22	0·14	1·06	0·94	1·19	0·41
	**2013**	3,125	867	27·7%	(26·2%, 29·3%)	(ref)	(ref)	(ref)		(ref)	(ref)	(ref)	
	**2014**	3,051	856	28·1%	(26·5%, 29·7%)	1·02	0·91	1·14		1·02	0·91	1·15	
	**2015**	3,072	835	27·2%	(25·6%, 28·8%)	0·97	0·87	1·09		0·95	0·84	1·07	
	**2016**	3,182	855	26·9%	(25·4%, 28·4%)	0·96	0·86	1·07		0·97	0·86	1·09	
**Age group**	**15-64**	4,432	1,003	22·6%	(21·4%, 23·9%)	0·90	0·82	0·99	<0·0001	0·94	0·85	1·04	<0·0001
	**65-74**	4,965	1,220	24·6%	(23·4%, 25·8%)	(ref)	(ref)	(ref)		(ref)	(ref)	(ref)	
	**75-84**	4,408	1,351	30·6%	(29·3%, 32·0%)	1·36	1·24	1·49		1·32	1·20	1·46	
	**85+**	1,696	748	44·1%	(41·8%, 46·5%)	2·42	2·16	2·72		2·21	1·94	2·51	
**Stage**	**1**	2,221	172	7·7%	(6·7%, 8·9%)	(ref)	(ref)	(ref)	<0·0001	(ref)	(ref)	(ref)	<0·0001
	**2**	2,234	295	13·2%	(11·9%, 14·7%)	1·81	1·49	2·21		1·87	1·53	2·29	
	**3**	3,584	734	20·5%	(19·2%, 21·8%)	3·07	2·58	3·67		3·17	2·66	3·79	
	**4**	5,184	2,175	42·0%	(40·6%, 43·3%)	8·61	7·32	10·19		8·32	7·06	9·87	
	**Unknown**	2,278	946	41·5%	(39·5%, 43·6%)	8·46	7·11	10·12		6·88	5·73	8·29	
**Cancer site**	**Oesophageal**	972	193	19·9%	(17·5%, 22·5%)	0·79	0·67	0·94	<0·0001	0·56	0·47	0·68	<0·0001
	**Stomach**	990	293	29·6%	(26·8%, 32·5%)	1·35	1·15	1·57		0·85	0·72	1.00	
	**Colon**	3,846	914	23·8%	(22·4%, 25·1%)	(ref)	(ref)	(ref)		(ref)	(ref)	(ref)	
	**Rectal**	1,376	125	9·1%	(7·7%, 10·7%)	0·32	0·26	0·39		0·36	0·29	0·44	
	**Liver**	582	215	36·9%	(33·1%, 40·9%)	1·88	1·56	2·26		1·36	1·12	1·67	
	**Pancreatic**	1,162	496	42·7%	(39·9%, 45·5%)	2·39	2·08	2·74		1·50	1·29	1·74	
	**Lung**	5,752	1,843	32·0%	(30·8%, 33·3%)	1·51	1·38	1·66		1·17	1·06	1·30	
	**Ovarian**	821	243	29·6%	(26·6%, 32·8%)	1·35	1·14	1·59		1·40	1·16	1·68	

Scotland		All	EP	%EP	95% CI	Crude OR	Crude OR (LCI)	Crude OR (UCI)	p(COR)	Adjusted OR	Adj OR (LCI)	Adj OR (UCI)	p(AOR)
	**Total**	45,029	17,321	38·5%	(38·0%, 38·9%)								
**Sex**	**Male**	23,042	8,506	36·9%	(36·3%, 37·5%)	0·87	0·84	0·91	<0·0001	0·96	0·92	1.00	0·081
	**Female**	21,987	8,815	40·1%	(39·4%, 40·7%)	(ref)	(ref)	(ref)		(ref)	(ref)	(ref)	
**Diagnosis year**	**2014**	11,599	4,511	38·9%	(38·0%, 39·8%)	(ref)	(ref)	(ref)	0.048	(ref)	(ref)	(ref)	0.18
	**2015**	11,091	4,346	39·2%	(38·3%, 40·1%)	1·01	0·96	1·07		1·01	0·96	1·07	
	**2016**	11,064	4,236	38·3%	(37·4%, 39·2%)	0·97	0·92	1·03		0·98	0·93	1·04	
	**2017**	11,275	4,228	37·5%	(36·6%, 38·4%)	0·94	0·89	0·99		0·95	0·9	1·01	
**Age group**	**15-64**	12,024	3,920	32·6%	(31·8%, 33·4%)	0·95	0·90	1.00	<0·0001	0·99	0·94	1·05	<0·0001
	**65-74**	14,233	4,817	33·8%	(33·1%, 34·6%)	(ref)	(ref)	(ref)		(ref)	(ref)	(ref)	
	**75-84**	13,399	5,580	41·6%	(40·8%, 42·5%)	1·39	1·33	1·46		1·39	1·32	1·47	
	**85+**	5,373	3,004	55·9%	(54·6%, 57·2%)	2·48	2·32	2·64		2·38	2·23	2·55	
**Stage**	**1**	5,858	1,216	20·8%	(19·7%, 21·8%)	(ref)	(ref)	(ref)	<0·0001	(ref)	(ref)	(ref)	<0·0001
	**2**	5,857	1,534	26·2%	(25·1%, 27·3%)	1·35	1·24	1·48		1·43	1·31	1·56	
	**3**	9,180	2,736	29·8%	(28·9%, 30·7%)	1·62	1·50	1·75		1·71	1·58	1·85	
	**4**	17,250	8,585	49·8%	(49·0%, 50·5%)	3·78	3·53	4·06		3·52	3·28	3·79	
	**Unknown**	6,884	3,250	47·2%	(46·0%, 48·4%)	3·41	3·16	3·70		2·98	2·74	3·24	
**Cancer site**	**Oesophageal**	3,378	851	25·2%	(23·8%, 26·7%)	0·62	0·57	0·68	<0·0001	0·54	0·49	0·59	<0·0001
	**Stomach**	2,337	913	39·1%	(37·1%, 41·1%)	1·19	1·08	1·30		0·92	0·83	1·01	
	**Colon**	9,600	3,366	35·1%	(34·1%, 36·0%)	(ref)	(ref)	(ref)		(ref)	(ref)	(ref)	
	**Rectal**	3,535	499	14·1%	(13·0%, 15·3%)	0·30	0·27	0·34		0·34	0·30	0·37	
	**Liver**	2,340	1,111	47·5%	(45·5%, 49·5%)	1·67	1·53	1·83		1·44	1·31	1·59	
	**Pancreatic**	3,054	1,809	59·2%	(57·5%, 61·0%)	2·69	2·48	2·93		2·14	1·96	2·34	
	**Lung**	18,662	7,863	42·1%	(41·4%, 42·8%)	1·35	1·28	1·42		1·20	1·13	1·26	
	**Ovarian**	2,123	909	42·8%	(40·7%, 44·9%)	1·39	1·26	1·53		1·47	1·33	1·63	

Wales		All	EP	%EP	95% CI	Crude OR	Crude OR (LCI)	Crude OR (UCI)	p(COR)	Adjusted OR	Adj OR (LCI)	Adj OR (UCI)	p(AOR)
	**Total**	26,139	9,763	37·4%	(36·8%, 37·9%)								
**Sex**	**Male**	14,017	4,977	35·5%	(34·7%, 36·3%)	(ref)	(ref)	(ref)	<0·0001	(ref)	(ref)	(ref)	0·00079
	**Female**	12,122	4,786	39·5%	(38·6%, 40·4%)	1·18	1·13	1·25		1·10	1·04	1·16	
**Diagnosis year**	**2012**	-	-										
	**2013**	6,537	2,490	38·1%	(36·9%, 39·3%)	(ref)	(ref)	(ref)	<0·0001	(ref)	(ref)	(ref)	0·46
	**2014**	6,586	2,482	37·7%	(36·5%, 38·9%)	0·98	0·92	1·05		0·99	0·92	1·06	
	**2015**	6,545	2,408	36·8%	(35·6%, 38·0%)	0·95	0·88	1·02		0·94	0·88	1·02	
	**2016**	6,471	2,383	36·8%	(35·7%, 38·0%)	0·95	0·88	1·02		0·98	0·91	1·06	
**Age group**	**15-64**	6,645	2,133	32·1%	(31·0%, 33·2%)	0·96	0·90	1·03	<0·0001	0·96	0·90	1·04	<0·0001
	**65-74**	8,453	2,791	33·0%	(32·0%, 34·0%)	(ref)	(ref)	(ref)		(ref)	(ref)	(ref)	
	**75-84**	7,758	3,088	39·8%	(38·7%, 40·9%)	1·34	1·26	1·43		1·35	1·26	1·45	
	**85+**	3,283	1,751	53·3%	(51·6%, 55·0%)	2·32	2·14	2·52		2·32	2·13	2·54	
**Stage**	**1**	3,310	575	17·4%	(16·1%, 18·7%)	(ref)	(ref)	(ref)	<0·0001	(ref)	(ref)	(ref)	<0·0001
	**2**	3,451	941	27·3%	(25·8%, 28·8%)	1·78	1·59	2·01		1·88	1·66	2·12	
	**3**	6,038	1,680	27·8%	(26·7%, 29·0%)	1·83	1·65	2·04		2·04	1·83	2·27	
	**4**	9,006	4,485	49·8%	(48·8%, 50·8%)	4·72	4·28	5·21		4·51	4·07	5.00	
	**Unknown**	4,334	2,082	48·0%	(46·6%, 49·5%)	4·40	3·95	4·90		3·64	3·25	4·08	
**Cancer site**	**Oesophageal**	1,703	435	25·5%	(23·5%, 27·7%)	0·66	0·58	0·74	<0·0001	0·6	0·53	0·68	<0·0001
	**Stomach**	1,656	648	39·1%	(36·8%, 41·5%)	1·24	1·10	1·38		0·98	0·88	1·11	
	**Colon**	6,142	2,102	34·2%	(33·0%, 35·4%)	(ref)	(ref)	(ref)		(ref)	(ref)	(ref)	
	**Rectal**	2,577	355	13·8%	(12·5%, 15·2%)	0·31	0·27	0·35		0·34	0·30	0·38	
	**Liver**	1,155	583	50·5%	(47·6%, 53·4%)	1·96	1·73	2·22		1·44	1·26	1·65	
	**Pancreatic**	1,965	1,115	56·7%	(54·5%, 58·9%)	2·52	2·27	2·8		1·85	1·65	2·06	
	**Lung**	9,365	3,882	41·5%	(40·5%, 42·5%)	1·36	1·27	1·45		1·20	1·12	1·29	
	**Ovarian**	1,576	643	40·8%	(38·4%, 43·2%)	1·32	1·18	1·48		1·48	1·31	1·68	

Alberta		All	EP	%EP	95% CI	Crude OR	Crude OR (LCI)	Crude OR (UCI)	p(COR)	Adjusted OR	Adj OR (LCI)	Adj OR (UCI)	p(AOR)
	**Total**	19,969	5,981	30·0%	(29·3%, 30·6%)								
**Sex**	**Male**	10,455	3,116	29·8%	(28·9%, 30·7%)	(ref)	(ref)	(ref)	0·63	(ref)	(ref)	(ref)	0·34
	**Female**	9,514	2,865	30·1%	(29·2%, 31·0%)	1·03	0·96	1·08		0·97	0·91	1·03	
**Diagnosis year**	**2012**	4,628	1,392	30·1%	(28·8%, 31·4%)	0·96	0·88	1·05	0·38	0·99	0·90	1·08	0·94
	**2013**	4,812	1,485	30·9%	(29·6%, 32·2%)	(ref)	(ref)	(ref)		(ref)	(ref)	(ref)	
	**2014**	5,307	1,570	29·6%	(28·4%, 30·8%)	0·94	0·86	1·02		0·97	0·89	1·07	
	**2015**	5,222	1,534	29·4%	(28·2%, 30·6%)	0·93	0·86	1·01		0·97	0·89	1·07	
**Age group**	**15-64**	7,701	1,947	25·3%	(24·3%, 26·3%)	0·96	0·89	1·04	<0·0001	0·98	0·9	1·06	<0·0001
	**65-74**	5,714	1,492	26·1%	(25·0%, 27·3%)	(ref)	(ref)	(ref)		(ref)	(ref)	(ref)	
	**75-84**	4,711	1,635	34·7%	(33·4%, 36·1%)	1·52	1·38	1·64		1·53	1·4	1·67	
	**85+**	1,843	907	49·2%	(46·9%, 51·5%)	2·74	2·46	3·06		2·71	2·41	3·04	
**Stage**	**1**	3,827	491	12·8%	(11·8%, 13·9%)	(ref)	(ref)	(ref)	<0·0001	(ref)	(ref)	(ref)	<0·0001
	**2**	3,042	725	23·8%	(22·4%, 25·4%)	2·01	1·87	2·41		1·87	1·75	2·27	
	**3**	4,420	1,038	23·5%	(22·3%, 24·8%)	1·99	1·86	2·35		2·09	1·96	2·49	
	**4**	7,733	3,399	44·0%	(42·9%, 45·1%)	5·13	4·80	5·92		5·01	4·70	5·84	
	**Unknown**	947	328	34·6%	(31·7%, 37·7%)	3·40	3·05	4·24		2·87	2·57	3·63	
**Cancer site**	**Oesophageal**	708	163	23·0%	(20·1%, 26·3%)	0·73	0·62	0·90	<0·0001	0·54	0·46	0·67	<0·0001
	**Stomach**	1,020	353	34·6%	(31·8%, 37·6%)	1·27	1·14	1·52		0·94	0·84	1·13	
	**Colon**	5,142	1,471	28·6%	(27·4%, 29·9%)	(ref)	(ref)	(ref)		(ref)	(ref)	(ref)	
	**Rectal**	1,976	254	12·9%	(11·5%, 14·4%)	0·37	0·32	0·42		0·39	0·33	0·45	
	**Liver**	1,054	334	31·7%	(29·0%, 34·6%)	1·15	1.00	1·33		1·21	1·04	1·41	
	**Pancreatic**	1,671	683	40·9%	(38·5%, 43·2%)	1·74	1·54	1·93		1·16	1·02	1·30	
	**Lung**	7,468	2,484	33·3%	(32·2%, 34·3%)	1·24	1·15	1·34		0·90	0·83	0·99	
	**Ovarian**	930	239	25·7%	(23·0%, 28·6%)	0·86	0·74	1·01		0·90	0·77	1·09	

Atlantic Canada $		All	EP	%EP	95% CI	Crude OR	Crude OR (LCI)	Crude OR (UCI)	p(COR)	Adjusted OR	Adj OR (LCI)	Adj OR (UCI)	p(AOR)
	**Total**	22,125	5,950	26·9%	(26·3%, 27·5%)								
**Sex**	**Male**	12,120	3,190	26·3%	(25·5%, 27·1%)	(ref)	(ref)	(ref)	0·054	(ref)	(ref)	(ref)	0·90
	**Female**	10,020	2,760	27·5%	(26·7%, 28·4%)	1·06	1.00	1·13		1.00	0·94	1·07	
**Diagnosis**	**2012**	5,405	1,450	26·8%	(25·7%, 28·0%)	1·04	0·95	1·13	0·35	1·05	0·96	1·14	0·33
**year**	**2013**	5,555	1,465	26·4%	(25·2%, 27·5%)	(ref)	(ref)	(ref)		(ref)	(ref)	(ref)	
	**2014**	5,660	1,565	27·7%	(26·5%, 28·8%)	1·08	0·99	1·17		1·08	0·99	1·18	
	**2015**	5,500	1,470	26·7%	(25·6%, 27·9%)	1·04	0·95	1·13		1·06	0·97	1·16	
**Age group**	**15-64**	7,110	1,570	22·1%	(21·1%, 23·1%)	0·91	0·84	0·98	<0·0001	0·89	0·82	0·97	<0·0001
	**65-74**	7,460	1,785	23·9%	(23·0%, 24·9%)	(ref)	(ref)	(ref)		(ref)	(ref)	(ref)	
	**75-84**	5,510	1,710	31·0%	(29·8%, 32·3%)	1·44	1·33	1·56		1·45	1·34	1·57	
	**85+**	2,035	890	43·7%	(41·6%, 45·9%)	2·46	2·22	2·73		2·32	2·09	2·59	
**Stage**	**1**	4,550	560	12·3%	(11·4%, 13·3%)	(ref)	(ref)	(ref)	<0·0001	(ref)	(ref)	(ref)	<0·0001
	**2**	3,250	660	20·3%	(19·0%, 21·7%)	1·82	1·61	2·06		1·68	1·48	1·90	
	**3**	4,515	970	21·5%	(20·3%, 22·7%)	1·92	1·72	2·15		2·02	1·80	2·27	
	**4**	7,275	2,830	38·9%	(37·8%, 40·0%)	4·47	4·04	4·94		4·65	4·20	5·15	
	**Unknown**	2,530	925	36·6%	(34·7%, 38·5%)	3·95	3·50	4·45		3·34	2·92	3·82	
**Cancer site**	**Oesophageal**	675	140	20·7%	(17·9%, 24·0%)	0·69	0·56	0·83	<0·0001	0·57	0·46	0·69	<0·0001
	**Stomach**	1,150	350	30·4%	(27·8%, 33·2%)	1·13	0·98	1·29		0·83	0·71	0·96	
	**Colon**	6,135	1,745	28·4%	(27·3%, 29·6%)	(ref)	(ref)	(ref)		(ref)	(ref)	(ref)	
	**Rectal**	2,100	265	12·6%	(11·3%, 14·1%)	0·36	0·31	0·41		0·37	0·32	0·43	
	**Liver**	685	235	34·3%	(30·8%, 37·9%)	1·28	1·08	1·51		1·02	0·85	1·23	
	**Pancreatic**	1,445	560	38·8%	(36·3%, 41·3%)	1·59	1·41	1·79		1·10	0·97	1·26	
	**Lung**	9,135	2,430	26·6%	(25·7%, 27·5%)	0·91	0·85	0·98		0·71	0·65	0·77	
	**Ovarian**	800	225	28·1%	(25·1%, 31·3%)	1.00	0·85	1·18		0·90	0·75	1·08	

British Columbia $		All	EP	%EP	95% CI	Crude OR	Crude OR (LCI)	Crude OR (UCI)	p(COR)	Adjusted OR	Adj OR (LCI)	Adj OR (UCI)	p(AOR)
	**Total**	33,655	10,255	30·5%	(30·0%, 31·0%)								
**Sex**	**Male**	17,645	5,330	30·2%	(29·5%, 30·9%)	(ref)	(ref)	(ref)	0.30	(ref)	(ref)	(ref)	0·34
	**Female**	16,010	4,925	30·8%	(30·1%, 31·5%)	1·03	0·98	1·07		0·98	0·93	1·03	
**Diagnosis year**	**2012**	8,110	2,500	30·8%	(29·8%, 31·8%)	0·94	0·88	1.00	<0·0001	0·95	0·89	1·02	0.00058
	**2013**	8,370	2,690	32·1%	(31·1%, 33·1%)	(ref)	(ref)	(ref)		(ref)	(ref)	(ref)	
	**2014**	8,710	2,610	30·0%	(29·0%, 30·9%)	0·91	0·85	0·97		0·90	0·84	0·96	
	**2015**	8,460	2,440	28·8%	(27·9%, 29·8%)	0·85	0·80	0·91		0·87	0·82	0·94	
**Age group**	**15-64**	10,700	2,820	26·4%	(25·5%, 27·2%)	1·03	0·97	1·10	<0·0001	1·02	0·96	1·09	<0·0001
	**65-74**	10,140	2,610	25·7%	(24·9%, 26·6%)	(ref)	(ref)	(ref)		(ref)	(ref)	(ref)	
	**75-84**	8,845	2,855	32·3%	(31·3%, 33·3%)	1·38	1·29	1·47		1·38	1·29	1·47	
	**85+**	3,950	1,960	49·6%	(48·1%, 51·2%)	2·83	2·62	3·06		2·80	2·58	3·03	
**Stage**	**1**	4,500	425	9·4%	(8·6%, 10·3%)	(ref)	(ref)	(ref)	<0·0001	(ref)	(ref)	(ref)	<0·0001
	**2**	3,810	835	21·9%	(20·6%, 23·3%)	2·73	2·41	3·10		2·67	2·35	3·04	
	**3**	5,880	1,380	23·5%	(22·4%, 24·6%)	2·96	2·64	3·34		3·07	2·73	3·46	
	**4**	8,505	3,715	43·7%	(42·6%, 44·7%)	7·53	6·76	8·41		7·30	6·54	8·17	
	**Unknown ^**	10,965	3,870	35·3%	(34·4%, 36·2%)	5·28	4·74	5·88		3·84	3·34	4·42	
**Cancer site**	**Oesophageal**	1,165	330	28·3%	(25·8%, 31·0%)	1·01	0·88	1·16	<0·0001	0·86	0·73	1·02	<0·0001
	**Stomach**	1,590	640	40·3%	(37·9%, 42·7%)	1·77	1·58	1·97		1·47	1·27	1·70	
	**Colon**	9,160	2,520	27·5%	(26·6%, 28·4%)	(ref)	(ref)	(ref)		(ref)	(ref)	(ref)	
	**Rectal**	3,430	410	12·0%	(10·9%, 13·1%)	0·36	0·32	0·40		0·36	0·32	0·40	
	**Liver**	1,760	670	38·1%	(35·8%, 40·4%)	1·62	1·46	1·80		1·43	1·24	1·65	
	**Pancreatic**	2,620	1,085	41·4%	(39·5%, 43·3%)	1·87	1·71	2·05		1·54	1·35	1·75	
	**Lung**	12,320	4,090	33·2%	(32·4%, 34·0%)	1·31	1·24	1·39		0·97	0·91	1·04	
	**Ovarian**	1,610	510	31·7%	(29·5%, 34·0%)	1·21	1·08	1·36		1·10	0·94	1·28	

Ontario		All	EP	%EP	95% CI	Crude OR	Crude OR (LCI)	Crude OR (UCI)	p(COR)	Adjusted OR	Adj OR (LCI)	Adj OR (UCI)	p(AOR)
	**Total**	92,447	24,131	26·1%	(25·8%, 26·4%)								
**Sex**	**Male**	48,146	12,434	25·8%	(25·4%, 26·2%)	(ref)	(ref)	(ref)	0·046	(ref)	(ref)	(ref)	0·20
	**Female**	44,301	11,697	26·4%	(26·0%, 26·8%)	1·03	1.00	1·06		1·02	0·99	1·05	
**Diagnosis year**	**2012**	18,090	4,581	25·3%	(24·7%, 26·0%)	1.00	0·95	1·05	0·00031	0·99	0·95	1·05	0.0014
	**2013**	17,978	4,557	25·3%	(24·7%, 26·0%)	(ref)	(ref)	(ref)		(ref)	(ref)	(ref)	
	**2014**	18,513	4,915	26·5%	(25·9%, 27·2%)	1·06	1·02	1·12		1·06	1·01	1·11	
	**2015**	18,859	5,093	27·0%	(26·4%, 27·6%)	1·09	1·04	1·14		1·08	1·03	1·14	
	**2016**	19,007	4,985	26·2%	(25·6%, 26·9%)	1·05	1.00	1·10		1·04	0·99	1·09	
**Age group**	**15-64**	33,177	7,293	22·0%	(21·5%, 22·4%)	0·91	0·87	0·94	<0·0001	0·92	0·88	0·95	<0·0001
	**65-74**	28,027	6,643	23·7%	(23·2%, 24·2%)	(ref)	(ref)	(ref)		(ref)	(ref)	(ref)	
	**75-84**	23,184	6,805	29·4%	(28·8%, 29·9%)	1·34	1·29	1·39		1·36	1·31	1·42	
	**85+**	8,059	3,390	42·1%	(41·0%, 43·1%)	2·34	2·22	2·46		2·46	2·33	2·60	
**Stage**	**1**	16,620	1,909	11·5%	(11·0%, 12·0%)	(ref)	(ref)	(ref)	<0·0001	(ref)	(ref)	(ref)	<0·0001
	**2**	13,177	2,858	21·7%	(21·0%, 22·4%)	2·13	2.00	2·27		1·94	1·82	2·07	
	**3**	19,476	4,233	21·7%	(21·2%, 22·3%)	2·14	2·02	2·27		2·19	2·06	2·32	
	**4**	27,579	10,331	37·5%	(36·9%, 38·0%)	4·62	4·38	4·87		4·72	4·47	4·99	
	**Unknown**	15,595	4,800	30·8%	(30·1%, 31·5%)	3·43	3·23	3·63		3·40	3·19	3·63	
**Cancer site**	**Oesophageal**	3,099	630	20·3%	(18·9%, 21·8%)	0·68	0·62	0·75	<0·0001	0·50	0·45	0·55	<0·0001
	**Stomach**	6,465	1,738	26·9%	(25·8%, 28·0%)	0·99	0·93	1·05		0·70	0·66	0·75	
	**Colon**	24,481	6,653	27·2%	(26·6%, 27·7%)	(ref)	(ref)	(ref)		(ref)	(ref)	(ref)	
	**Rectal**	7,845	860	11·0%	(10·3%, 11·7%)	0·33	0·31	0·36		0·35	0·32	0·38	
	**Liver**	4,129	1,167	28·3%	(26·9%, 29·7%)	1·05	0·98	1·14		0·89	0·82	0·96	
	**Pancreatic**	6,456	2,279	35·3%	(34·1%, 36·5%)	1·46	1·38	1·55		1·09	1·02	1·16	
	**Lung**	35,092	9,640	27·5%	(27·0%, 27·9%)	1·01	0·98	1·05		0·80	0·77	0·83	
	**Ovarian**	4,880	1,164	23·9%	(22·7%, 25·1%)	0·84	0·78	0·90		0·80	0·74	0·87	

Saskatchewan-Manitoba $		All	EP	%EP	95% CI	Crude OR	Crude OR (LCI)	Crude OR (UCI)	p(COR)	Adjusted OR	Adj OR (LCI)	Adj OR (UCI)	p(AOR)
	**Total**	16,655	4,715	28·3%	(27·6%, 29·0%)								
**Sex**	**Male**	8,780	2,375	27·1%	(26·1%, 28·0%)	(ref)	(ref)	(ref)	0·00036	(ref)	(ref)	(ref)	0·058
	**Female**	7,875	2,330	29·6%	(28·6%, 30·6%)	1·13	1·06	1·21		1·07	1.00	1·16	
**Diagnosis year**	**2012**	4,080	1,150	28·2%	(26·8%, 29·6%)	0·95	0·87	1·05	0·41	0·95	0·86	1·05	0·55
	**2013**	4,285	1,255	29·3%	(27·9%, 30·7%)	(ref)	(ref)	(ref)		(ref)	(ref)	(ref)	
	**2014**	4,250	1,220	28·7%	(27·4%, 30·1%)	0·98	0·89	1·07		0·97	0·88	1·07	
	**2015**	4,045	1,105	27·3%	(26·0%, 28·7%)	0·92	0·84	1·02		0·93	0·84	1·03	
**Age group**	**15-64**	5,545	1,275	23·0%	(21·9%, 24·1%)	0·90	0·82	0·98	<0·0001	0·91	0·83	1.00	<0·0001
	**65-74**	4,990	1,275	25·6%	(24·4%, 26·8%)	(ref)	(ref)	(ref)		(ref)	(ref)	(ref)	
	**75-84**	4,215	1,335	31·7%	(30·3%, 33·1%)	1·36	1·24	1·49		1·42	1·30	1·57	
	**85+**	1,910	825	43·2%	(41·0%, 45·4%)	2·31	2·06	2·58		2·44	2·17	2·74	
**Stage**	**1**	3,340	470	14·1%	(12·9%, 15·3%)	(ref)	(ref)	(ref)	<0·0001	(ref)	(ref)	(ref)	<0·0001
	**2**	2,630	610	23·2%	(21·6%, 24·8%)	1·79	1·57	2·04		1·71	1·49	1·96	
	**3**	3,680	825	22·4%	(21·1%, 23·8%)	1·74	1·54	1·97		1·90	1·68	2·16	
	**4**	6,145	2,520	41·0%	(39·8%, 42·2%)	4·16	3·73	4·65		4·41	3·94	4·94	
	**Unknown**	870	290	33·3%	(30·3%, 36·5%)	3·13	2·64	3·71		2·71	2·26	3·24	
**Cancer site**	**Oesophageal**	460	85	18·5%	(15·2%, 22·3%)	0·59	0·46	0·75	<0·0001	0·49	0·38	0·62	<0·0001
	**Stomach**	895	285	31·8%	(28·9%, 35·0%)	1·19	1·02	1·39		0·97	0·82	1·14	
	**Colon**	4,470	1,240	27·7%	(26·4%, 29·1%)	(ref)	(ref)	(ref)		(ref)	(ref)	(ref)	
	**Rectal**	1,820	250	13·7%	(12·2%, 15·4%)	0·41	0·35	0·48		0·42	0·36	0·49	
	**Liver**	585	195	33·3%	(29·6%, 37·3%)	1·27	1·06	1·53		1·28	1·05	1·56	
	**Pancreatic**	1,300	485	37·3%	(34·7%, 40·0%)	1·56	1·37	1·77		1·08	0·94	1·24	
	**Lung**	6,395	1,955	30·6%	(29·5%, 31·7%)	1·15	1·06	1·25		0·85	0·78	0·94	
	**Ovarian**	730	220	30·1%	(26·9%, 33·6%)	1·12	0·94	1·33		1·21	1.00	1·45	

New South Wales		All	EP	%EP	95% CI	Crude OR	Crude OR (LCI)	Crude OR (UCI)	p(COR)	Adjusted OR	Adj OR (LCI)	Adj OR (UCI)	p(AOR)
	**Total**	53,198	16,445	30·9%	(30·5%, 31·3%)								
**Sex**	**Male**	29,037	8,925	30·7%	(30·2%, 31·3%)	(ref)	(ref)	(ref)	0·34	(ref)	(ref)	(ref)	0·83
	**Female**	24,161	7,520	31·1%	(30·5%, 31·7%)	1·02	0·98	1·06		1.00	0·96	1·04	
**Diagnosis year**	**2012**	10,361	3,294	31·8%	(30·9%, 32·7%)	(ref)	(ref)	(ref)	0·045	(ref)	(ref)	(ref)	0·010
	**2013**	10,368	3,197	30·8%	(30·0%, 31·7%)	0·96	0·90	1·01		0·97	0·91	1·03	
	**2014**	10,432	3,112	29·8%	(29·0%, 30·7%)	0·91	0·86	0·97		0·93	0·88	0·99	
	**2015**	10,903	3,390	31·1%	(30·2%, 32·0%)	0·97	0·91	1·03		1·02	0·96	1·09	
	**2016**	11,134	3,452	31·0%	(30·2%, 31·9%)	0·96	0·91	1·03		1·02	0·96	1·09	
**Age group**	**15-64**	17,708	4,913	27·7%	(27·1%, 28·4%)	1·09	1·04	1·14	<0·0001	1·11	1·06	1·17	<0·0001
	**65-74**	16,085	4,190	26·0%	(25·4%, 26·7%)	(ref)	(ref)	(ref)		(ref)	(ref)	(ref)	
	**75-84**	13,802	4,605	33·4%	(32·6%, 34·2%)	1·42	1·35	1·49		1·46	1·39	1·54	
	**85+**	5,603	2,737	48·8%	(47·5%, 50·2%)	2·71	2·55	2·89		3·07	2·87	3·28	
**Stage**	**Localised**	14,325	3,038	21·2%	(20·5%, 21·9%)	(ref)	(ref)	(ref)	<0·0001	(ref)	(ref)	(ref)	<0·0001
	**Inv. adjc. tissue/organ**	6,262	1,683	26·9%	(25·8%, 28·0%)	1·37	1·27	1·46		1·40	1·30	1·50	
	**Inv. regional LNs**	8,854	2,088	23·6%	(22·7%, 24·5%)	1·15	1·08	1·22		1·26	1·18	1·35	
	**Distant metastasis**	17,181	8,023	46·7%	(46·0%, 47·4%)	3·25	3·10	3·42		3·19	3·02	3·36	
	**Unknown**	6,576	1,613	24·5%	(23·5%, 25·6%)	1·21	1·13	1·29		1·03	0·96	1·11	
**Cancer site**	**Oesophageal**	1,970	530	26·9%	(25·0%, 28·9%)	0·97	0·87	1·07	<0·0001	0·94	0·84	1·05	<0·0001
	**Stomach**	3,179	969	30·5%	(28·9%, 32·1%)	1·15	1·06	1·25		1·08	0·99	1·18	
	**Colon**	16,316	4,499	27·6%	(26·9%, 28·3%)	(ref)	(ref)	(ref)		(ref)	(ref)	(ref)	
	**Rectal**	5,459	660	12·1%	(11·3%, 13·0%)	0·36	0·33	0·39		0·41	0·37	0·45	
	**Liver**	3,155	1,161	36·8%	(35·1%, 38·5%)	1·53	1·41	1·66		1·91	1·75	2·08	
	**Pancreatic**	4,498	2,036	45·3%	(43·8%, 46·7%)	2·17	2·03	2·32		1·74	1·62	1·87	
	**Lung**	16,334	5,941	36·4%	(35·6%, 37·1%)	1·50	1·43	1·57		1·26	1·19	1·32	
	**Ovarian**	2,287	649	28·4%	(26·6%, 30·3%)	1·04	0·94	1·15		0·72	0·64	0·79	

Victoria		All	EP	%EP	95% CI	Crude OR	Crude OR (LCI)	Crude OR (UCI)	p(COR)	Adjusted OR	Adj OR (LCI)	Adj OR (UCI)	p(AOR)
	**Total**	38,212	9,165	24·0%	(23·6%, 24·4%)								
**Sex**	**Male**	20,720	4,980	24·0%	(23·5%, 24·6%)	(ref)	(ref)	(ref)	0·80	(ref)	(ref)	(ref)	0·13
	**Female**	17,492	4,185	23·9%	(23·3%, 24·6%)	0·99	0·95	1·04		0·96	0·91	1·01	
**Diagnosis year**	**2012**	7,045	1,615	22·9%	(22·0%, 23·9%)	(ref)	(ref)	(ref)	0·00024	(ref)	(ref)	(ref)	0·0040
	**2013**	7,232	1,638	22·6%	(21·7%, 23·6%)	0·98	0·91	1·06		0·99	0·91	1·07	
	**2014**	7,646	1,832	24·0%	(23·0%, 24·9%)	1·06	0·98	1·14		1·03	0·95	1·11	
	**2015**	7,961	1,998	25·1%	(24·2%, 26·1%)	1·13	1·05	1·21		1·11	1·02	1·20	
	**2016**	8,328	2,082	25·5%	(24·1%, 25·9%)	1·12	1·04	1·21		1·10	1·02	1·19	
**Age group**	**15-64**	12,568	2,618	20·8%	(20·1%, 21·5%)	1·04	0·98	1·11	<0·0001	1·05	0·99	1·13	<0·0001
	**65-74**	11,016	2,226	20·2%	(19·5%, 21·0%)	(ref)	(ref)	(ref)		(ref)	(ref)	(ref)	
	**75-84**	10,188	2,653	26·0%	(25·2%, 26·9%)	1·39	1·30	1·48		1·38	1·29	1·47	
	**85+**	4,440	1,668	37·6%	(36·2%, 39·0%)	2·38	2·2	2·56		2·29	2·11	2·47	
**Stage***	**Localised**	3,587	193	5·4%	(4·7%, 6·2%)	(ref)	(ref)	(ref)	<0·0001	(ref)	(ref)	(ref)	<0·0001
	**Inv. adjc. tissue/organ**	4,011	710	17·7%	(16·6%, 18·9%)	3·78	3·21	4·48		3·25	2·76	3·86	
	**Inv. regional LNs**	3,708	704	19·0%	(17·8%, 20·3%)	4·12	3·5	4·88		3·90	3·30	4·63	
	**Distant metastasis**	2,727	903	33·1%	(31·4%, 34·9%)	8·71	7·39	10·3		8·21	6·95	9·73	
	**Unknown ^**	24,146	6,651	27·5%	(27·0%, 28·1%)	6·69	5·78	7·77		7.00	5·87	8·38	
**Cancer site**	**Oesophageal**	1,576	315	20·0%	(18·1%, 22·0%)	0·84	0·74	0·96	<0·0001	0·47	0·40	0·55	<0·0001
	**Stomach**	2,597	616	23·7%	(22·1%, 25·4%)	1·05	0·95	1·16		0·59	0·51	0·68	
	**Colon**	11,747	2,689	22·9%	(22·1%, 23·7%)	(ref)	(ref)	(ref)		(ref)	(ref)	(ref)	
	**Rectal**	4,438	403	9·1%	(8·3%, 10·0%)	0·34	0·30	0·38		0·34	0·30	0·38	
	**Liver**	2,323	742	31·9%	(30·1%, 33·9%)	1·58	1·43	1·74		0·93	0·81	1·07	
	**Pancreatic**	3,172	1,083	34·1%	(32·5%, 35·8%)	1·75	1·60	1·90		0·97	0·85	1·10	
	**Lung**	10,620	2,954	27·8%	(27·0%, 28·7%)	1·30	1·22	1·38		0·74	0·66	0·83	
	**Ovarian**	1,739	363	20·9%	(19·0%, 22·8%)	0·89	0·78	1.00		0·55	0·47	0·65	

New Zealand		All	EP	%EP	95% CI	Crude OR	Crude OR (LCI)	Crude OR (UCI)	p(COR)	Adjusted OR	Adj OR (LCI)	Adj OR (UCI)	p(AOR)
	**Total**	28,794	12,238	42·5%	(41·9%, 43·1%)								
**Sex**	**Male**	14,751	6,164	41·8%	(41·0%, 42·6%)	(ref)	(ref)	(ref)	0·012	(ref)	(ref)	(ref)	0·66
	**Female**	14,043	6,074	43·3%	(42·4%, 44·1%)	1·06	1·01	1·11		0·99	0·94	1·04	
**Diagnosis year**	**2012**	6,961	2,917	41·9%	(40·8%, 43·1%)	(ref)	(ref)	(ref)	0·25	(ref)	(ref)	(ref)	0·21
	**2013**	6,994	3,009	43·0%	(41·9%, 44·2%)	1·05	0·98	1·12		1·06	0·99	1·14	
	**2014**	7,447	3,122	41·9%	(40·8%, 43·0%)	1.00	0·94	1·07		1·02	0·95	1·09	
	**2015**	7,392	3,190	43·2%	(42·0%, 44·3%)	1·05	0·99	1·12		1·07	0·99	1·15	
**Age group**	**15-64**	8,819	3,622	41·1%	(40·0%, 42·1%)	1·08	1·01	1·14	<0·0001	1·06	1.00	1·14	<0·0001
	**65-74**	8,785	3,452	39·3%	(38·3%, 40·3%)	(ref)	(ref)	(ref)		(ref)	(ref)	(ref)	
	**75-84**	7,887	3,426	43·4%	(42·3%, 44·5%)	1·19	1·12	1·26		1·27	1·19	1·36	
	**85+**	3,303	1,738	52·6%	(50·9%, 54·3%)	1·72	1·58	1·86		1·86	1·71	2·03	
**Stage**	**Localised**	3,893	661	17·0%	(15·8%, 18·2%)	0·12	0·11	0·13	<0·0001	0·15	0·13	0·16	<0·0001
	**Inv. adjc. tissue/organ**	2,421	793	32·8%	(30·9%, 34·7%)	0·29	0·26	0·32		0·33	0·30	0·37	
	**Inv. regional LNs**	4,213	1,545	36·7%	(35·2%, 38·1%)	0·34	0·32	0·37		0·4	0·37	0·44	
	**Distant metastasis**	9,766	6,130	62·8%	(61·8%, 63·7%)	(ref)	(ref)	(ref)		(ref)	(ref)	(ref)	
	**Unknown**	8,501	3,109	36·6%	(35·6%, 37·6%)	0·34	0·32	0·36		0·35	0·32	0·37	
**Cancer site**	**Oesophageal**	1,196	440	36·8%	(34·1%, 39·6%)	1·01	0·89	1·14	<0·0001	0·86	0·75	0·99	<0·0001
	**Stomach**	1,520	726	47·8%	(45·3%, 50·3%)	1·58	1·42	1·76		1·35	1·20	1·52	
	**Colon**	9,502	3,480	36·6%	(35·7%, 37·6%)	(ref)	(ref)	(ref)		(ref)	(ref)	(ref)	
	**Rectal**	3,247	643	19·8%	(18·5%, 21·2%)	0·43	0·39	0·47		0·45	0·41	0·50	
	**Liver**	1,243	618	49·7%	(46·9%, 52·5%)	1·71	1·52	1·93		1·76	1·55	2·01	
	**Pancreatic**	2,182	1,317	60·4%	(58·3%, 62·4%)	2·63	2·40	2·90		1·78	1·60	1·97	
	**Lung**	8,481	4,330	51·1%	(50·0%, 52·1%)	1·81	1·70	1·92		1·38	1·29	1·48	
	**Ovarian**	1,423	684	48·1%	(45·5%, 50·7%)	1·60	1·43	1·79		1·07	0·95	1·22	

All: All patients; EP: Emergency Presentation; OR: Odds ratio; LCI: Lower confidence interval; UCI: Upper confidence interval; COR: Crude odds ratio; AOR: Adjusted odds ratio; Inv: Invading; Adjc: Adjacent; LNs: Lymph Nodes; Ref: Reference category.

* Data shown for stage categories in Victoria does not include 33 cases in groups other than shown.

^ For British Columbia the unknown stage category includes all cases other than colon, rectal and lung, and the proportion of cases with the latter three cancers with unknown stage. For Victoria, the unknown stage category includes all cases other than colon and rectal, and the proportion of cases with the latter two cancers with unknown stage.

$ For Atlantic Canada, British Columbia, and Saskatchewan-Manitoba, random rounding within cells to a multiple of 5 was used for the tabulated variable groups, to adhere to rules for minimising residual disclosure risk.

Supplementary Material 8. Meta-analysis of associations between patient-level variables (cancer site, age group, sex) and risk of emergency presentation (defined as diagnosis of cancer within 30 days after an emergency hospital admission) adjusted for emergency presentation broad/narrow definition subtype

Across the graphics, superscript indicators ‘n’ and ‘b’ after jurisdictional names describe whether a narrow or broad operational definition of emergency presentation was used – please also see main paper-Methods, and appendix pp.4-5.

A. Meta-analysis of risk of emergency presentation by cancer site

Oesophageal vs Colon (reference)

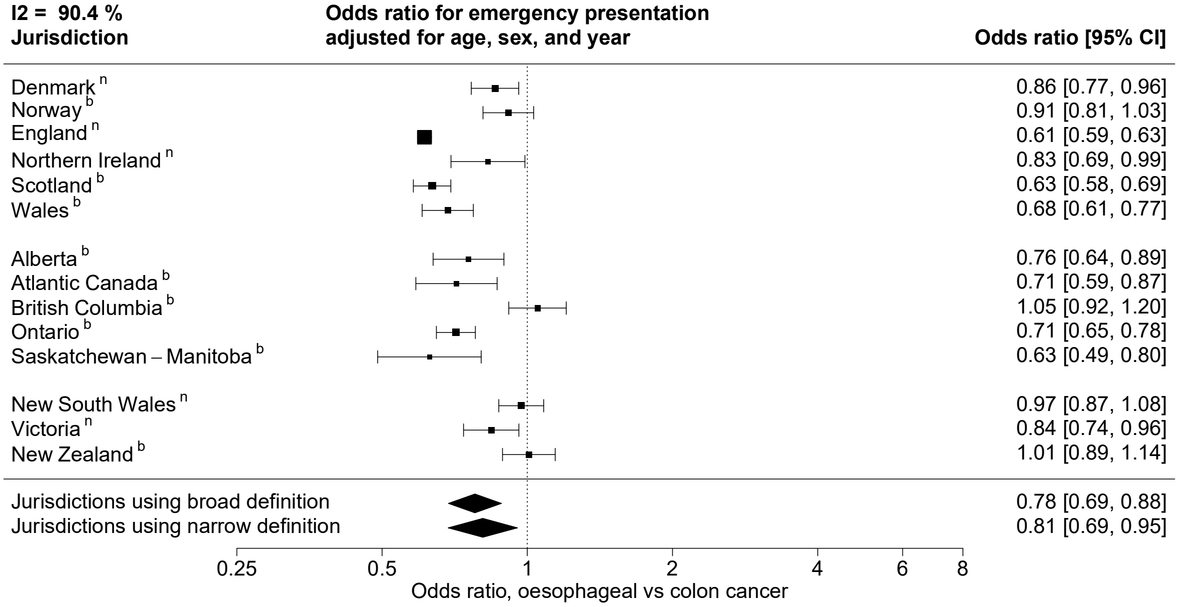

Stomach vs Colon (reference)

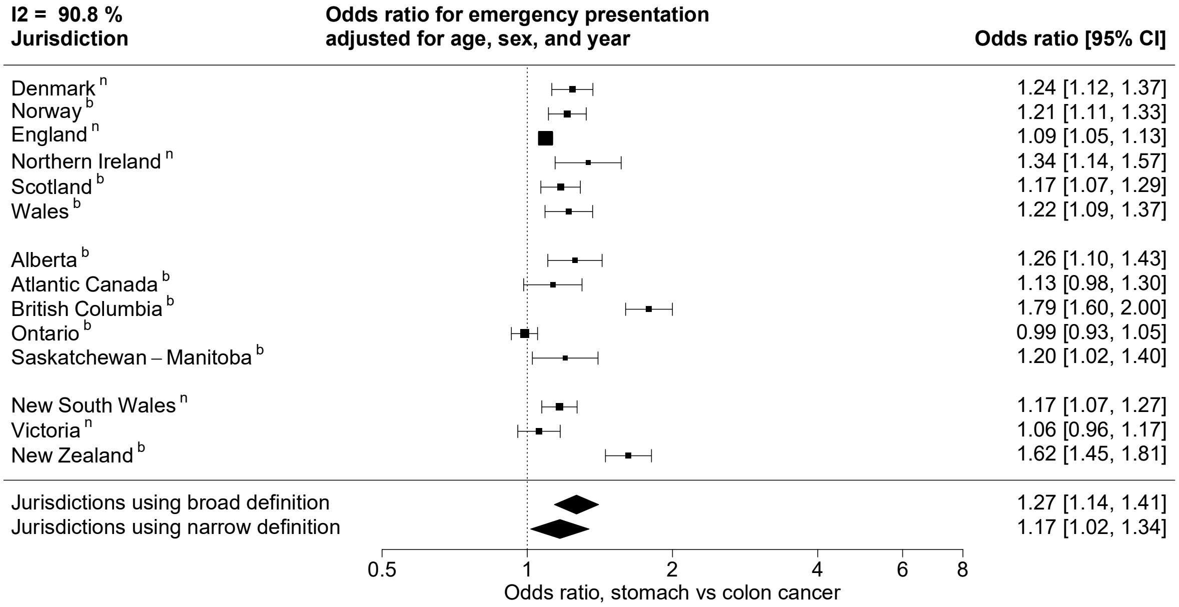

Rectal vs Colon (reference)

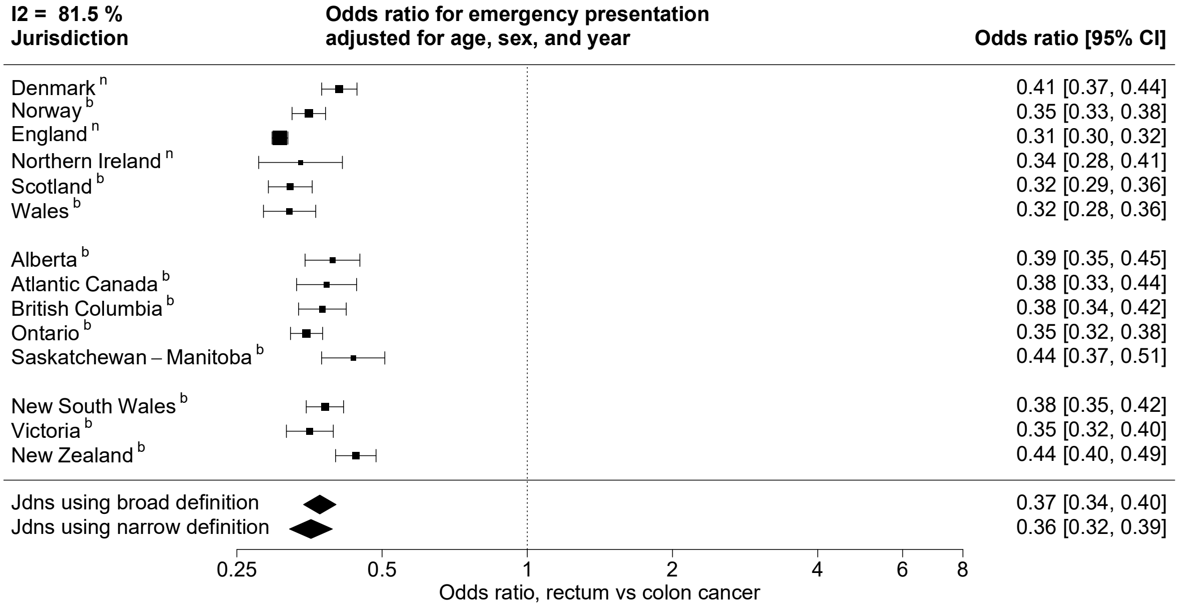

Liver vs. Colon (reference)

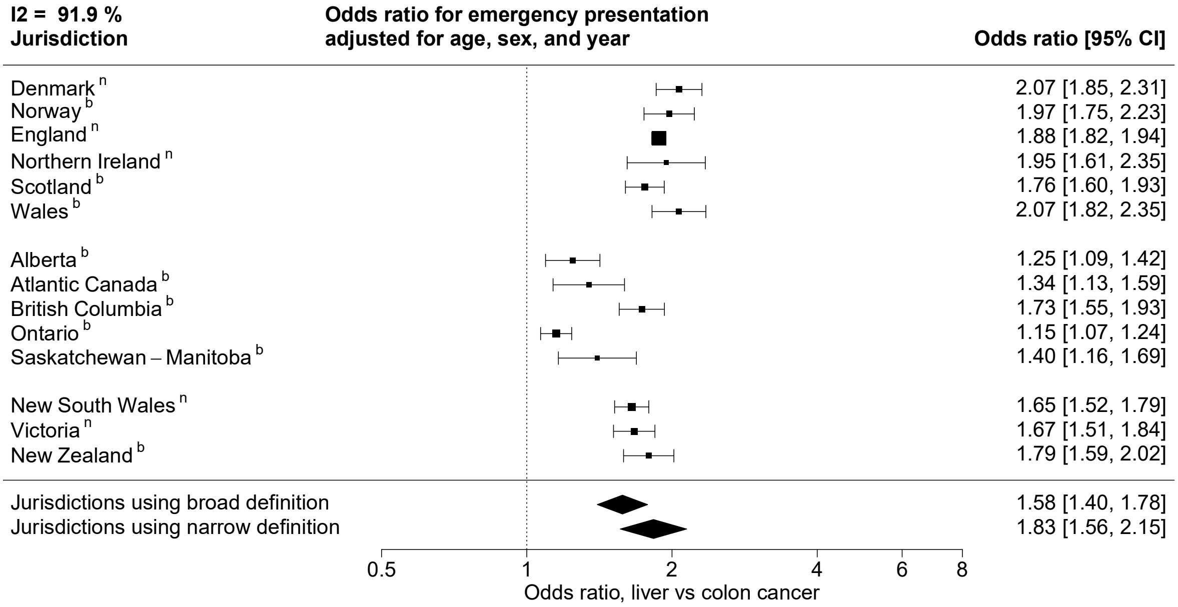

Pancreatic vs. Colon (reference)

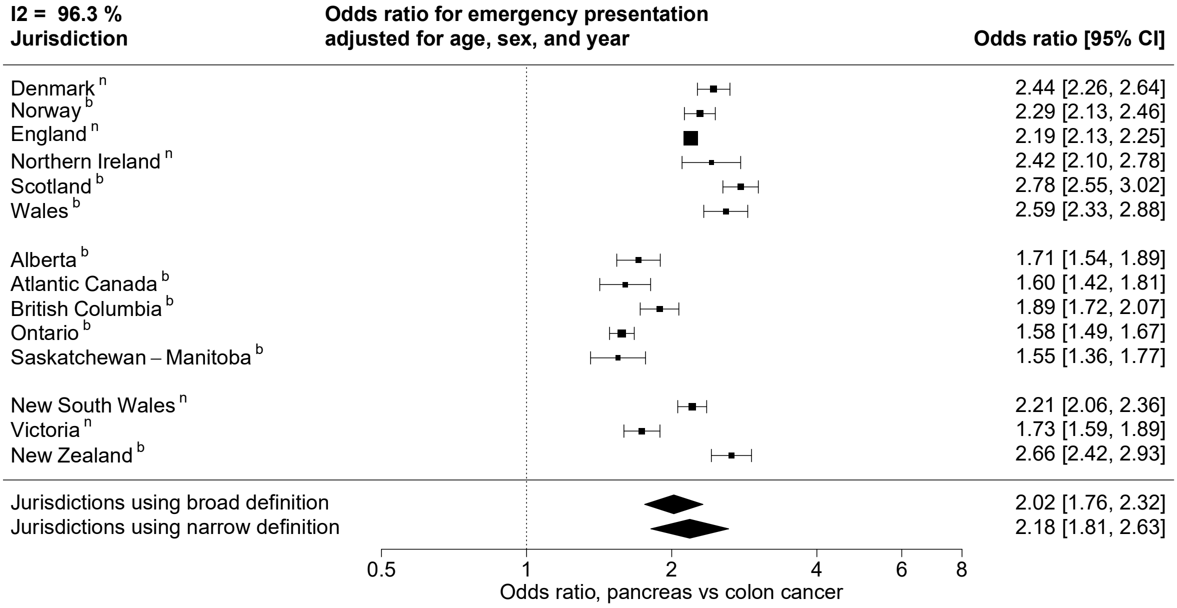

Lung vs. Colon (reference)

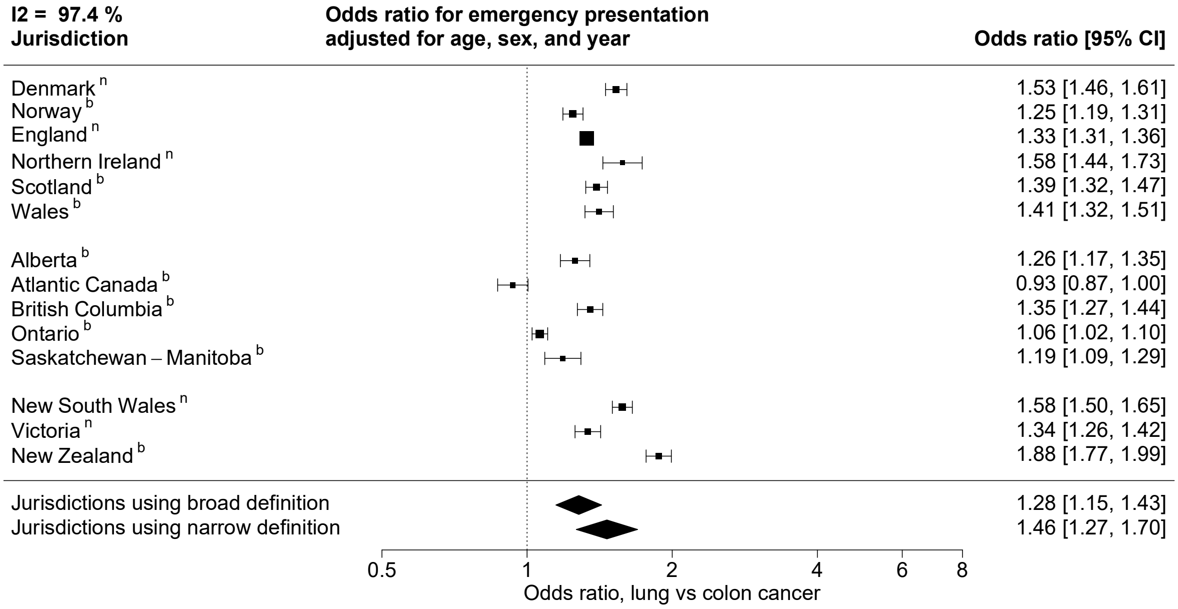

Ovarian vs. Colon (reference)

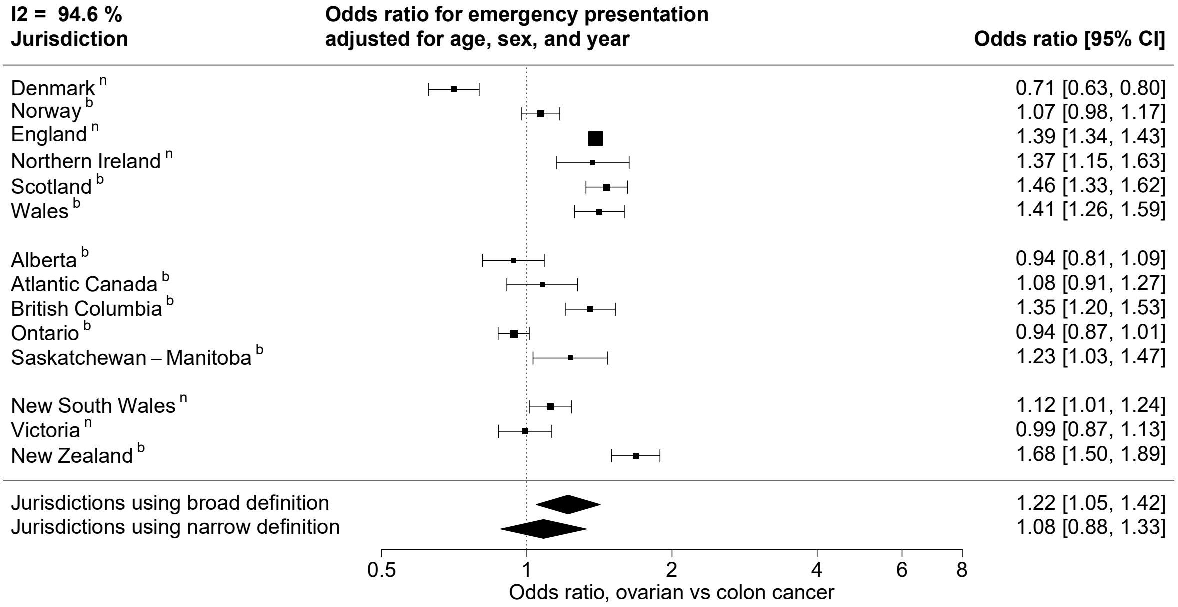

B. Meta-analysis of risk of emergency presentation by age group

15-64 vs 65-74 (reference)

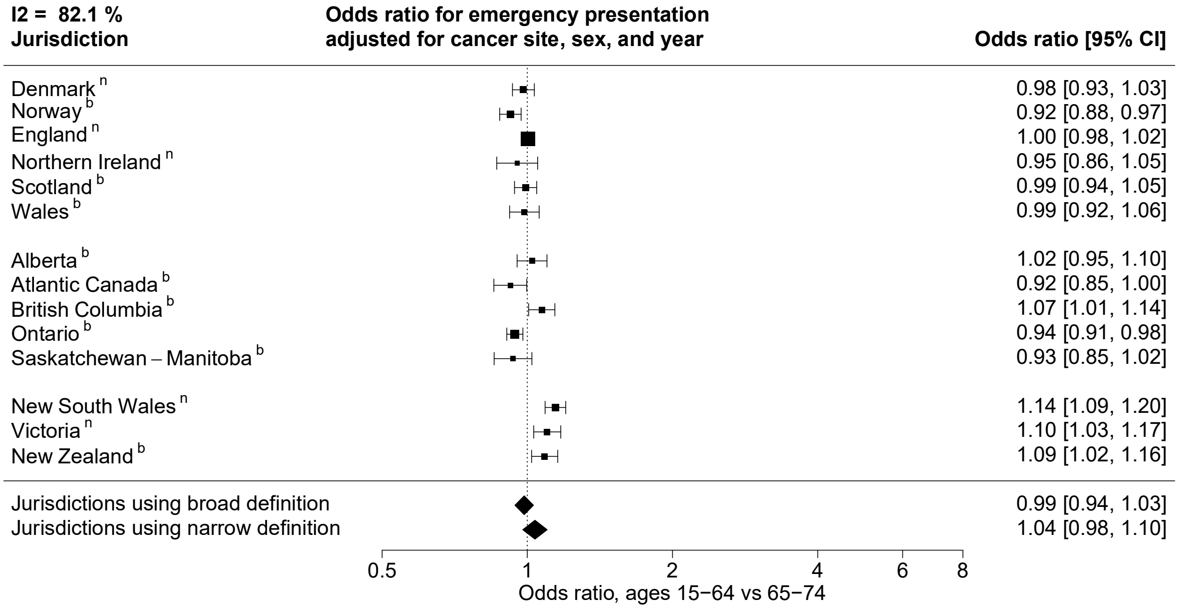

75-84 vs 65-74 (reference)

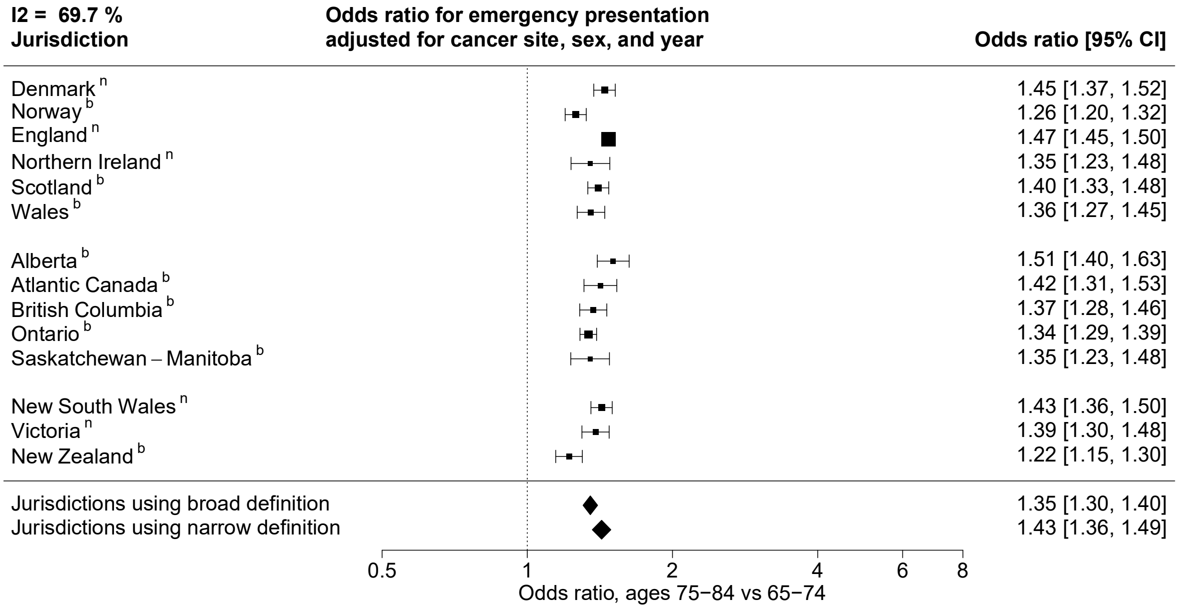

85-99 vs 65-74 (reference)

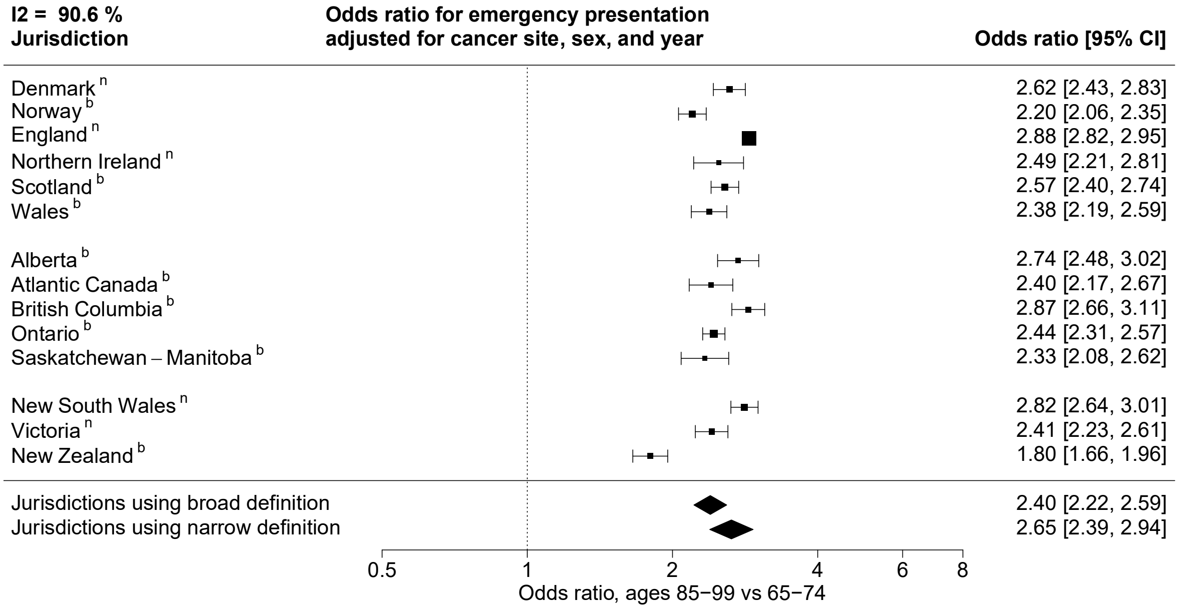

C. Meta-analysis of risk of emergency presentation by sex

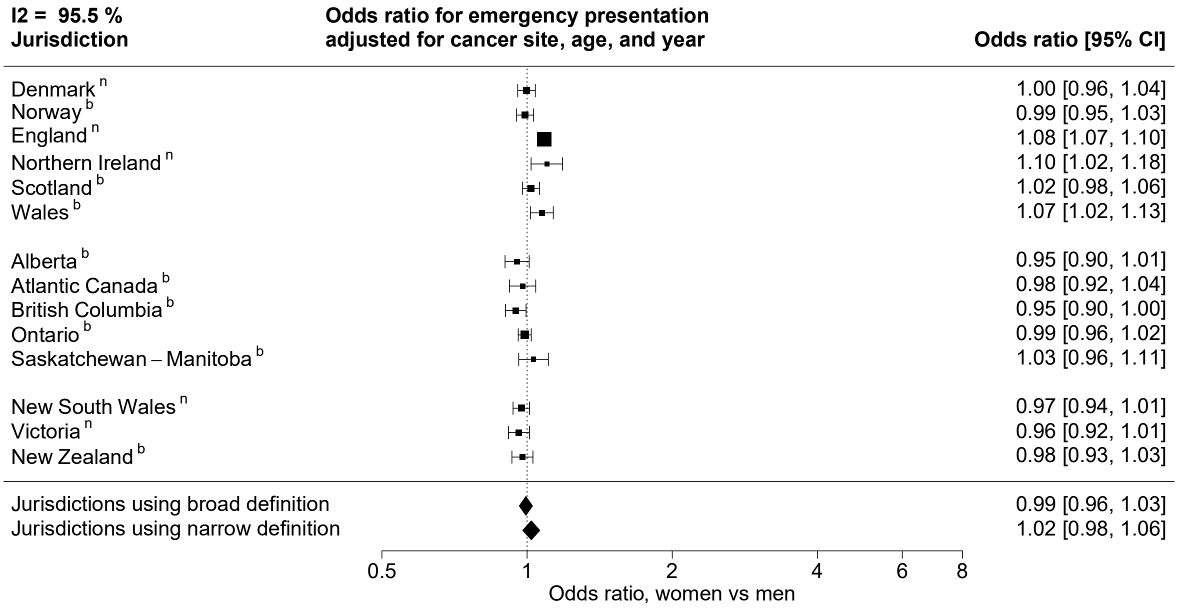

Supplementary Material 9. Risk of mortality by emergency presentation status (defined as diagnosis of cancer within 30 days after an emergency hospital admission) and cancer site, at 1-, 3- and 12-month follow-up, by jurisdiction

Across the jurisdictions, the median odds ratio value (for mortality) for emergency vs non-emergency presentation was 5·64 at 1-month, 5·22 at 3- months and 4·09 at 12 months.

**A.**	**Oesophageal**					**Stomach**					**Colon**					**Rectal**				
	Mortality					*Mortality*					*Mortality*					*Mortality*				
	EP *(%)*	***non-EP*** *(%)*	***Diff*** *(%)*	**Crude OR**	**95% CI** *(LCI, UCI)*	**EP** *(%)*	**non-EP** *(%)*	***Diff*** *(%)*	**Crude OR**	**95% CI** *(LCI, UCI)*	**EP** *(%)*	**non-EP** *(%)*	***Diff*** *(%)*	**Crude OR**	**95% CI** *(LCI, UCI)*	***EP*** *(%)*	***non-EP*** *(%)*	***Diff*** *(%)*	**Crude OR**	**95% CI** *(LCI, UCI)*
1-month																				
**Denmark**	20	4	*16*	*6·14*	*(4·32, 8·73)*	17	3	*14*	*6·22*	*(4·41, 8·78)*	11	2	*10*	*7·44*	*(6·16, 8·98)*	9	1	*8*	*9·76*	*(6·72, 14·19)*
**Norway**	20	3	*17*	*8·26*	*(5·23, 13·02)*	16	3	*14*	*7·41*	*(5·10, 10·77)*	11	2	*9*	*5·64*	*(4·79, 6·64)*	10	1	*9*	*11·85*	*(8·19, 17·13)*
**England**	30	4	*26*	*11·06*	*(10·09, 12·12)*	29	5	*24*	*7·35*	*(6·70, 8·06)*	20	3	*17*	*8·29*	*(7·84, 8·77)*	19	1	*18*	*17·47*	*(15·36, 19·87)*
**Northern Ireland**	23	4	*19*	*7·34*	*(4·49, 11·98)*	27	5	*22*	*6·40*	*(4·22, 9·71)*	17	3	*14*	*7·08*	*(5·34, 9·37)*	17	1	*16*	*19·23*	*(9·36, 39·51)*
**Scotland**	19	3	*16*	*8·50*	*(6·30, 11·47)*	20	4	*17*	*6·70*	*(4·87, 9·24)*	15	2	*13*	*8·19*	*(6·72, 9·97)*	11	1	*10*	*13·04*	*(8·17, 20·80)*
**Wales**	23	4	*19*	*7·23*	*(5·03, 10·41)*	25	4	*21*	*7·48*	*(5·25, 10·65)*	17	2	*15*	*9·87*	*(7·72, 12·64)*	16	1	*15*	*18·29*	*(11·10, 30·12)*
**Alberta**	17	4	*13*	*4·54*	*(2·54, 8·10)*	22	7	*15*	*3·80*	*(2·56, 5·63)*	13	2	*11*	*6·89*	*(5·26, 9·03)*	10	1	*8*	*8·53*	*(4·68, 15·53)*
**Atlantic Canada**	33	6	*27*	*8·00*	*(4·84, 13·23)*	27	4	*23*	*9·38*	*(6·09, 14·46)*	11	2	*9*	*5·41*	*(4·22, 6·93)*	17	1	*16*	*18·50*	*(10·68, 32·06)*
**British Columbia**	21	2	*18*	*10·83*	*(6·43, 18·22)*	20	4	*16*	*5·42*	*(3·77, 7·78)*	10	2	*8*	*6·91*	*(5·47, 8·73)*	11	1	*10*	*17·32*	*(10·20, 29·39)*
**Ontario**	20	3	*17*	*7·61*	*(5·67, 10·23)*	18	3	*14*	*6·06*	*(4·96, 7·41)*	9	1	*7*	*6·44*	*(5·54, 7·49)*	9	1	*8*	*15·95*	*(10·81, 23·52)*
**Saskatchewan-Manitoba**	43	3	*40*	*26·25*	*(12·39, 55·62)*	17	3	*14*	*5·83*	*(3·42, 9·94)*	12	2	*10*	*8·60*	*(6·18, 11·97)*	15	1	*15*	*28·73*	*(14·07, 58·66)*
**New South Wales**	18	3	*15*	*6·87*	*(4·76, 9·92)*	14	2	*12*	*6·62*	*(4·80, 9·13)*	9	1	*8*	*8·41*	*(6·94, 10·20)*	6	0	*6*	*14·11*	*(8·43, 23·63)*
**Victoria**	25	3	*22*	*9·26*	*(6·24, 13·73)*	19	2	*17*	*10·18*	*(7·14, 14·50)*	11	1	*9*	*8·24*	*(6·66, 10·18)*	8	1	*8*	*14·31*	*(8·42, 24·32)*
**New Zealand**	14	2	*12*	*9·98*	*(5·31, 18·76)*	18	3	*15*	*7·26*	*(4·64, 11·36)*	10	1	*9*	*8·52*	*(6·66, 10·90)*	7	1	*7*	*11·33*	*(6·53, 19·65)*
3-month																				
**Denmark**	41	14	*28*	*4·50*	*(3·54, 5·71)*	38	12	*26*	*4·59*	*(3·69, 5·70)*	21	5	*16*	*5·25*	*(4·65, 5·94)*	18	3	*15*	*6·50*	*(5·13, 8·24)*
**Norway**	36	11	*25*	*4·65*	*(3·49, 6·19)*	34	9	*25*	*5·06*	*(4·04, 6·33)*	20	5	*15*	*4·92*	*(4·39, 5·51)*	19	2	*17*	*9·17*	*(7·16, 11·76)*
**England**	55	14	*41*	*7·24*	*(6·78, 7·73)*	53	17	*36*	*5·33*	*(4·98, 5·69)*	34	8	*26*	*5·80*	*(5·58, 6·03)*	35	4	*31*	*11·79*	*(10·80, 12·86)*
**Northern Ireland**	43	13	*30*	*4·84*	*(3·41, 6·88)*	54	17	*37*	*5·61*	*(4·14, 7·59)*	30	7	*23*	*5·49*	*(4·50, 6·70)*	38	4	*34*	*14·47*	*(9·14, 22·91)*
**Scotland**	48	13	*34*	*5·99*	*(5·02, 7·15)*	47	16	*31*	*4·74*	*(3·91, 5·75)*	30	7	*22*	*5·31*	*(4·71, 5·99)*	28	3	*25*	*11·95*	*(9·01, 15·86)*
**Wales**	51	16	*35*	*5·57*	*(4·38, 7·08)*	49	18	*31*	*4·26*	*(3·41, 5·32)*	33	8	*25*	*5·68*	*(4·91, 6·58)*	36	5	*31*	*11·58*	*(8·63, 15·54)*
**Alberta**	49	13	*35*	*6·13*	*(4·13, 9·09)*	43	17	*26*	*3·58*	*(2·67, 4·78)*	23	6	*17*	*4·54*	*(3·80, 5·44)*	21	4	*17*	*6·80*	*(4·58, 10·09)*
**Atlantic Canada**	36	19	*16*	*2·33*	*(1·55, 3·51)*	47	18	*30*	*4·22*	*(3·19, 5·57)*	18	5	*13*	*3·88*	*(3·24, 4·63)*	32	5	*27*	*9·31*	*(6·66, 13·01)*
**British Columbia**	39	16	*23*	*3·28*	*(2·46, 4·37)*	38	17	*21*	*3·08*	*(2·44, 3·89)*	17	5	*13*	*4·38*	*(3·75, 5·11)*	23	3	*20*	*10·22*	*(7·45, 14·03)*
**Ontario**	47	14	*32*	*5·32*	*(4·38, 6·45)*	39	13	*26*	*4·41*	*(3·88, 5·02)*	18	5	*13*	*4·52*	*(4·12, 4·97)*	21	3	*18*	*9·56*	*(7·67, 11·91)*
**Saskatchewan-Manitoba**	63	16	*46*	*8·61*	*(5·13, 14·45)*	40	14	*26*	*4·08*	*(2·94, 5·68)*	18	5	*13*	*4·06*	*(3·28, 5·02)*	29	4	*25*	*8·94*	*(6·21, 12·87)*
**New South Wales**	40	13	*27*	*4·49*	*(3·56, 5·66)*	33	10	*23*	*4·57*	*(3·77, 5·55)*	17	4	*14*	*5·40*	*(4·78, 6·10)*	16	2	*14*	*8·40*	*(6·34, 11·14)*
**Victoria**	46	11	*35*	*6·65*	*(5·02, 8·81)*	38	10	*28*	*5·66*	*(4·54, 7·06)*	17	4	*13*	*4·85*	*(4·20, 5·61)*	19	2	*16*	*9·59*	*(6·94, 13·25)*
**New Zealand**	38	12	*25*	*4·32*	*(3·23, 5·77)*	41	12	*28*	*4·81*	*(3·71, 6·22)*	21	5	*16*	*5·60*	*(4·84, 6·48)*	16	3	*13*	*6·41*	*(4·71, 8·72)*
12-month																				
**Denmark**	77	46	*30*	*3·77*	*(2·97, 4·79)*	64	37	*27*	*3·01*	*(2·50, 3·62)*	35	12	*23*	*4·03*	*(3·67, 4·41)*	35	10	*25*	*5·03*	*(4·24, 5·97)*
**Norway**	74	43	*31*	*3·70*	*(2·88, 4·76)*	65	34	*31*	*3·63*	*(3·05, 4·32)*	36	12	*24*	*4·16*	*(3·83, 4·52)*	36	9	*27*	*5·77*	*(4·87, 6·83)*
**England**	84	50	*34*	*5·29*	*(4·90, 5·72)*	79	48	*31*	*3·96*	*(3·70, 4·24)*	52	20	*32*	*4·27*	*(4·14, 4·41)*	58	14	*44*	*8·75*	*(8·13, 9·42)*
**Northern Ireland**	82	46	*36*	*5·39*	*(3·62, 8·01)*	84	48	*35*	*5·42*	*(3·85, 7·64)*	50	17	*33*	*4·75*	*(4·05, 5·58)*	59	12	*47*	*10·57*	*(7·12, 15·69)*
**Scotland**	82	51	*31*	*4·31*	*(3·56, 5·22)*	75	50	*24*	*2·92*	*(2·43, 3·50)*	51	20	*31*	*4·13*	*(3·77, 4·53)*	49	13	*36*	*6·23*	*(5·08, 7·65)*
**Wales**	80	51	*29*	*3·90*	*(3·00, 5·05)*	79	51	*28*	*3·58*	*(2·85, 4·48)*	51	21	*30*	*3·89*	*(3·47, 4·36)*	57	15	*42*	*7·46*	*(5·86, 9·48)*
**Alberta**	75	51	*24*	*2·87*	*(1·94, 4·25)*	64	46	*18*	*2·07*	*(1·59, 2·70)*	37	15	*23*	*3·47*	*(3·02, 3·99)*	40	11	*30*	*5·70*	*(4·25, 7·64)*
**Atlantic Canada**	67	51	*16*	*1·92*	*(1·30, 2·84)*	69	43	*26*	*2·89*	*(2·21, 3·77)*	30	12	*18*	*3·05*	*(2·66, 3·49)*	52	12	*40*	*7·72*	*(5·85, 10·20)*
**British Columbia**	77	51	*26*	*3·14*	*(2·35, 4·19)*	67	42	*25*	*2·81*	*(2·28, 3·46)*	28	11	*17*	*3·30*	*(2·94, 3·72)*	40	11	*29*	*5·51*	*(4·38, 6·92)*
**Ontario**	78	49	*29*	*3·62*	*(2·95, 4·44)*	66	35	*31*	*3·57*	*(3·18, 4·01)*	33	11	*22*	*3·97*	*(3·71, 4·26)*	41	10	*32*	*6·63*	*(5·66, 7·76)*
**Saskatchewan-Manitoba**	75	58	*17*	*2·14*	*(1·26, 3·65)*	64	42	*22*	*2·45*	*(1·83, 3·27)*	29	12	*18*	*3·13*	*(2·66, 3·68)*	44	12	*32*	*5·79*	*(4·32, 7·76)*
**New South Wales**	74	46	*29*	*3·43*	*(2·75, 4·28)*	63	32	*32*	*3·76*	*(3·21, 4·41)*	33	10	*22*	*4·20*	*(3·85, 4·57)*	39	8	*31*	*7·32*	*(6·06, 8·83)*
**Victoria**	76	43	*32*	*4·06*	*(3·07, 5·37)*	66	35	*31*	*3·58*	*(2·96, 4·33)*	32	11	*21*	*3·74*	*(3·37, 4·16)*	36	8	*28*	*6·37*	*(5·04, 8·04)*
**New Zealand**	78	51	*27*	*3·38*	*(2·59, 4·42)*	68	41	*27*	*3·11*	*(2·52, 3·84)*	38	14	*24*	*3·86*	*(3·49, 4·27)*	35	11	*25*	*4·65*	*(3·79, 5·71)*

B.	Liver					Pancreatic					Lung					Ovarian				
	Mortality					*Mortality*					*Mortality*					*Mortality*				
	EP	***non-EP***	***Diff***	**Crude**	**95% CI**	**EP**	**non-EP**	***Diff***	**Crude**	**95% CI**	**EP**	**non-EP**	***Diff***	**Crude**	**95% CI**	***EP***	***non-EP***	***Diff***	**Crude**	**95% CI**
	(%)	***(%)***	***(%)***	**OR**	**(LCI, UCI)**	***(%)***	***(%)***	***(%)***	**OR**	**(LCI, UCI)**	***(%)***	***(%)***	***(%)***	**OR**	**(LCI, UCI)**	***(%)***	***(%)***	***(%)***	**OR**	**(LCI, UCI)**
1-month																				
**Denmark**	28	5	*23*	*7·49*	*(5·29, 10·59)*	27	10	*17*	*3·37*	*(2·78, 4·07)*	22	4	*18*	*6·12*	*(5·46, 6·85)*	13	2	*12*	*9·68*	(5·89, 15·91)
**Norway**	37	13	*25*	*4·08*	*(3·04, 5·48)*	33	14	*19*	*3·08*	*(2·61, 3·65)*	25	5	*20*	*6·06*	*(5·42, 6·79)*	14	2	*12*	*9·56*	(6·29, 14·53)
**England**	35	9	*26*	*5·63*	*(5·18, 6·12)*	37	13	*24*	*4·04*	*(3·82, 4·28)*	37	8	*29*	*6·66*	*(6·45, 6·86)*	20	3	*17*	*7·01*	(6·29, 7·82)
**Northern Ireland**	40	9	*31*	*6·75*	*(4·30, 10·58)*	43	12	*30*	*5·24*	*(3·92, 7·01)*	35	7	*28*	*6·93*	*(5·94, 8·09)*	19	5	*14*	*4·46*	(2·71, 7·35)
**Scotland**	24	4	*19*	*6·65*	*(4·91, 9·01)*	25	10	*15*	*3·05*	*(2·46, 3·77)*	25	6	*19*	*5·45*	*(4·95, 6·00)*	14	4	*10*	*4·20*	(2·96, 5·95)
**Wales**	28	7	*21*	*5·21*	*(3·60, 7·53)*	29	13	*16*	*2·78*	*(2·19, 3·53)*	30	6	*24*	*6·66*	*(5·84, 7·59)*	14	3	*11*	*5·93*	(3·73, 9·43)
**Alberta**	34	7	*27*	*6·92*	*(4·78, 10·00)*	29	9	*20*	*4·04*	*(3·08, 5·31)*	25	7	*19*	*4·84*	*(4·19, 5·59)*	8	3	*5*	*2·56*	(1·36, 4·85)
**Atlantic Canada**	35	12	*23*	*4·05*	*(2·73, 6·01)*	34	13	*21*	*3·34*	*(2·57, 4·33)*	30	7	*23*	*5·44*	*(4·80, 6·17)*	26	3	*23*	*10·24*	(5·94, 17·63)
**British Columbia**	35	8	*27*	*5·94*	*(4·55, 7·75)*	30	11	*19*	*3·42*	*(2·79, 4·20)*	31	6	*24*	*6·73*	*(6·02, 7·53)*	16	3	*13*	*6·22*	(4·08, 9·48)
**Ontario**	16	4	*12*	*4·55*	*(3·58, 5·79)*	18	6	*12*	*3·55*	*(3·00, 4·21)*	20	4	*16*	*5·83*	*(5·39, 6·31)*	14	2	*13*	*10·31*	(7·60, 13·99)
**Saskatchewan-Manitoba**	33	11	*23*	*4·25*	*(2·74, 6·60)*	33	10	*24*	*4·69*	*(3·47, 6·32)*	30	7	*23*	*5·71*	*(4·91, 6·64)*	18	4	*14*	*5·44*	(3·10, 9·56)
**New South Wales**	27	5	*22*	*7·09*	*(5·57, 9·03)*	20	6	*14*	*3·99*	*(3·27, 4·86)*	20	4	*16*	*6·46*	*(5·73, 7·28)*	14	2	*13*	*10·11*	(6·52, 15·67)
**Victoria**	23	6	*17*	*4·91*	*(3·73, 6·46)*	26	7	*19*	*4·68*	*(3·76, 5·82)*	22	5	*17*	*5·65*	*(4·92, 6·48)*	14	2	*12*	*8·17*	(5·04, 13·23)
**New Zealand**	23	4	*20*	*8·33*	*(5·23, 13·25)*	22	6	*16*	*4·08*	*(3·02, 5·51)*	22	4	*18*	*7·11*	*(5·96, 8·48)*	11	1	*9*	*7·79*	(4·09, 14·82)
3-month																				
**Denmark**	56	19	*37*	*5·55*	*(4·41, 6·98)*	54	26	*28*	*3·28*	*(2·85, 3·78)*	43	15	*27*	*4·13*	*(3·83, 4·45)*	26	5	*21*	*6·73*	(4·87, 9·30)
**Norway**	55	22	*33*	*4·42*	*(3·43, 5·70)*	57	27	*30*	*3·57*	*(3·11, 4·10)*	46	13	*33*	*5·75*	*(5·30, 6·24)*	23	5	*18*	*5·31*	(4·09, 6·91)
**England**	61	23	*38*	*5·31*	*(4·97, 5·66)*	65	35	*31*	*3·55*	*(3·38, 3·72)*	63	22	*41*	*6·16*	*(6·01, 6·31)*	37	8	*28*	*6·26*	(5·79, 6·76)
**Northern Ireland**	71	25	*46*	*7·27*	*(4·99, 10·60)*	74	32	*42*	*5·97*	*(4·61, 7·73)*	66	22	*45*	*7·21*	*(6·38, 8·16)*	40	12	*28*	*5·07*	(3·53, 7·27)
**Scotland**	52	16	*36*	*5·72*	*(4·72, 6·94)*	56	32	*24*	*2·72*	*(2·34, 3·17)*	53	20	*32*	*4·38*	*(4·11, 4·68)*	31	10	*22*	*4·19*	(3·31, 5·29)
**Wales**	59	22	*37*	*5·18*	*(4·00, 6·71)*	57	35	*22*	*2·47*	*(2·05, 2·97)*	57	20	*38*	*5·51*	*(5·03, 6·05)*	27	8	*20*	*4·44*	(3·30, 5·96)
**Alberta**	57	19	*38*	*5·69*	*(4·27, 7·57)*	58	28	*30*	*3·47*	*(2·83, 4·27)*	51	18	*33*	*4·76*	*(4·28, 5·30)*	24	7	*18*	*4·50*	(2·96, 6·86)
**Atlantic Canada**	65	29	*37*	*4·69*	*(3·34, 6·57)*	59	34	*24*	*2·73*	*(2·20, 3·40)*	56	19	*37*	*5·40*	*(4·88, 5·97)*	39	10	*29*	*5·89*	(4·02, 8·65)
**British Columbia**	61	23	*38*	*5·10*	*(4·14, 6·29)*	59	32	*27*	*3·10*	*(2·64, 3·64)*	57	18	*38*	*5·75*	*(5·29, 6·25)*	30	4	*26*	*10·29*	(7·20, 14·69)
**Ontario**	42	13	*29*	*4·72*	*(4·03, 5·52)*	42	23	*18*	*2·35*	*(2·11, 2·62)*	48	15	*32*	*5·07*	*(4·81, 5·34)*	29	5	*23*	*7·44*	(6·13, 9·02)
**Saskatchewan-Manitoba**	52	29	*23*	*2·70*	*(1·89, 3·86)*	62	35	*27*	*3·09*	*(2·45, 3·90)*	54	21	*34*	*4·60*	*(4·10, 5·16)*	33	4	*29*	*12·00*	(7·10, 20·27)
**New South Wales**	50	14	*36*	*6·20*	*(5·22, 7·35)*	46	22	*24*	*3·06*	*(2·69, 3·48)*	44	14	*30*	*4·86*	*(4·51, 5·25)*	24	4	*20*	*7·73*	(5·70, 10·48)
**Victoria**	42	15	*28*	*4·31*	*(3·52, 5·27)*	44	22	*22*	*2·83*	*(2·42, 3·32)*	45	14	*31*	*5·03*	*(4·56, 5·54)*	24	4	*20*	*7·04*	(4·93, 10·04)
**New Zealand**	50	12	*38*	*7·06*	*(5·31, 9·38)*	50	28	*22*	*2·53*	*(2·11, 3·04)*	47	14	*34*	*5·62*	*(5·05, 6·25)*	22	5	*17*	*5·70*	(3·88, 8·37)
12-month																				
**Denmark**	81	48	*33*	*4·59*	*(3·63, 5·80)*	81	60	*21*	*2·85*	*(2·44, 3·33)*	71	43	*27*	*3·15*	*(2·94, 3·38)*	47	15	*32*	*5·10*	(4·00, 6·50)
**Norway**	75	43	*32*	*3·98*	*(3·10, 5·10)*	84	57	*27*	*4·03*	*(3·45, 4·69)*	77	39	*38*	*5·33*	*(4·95, 5·75)*	37	11	*25*	*4·56*	(3·73, 5·58)
**England**	84	52	*32*	*4·85*	*(4·51, 5·22)*	89	69	*20*	*3·59*	*(3·37, 3·81)*	86	52	*34*	*5·70*	*(5·54, 5·87)*	54	19	*35*	*4·95*	(4·65, 5·28)
**Northern Ireland**	87	53	*34*	*6·14*	*(3·91, 9·66)*	93	68	*26*	*6·69*	*(4·53, 9·87)*	90	56	*34*	*7·21*	*(6·11, 8·50)*	56	26	*30*	*3·59*	(2·62, 4·91)
**Scotland**	80	44	*36*	*5·20*	*(4·32, 6·26)*	83	69	*14*	*2·20*	*(1·85, 2·62)*	81	52	*29*	*3·84*	*(3·59, 4·11)*	49	21	*28*	*3·58*	(2·96, 4·33)
**Wales**	84	53	*31*	*4·65*	*(3·53, 6·12)*	83	68	*15*	*2·33*	*(1·88, 2·88)*	83	53	*30*	*4·31*	*(3·90, 4·76)*	40	16	*25*	*3·67*	(2·89, 4·65)
**Alberta**	80	38	*41*	*6·32*	*(4·66, 8·59)*	85	64	*21*	*3·13*	*(2·45, 4·01)*	78	45	*33*	*4·24*	*(3·80, 4·73)*	47	20	*27*	*3·50*	(2·56, 4·80)
**Atlantic Canada**	83	52	*31*	*4·57*	*(3·09, 6·74)*	88	72	*15*	*2·69*	*(2·01, 3·60)*	82	47	*35*	*5·10*	*(4·55, 5·71)*	63	23	*39*	*5·48*	(3·93, 7·63)
**British Columbia**	85	53	*32*	*5·18*	*(4·05, 6·61)*	86	68	*18*	*2·89*	*(2·36, 3·54)*	82	45	*37*	*5·64*	*(5·15, 6·18)*	47	14	*33*	*5·52*	(4·33, 7·05)
**Ontario**	72	38	*34*	*4·15*	*(3·58, 4·81)*	74	58	*17*	*2·11*	*(1·88, 2·36)*	76	42	*35*	*4·50*	*(4·27, 4·75)*	48	15	*33*	*5·33*	(4·61, 6·18)
**Saskatchewan-Manitoba**	77	55	*22*	*2·75*	*(1·86, 4·06)*	84	69	*16*	*2·45*	*(1·84, 3·26)*	82	46	*36*	*5·43*	*(4·76, 6·18)*	45	12	*33*	*6·00*	(4·12, 8·74)
**New South Wales**	75	38	*38*	*5·02*	*(4·27, 5·90)*	77	57	*20*	*2·58*	*(2·26, 2·94)*	74	41	*32*	*3·94*	*(3·68, 4·23)*	42	14	*28*	*4·46*	(3·61, 5·50)
**Victoria**	72	36	*36*	*4·51*	*(3·73, 5·46)*	76	54	*22*	*2·73*	*(2·32, 3·22)*	74	41	*33*	*4·17*	*(3·80, 4·59)*	37	12	*26*	*4·43*	(3·39, 5·79)
**New Zealand**	76	41	*35*	*4·52*	*(3·54, 5·76)*	83	69	*14*	*2·21*	*(1·81, 2·72)*	77	45	*32*	*4·13*	*(3·76, 4·53)*	42	17	*25*	*3·52*	(2·75, 4·50)

EP: Emergency presentation; OR: Odds ratio; CI: Confidence Interval; LCI: Lower Confidence Interval; UCI: Upper Confidence Interval

Supplementary Material 10. 12-month mortality by emergency presentation (defined as diagnosis of cancer within 30 days after an emergency hospital admission) status and other variables; odds ratios from crude and adjusted (with/without adjustment for stage) models

Denmark		All patients	12-month mortality			Crude odds ratios				Adjusted odds ratios (stage unadjusted)				Adjusted odds ratios (stage adjusted)			
			Mort	%Mort	95% CI	Crude OR	Crude OR (LCI)	Crude OR (UCI)	p(COR)	Adj OR	Adj OR (LCI)	Adj OR (UCI)	p(AOR)	Adj OR	Adj OR (LCI)	Adj OR (UCI)	p(AOR)
	**Total**	46,003	17,610	38·3%	(37·8%, 38·7%)												
**Presentation status**	**Elective**	31,804	9,013	28·3%	(27·8%, 28·8%)	(ref)	(ref)	(ref)	<0·0001	(ref)	(ref)	(ref)	<0·0001	(ref)	(ref)	(ref)	<0·0001
	**Emergency**	14,199	8,597	60·5%	(59·7%, 61·3%)	3·88	3·72	4·05		3·28	3·13	3·43		2·62	2·49	2·76	
**Sex**	**Male**	24,016	9,597	40·0%	(39·3%, 40·6%)	(ref)	(ref)	(ref)	<0·0001	(ref)	(ref)	(ref)	<0·0001	(ref)	(ref)	(ref)	<0·0001
	**Female**	21,987	8,013	36·4%	(35·8%, 37·1%)	0·86	0·83	0·89		0·82	0·78	0·86		0·81	0·77	0·85	
**Diagnosis year**	**2014**	11,354	4,581	40·3%	(39·4%, 41·3%)	(ref)	(ref)	(ref)	<0·0001	(ref)	(ref)	(ref)	<0·0001	(ref)	(ref)	(ref)	<0·0001
	**2015**	11,423	4,371	38·3%	(37·4%, 39·2%)	0·92	0·87	0·97		0·95	0·89	1·01		0·95	0·89	1·01	
	**2016**	11,466	4,337	37·8%	(36·9%, 38·7%)	0·90	0·85	0·95		0·90	0·85	0·96		0·92	0·86	0·98	
	**2017**	11,760	4,321	36·7%	(35·9%, 37·6%)	0·86	0·81	0·91		0·87	0·82	0·93		0·87	0·81	0·93	
**Age group**	**15-64**	13,131	3,817	29·1%	(28·3%, 29·9%)	0·76	0·72	0·79	<0·0001	0·75	0·71	0·79	<0·0001	0·68	0·64	0·72	<0·0001
	**65-74**	17,336	10,303	35·4%	(34·9%, 36·0%)	(ref)	(ref)	(ref)		(ref)	(ref)	(ref)		(ref)	(ref)	(ref)	
	**75-84**	11,995	5,509	45·9%	(45·0%, 46·8%)	1·57	1·49	1·64		1·67	1·58	1·77		1·89	1·78	2·01	
	**85+**	3,541	2,186	61·7%	(60·1%, 63·3%)	2·97	2·76	3·20		4·17	3·82	4·55		4·81	4·37	5·29	
**Stage**	**1**	5,977	434	7·3%	(6·6%, 7·9%)	(ref)	(ref)	(ref)	<0·0001					(ref)	(ref)	(ref)	<0·0001
	**2**	6,064	851	14·0%	(13·2%, 14·9%)	2·09	1·86	2·36						2·01	1·76	2·28	
	**3**	7,231	1,948	26·9%	(25·9%, 28·0%)	4·48	4·02	4·99						4·34	3·86	4·88	
	**4**	15,359	10,315	67·2%	(66·4%, 67·9%)	24·82	22·45	27·50						21·63	19·41	24·15	
	**Unknown**	11,372	4,001	35·2%	(34·3%, 36·1%)	6·59	5·95	7·31						7·78	6·95	8·73	
**Cancer site**	**Oesophageal**	1,896	1,019	53·7%	(51·5%, 56·0%)	5·09	4·61	5·63	<0·0001	7·07	6·35	7·88	<0·0001	6·67	5·94	7·49	<0·0001
	**Stomach**	2,231	1,024	45·9%	(43·8%, 48·0%)	3·72	3·38	4·09		4·38	3·96	4·85		4·20	3·76	4·69	
	**Colon**	12,928	2,401	18·6%	(17·9%, 19·3%)	(ref)	(ref)	(ref)		(ref)	(ref)	(ref)		(ref)	(ref)	(ref)	
	**Rectal**	6,005	790	13·2%	(12·3%, 14·0%)	0·66	0·61	0·72		0·86	0·78	0·94		0·84	0·76	0·92	
	**Liver**	1,541	958	62·2%	(59·7%, 64·6%)	7·20	6·44	8·06		8·29	7·35	9·35		10·69	9·36	12·22	
	**Pancreatic**	3,491	2,458	70·4%	(68·9%, 71·9%)	10·43	9·58	11·36		11·79	10·76	12·92		10·03	9·09	11·09	
	**Lung**	16,003	8,547	53·4%	(52·6%, 54·2%)	5·03	4·76	5·31		6·14	5·79	6·51		5·30	4·95	5·67	
	**Ovarian**	1,908	413	21·6%	(19·9%, 23·5%)	1·21	1·08	1·36		1·79	1·57	2·03		1·28	1·12	1·47	

Norway		All patients	12-month mortality			Crude odds ratios				Adjusted odds ratios (stage unadjusted)				Adjusted odds ratios (stage adjusted)			
			Mort	%Mort	95% CI	Crude OR	Crude OR (LCI)	Crude OR (UCI)	p(COR)	Adj OR	Adj OR (LCI)	Adj OR (UCI)	p(AOR)	Adj OR	Adj OR (LCI)	Adj OR (UCI)	p(AOR)
	**Total**	46,147	17,329	37·6%	(37·1%, 38%)												
**Presentation status**	**Elective**	29,281	7,222	24·7%	(24·2%, 25·2%)	(ref)	(ref)	(ref)	<0·0001	(ref)	(ref)	(ref)	<0·0001	(ref)	(ref)	(ref)	<0·0001
	**Emergency**	16,866	10,107	59·9%	(59·2%, 60·7%)	4·57	4·39	4·76		4·37	4·17	4·58		3·31	3·15	3·49	
**Sex**	**Male**	23,319	9,423	40·4%	(39·8%, 41%)	(ref)	(ref)	(ref)	<0·0001	(ref)	(ref)	(ref)	<0·0001	(ref)	(ref)	(ref)	<0·0001
	**Female**	22,828	7,906	34·6%	(34%, 35·3%)	0·78	0·75	0·81		0·78	0·75	0·82		0·77	0·73	0·81	
**Diagnosis year**	**2012**	8,962	3,688	41·2%	(40·1%, 42·2%)	(ref)	(ref)	(ref)	<0·0001	(ref)	(ref)	(ref)	<0·0001	(ref)	(ref)	(ref)	<0·0001
	**2013**	9,060	3,431	37·9%	(36·9%, 38·9%)	0·87	0·82	0·93		0·89	0·82	0·95		0·86	0·79	0·93	
	**2014**	9,293	3,502	37·7%	(36·7%, 38·7%)	0·86	0·81	0·92		0·87	0·81	0·94		0·84	0·78	0·91	
	**2015**	9,561	3,508	36·7%	(35·7%, 37·7%)	0·83	0·78	0·88		0·82	0·76	0·88		0·78	0·72	0·84	
	**2016**	9,271	3,200	34·5%	(33·6%, 35·5%)	0·75	0·71	0·80		0·78	0·72	0·83		0·72	0·67	0·78	
**Age group**	**15-64**	13,277	3,511	26·4%	(25·7%, 27·2%)	0·66	0·63	0·69	<0·0001	0·68	0·64	0·72	<0·0001	0·60	0·56	0·64	<0·0001
	**65-74**	15,278	5,394	35·3%	(34·6%, 36·1%)	(ref)	(ref)	(ref)		(ref)	(ref)	(ref)		(ref)	(ref)	(ref)	
	**75-84**	12,424	5,461	44·0%	(43·1%, 44·8%)	1·44	1·37	1·51		1·71	1·61	1·81		1·97	1·85	2·10	
	**85+**	5,168	2,963	57·3%	(56·0%, 58·7%)	2·46	2·31	2·63		3·28	3·04	3·54		3·91	3·59	4·26	
**Stage**	**Local**	8,515	957	11·2%	(10·6%, 11·9%)	(ref)	(ref)	(ref)	<0·0001					(ref)	(ref)	(ref)	<0·0001
	**Regional**	16,460	3,285	20·0%	(19·4%, 20·6%)	1·97	1·82	2·13						2·23	2·04	2·43	
	**Distant**	15,613	9,826	62·9%	(62·2%, 63·7%)	13·41	12·45	14·46						15·16	13·9	16·54	
	**Unknown**	5,559	3,261	58·7%	(57·4%, 59·9%)	11·21	10·29	12·22						7·81	7·09	8·61	
**Cancer site**	**Oesophageal**	1,332	705	52·9%	(50·2%, 55·6%)	4·27	3·81	4·79	<0·0001	6·29	5·55	7·14	<0·0001	5·47	4·76	6·28	<0·0001
	**Stomach**	2,357	1,094	46·4%	(44·4%, 48·4%)	3·29	3.00	3·60		3·92	3·54	4·33		3·29	2·95	3·67	
	**Colon**	14,941	3,116	20·9%	(20·2%, 21·5%)	(ref)	(ref)	(ref)		(ref)	(ref)	(ref)		(ref)	(ref)	(ref)	
	**Rectal**	5,612	752	13·4%	(12·5%, 14·3%)	0·59	0·54	0·64		0·88	0·80	0·97		0·89	0·81	0·98	
	**Liver**	1,181	704	59·6%	(56·8%, 62·4%)	5·60	4·96	6·34		6·87	6.00	7·86		8·03	6·89	9·38	
	**Pancreatic**	3,715	2,668	71·8%	(70·3%, 73·2%)	9·67	8·91	10·50		11·39	10·41	12·46		8·4	7·62	9·27	
	**Lung**	14,402	7,765	53·9%	(53·1%, 54·7%)	4·44	4·22	4·67		6·18	5·83	6·55		5·38	5·05	5·74	
	**Ovarian**	2,607	525	20·1%	(18·6%, 21·7%)	0·96	0·86	1·06		1·39	1·24	1·56		0·56	0·49	0·63	

England		All patients	12-month mortality			Crude odds ratios				Adjusted odds ratios (stage unadjusted)				Adjusted odds ratios (stage adjusted)			
			Mort	%Mort	95% CI	Crude OR	Crude OR (LCI)	Crude OR (UCI)	p(COR)	Adj OR	Adj OR (LCI)	Adj OR (UCI)	p(AOR)	Adj OR	Adj OR (LCI)	Adj OR (UCI)	p(AOR)
	**Total**	373,194	187,698	50·3%	(50·1%, 50·5%)												
**Presentation status**	**Elective**	256,260	99,437	38·8%	(36·9%, 37·3%)	(ref)	(ref)	(ref)	<0·0001	(ref)	(ref)	(ref)	<0·0001	(ref)	(ref)	(ref)	<0·0001
	**Emergency**	116,934	88,261	75·5%	(72·5%, 73·0%)	4·85	4·78	4·93		4·54	4·46	4·62		3·46	3·39	3·53	
**Sex**	**Male**	197,003	101,355	51·4%	(51·2%, 51·7%)	(ref)	(ref)	(ref)	<0·0001	(ref)	(ref)	(ref)	<0·0001	(ref)	(ref)	(ref)	<0·0001
	**Female**	176,191	86,343	49·0%	(48·8%, 49·2%)	0·91	0·90	0·92		0·86	0·85	0·87		0·88	0·86	0·89	
**Diagnosis year**	**2013**	90,993	45,991	50·5%	(50·2%, 50·9%)	(ref)	(ref)	(ref)	0·0098	(ref)	(ref)	(ref)	<0·0001	(ref)	(ref)	(ref)	0·0010
	**2014**	92,609	46,859	50·6%	(50·3%, 50·9%)	1.00	0·98	1·02		1.00	0·98	1·02		1·04	1·02	1·07	
	**2015**	94,296	47,232	50·1%	(49·8%, 50·4%)	0·98	0·96	1.00		0·98	0·96	1.00		1·04	1·02	1·07	
	**2016**	95,296	47,616	50·0%	(49·6%, 50·3%)	0·98	0·96	1.00		0·98	0·96	1.00		1·05	1·03	1·08	
**Age group**	**15-64**	98,419	35,732	36·3%	(36%, 36·6%)	0·66	0·65	0·67	<0·0001	0·70	0·68	0·71	<0·0001	0·62	0·60	0·63	<0·0001
	**65-74**	112,970	52,332	46·3%	(46·0%, 46·6%)	(ref)	(ref)	(ref)		(ref)	(ref)	(ref)		(ref)	(ref)	(ref)	
	**75-84**	110,521	62,784	56·8%	(56·5%, 57·1%)	1·52	1·50	1·55		1·54	1·51	1·57		1·79	1·75	1·83	
	**85+**	51,284	36,850	71·9%	(71·5%, 72·2%)	2·96	2·89	3·03		3·02	2·94	3·09		3·63	3·53	3·74	
**Stage**	**1**	48,445	6,141	12·7%	(12·4%, 13·0%)	(ref)	(ref)	(ref)	<0·0001					(ref)	(ref)	(ref)	<0·0001
	**2**	49,375	10,182	20·6%	(20·3%, 21·0%)	1·79	1·73	1·85						2·16	2·08	2·25	
	**3**	80,853	28,657	35·4%	(35·1%, 35·8%)	3·78	3·67	3·90						4·93	4·76	5·10	
	**4**	136,382	105,420	77·3%	(77·1%, 77·5%)	23·46	22·77	24·16						23·33	22·57	24·12	
	**Unknown**	58,139	37,298	64·2%	(63·8%, 64·5%)	12·33	11·94	12·73						9·47	9·13	9·82	
**Cancer site**	**Oesophageal**	27,274	15,358	56·3%	(55·7%, 56·9%)	3·09	3.00	3·18	<0·0001	4·48	4·34	4·62	<0·0001	4·37	4·22	4·52	<0·0001
	**Stomach**	19,813	11,405	57·6%	(56·9%, 58·2%)	3·25	3·15	3·36		3·71	3·58	3·84		3·14	3·02	3·26	
	**Colon**	88,219	25,975	29·4%	(29·1%, 29·7%)	(ref)	(ref)	(ref)		(ref)	(ref)	(ref)		(ref)	(ref)	(ref)	
	**Rectal**	33,905	6,241	18·4%	(18·0%, 18·8%)	0·54	0·52	0·56		0·78	0·75	0·80		0·82	0·79	0·85	
	**Liver**	17,699	11,641	65·8%	(65·1%, 66·5%)	4·60	4·45	4·77		5·07	4·88	5·26		3·90	3·74	4·06	
	**Pancreatic**	30,442	23,782	78·1%	(77·7%, 78·6%)	8·56	8·30	8·82		9·51	9·20	9·83		7·24	6·98	7·51	
	**Lung**	135,396	86,848	64·1%	(63·9%, 64·4%)	4·29	4·21	4·37		5·20	5·10	5·31		4·57	4·46	4·67	
	**Ovarian**	20,446	6,448	31·5%	(30·9%, 32·2%)	1·10	1·07	1·14		1·29	1·24	1·34		1·23	1·18	1·28	

Northern Ireland		All patients	12-month mortality			Crude odds ratios				Adjusted odds ratios (stage unadjusted)				Adjusted odds ratios (stage adjusted)			
			Mort	%Mort	95% CI	Crude OR	Crude OR (LCI)	Crude OR (UCI)	p(COR)	Adj OR	Adj OR (LCI)	Adj OR (UCI)	p(AOR)	Adj OR	Adj OR (LCI)	Adj OR (UCI)	p(AOR)
	**Total**	15,501	7,708	49·7%	(48·9%, 50·5%)												
**Presentation status**	**Elective**	11,179	4,328	38·7%	(37·8%, 39·6%)	(ref)	(ref)	(ref)	<0·0001	(ref)	(ref)	(ref)	<0·0001	(ref)	(ref)	(ref)	<0·0001
	**Emergency**	4,322	3,380	78·2%	(76·9%, 79·4%)	5·68	5·24	6·17		5·46	4·98	5·99		3·53	3·19	3·92	
**Sex**	**Male**	8,134	4,028	49·5%	(48·4%, 50·6%)	(ref)	(ref)	(ref)	0·59	(ref)	(ref)	(ref)	0·19	(ref)	(ref)	(ref)	0·97
	**Female**	7,367	3,680	50·0%	(48·8%, 51·1%)	1·02	0·96	1·08		0·95	0·88	1·03		1.00	0·92	1·10	
**Diagnosis year**	**2012**	3,071	1,547	50·4%	(48·6%, 52·1%)	1·04	0·94	1·15	0·83	1·03	0·91	1·16	0·052	0·95	0·83	1·09	0·73
	**2013**	3,125	1,544	49·4%	(47·7%, 51·2%)	(ref)	(ref)	(ref)		(ref)	(ref)	(ref)		(ref)	(ref)	(ref)	
	**2014**	3,051	1,494	49·0%	(47·2%, 50·7%)	0·98	0·89	1·09		0·92	0·82	1·04		0·94	0·82	1·07	
	**2015**	3,072	1,533	49·9%	(48·1%, 51·7%)	1·02	0·92	1·13		0·94	0·83	1·05		0·93	0·81	1·07	
	**2016**	3,182	1,590	50·0%	(48·2%, 51·7%)	1·02	0·93	1·13		0·94	0·84	1·06		1·01	0·88	1·15	
**Age group**	**15-64**	4,432	1,640	37·0%	(35·6%, 38·4%)	0·67	0·62	0·73	<0·0001	0·72	0·65	0·79	<0·0001	0·62	0·55	0·69	<0·0001
	**65-74**	4,965	2,319	46·7%	(45·3%, 48·1%)	(ref)	(ref)	(ref)		(ref)	(ref)	(ref)		(ref)	(ref)	(ref)	
	**75-84**	4,408	2,549	57·8%	(56·4%, 59·3%)	1·56	1·44	1·70		1·70	1·54	1·87		1·97	1·76	2·20	
	**85+**	1,696	1,200	70·8%	(68·5%, 72·9%)	2·76	2·45	3·11		3·19	2·77	3·67		3·57	3·05	4·19	
**Stage**	**1**	2,221	290	13·1%	(11·7%, 14·5%)	(ref)	(ref)	(ref)	<0·0001					(ref)	(ref)	(ref)	<0·0001
	**2**	2,234	351	15·7%	(14·3%, 17·3%)	1·24	1·05	1·47						1·72	1·43	2·08	
	**3**	3,584	1,330	37·1%	(35·5%, 38·7%)	3·93	3·42	4·53						4·87	4·16	5·71	
	**4**	5,184	4,132	79·7%	(78·6%, 80·8%)	26·15	22·75	30·15						26·71	22·75	31·44	
	**Unknown**	2,278	1,605	70·5%	(68·5%, 72·3%)	15·88	13·65	18·53						10·81	9·07	12·92	
**Cancer site**	**Oesophageal**	972	521	53·6%	(50·5%, 56·7%)	3·47	3.00	4·01	<0·0001	5·04	4·29	5·91	<0·0001	3·90	3·26	4·68	<0·0001
	**Stomach**	990	583	58·9%	(55·8%, 61·9%)	4·30	3·72	4·98		4·91	4·19	5·77		3·23	2·70	3·87	
	**Colon**	3,846	961	25·0%	(23·6%, 26·4%)	(ref)	(ref)	(ref)		(ref)	(ref)	(ref)		(ref)	(ref)	(ref)	
	**Rectal**	1,376	225	16·4%	(14·5%, 18·4%)	0·59	0·50	0·69		0·84	0·71	1.00		0·91	0·75	1·10	
	**Liver**	582	383	65·8%	(61·9%, 69·5%)	5·78	4·80	6·97		6·46	5·27	7·93		6·18	4·90	7·83	
	**Pancreatic**	1,162	914	78·7%	(76·2%, 80·9%)	11·06	9·46	12·98		12·49	10·54	14·85		9·80	8·08	11·91	
	**Lung**	5,752	3,831	66·6%	(65·4%, 67·8%)	5·99	5·47	6·56		7·48	6·76	8·29		7·17	6·38	8·07	
	**Ovarian**	821	290	35·3%	(32·1%, 38·7%)	1·64	1·40	1·92		1·85	1·54	2·23		2·16	1·76	2·65	

Scotland		All patients	12-month mortality Mort	%Mort	95% CI	Crude odds ratios Crude OR	Crude OR (LCI)	Crude OR (UCI)	p(COR)	Adjusted odds ratios (stage unadjusted) Adj OR	Adj OR (LCI)	Adj OR (UCI)	p(AOR)	Adjusted odds ratios (stage adjusted) Adj OR	Adj OR (LCI)	Adj OR (UCI)	p(AOR)
	**Total**	45,029	23,417	52·0%	(51·5%, 52·5%)												
**Presentation status**	**Elective**	27,708	10,910	39·4%	(38·8%, 40·0%)	(ref)	(ref)	(ref)	<0·0001	(ref)	(ref)	(ref)	<0·0001	(ref)	(ref)	(ref)	<0·0001
	**Emergency**	17,321	12,507	72·2%	(71·5%, 72·9%)	4.00	3·84	4·17		3·60	3·44	3·77		2·95	2·80	3·11	
**Sex**	**Male**	23,042	12,279	53·3%	(52·6%, 53·9%)	1·11	1·07	1·15	<0·0001	1·19	1·14	1·25	<0·0001	1·17	1·11	1·23	<0·0001
	**Female**	21,987	11,138	50·7%	(50·0%, 51·3%)	(ref)	(ref)	(ref)		(ref)	(ref)	(ref)		(ref)	(ref)	(ref)	
**Diagnosis year**	**2014**	11,599	6,077	52·4%	(51·5%, 53·3%)	(ref)	(ref)	(ref)	0·36	(ref)	(ref)	(ref)	0·43	(ref)	(ref)	(ref)	0·27
	**2015**	11,091	5,708	51·5%	(50·5%, 52·4%)	0·96	0·91	1·02		0·95	0·9	1·01		0·94	0·88	1·01	
	**2016**	11,064	5,721	51·7%	(50·8%, 52·6%)	0·97	0·92	1·03		0·99	0·93	1·05		0·98	0·92	1·05	
	**2017**	11,275	5,911	52·4%	(51·5%, 53·3%)	1.00	0·95	1·05		1·02	0·96	1·08		1·02	0·96	1·10	
**Age group**	**15-64**	12,024	4,686	39·0%	(38·1%, 39·8%)	0·66	0·63	0·70	<0·0001	0·70	0·67	0·74	<0·0001	0·61	0·57	0·65	<0·0001
	**65-74**	14,233	6,986	49·1%	(48·3%, 49·9%)	(ref)	(ref)	(ref)		(ref)	(ref)	(ref)		(ref)	(ref)	(ref)	
	**75-84**	13,399	7,830	58·4%	(57·6%, 59·3%)	1·46	1·39	1·53		1·47	1·40	1·55		1·72	1·62	1·83	
	**85+**	5,373	3,915	72·9%	(71·7%, 74·0%)	2·79	2·60	2·98		2·88	2·66	3·11		3·46	3·17	3·77	
**Stage**	**1**	5,858	740	12·6%	(11·8%, 13·5%)	(ref)	(ref)	(ref)	<0·0001					(ref)	(ref)	(ref)	<0·0001
	**2**	5,857	1,113	19·0%	(18·0%, 20·0%)	1·62	1·47	1·80						2·23	2.00	2·50	
	**3**	9,180	3,542	38·6%	(37·6%, 39·6%)	4·35	3·98	4·75						5·85	5·31	6·45	
	**4**	17,250	13,918	80·7%	(80·1%, 81·3%)	28·89	26·53	31·5						31·94	29·06	35·15	
	**Unknown**	6,884	4,104	59·6%	(58·5%, 60·8%)	10·21	9·33	11·19						9·09	8·21	10·08	
**Cancer site**	**Oesophageal**	3,378	1,985	58·8%	(57·1%, 60·4%)	3·22	2·97	3·49	<0·0001	4·37	4·01	4·77	<0·0001	3·97	3·60	4·38	<0·0001
	**Stomach**	2,337	1,398	59·8%	(57·8%, 61·8%)	3·36	3·06	3·69		3·70	3·34	4·09		2·77	2·48	3·10	
	**Colon**	9,600	2,948	30·7%	(29·8%, 31·6%)	(ref)	(ref)	(ref)		(ref)	(ref)	(ref)		(ref)	(ref)	(ref)	
	**Rectal**	3,535	648	18·3%	(17·1%, 19·6%)	0·51	0·46	0·56		0·71	0·64	0·79		0·75	0·67	0·84	
	**Liver**	2,340	1,432	61·2%	(59·2%, 63·2%)	3·56	3·24	3·91		3·73	3·37	4·12		3·81	3·39	4·28	
	**Pancreatic**	3,054	2,373	77·7%	(76·2%, 79·1%)	7·86	7·15	8·66		7·67	6·93	8·50		6·22	5·55	6·98	
	**Lung**	18,662	11,924	63·9%	(63·2%, 64·6%)	3·99	3·79	4·21		4·55	4·29	4·81		4·04	3·78	4·32	
	**Ovarian**	2,123	709	33·4%	(31·4%, 35·4%)	1·13	1·02	1·25		1·26	1·13	1·40		1·19	1·05	1·35	

Wales		All patients	12-month mortality			Crude odds ratios				Adjusted odds ratios (stage unadjusted)				Adjusted odds ratios (stage adjusted)			
			Mort	%Mort	95% CI	Crude OR	Crude OR (LCI)	Crude OR (UCI)	p(COR)	Adj OR	Adj OR (LCI)	Adj OR (UCI)	p(AOR)	Adj OR	Adj OR (LCI)	Adj OR (UCI)	p(AOR)
	**Total**	26,139	13,270	50·8%	(50·2%, 51·4%)												
**Presentation status**	**Elective**	16,376	6,253	38·2%	(37·4%, 38·9%)	(ref)	(ref)	(ref)	<0·0001	(ref)	(ref)	(ref)	<0·0001	(ref)	(ref)	(ref)	<0·0001
	**Emergency**	9,763	7,017	71·9%	(71·0%, 72·8%)	4·14	3·92	4·37		3·79	3·57	4·03		2·99	2·79	3·19	
**Sex**	**Male**	14,017	7,338	52·4%	(51·5%, 53·2%)	(ref)	(ref)	(ref)	<0·0001	(ref)	(ref)	(ref)	<0·0001	(ref)	(ref)	(ref)	0·0034
	**Female**	12,122	5,932	48·9%	(48·0%, 49·8%)	0·87	0·83	0·92		0·87	0·82	0·92		0·91	0·85	0·97	
**Diagnosis year**	**2013**	6,537	3,409	52·1%	(50·9%, 53·4%)	(ref)	(ref)	(ref)	0·060	(ref)	(ref)	(ref)	0·013	(ref)	(ref)	(ref)	0·11
	**2014**	6,586	3,328	50·5%	(49·3%, 51·7%)	0·94	0·88	1.00		0·94	0·87	1·01		0·91	0·83	0·99	
	**2015**	6,545	3,306	50·5%	(49·3%, 51·7%)	0·94	0·87	1.00		0·91	0·84	0·98		0·92	0·84	1·01	
	**2016**	6,471	3,227	49·9%	(48·7%, 51·1%)	0·91	0·85	0·98		0·90	0·83	0·97		0·92	0·84	1.00	
**Age group**	**15-64**	6,645	2,507	37·7%	(36·6%, 38·9%)	0·69	0·64	0·73	<0·0001	0·73	0·68	0·79	<0·0001	0·63	0·58	0·68	<0·0001
	**65-74**	8,453	3,959	46·8%	(45·8%, 47·9%)	(ref)	(ref)	(ref)		(ref)	(ref)	(ref)		(ref)	(ref)	(ref)	
	**75-84**	7,758	4,451	57·4%	(56·3%, 58·5%)	1·53	1·44	1·63		1·60	1·49	1·72		1·83	1·69	1·98	
	**85+**	3,283	2,353	71·7%	(70·1%, 73·2%)	2·87	2·63	3·13		3·06	2·77	3·38		3·92	3·51	4·37	
**Stage**	**1**	3,310	424	12·8%	(11·7%, 14%)	(ref)	(ref)	(ref)	<0·0001					(ref)	(ref)	(ref)	<0·0001
	**2**	3,451	866	25·1%	(23·7%, 26·6%)	2·28	2·01	2·59						2·57	2·23	2·97	
	**3**	6,038	2,232	37·0%	(35·8%, 38·2%)	3·99	3·56	4·48						4·96	4·37	5·64	
	**4**	9,006	7,131	79·2%	(78·3%, 80·0%)	25·89	23·12	29·04						25·57	22·52	29·08	
	**Unknown**	4,334	2,617	60·4%	(58·9%, 61·8%)	10·37	9·22	11·69						8·59	7·49	9·87	
**Cancer site**	**Oesophageal**	1,703	996	58·5%	(56·1%, 60·8%)	3·10	2·77	3·46	<0·0001	4·37	3·88	4·92	<0·0001	4·42	3·88	5·04	<0·0001
	**Stomach**	1,656	1,029	62·1%	(59·8%, 64·4%)	3·61	3·22	4·04		3·92	3·47	4·43		3·19	2·79	3·66	
	**Colon**	6,142	1,920	31·3%	(30·1%, 32·4%)	(ref)	(ref)	(ref)		(ref)	(ref)	(ref)		(ref)	(ref)	(ref)	
	**Rectal**	2,577	532	20·6%	(19·1%, 22·3%)	0·57	0·51	0·64		0·80	0·71	0·90		0·84	0·74	0·95	
	**Liver**	1,155	791	68·5%	(65·7%, 71·1%)	4·78	4·18	5·48		4·99	4·32	5·78		3·81	3·24	4·47	
	**Pancreatic**	1,965	1,502	76·4%	(74·5%, 78·3%)	7·13	6·35	8·03		7·24	6·39	8·22		5·08	4·43	5·83	
	**Lung**	9,365	6,096	65·1%	(64·1%, 66·1%)	4·10	3·83	4·39		4·79	4·45	5·17		4·47	4·11	4·87	
	**Ovarian**	1,576	404	25·6%	(23·5%, 27·8%)	0·76	0·67	0·86		0·86	0·75	0·99		0·91	0·78	1·06	

Alberta		All patients	12-month mortality			Crude odds ratios				Adjusted odds ratios (stage unadjusted)				Adjusted odds ratios (stage adjusted)			
			Mort	%Mort	95% CI	Crude OR	Crude OR (LCI)	Crude OR (UCI)	p(COR)	Adj OR	Adj OR (LCI)	Adj OR (UCI)	p(AOR)	Adj OR	Adj OR (LCI)	Adj OR (UCI)	p(AOR)
	**Total**	25,182	10,438	41·5%	(40·8%, 42·1%)												
**Presentation status**	**Elective**	17,741	5,599	31·6%	(30·9%, 32·2%)	(ref)	(ref)	(ref)	<0·0001	(ref)	(ref)	(ref)	<0·0001	(ref)	(ref)	(ref)	<0·0001
	**Emergency**	7,441	4,839	65·0%	(63·9%, 66·1%)	4·03	3·81	4·27		3·86	3·62	4·12		2·63	2·45	2·83	
**Sex**	**Male**	13,235	5,702	43·1%	(42·2%, 43·9%)	(ref)	(ref)	(ref)	<0·0001	(ref)	(ref)	(ref)	<0·0001	(ref)	(ref)	(ref)	<0·0001
	**Female**	11,947	4,736	39·6%	(38·8%, 40·5%)	0·87	0·83	0·91		0·80	0·76	0·86		0·82	0·77	0·88	
**Diagnosis year**	**2012**	4,628	1,962	42·4%	(41·0%, 43·8%)	0·98	0·90	1·06	0·025	1·03	0·94	1·13	0·0090	1·04	0·93	1·15	0·26
	**2013**	4,812	2,067	43·0%	(41·6%, 44·4%)	(ref)	(ref)	(ref)		(ref)	(ref)	(ref)		(ref)	(ref)	(ref)	
	**2014**	5,307	2,193	41·3%	(40·0%, 42·7%)	0·94	0·86	1·01		0·97	0·89	1·06		1·01	0·90	1·12	
	**2015**	5,222	2,121	40·6%	(39·3%, 42·0%)	0·91	0·84	0·98		0·94	0·85	1·03		0·97	0·88	1·08	
	**2016**	5,213	2,095	40·2%	(38·9%, 41·5%)	0·89	0·82	0·97		0·91	0·83	0·99		0·94	0·84	1·04	
**Age group**	**15-64**	9,717	3,033	31·2%	(30·3%, 32·1%)	0·66	0·62	0·71	<0·0001	0·72	0·67	0·77	<0·0001	0·59	0·54	0·64	<0·0001
	**65-74**	7,245	2,939	40·6%	(39·4%, 41·7%)	(ref)	(ref)	(ref)		(ref)	(ref)	(ref)		(ref)	(ref)	(ref)	
	**75-84**	5,877	2,915	49·6%	(48·3%, 50·9%)	1·44	1·35	1·55		1·43	1·32	1·54		1·71	1·56	1·88	
	**85+**	2,343	1,551	66·2%	(64·3%, 68·1%)	2·87	2·60	3·16		3·02	2·70	3·38		3·91	3·44	4·45	
**Stage**	**1**	4,927	405	8·2%	(7·5%, 9·0%)	(ref)	(ref)	(ref)	<0·0001					(ref)	(ref)	(ref)	<0·0001
	**2**	3,804	740	19·5%	(18·2%, 20·7%)	2·70	2·37	3·07						2·47	2·14	2·85	
	**3**	5,575	1,557	27·9%	(26·8%, 29·1%)	4·33	3·85	4·87						5·47	4·81	6·23	
	**4**	9,703	7,142	73·6%	(72·7%, 74·5%)	31·14	27·89	34·84						29·3	25·93	33·18	
	**Unknown**	1,173	594	50·6%	(47·8%, 53·5%)	11·45	9·83	13·36						8·97	7·53	10·70	
**Cancer site**	**Oesophageal**	891	507	56·9%	(53·6%, 60·1%)	5·43	4·69	6·28	<0·0001	7·67	6·56	8·97	<0·0001	5·72	4·79	6·82	<0·0001
	**Stomach**	1,297	674	52·0%	(49·2%, 54·7%)	4·45	3·93	5·04		5·02	4·38	5·75		3·75	3·23	4·37	
	**Colon**	6,445	1,261	19·6%	(18·6%, 20·6%)	(ref)	(ref)	(ref)		(ref)	(ref)	(ref)		(ref)	(ref)	(ref)	
	**Rectal**	2,487	341	13·7%	(12·4%, 15·1%)	0·65	0·57	0·74		0·90	0·79	1·04		0·83	0·71	0·96	
	**Liver**	1,332	654	49·1%	(46·4%, 51·8%)	3·97	3·50	4·49		5·11	4·47	5·84		7·31	6·26	8·53	
	**Pancreatic**	2,117	1,541	72·8%	(70·9%, 74·6%)	11.00	9·82	12·33		12·67	11·22	14·32		11·06	9·62	12·74	
	**Lung**	9,441	5,171	54·8%	(53·8%, 55·8%)	4·98	4·63	5·36		5·85	5·40	6·34		4·14	3·77	4·53	
	**Ovarian**	1,172	289	24·7%	(22·3%, 27·2%)	1·35	1·16	1·56		1·87	1·59	2·20		1·84	1·54	2·20	

Atlantic Canada $		All patients	12-month mortality			Crude odds ratios				Adjusted odds ratios (stage unadjusted)				Adjusted odds ratios (stage adjusted)			
			Mort	%Mort	95% CI	Crude OR	Crude OR (LCI)	Crude OR (UCI)	p(COR)	Adj OR	Adj OR (LCI)	Adj OR (UCI)	p(AOR)	Adj OR	Adj OR (LCI)	Adj OR (UCI)	p(AOR)
	**Total**	10,970	4,600	41·9%	(41·0%, 42·9%)												
**Presentation status**	**Elective**	8,065	2,745	34·0%	(33·0%, 35·1%)	(ref)	(ref)	(ref)	<0·0001	(ref)	(ref)	(ref)	<0·0001	(ref)	(ref)	(ref)	<0·0001
	**Emergency**	2,905	1,855	63·9%	(62·1%, 65·6%)	3·42	3·13	3·74		3·79	3·42	4·21		2·74	2·44	3·08	
**Sex**	**Male**	6,010	2,615	43·5%	(42·3%, 44·8%)	(ref)	(ref)	(ref)	0·00031	(ref)	(ref)	(ref)	<0·0001	(ref)	(ref)	(ref)	0·0020
	**Female**	4,955	1,980	40·0%	(38·6%, 41·3%)	0·87	0·81	0·94		0·84	0·77	0·92		0·85	0·77	0·94	
**Diagnosis year**	**2012**	5,420	2,240	41·3%	(40·0%, 42·6%)	0·97	0·90	1·05	0·49	0·99	0·90	1·08	0·91	0·98	0·88	1·08	0·77
	**2013**	5,565	2,345	42·1%	(40·8%, 43·4%)	(ref)	(ref)	(ref)		(ref)	(ref)	(ref)		(ref)	(ref)	(ref)	
**Age group**	**15-64**	3,635	1,210	33·3%	(31·8%, 34·8%)	0·74	0·67	0·81	<0·0001	0·74	0·66	0·83	<0·0001	0·62	0·55	0·70	<0·0001
	**65-74**	3,615	1,460	40·4%	(38·8%, 42%)	(ref)	(ref)	(ref)		(ref)	(ref)	(ref)		(ref)	(ref)	(ref)	
	**75-84**	2,705	1,295	47·9%	(46·0%, 49·8%)	1·36	1·23	1·50		1·40	1·25	1·57		1·69	1·49	1·93	
	**85+**	1,015	640	63·1%	(60·0%, 66·0%)	2·49	2·16	2·87		2·91	2·45	3·45		3·67	3·04	4·43	
**Stage**	**1**	2,215	315	14·2%	(12·8%, 15·7%)	(ref)	(ref)	(ref)	<0·0001					(ref)	(ref)	(ref)	<0·0001
	**2**	1,615	265	16·4%	(14·7%, 18·3%)	1·22	1·03	1·46						1·66	1·36	2·02	
	**3**	2,255	655	29·0%	(27·2%, 31·0%)	2·39	2·06	2·78						3·49	2·95	4·13	
	**4**	3,630	2,655	73·1%	(71·7%, 74·6%)	16·32	14·21	18·79						18·51	15·81	21·74	
	**Unknown**	1,245	705	56·6%	(53·9%, 59·4%)	8·14	6·92	9·60						5·07	4·15	6·19	
**Cancer site**	**Oesophageal**	330	180	54·5%	(49·2%, 59·8%)	5·82	4·60	7·39	<0·0001	8·24	6·40	10·63	<0·0001	9·93	7·49	13·18	<0·0001
	**Stomach**	585	295	50·4%	(46·4%, 54·5%)	5·10	4·23	6·15		6·07	4·95	7·43		6·51	5·17	8·21	
	**Colon**	3,055	515	16·9%	(15·6%, 18·2%)	(ref)	(ref)	(ref)		(ref)	(ref)	(ref)		(ref)	(ref)	(ref)	
	**Rectal**	1,060	175	16·5%	(14·4%, 18·9%)	0·98	0·81	1·18		1·41	1·15	1·71		1·47	1·18	1·82	
	**Liver**	335	220	65·7%	(60·4%, 70·6%)	8·60	6·77	10·98		10·70	8·27	13·88		12·82	9·54	17·28	
	**Pancreatic**	730	570	78·1%	(74·9%, 80·9%)	16·77	13·79	20·48		20·97	17·05	25·92		20·50	16·23	26·02	
	**Lung**	4,470	2,495	55·8%	(54·4%, 57·3%)	6·16	5·52	6·89		8·19	7·25	9·26		7·21	6·30	8·27	
	**Ovarian**	420	135	32·1%	(27·9%, 36·8%)	2·38	1·90	2·97		3·17	2·48	4·06		3·46	2·62	4·54	

British Columbia $		All patients	12-month mortality			Crude odds ratios				Adjusted odds ratios (stage unadjusted)				Adjusted odds ratios (stage adjusted)			
			Mort	%Mort	95% CI	Crude OR	Crude OR (LCI)	Crude OR (UCI)	p(COR)	Adj OR	Adj OR (LCI)	Adj OR (UCI)	p(AOR)	Adj OR	Adj OR (LCI)	Adj OR (UCI)	p(AOR)
	**Total**	16,490	6,875	41·7%	(40·9%, 42·4%)												
**Presentation status**	**Elective**	11,295	3,555	31·5%	(30·6%, 32·3%)	(ref)	(ref)	(ref)	<0·0001	(ref)	(ref)	(ref)	<0·0001	(ref)	(ref)	(ref)	<0·0001
	**Emergency**	5,195	3,320	63·9%	(62·6%, 65·2%)	3·84	3·59	4·12		4·01	3·70	4·36		3·11	2·85	3·39	
**Sex**	**Male**	8,655	3,760	43·4%	(42·4%, 44·5%)	(ref)	(ref)	(ref)	<0·0001	(ref)	(ref)	(ref)	<0·0001	(ref)	(ref)	(ref)	<0·0001
	**Female**	7,840	3,115	39·7%	(38·7%, 40·8%)	0·86	0·81	0·92		0·84	0·77	0·90		0·85	0·79	0·93	
**Diagnosis year**	**2012**	8,110	3,315	40·9%	(39·8%, 41·9%)	0·95	0·89	1·01	0·081	0·95	0·88	1·03	0·096	0·95	0·88	1·03	0·14
	**2013**	8,370	3,525	42·1%	(41·1%, 43·2%)	(ref)	(ref)	(ref)		(ref)	(ref)	(ref)		(ref)	(ref)	(ref)	
**Age group**	**15-64**	5,315	1,735	32·6%	(31·4%, 33·9%)	0·74	0·68	0·80	<0·0001	0·76	0·69	0·83	<0·0001	0·68	0·62	0·76	<0·0001
	**65-74**	4,830	1,925	39·9%	(38·5%, 41·2%)	(ref)	(ref)	(ref)		(ref)	(ref)	(ref)		(ref)	(ref)	(ref)	
	**75-84**	4,375	2,025	46·3%	(44·8%, 47·8%)	1·31	1·21	1·43		1·38	1·26	1·53		1·50	1·35	1·67	
	**85+**	1,960	1,190	60·7%	(58·5%, 62·9%)	2·31	2·07	2·57		2·77	2·43	3·15		3·02	2·62	3·49	
**Stage**	**1**	2,110	155	7·3%	(6·3%, 8·5%)	(ref)	(ref)	(ref)	<0·0001					(ref)	(ref)	(ref)	<0·0001
	**2**	1,965	210	10·7%	(9·4%, 12·1%)	1·49	1·20	1·85						2·10	1·67	2·65	
	**3**	2,820	745	26·4%	(24·8%, 28·1%)	4·46	3·73	5·37						5·08	4·18	6·19	
	**4**	4,180	2,805	67·1%	(65·7%, 68·5%)	25·38	21·38	30·32						21·38	17·78	25·87	
	**Unknown^**	5,405	2,930	54·2%	(52·9%, 55·5%)	14·68	12·42	17·48						10·43	8·34	13·10	
**Cancer site**	**Oesophageal**	565	325	57·5%	(53·4%, 61·5%)	7·31	6·08	8·79	<0·0001	10·89	8·94	13·28	<0·0001	6·37	5·01	8·11	<0·0001
	**Stomach**	770	395	51·3%	(47·8%, 54·8%)	5·84	4·97	6·88		6·47	5·42	7·72		3·93	3·15	4·91	
	**Colon**	4,540	715	15·7%	(14·7%, 16·8%)	(ref)	(ref)	(ref)		(ref)	(ref)	(ref)		(ref)	(ref)	(ref)	
	**Rectal**	1,655	235	14·2%	(12·6%, 16%)	0·92	0·78	1·07		1·35	1·14	1·59		1·20	1.00	1·43	
	**Liver**	870	560	64·4%	(61·1%, 67·5%)	10·01	8·53	11·76		13·44	11·30	16·01		8·17	6·55	10·21	
	**Pancreatic**	1,305	980	75·1%	(72·7%, 77·4%)	16·44	14·18	19·11		21·08	17·98	24·77		12·48	10·11	15·43	
	**Lung**	6,015	3,440	57·2%	(55·9%, 58·4%)	7·17	6·53	7·89		9·40	8·47	10·44		7·60	6·78	8·53	
	**Ovarian**	760	190	25·0%	(22·1%, 28·2%)	1·78	1·48	2·13		2·31	1·88	2·82		1·44	1·13	1·83	

Ontario		All patients	12-month mortality			Crude odds ratios				Adjusted odds ratios (stage unadjusted)				Adjusted odds ratios (stage adjusted)			
			Mort	%Mort	95% CI	Crude OR	Crude OR (LCI)	Crude OR (UCI)	p(COR)	Adj OR	Adj OR (LCI)	Adj OR (UCI)	p(AOR)	Adj OR	Adj OR (LCI)	Adj OR (UCI)	p(AOR)
	**Total**	92,447	34,820	37·7%	(37·4%, 38%)												
**Presentation status**	**Elective**	68,316	20,197	29·6%	(29·2%, 29·9%)	(ref)	(ref)	(ref)	<0·0001	(ref)	(ref)	(ref)	<0·0001	(ref)	(ref)	(ref)	<0·0001
	**Emergency**	24,131	14,623	60·6%	(60·0%, 61·2%)	3·66	3·55	3·78		3·87	3·74	4.00		2·95	2·84	3·06	
**Sex**	**Male**	48,146	19,457	40·4%	(40·0%, 40·9%)	(ref)	(ref)	(ref)	<0·0001	(ref)	(ref)	(ref)	<0·0001	(ref)	(ref)	(ref)	<0·0001
	**Female**	44,301	15,363	34·7%	(34·2%, 35·1%)	0·78	0·76	0·80		0·77	0·75	0·80		0·79	0·76	0·82	
**Diagnosis year**	**2012**	18,090	6,974	38·6%	(37·8%, 39·3%)	1·03	0·99	1·08	0·037	1·06	1·01	1·11	<0·0001	1·05	1.00	1·11	<0·0001
	**2013**	17,978	6,794	37·8%	(37·1%, 38·5%)	(ref)	(ref)	(ref)		(ref)	(ref)	(ref)		(ref)	(ref)	(ref)	
	**2014**	18,513	6,875	37·1%	(36·4%, 37·8%)	0·97	0·93	1·01		0·94	0·89	0·99		0·94	0·89	0·99	
	**2015**	18,859	7,108	37·7%	(37·0%, 38·4%)	1.00	0·95	1·04		0·95	0·90	0·99		0·95	0·90	1.00	
	**2016**	19,007	7,069	37·2%	(36·5%, 37·9%)	0·97	0·93	1·02		0·91	0·87	0·95		0·91	0·87	0·96	
**Age group**	**15-64**	33,177	9,876	29·8%	(29·3%, 30·3%)	0·71	0·68	0·73	<0·0001	0·78	0·75	0·81	<0·0001	0·69	0·66	0·72	<0·0001
	**65-74**	28,027	10,512	37·5%	(36·9%, 38·1%)	(ref)	(ref)	(ref)		(ref)	(ref)	(ref)		(ref)	(ref)	(ref)	
	**75-84**	23,184	10,210	44·0%	(43·4%, 44·7%)	1·31	1·27	1·36		1·39	1·34	1·45		1·59	1·52	1·66	
	**85+**	8,059	4,222	52·4%	(51·3%, 53·5%)	1·83	1·74	1·93		2·30	2·17	2·43		2·95	2·77	3·15	
**Stage**	**1**	16,620	1,249	7·5%	(7·1%, 7·9%)	(ref)	(ref)	(ref)	<0·0001					(ref)	(ref)	(ref)	<0·0001
	**2**	13,177	1,864	14·1%	(13·6%, 14·8%)	2·03	1·88	2·19						2·21	2·04	2·40	
	**3**	19,476	5,201	26·7%	(26·1%, 27·3%)	4·48	4·20	4·79						5·07	4·73	5·44	
	**4**	27,579	19,015	68·9%	(68·4%, 69·5%)	27·32	25·67	29·12						22·73	21·27	24·32	
	**Unknown**	15,595	7,491	48·0%	(47·3%, 48·8%)	11·38	10·66	12·15						8·62	8.00	9·29	
**Cancer site**	**Oesophageal**	3,099	1,710	55·2%	(53·4%, 56·9%)	6.00	5·55	6·49	<0·0001	8·2	7·55	8·91	<0·0001	5·44	4·97	5·96	<0·0001
	**Stomach**	6,465	2,796	43·2%	(42·0%, 44·5%)	3·72	3·50	3·94		4·34	4·07	4·63		2·87	2·67	3·09	
	**Colon**	24,481	4,167	17·0%	(16·6%, 17·5%)	(ref)	(ref)	(ref)		(ref)	(ref)	(ref)		(ref)	(ref)	(ref)	
	**Rectal**	7,845	1,016	13·0%	(12·2%, 13·7%)	0·73	0·67	0·78		1·01	0·94	1·09		0·99	0·91	1·07	
	**Liver**	4,129	1,964	47·6%	(46·0%, 49·1%)	4·42	4·13	4·74		5·55	5·16	5·98		4·59	4·22	4·98	
	**Pancreatic**	6,456	4,089	63·3%	(62·2%, 64·5%)	8·42	7·93	8·95		10·48	9·82	11·18		7·29	6·78	7·83	
	**Lung**	35,092	17,973	51·2%	(50·7%, 51·7%)	5·12	4·92	5·32		6·51	6·24	6·80		4·59	4·38	4·81	
	**Ovarian**	4,880	1,105	22·6%	(21·5%, 23·8%)	1·43	1·32	1·54		2·08	1·92	2·26		1·72	1·57	1·87	

Saskatchewan-Manitoba $		All patients	12-month mortality			Crude odds ratios				Adjusted odds ratios (stage unadjusted)				Adjusted odds ratios (stage adjusted)			
			Mort	%Mort	95% CI	Crude OR	Crude OR (LCI)	Crude OR (UCI)	p(COR)	Adj OR	Adj OR (LCI)	Adj OR (UCI)	p(AOR)	Adj OR	Adj OR (LCI)	Adj OR (UCI)	p(AOR)
	**Total**	8,385	3,475	41·4%	(40·4%, 42·5%)												
**Presentation status**	**Elective**	5,985	1,955	32·7%	(31·5%, 33·9%)	(ref)	(ref)	(ref)	<0·0001	(ref)	(ref)	(ref)	<0·0001	(ref)	(ref)	(ref)	<0·0001
	**Emergency**	2,400	1,520	63·3%	(61·4%, 65·2%)	3·57	3·24	3·95		3·77	3·36	4·24		2·72	2·39	3·09	
**Sex**	**Male**	4,405	1,885	42·8%	(41·3%, 44·3%)	(ref)	(ref)	(ref)	0·0031	(ref)	(ref)	(ref)	<0·0001	(ref)	(ref)	(ref)	0·0015
	**Female**	3,975	1,590	40·0%	(38·5%, 41·5%)	0·88	0·80	0·96		0·8	0·72	0·89		0·83	0·73	0·93	
**Diagnosis year**	**2012**	4,080	1,720	42·2%	(40·6%, 43·7%)	1·05	0·97	1·15	0·24	1·09	0·99	1·21	0·13	1·07	0·96	1·20	0·34
	**2013**	4,270	1,755	41·1%	(39·6%, 42·6%)	(ref)	(ref)	(ref)		(ref)	(ref)	(ref)		(ref)	(ref)	(ref)	
**Age group**	**15-64**	2,770	865	31·2%	(29·5%, 33·0%)	0·65	0·58	0·73	<0·0001	0·73	0·64	0·83	<0·0001	0·63	0·54	0·73	<0·0001
	**65-74**	2,510	1,025	40·8%	(38·9%, 42·8%)	(ref)	(ref)	(ref)		(ref)	(ref)	(ref)		(ref)	(ref)	(ref)	
	**75-84**	2,110	1,010	47·9%	(45·7%, 50·0%)	1·34	1·19	1·50		1·34	1·17	1·54		1·64	1·40	1·91	
	**85+**	990	580	58·6%	(55·5%, 61·6%)	2·01	1·74	2·34		2·44	2·04	2·91		3·23	2·65	3·95	
**Stage**	**1**	1,705	255	15·0%	(13·3%, 16·7%)	(ref)	(ref)	(ref)	<0·0001					(ref)	(ref)	(ref)	<0·0001
	**2**	1,345	290	21·6%	(19·4%, 23·8%)	1·62	1·34	1·96						1·72	1·39	2·13	
	**3**	1,810	505	27·9%	(25·9%, 30·0%)	2·41	2·04	2·87						3·19	2·63	3·87	
	**4**	3,070	2,215	72·1%	(70·5%, 73·7%)	16·31	13·95	19·14						15·57	13·01	18·70	
	**Unknown**	435	220	50·6%	(45·9%, 55·2%)	6·33	5·03	7·99						5·21	3·95	6·87	
**Cancer site**	**Oesophageal**	220	130	59·1%	(52·5%, 65·4%)	8·05	6·01	10·84	<0·0001	12·41	9·12	16·99	<0·0001	12·09	8·64	17·01	<0·0001
	**Stomach**	430	225	52·3%	(47·6%, 57·0%)	5·09	4·10	6·34		5·70	4·51	7·22		5·02	3·88	6·49	
	**Colon**	2,285	380	16·6%	(15·2%, 18·2%)	(ref)	(ref)	(ref)		(ref)	(ref)	(ref)		(ref)	(ref)	(ref)	
	**Rectal**	920	150	16·3%	(14·1%, 18·8%)	0·97	0·78	1·19		1·35	1·08	1·68		1·31	1·03	1·65	
	**Liver**	295	190	64·4%	(58·8%, 69·7%)	9·31	7·17	12·16		11·88	8·98	15·79		14·41	10·57	19·73	
	**Pancreatic**	660	495	75·0%	(71·6%, 78·2%)	15·02	12·23	18·53		18·43	14·8	23·05		15·97	12·52	20·46	
	**Lung**	3,185	1,825	57·3%	(55·6%, 59·0%)	6·73	5·91	7·68		8·54	7·41	9·87		6·10	5·22	7·15	
	**Ovarian**	355	80	22·5%	(18·5%, 27·2%)	1·52	1·16	1·98		2·08	1·55	2·78		2·05	1·48	2·82	

New South Wales		All patients	12-month mortality			Crude odds ratios				Adjusted odds ratios (stage unadjusted)				Adjusted odds ratios (stage adjusted)			
			Mort	%Mort	95% CI	Crude OR	Crude OR (LCI)	Crude OR (UCI)	p(COR)	Adj OR	Adj OR (LCI)	Adj OR (UCI)	p(AOR)	Adj OR	Adj OR (LCI)	Adj OR (UCI)	p(AOR)
	**Total**	53,198	19,499	36·7%	(36·2%, 37·1%)												
**Presentation status**	**Elective**	36,753	9,663	26·3%	(25·8%, 26·7%)	(ref)	(ref)	(ref)	<0·0001	(ref)	(ref)	(ref)	<0·0001	(ref)	(ref)	(ref)	<0·0001
	**Emergency**	16,445	9,836	59·8%	(59·1%, 60·6%)	4·17	4·01	4·34		3·74	3·58	3·91		2·89	2·75	3·03	
**Sex**	**Male**	29,037	11,366	39·1%	(38·6%, 39·7%)	(ref)	(ref)	(ref)	<0·0001	(ref)	(ref)	(ref)	<0·0001	(ref)	(ref)	(ref)	<0·0001
	**Female**	24,161	8,133	33·7%	(33·1%, 34·3%)	0·79	0·76	0·82		0·80	0·77	0·84		0·82	0·78	0·86	
**Diagnosis year**	**2012**	10,361	3,961	38·2%	(37·3%, 39·2%)	(ref)	(ref)	(ref)	<0·0001	(ref)	(ref)	(ref)	<0·0001	(ref)	(ref)	(ref)	0·098
	**2013**	10,368	3,870	37·3%	(36·4%, 38·3%)	0·96	0·91	1·02		0·94	0·88	1.00		0·96	0·89	1·03	
	**2014**	10,432	3,842	36·8%	(35·9%, 37·8%)	0·94	0·89	1.00		0·94	0·88	1.00		0·96	0·90	1·03	
	**2015**	10,903	3,860	35·4%	(34·5%, 36·3%)	0·89	0·84	0·94		0·84	0·79	0·90		0·91	0·84	0·97	
	**2016**	11,134	3,966	35·6%	(34·7%, 36·5%)	0·89	0·85	0·94		0·85	0·80	0·91		0·92	0·85	0·98	
**Age group**	**15-64**	17,708	4,796	27·1%	(26·4%, 27·7%)	0·74	0·70	0·77	<0·0001	0·72	0·68	0·76	<0·0001	0·63	0·59	0·66	<0·0001
	**65-74**	16,085	5,384	33·5%	(32·7%, 34·2%)	(ref)	(ref)	(ref)		(ref)	(ref)	(ref)		(ref)	(ref)	(ref)	
	**75-84**	13,802	6,033	43·7%	(42·9%, 44·5%)	1·54	1·47	1·62		1·65	1·56	1·74		1·85	1·75	1·97	
	**85+**	5,603	3,286	58·6%	(57·4%, 59·9%)	2·82	2·65	3.00		3·27	3·03	3·52		4·31	3·98	4·67	
**Stage**	**Localised**	14,325	2,589	18·1%	(17·5%, 18·7%)	(ref)	(ref)	(ref)	<0·0001					(ref)	(ref)	(ref)	<0·0001
	**Inv. adjc. tissue/organ**	6,262	1,198	19·1%	(18·2%, 20·1%)	1·07	0·99	1·16						1·41	1·29	1·54	
	**Inv. regional LNs**	8,854	1,766	19·9%	(19·1%, 20·8%)	1·13	1·06	1·21						1·53	1·42	1·66	
	**Distant metastasis**	17,181	11,431	66·5%	(65·8%, 67·2%)	9·01	8·55	9·50						10·56	9·90	11·27	
	**Unknown**	6,576	2,515	38·2%	(37·1%, 39·4%)	2·81	2·63	3.00						2·25	2·08	2·42	
**Cancer site**	**Oesophageal**	1,970	1,053	53·5%	(51·2%, 55·6%)	5·76	5·22	6·35	<0·0001	7·34	6·61	8·15	<0·0001	8·19	7·30	9·18	<0·0001
	**Stomach**	3,179	1,313	41·3%	(39·6%, 43·0%)	3·53	3·25	3·83		4·09	3·75	4·47		3·82	3·47	4·21	
	**Colon**	16,316	2,714	16·6%	(16·1%, 17·2%)	(ref)	(ref)	(ref)		(ref)	(ref)	(ref)		(ref)	(ref)	(ref)	
	**Rectal**	5,459	645	11·8%	(11·0%, 12·7%)	0·67	0·61	0·74		0·96	0·87	1·05		1·03	0·92	1·14	
	**Liver**	3,155	1,630	51·7%	(49·9%, 53·4%)	5·36	4·94	5·81		6·81	6·24	7·45		10·67	9·65	11·79	
	**Pancreatic**	4,498	2,965	65·9%	(64·5%, 67·3%)	9·69	9.00	10·44		10·74	9·91	11·64		8·70	7·97	9·50	
	**Lung**	16,334	8,681	53·1%	(52·4%, 53·9%)	5·69	5·40	5·99		6·68	6·31	7·06		5·12	4·81	5·45	
	**Ovarian**	2,287	498	21·8%	(20·1%, 23·5%)	1·40	1·25	1·55		1·85	1·64	2·07		0·72	0·64	0·82	

Victoria		All patients	12-month mortality			Crude odds ratios				Adjusted odds ratios (stage unadjusted)				Adjusted odds ratios (stage adjusted)			
			Mort	%Mort	95% CI	Crude OR	Crude OR (LCI)	Crude OR (UCI)	p(COR)	Adj OR	Adj OR (LCI)	Adj OR (UCI)	p(AOR)	Adj OR	Adj OR (LCI)	Adj OR (UCI)	p(AOR)
	**Total**	38,212	12,885	33·7%	(33·2%, 34·2%)												
**Presentation status**	**Elective**	29,047	7,557	26·0%	(25·5%, 26·5%)	(ref)	(ref)	(ref)	<0·0001	(ref)	(ref)	(ref)	<0·0001	(ref)	(ref)	(ref)	<0·0001
	**Emergency**	9,165	5,328	58·1%	(57·1%, 59·1%)	3·95	3·76	4·15		3·70	3·50	3·92		3·33	3·15	3·53	
**Sex**	**Male**	20,720	7,484	36·1%	(35·5%, 36·8%)	(ref)	(ref)	(ref)	<0·0001	(ref)	(ref)	(ref)	<0·0001	(ref)	(ref)	(ref)	<0·0001
	**Female**	17,492	5,401	30·9%	(30·2%, 31·6%)	0·79	0·76	0·82		0·82	0·78	0·86		0·82	0·78	0·86	
**Diagnosis year**	**2012**	7,045	2,333	33·1%	(32·0%, 34·2%)	(ref)	(ref)	(ref)	0·0067	(ref)	(ref)	(ref)	0·049	(ref)	(ref)	(ref)	0·035
	**2013**	7,232	2,373	32·8%	(31·7%, 33·9%)	0·99	0·92	1·06		0·98	0·91	1·06		0·98	0·90	1·07	
	**2014**	7,646	2,530	33·1%	(32·0%, 34·2%)	1.00	0·93	1·07		0·93	0·86	1·01		0·92	0·85	1.00	
	**2015**	7,961	2,807	35·3%	(34·2%, 36·3%)	1·10	1·03	1·18		1·03	0·95	1·12		1·02	0·94	1·11	
	**2016**	8,328	2,842	34·1%	(33·1%, 35·2%)	1·05	0·98	1·12		0·95	0·88	1·03		0·95	0·88	1·03	
**Age group**	**15-64**	12,568	2,820	22·4%	(21·7%, 23·2%)	0·65	0·61	0·69	<0·0001	0·69	0·65	0·74	<0·0001	0·65	0·61	0·69	<0·0001
	**65-74**	11,016	3,390	30·8%	(29·9%, 31·6%)	(ref)	(ref)	(ref)		(ref)	(ref)	(ref)		(ref)	(ref)	(ref)	
	**75-84**	10,188	4,131	40·5%	(39·6%, 41·5%)	1·53	1·45	1·62		1·64	1·54	1·75		1·69	1·58	1·81	
	**85+**	4,440	2,544	57·3%	(55·8%, 58·7%)	3·02	2·81	3·24		3·63	3·34	3·96		3·83	3·50	4·19	
**Stage ***	**Localised**	3,587	85	2·4%	(1·9%, 2·9%)	(ref)	(ref)	(ref)	<0·0001					(ref)	(ref)	(ref)	<0·0001
	**Inv. adjc. tissue/organ**	4,011	194	4·8%	(4·2%, 5·5%)	2·09	1·62	2·73						1·47	1·13	1·93	
	**Inv. regional LNs**	3,708	316	8·5%	(7·7%, 9·5%)	3·84	3·02	4·93						3·14	2·46	4·05	
	**Distant metastasis**	2,727	1,188	43·6%	(41·7%, 45·4%)	31·8	25·48	40·22						27·31	21·75	34·72	
	**Unknown^**	24,146	11,096	46·0%	(45·3%, 46·6%)	35·03	28·41	43·83						10·08	7·95	12·94	
**Cancer site**	**Oesophageal**	1,576	783	49·7%	(47·2%, 52·1%)	5·27	4·72	5·89	<0·0001	6·80	6·04	7·66	<0·0001	3·65	3·12	4·29	<0·0001
	**Stomach**	2,597	1,105	42·5%	(40·7%, 44·5%)	3·95	3·61	4·34		4·79	4·34	5·30		2·59	2·24	3.00	
	**Colon**	11,747	1,853	15·8%	(15·1%, 16·4%)	(ref)	(ref)	(ref)		(ref)	(ref)	(ref)		(ref)	(ref)	(ref)	
	**Rectal**	4,438	468	10·5%	(9·7%, 11·5%)	0·63	0·56	0·70		0·89	0·79	0·99		0·79	0·69	0·89	
	**Liver**	2,323	1,097	47·2%	(45·2%, 49·3%)	4·78	4·34	5·26		5·89	5·31	6·54		3·25	2·81	3·78	
	**Pancreatic**	3,172	1,959	61·8%	(60·1%, 63·4%)	8·62	7·91	9·41		9·98	9·08	10·97		5·43	4·71	6·26	
	**Lung**	10,620	5,320	50·1%	(49·1%, 51·0%)	5·36	5·04	5·71		6·48	6·05	6·94		3·51	3·09	3·98	
	**Ovarian**	1,739	300	17·3%	(15·5%, 19·1%)	1·11	0·97	1·27		1·63	1·41	1·89		0·90	0·75	1·08	

New Zealand		All patients	12-month mortality			Crude odds ratios				Adjusted odds ratios (stage unadjusted)				Adjusted odds ratios (stage adjusted)			
			Mort	%Mort	95% CI	Crude OR	Crude OR (LCI)	Crude OR (UCI)	p(COR)	Adj OR	Adj OR (LCI)	Adj OR (UCI)	p(AOR)	Adj OR	Adj OR (LCI)	Adj OR (UCI)	p(AOR)
	**Total**	28,794	12,225	42·5%	(41·9%, 43·0%)												
**Presentation status**	**Elective**	16,556	4,653	28·1%	(27·4%, 28·8%)	(ref)	(ref)	(ref)	<0·0001	(ref)	(ref)	(ref)	<0·0001	(ref)	(ref)	(ref)	<0·0001
	**Emergency**	12,238	7,572	61·9%	(61·0%, 62·7%)	4·15	3·95	4·36		3·73	3·53	3·95		2·57	2·42	2·74	
**Sex**	**Male**	14,751	6,496	44·0%	(43·2%, 44·8%)	(ref)	(ref)	(ref)	<0·0001	(ref)	(ref)	(ref)	<0·0001	(ref)	(ref)	(ref)	<0·0001
	**Female**	14,043	5,729	40·8%	(40·0%, 41·6%)	0·88	0·84	0·92		0·88	0·83	0·93		0·86	0·81	0·92	
**Diagnosis year**	**2012**	6,961	3,009	43·2%	(42·1%, 44·4%)	(ref)	(ref)	(ref)	0·059	(ref)	(ref)	(ref)	0·00074	(ref)	(ref)	(ref)	0·011
	**2013**	6,994	2,981	42·6%	(41·5%, 43·8%)	0·98	0·91	1·04		0·96	0·88	1·04		0·97	0·89	1·06	
	**2014**	7,447	3,193	42·9%	(41·8%, 44·0%)	0·99	0·92	1·05		0·99	0·91	1·07		1·03	0·94	1·12	
	**2015**	7,392	3,042	41·2%	(40·0%, 42·3%)	0·92	0·86	0·98		0·85	0·78	0·92		0·88	0·81	0·96	
**Age group**	**15-64**	8,819	2,992	33·9%	(32·9%, 34·9%)	0·80	0·75	0·85	<0·0001	0·74	0·69	0·79	<0·0001	0·66	0·61	0·72	<0·0001
	**65-74**	8,785	3,430	39·0%	(38·0%, 40·1%)	(ref)	(ref)	(ref)		(ref)	(ref)	(ref)		(ref)	(ref)	(ref)	
	**75-84**	7,887	3,716	47·1%	(46·0%, 48·2%)	1·39	1·31	1·48		1·65	1·53	1·78		1·89	1·74	2·04	
	**85+**	3,303	2,087	63·2%	(61·5%, 64·8%)	2·68	2·47	2·91		3·62	3·28	3·99		4·27	3·83	4·76	
**Stage**	**Local**	3,893	184	4·7%	(4·1%, 5·4%)	0·02	0·02	0·02	<0·0001					0·03	0·03	0·04	<0·0001
	**Inv. adjc. tissue/organ**	2,421	299	12·4%	(11·1%, 13·7%)	0·06	0·05	0·06						0·08	0·07	0·09	
	**Inv. regional LNs**	4,213	890	21·1%	(19·9%, 22·4%)	0·11	0·10	0·12						0·13	0·12	0·14	
	**Distant metastasis**	9,766	6,990	71·6%	(70·7%, 72·5%)	(ref)	(ref)	(ref)						(ref)	(ref)	(ref)	
	**Unkown**	8,501	3,862	45·4%	(44·4%, 46·5%)	0·33	0·31	0·35						0·24	0·22	0·26	
**Cancer site**	**Oesophageal**	1,196	733	61·3%	(58·5%, 64·0%)	5·46	4·82	6·20	<0·0001	7·24	6·31	8·30	<0·0001	5·09	4·38	5·91	<0·0001
	**Stomach**	1,520	821	54·0%	(51·5%, 56·5%)	4·05	3·62	4·53		4·81	4·26	5·44		3·51	3·07	4·03	
	**Colon**	9,502	2,136	22·5%	(21·7%, 23·3%)	(ref)	(ref)	(ref)		(ref)	(ref)	(ref)		(ref)	(ref)	(ref)	
	**Rectal**	3,247	503	15·5%	(14·3%, 16·8%)	0·63	0·57	0·70		0·89	0·79	1.00		0·77	0·68	0·87	
	**Liver**	1,243	721	58·0%	(55·2%, 60·7%)	4·76	4·22	5·39		6·15	5·38	7·04		5·61	4·82	6·53	
	**Pancreatic**	2,182	1,694	77·6%	(75·8%, 79·3%)	11·97	10·71	13·40		12·97	11·51	14·63		7·70	6·75	8·80	
	**Lung**	8,481	5,209	61·4%	(60·4%, 62·5%)	5·49	5·14	5·86		6·53	6·08	7·02		4·42	4·08	4·80	
	**Ovarian**	1,423	408	28·7%	(26·4%, 31·1%)	1·39	1·22	1·57		1·61	1·40	1·85		0·64	0·55	0·74	

Mort: 12-month mortality; CI: Confidence Interval; OR: Odds ratio; LCI: Lower confidence interval; UCI: Upper confidence interval; COR: Crude odds ratio; Adj: Adjusted; AOR: Adjusted odds ratio; Inv: Invading; Adjc: Adjacent; LNs: Lymph Nodes; Ref: Reference category

* Data shown for stage categories in Victoria does not include 33 cases in groups other than shown.

^ For British Columbia the unknown stage category includes all cases other than colon, rectal and lung, and the proportion of cases with the latter three cancers with unknown stage. For Victoria, the unknown stage category includes all cases other than colon and rectal, and the proportion of cases with the latter two cancers with unknown stage.

$ For Atlantic Canada, British Columbia, and Saskatchewan-Manitoba, random rounding within cells to a multiple of 5 was used for the tabulated variable groups, to adhere to rules for minimising residual disclosure risk.

Supplementary Material 11. Meta-analysis of associations between emergency presentation status (defined as diagnosis of cancer within 30 days after an emergency hospital admission) and 12-month mortality (adjusted for person-level variables other than stage, and emergency presentation broad/narrow definition subtype)

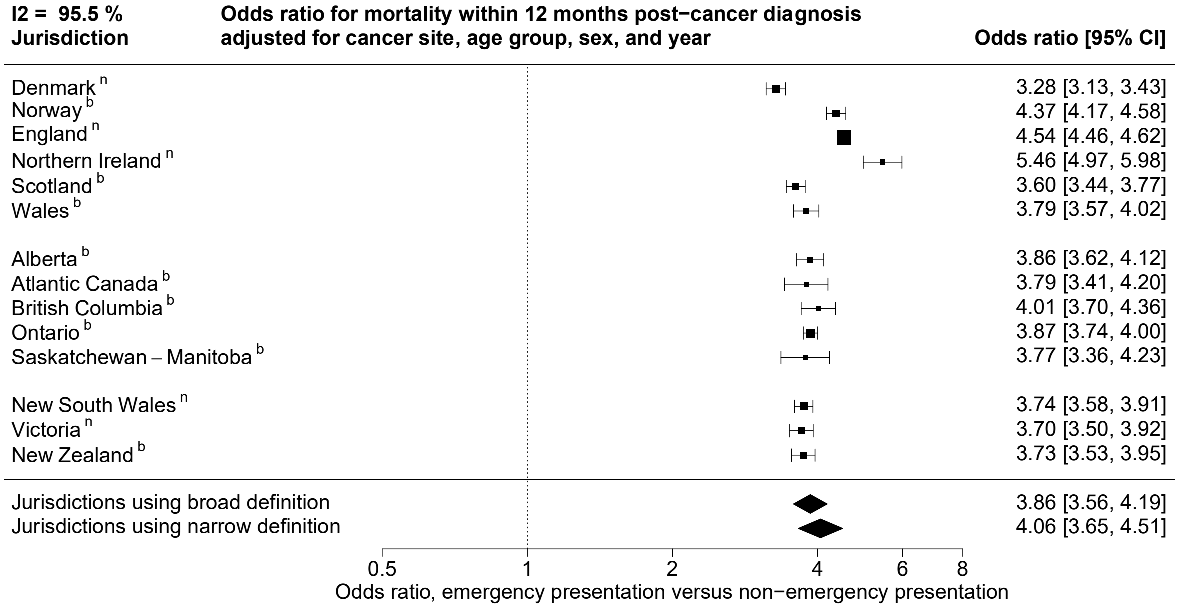

Supplementary Material 12. Estimates used in analysis of jursidiction-specific associations between emergency presentation (defined as diagnosis of cancer within 30 days after an emergency hospital admission) and cancer survival. 1-year net survival data relate to references 1 (Arnold et al.) and 18 (Rutherford et al.); data on percentage of emergency presentations relate to the present study.

	Oesophageal		Stomach		Colon		Rectal	
**A.**	**EP (%)**	**1-year net survival (%)**	**EP (%)**	**1-year net survival (%)**	**EP (%)**	**1-year net survival (%)**	**EP (%)**	**1-year net survival (%)**
**Denmark**	24·3	44·4	32·2	52·5	28·3	82·8	13·5	87·6
**Norway**	32·7	47·3	40·0	53·5	35·8	82·6	15·8	89·1
**England**	19·7	46·6	31·4	47·4	29·3	77.0	10·7	84·9
**Northern Ireland**	19·9	48·5	29·6	45.0	23·8	80.0	9·1	84·6
**Scotland**	25·2	44·9	39·1	45.0	35·1	77·4	14·1	84·6
**Wales**	25·5	46·4	39·1	43·6	34·2	74·7	13·8	84·9
**Alberta**	23·0	41·1	34·6	49.0	28·6	82·3	12·9	86·5
**British Columbia**	28·3	46·5	40·3	48·3	27·5	83·6	12·0	87·7
**Manitoba**	14·0	37·5	24·5	52·5	24·5	81·5	11·7	84·5
**New Brunswick**	18·2	46·7	27·6	49·7	30·2	84·1	11·6	85·3
**Nova Scotia**	20·6	41·5	37·3	47.0	28·3	78·8	13·1	81·6
**Ontario**	20·3	44·3	26·9	55·4	27·2	84·2	11·0	87.0
**Prince Edward Island**	33·3	36·8	25·0	49·9	24·0	82·1	13·6	87·3
**Saskatchewan**	23·3	41·3	37·8	51·5	30·8	83·1	15·6	85·2
**New South Wales**	26·9	46·5	30·5	56·1	27·6	86·9	12·1	88·7
**Victoria**	20·0	53·4	23·7	60·7	22·9	87·1	9·1	90·8
**New Zealand**	36·8	44·1	47·8	49.0	36·6	80·8	19·8	85·9

	Liver		Pancreatic		Lung		Ovarian	
**B.**	**EP (%)**	**1-year net survival (%)**	**EP (%)**	**1-year net survival (%)**	**EP (%)**	**1-year net survival (%)**	**EP (%)**	**1-year net survival (%)**
**Denmark**	43·3	37·1	48·0	31·1	36·4	46·2	20·6	77·4
**Norway**	50·6	40·9	55·4	30·5	39·4	46·7	34·9	77·5
**England**	42·4	36·4	46·9	26·7	34·7	39·2	34·8	70·8
**Northern Ireland**	36·9	37·2	42·7	20·9	32·0	36·9	29·6	66·2
**Scotland**	47·5	42·7	59·2	24·4	42·1	38·5	42·8	68·5
**Wales**	50·5	37·2	56·7	25·6	41·5	36·8	40·8	68·6
**Alberta**	31·7	45·9	40·9	29·8	33·3	47.0	25·7	71·2
**British Columbia**	38·1	36·9	41·4	29·7	33·2	46·4	31·7	75·2
**Manitoba**	26·5	35·0	30·8	31·2	26·1	45·3	22·8	77·1
**New Brunswick**	42·5	39·1	45·6	28·5	26·2	51·7	37·5	67·8
**Nova Scotia**	24·1	40·9	30·8	21·6	23·8	43·4	23·9	67.0
**Ontario**	28·3	46·0	35·3	33·8	27·5	47·5	23·9	72·4
**Prince Edward Island**	50·0	49·3	37·5	27·2	38·7	41·6	22·2	65·9
**Saskatchewan**	41·7	35·4	44·1	30·2	36·1	46·1	39·7	71·7
**New South Wales**	36·8	46·4	45·3	35·5	36·4	46·5	28·4	76·3
**Victoria**	31·9	51·5	34·1	40·6	27·8	49·7	20·9	78·5
**New Zealand**	49·7	41·8	60·4	24·8	51·1	39·9	48·1	71·5

EP: Emergency presentation.

Supplementary Material 13: Numerator and denominator values relating to 1-, 3- and 12-month mortality by emergency presentation status. This supplement is an adjunct to Supplementary Material 9.

Across the tables, superscript indicators ‘n’ and ‘b’ after jurisdictional names describe whether a narrow or broad operational definition of emergency presentation was used – please also see main paper-Methods, and appendix pp.4-5.

A. 1-month mortality

	EP				Non-EP				Difference, EP - non-EP	
Jurisdiction	**Patients**	**Deaths (1m)**	**Mortality %**	**(95% CI)**	**Patients**	**Deaths (1m)**	**Mortality %**	**(95% CI)**	**%-point**	**(95% CI)**
Oesophageal cancer										
**Denmark** [n]	461	92	20·0%	(16·6%, 23·8%)	1,435	56	3·9%	(3·0%, 5·0%)	16·1%	(12·3%, 19·8%)
**Norway** [b]	435	86	19·8%	(16·3%, 23·8%)	897	26	2·9%	(2·0%, 4·2%)	16·9%	(13·0%, 20·8%)
**England** [n]	5,364	1,594	29·7%	(28·5%, 31·0%)	21,910	807	3·7%	(3·4%, 3·9%)	26·0%	(24·8%, 27·3%)
**Northern Ireland** [n]	193	45	23·3%	(17·9%, 29·8%)	779	31	4·0%	(2·8%, 5·6%)	19·3%	(13·2%, 25·5%)
**Scotland** [b]	851	158	18·6%	(16·1%, 21·3%)	2,527	66	2·6%	(2·1%, 3·3%)	16·0%	(13·3%, 18·6%)
**Wales** [b]	435	98	22·5%	(18·9%, 26·7%)	1,268	49	3·9%	(2·9%, 5·1%)	18·7%	(14·6%, 22·7%)
**Alberta** [b]	76	13	17·1%	(10·3%, 27·1%)	276	12	4·3%	(2·5%, 7·4%)	12·8%	(4·0%, 21·6%)
**Atlantic Canada** [b]$	75	25	33·3%	(23·7%, 44·6%)	255	15	5·9%	(3·6%, 9·5%)	27·5%	(16·4%, 38·5%)
**British Columbia** [b]$	145	30	20·7%	(14·9%, 28·0%)	425	10	2·4%	(1·3%, 4·3%)	18·3%	(11·6%, 25·1%)
**Ontario** [b]	630	128	20·3%	(17·4%, 23·6%)	2,469	80	3·2%	(2·6%, 4·0%)	17·1%	(13·9%, 20·3%)
**Saskatchewan-Manitoba** [b] $	35	15	42·9%	(28·0%, 59·1%)	180	5	2·8%	(1·2%, 6·3%)	40·1%	(23·5%, 56·6%)
**New South Wales** [n]	530	98	18·5%	(15·4%, 22·0%)	1,440	46	3·2%	(2·4%, 4·2%)	15·3%	(11·9%, 18·7%)
**Victoria** [n]	315	79	25·1%	(20·6%, 30·1%)	1,261	44	3·5%	(2·6%, 4·7%)	21·6%	(16·7%, 26·5%)
**New Zealand** [b]	440	61	13·9%	(10·9%, 17·4%)	756	12	1·6%	(0·9%, 2·8%)	12·3%	(8·9%, 15·6%)
Stomach cancer										
**Denmark** [n]	719	124	17·2%	(14·7%, 20·2%)	1,512	49	3·2%	(2·5%, 4·3%)	14·0%	(11·1%, 16·9%)
**Norway** [b]	943	153	16·2%	(14·0%, 18·7%)	1,414	36	2·5%	(1·8%, 3·5%)	13·7%	(11·2%, 16·2%)
**England** [n]	6,224	1,829	29·4%	(28·3%, 30·5%)	13,589	728	5·4%	(5·0%, 5·7%)	24·0%	(22·8%, 25·2%)
**Northern Ireland** [n]	293	79	27·0%	(22·2%, 32·3%)	697	38	5·5%	(4·0%, 7·4%)	21·5%	(16·2%, 26·9%)
**Scotland** [b]	913	185	20·3%	(17·8%, 23·0%)	1,424	52	3·7%	(2·8%, 4·8%)	16·6%	(13·8%, 19·4%)
**Wales** [b]	648	162	25·0%	(21·8%, 28·5%)	1,008	43	4·3%	(3·2%, 5·7%)	20·7%	(17·2%, 24·3%)
**Alberta** [b]	161	35	21·7%	(16·1%, 28·7%)	323	22	6·8%	(4·5%, 10·1%)	14·9%	(8·0%, 21·9%)
**Atlantic Canada** [b]$	185	50	27·0%	(21·1%, 33·8%)	395	15	3·8%	(2·3%, 6·2%)	23·2%	(16·6%, 29·9%)
**British Columbia** [b]$	320	65	20·3%	(16·3%, 25·1%)	445	20	4·5%	(2·9%, 6·8%)	15·8%	(11·0%, 20·6%)
**Ontario** [b]	1,738	305	17·5%	(15·8%, 19·4%)	4,727	160	3·4%	(2·9%, 3·9%)	14·2%	(12·3%, 16·0%)
**Saskatchewan-Manitoba** [b] $	145	25	17·2%	(12·0%, 24·2%)	290	10	3·4%	(1·9%, 6·2%)	13·8%	(7·3%, 20·3%)
**New South Wales** [n]	969	140	14·4%	(12·4%, 16·8%)	2,210	55	2·5%	(1·9%, 3·2%)	12·0%	(9·7%, 14·3%)
**Victoria** [n]	616	120	19·5%	(16·5%, 22·8%)	1,981	46	2·3%	(1·7%, 3·1%)	17·2%	(14·0%, 20·4%)
**New Zealand** [b]	726	134	18·5%	(15·8%, 21·4%)	794	24	3·0%	(2·0%, 4·5%)	15·4%	(12·4%, 18·5%)
Colon cancer										
**Denmark** [n]	3,655	415	11·4%	(10·4%, 12·4%)	9,273	157	1·7%	(1·4%, 2·0%)	9·7%	(8·6%, 10·7%)
**Norway** [b]	5,354	585	10·9%	(10·1%, 11·8%)	9,587	204	2·1%	(1·9%, 2·4%)	8·8%	(7·9%, 9·7%)

**England** [n]	25,859	5,104	19·7%	(19·3%, 20·2%)	62,360	1,797	2·9%	(2·8%, 3·0%)	16·9%	(16·4%, 17·4%)
**Northern Ireland** [n]	914	153	16·7%	(14·5%, 19·3%)	2,932	81	2·8%	(2·2%, 3·4%)	14·0%	(11·5%, 16·5%)
**Scotland** [b]	3,366	503	14·9%	(13·8%, 16·2%)	6,234	131	2·1%	(1·8%, 2·5%)	12·8%	(11·6%, 14·1%)
**Wales** [b]	2,102	357	17·0%	(15·4%, 18·6%)	4,040	82	2·0%	(1·6%, 2·5%)	15·0%	(13·3%, 16·6%)
**Alberta** [b]	747	98	13·1%	(10·9%, 15·7%)	1,633	35	2·1%	(1·5%, 3·0%)	11·0%	(8·5%, 13·5%)
**Atlantic Canada** [b]$	850	95	11·2%	(9·2%, 13·5%)	2,200	50	2·3%	(1·7%, 3·0%)	8·9%	(6·7%, 11·1%)
**British Columbia** [b]$	1,360	135	9·9%	(8·4%, 11·6%)	3,185	50	1·6%	(1·2%, 2·1%)	8·4%	(6·7%, 10·0%)
**Ontario** [b]	6,653	569	8·6%	(7·9%, 9·2%)	17,828	255	1·4%	(1·3%, 1·6%)	7·1%	(6·4%, 7·8%)
**Saskatchewan-Manitoba** [b] $	640	75	11·7%	(9·5%, 14·4%)	1,645	25	1·5%	(1·0%, 2·2%)	10·2%	(7·6%, 12·8%)
**New South Wales** [n]	4,499	423	9·4%	(8·6%, 10·3%)	11,817	144	1·2%	(1·0%, 1·4%)	8·2%	(7·3%, 9·1%)
**Victoria** [n]	2,689	288	10·7%	(9·6%, 11·9%)	9,058	130	1·4%	(1·2%, 1·7%)	9·3%	(8·1%, 10·5%)
**New Zealand** [b]	3,480	358	10·3%	(9·3%, 11·3%)	6,022	80	1·3%	(1·1%, 1·7%)	9·0%	(7·9%, 10·0%)
Rectal cancer										
**Denmark** [n]	811	69	8·5%	(6·8%, 10·6%)	5,194	49	0·9%	(0·7%, 1·2%)	7·6%	(5·6%, 9·5%)
**Norway** [b]	888	89	10·0%	(8·2%, 12·2%)	4,724	44	0·9%	(0·7%, 1·2%)	9·1%	(7·1%, 11·1%)
**England** [n]	3,628	692	19·1%	(17·8%, 20·4%)	30,277	403	1·3%	(1·2%, 1·5%)	17·7%	(16·5%, 19·0%)
**Northern Ireland** [n]	125	21	16·8%	(11·3%, 24·3%)	1,251	13	1·0%	(0·6%, 1·8%)	15·8%	(9·2%, 22·3%)
**Scotland** [b]	499	54	10·8%	(8·4%, 13·9%)	3,036	28	0·9%	(0·6%, 1·3%)	9·9%	(7·2%, 12·6%)
**Wales** [b]	355	57	16·1%	(12·6%, 20·2%)	2,222	23	1·0%	(0·7%, 1·5%)	15·0%	(11·2%, 18·9%)
**Alberta** [b]	136	13	9·6%	(5·7%, 15·7%)	817	10	1·2%	(0·7%, 2·2%)	8·3%	(3·3%, 13·3%)
**Atlantic Canada** [b]$	120	20	16·7%	(11·1%, 24·3%)	935	10	1·1%	(0·6%, 2·0%)	15·6%	(8·9%, 22·3%)
**British Columbia** [b]$	230	25	10·9%	(7·5%, 15·6%)	1,430	10	0·7%	(0·4%, 1·3%)	10·2%	(6·1%, 14·2%)
**Ontario** [b]	860	74	8·6%	(6·9%, 10·7%)	6,985	41	0·6%	(0·4%, 0·8%)	8·0%	(6·1%, 9·9%)
**Saskatchewan-Manitoba** [b] $	130	20	15·4%	(10·2%, 22·6%)	795	5	0·6%	(0·3%, 1·5%)	14·8%	(8·5%, 21·0%)
**New South Wales** [n]	660	42	6·4%	(4·7%, 8·5%)	4,799	23	0·5%	(0·3%, 0·7%)	5·9%	(4·0%, 7·8%)
**Victoria** [n]	403	33	8·2%	(5·9%, 11·3%)	4,035	25	0·6%	(0·4%, 0·9%)	7·6%	(4·9%, 10·3%)
**New Zealand** [b]	643	47	7·3%	(5·5%, 9·6%)	2,604	18	0·7%	(0·4%, 1·1%)	6·6%	(4·6%, 8·7%)
Liver cancer										
**Denmark** [n]	668	190	28·4%	(25·2%, 32·0%)	873	44	5·0%	(3·8%, 6·7%)	23·4%	(19·7%, 27·1%)
**Norway** [b]	597	222	37·2%	(33·4%, 41·1%)	584	74	12·7%	(10·2%, 15·6%)	24·5%	(19·8%, 29·2%)
**England** [n]	7,497	2,631	35·1%	(34·0%, 36·2%)	10,202	894	8·8%	(8·2%, 9·3%)	26·3%	(25·1%, 27·5%)
**Northern Ireland** [n]	215	86	40·0%	(33·7%, 46·7%)	367	33	9·0%	(6·5%, 12·4%)	31·0%	(23·8%, 38·2%)
**Scotland** [b]	1,111	264	23·8%	(21·4%, 26·4%)	1,229	55	4·5%	(3·5%, 5·8%)	19·3%	(16·5%, 22·0%)
**Wales** [b]	583	164	28·1%	(24·6%, 31·9%)	572	40	7·0%	(5·2%, 9·4%)	21·1%	(16·9%, 25·3%)
**Alberta** [b]	152	51	33·6%	(26·5%, 41·4%)	338	23	6·8%	(4·6%, 10·0%)	26·7%	(18·8%, 34·7%)
**Atlantic Canada** [b]$	115	40	34·8%	(26·7%, 43·9%)	215	25	11·6%	(8·0%, 16·6%)	23·2%	(13·5%, 32·9%)
**British Columbia** [b]$	340	120	35·3%	(30·4%, 40·5%)	535	45	8·4%	(6·3%, 11·1%)	26·9%	(21·3%, 32·5%)
**Ontario** [b]	1,167	188	16·1%	(14·1%, 18·3%)	2,962	119	4·0%	(3·4%, 4·8%)	12·1%	(9·9%, 14·3%)
**Saskatchewan-Manitoba** [b] $	105	35	33·3%	(25·0%, 42·8%)	190	20	10·5%	(6·9%, 15·7%)	22·8%	(12·8%, 32·8%)
**New South Wales** [n]	1,161	309	26·6%	(24·2%, 29·2%)	1,994	97	4·9%	(4·0%, 5·9%)	21·8%	(19·0%, 24·5%)
**Victoria** [n]	742	168	22·6%	(19·8%, 25·8%)	1,581	89	5·6%	(4·6%, 6·9%)	17·0%	(13·8%, 20·2%)
**New Zealand** [b]	618	144	23·3%	(20·1%, 26·8%)	625	22	3·5%	(2·3%, 5·3%)	19·8%	(16·1%, 23·4%)
Pancreatic cancer										
**Denmark** [n]	1,674	444	26·5%	(24·5%, 28·7%)	1,817	176	9·7%	(8·4%, 11·1%)	16·8%	(14·3%, 19·4%)
**Norway** [b]	2,059	677	32·9%	(30·9%, 34·9%)	1,656	227	13·7%	(12·1%, 15·4%)	19·2%	(16·6%, 21·8%)
**England** [n]	14,291	5,313	37·2%	(36·4%, 38·0%)	16,151	2,062	12·8%	(12·3%, 13·3%)	24·4%	(23·5%, 25·4%)

**Northern Ireland [n]**	496	212	42·7%	(38·5%, 47·1%)	666	83	12·5%	(10·2%, 15·2%)	30·3%	(25·3%, 35·3%)
**Scotland [b]**	1,809	456	25·2%	(23·3%, 27·3%)	1,245	124	10·0%	(8·4%, 11·7%)	15·2%	(12·6%, 17·8%)
**Wales [b]**	1,115	326	29·2%	(26·6%, 32·0%)	850	110	12·9%	(10·9%, 15·4%)	16·3%	(12·8%, 19·8%)
**Alberta [b]**	333	96	28·8%	(24·2%, 33·9%)	483	44	9·1%	(6·9%, 12·0%)	19·7%	(14·2%, 25·2%)
**Atlantic Canada [b]$**	280	95	33·9%	(28·6%, 39·7%)	450	60	13·3%	(10·5%, 16·8%)	20·6%	(14·2%, 27·0%)
**British Columbia [b]$**	545	165	30·3%	(26·6%, 34·3%)	755	85	11·3%	(9·2%, 13·7%)	19·0%	(14·5%, 23·5%)
**Ontario [b]**	2,279	401	17·6%	(16·1%, 19·2%)	4,177	237	5·7%	(5·0%, 6·4%)	11·9%	(10·2%, 13·6%)
**Saskatchewan-Manitoba [b] $**	255	85	33·3%	(27·8%, 39·3%)	415	40	9·6%	(7·2%, 12·9%)	23·7%	(17·2%, 30·1%)
**New South Wales [n]**	2,036	416	20·4%	(18·7%, 22·2%)	2,462	149	6·1%	(5·2%, 7·1%)	14·4%	(12·4%, 16·4%)
**Victoria [n]**	1,083	277	25·6%	(23·1%, 28·3%)	2,089	143	6·8%	(5·8%, 8·0%)	18·7%	(15·9%, 21·5%)
**New Zealand [b]**	1,317	290	22·0%	(19·9%, 24·3%)	865	56	6·5%	(5·0%, 8·3%)	15·5%	(12·8%, 18·3%)
Lung cancer										
**Denmark [n]**	5,818	1,276	21·9%	(20·9%, 23·0%)	10,185	447	4·4%	(4·0%, 4·8%)	17·5%	(16·4%, 18·7%)
**Norway [b]**	5,679	1,406	24·8%	(23·7%, 25·9%)	8,723	449	5·1%	(4·7%, 5·6%)	19·6%	(18·4%, 20·8%)
**England [n]**	46,951	17,140	36·5%	(36·1%, 36·9%)	88,445	7,033	8·0%	(7·8%, 8·1%)	28·6%	(28·1%, 29·0%)
**Northern Ireland [n]**	1,843	644	34·9%	(32·8%, 37·1%)	3,909	281	7·2%	(6·4%, 8·0%)	27·8%	(25·4%, 30·1%)
**Scotland [b]**	7,863	1,948	24·8%	(23·8%, 25·7%)	10,799	615	5·7%	(5·3%, 6·1%)	19·1%	(18·0%, 20·1%)
**Wales [b]**	3,882	1,147	29·5%	(28·1%, 31·0%)	5,483	325	5·9%	(5·3%, 6·6%)	23·6%	(22·1%, 25·2%)
**Alberta [b]**	1,169	295	25·2%	(22·8%, 27·8%)	2,347	153	6·5%	(5·6%, 7·6%)	18·7%	(16·0%, 21·4%)
**Atlantic Canada [b]$**	1,155	350	30·3%	(27·7%, 33·0%)	3,310	245	7·4%	(6·6%, 8·3%)	22·9%	(20·1%, 25·7%)
**British Columbia [b]$**	2,015	615	30·5%	(28·5%, 32·6%)	4,000	245	6·1%	(5·4%, 6·9%)	24·4%	(22·3%, 26·5%)
**Ontario [b]**	9,640	1,946	20·2%	(19·4%, 21·0%)	25,452	1,058	4·2%	(3·9%, 4·4%)	16·0%	(15·2%, 16·9%)
**Saskatchewan-Manitoba [b] $**	980	295	30·1%	(27·3%, 33·0%)	2,210	155	7·0%	(6·0%, 8·2%)	23·1%	(20·0%, 26·2%)
**New South Wales [n]**	5,941	1,185	19·9%	(18·9%, 21·0%)	10,393	386	3·7%	(3·4%, 4·1%)	16·2%	(15·2%, 17·3%)
**Victoria [n]**	2,954	637	21·6%	(20·1%, 23·1%)	7,666	356	4·6%	(4·2%, 5·1%)	16·9%	(15·4%, 18·5%)
**New Zealand [b]**	4,330	931	21·5%	(20·3%, 22·7%)	4,151	154	3·7%	(3·2%, 4·3%)	17·8%	(16·4%, 19·1%)
Ovarian cancer										
**Denmark [n]**	393	53	13·5%	(10·5%, 17·2%)	1,515	24	1·6%	(1·1%, 2·3%)	11·9%	(8·5%, 15·3%)
**Norway [b]**	911	126	13·8%	(11·7%, 16·2%)	1,696	28	1·7%	(1·1%, 2·4%)	12·2%	(9·9%, 14·5%)
**England [n]**	7,120	1,443	20·3%	(19·3%, 21·2%)	13,326	466	3·5%	(3·2%, 3·8%)	16·8%	(15·8%, 17·8%)
**Northern Ireland [n]**	243	45	18·5%	(14·1%, 23·9%)	578	28	4·8%	(3·4%, 6·9%)	13·7%	(8·5%, 18·9%)
**Scotland [b]**	909	129	14·2%	(12·1%, 16·6%)	1,214	46	3·8%	(2·9%, 5·0%)	10·4%	(7·9%, 12·9%)
**Wales [b]**	643	87	13·5%	(11·1%, 16·4%)	933	24	2·6%	(1·7%, 3·8%)	11·0%	(8·1%, 13·8%)
**Alberta [b]**	103	8	7·8%	(4·0%, 14·6%)	346	11	3·2%	(1·8%, 5·6%)	4·6%	(-0·9%, 10·1%)
**Atlantic Canada [b]$**	115	30	26·1%	(18·9%, 34·8%)	300	10	3·3%	(1·8%, 6·0%)	22·8%	(14·5%, 31·0%)
**British Columbia [b]$**	250	40	16·0%	(12·0%, 21·1%)	505	15	3·0%	(1·8%, 4·8%)	13·0%	(8·3%, 17·8%)
**Ontario [b]**	1,164	166	14·3%	(12·4%, 16·4%)	3,716	59	1·6%	(1·2%, 2·0%)	12·7%	(10·6%, 14·7%)
**Saskatchewan-Manitoba [b] $**	110	20	18·2%	(12·1%, 26·4%)	255	10	3·9%	(2·1%, 7·1%)	14·3%	(6·7%, 21·9%)
**New South Wales [n]**	649	94	14·5%	(12·0%, 17·4%)	1,638	27	1·6%	(1·1%, 2·4%)	12·8%	(10·1%, 15·6%)
**Victoria [n]**	363	51	14·0%	(10·9%, 18·0%)	1,376	27	2·0%	(1·4%, 2·8%)	12·1%	(8·4%, 15·7%)
**New Zealand [b]**	684	72	10·5%	(8·4%, 13·1%)	739	11	1·5%	(0·8%, 2·6%)	9·0%	(6·6%, 11·5%)

EP: Emergency Presentation; CI: Confidence interval, 1m: 1-month

$ To comply with requirements for minimising residual disclosure risk, all actual counts for these jurisdictions were randomly rounded to a higher or lower multiple of 5 within cells. Sample sizes used in different aspects of the analysis may vary minimally for this reason.

B. 3-month mortality

	EP				Non-EP				Difference, EP - non-EP	
Jurisdiction	**Patients**	**Deaths (3m)**	**Mortality %**	**(95% CI)**	**Patients**	**Deaths (3m)**	**Mortality %**	**(95% CI)**	**%-point**	**(95% CI)**
Oesophageal cancer										
**Denmark** [n]	461	191	41·4%	(37·0%, 46·0%)	1,435	195	13·6%	(11·9%, 15·5%)	27·8%	(23·0%, 32·7%)
**Norway** [b]	435	158	36·3%	(31·9%, 40·9%)	897	98	10·9%	(9·0%, 13·1%)	25·4%	(20·4%, 30·4%)
**England** [n]	5,364	2,951	55·0%	(53·7%, 56·3%)	21,910	3,167	14·5%	(14·0%, 14·9%)	40·6%	(39·1%, 42·0%)
**Northern Ireland** [n]	193	83	43·0%	(36·2%, 50·1%)	779	105	13·5%	(11·3%, 16·1%)	29·5%	(22·1%, 36·9%)
**Scotland** [b]	851	406	47·7%	(44·4%, 51·1%)	2,527	334	13·2%	(12·0%, 14·6%)	34·5%	(30·9%, 38·1%)
**Wales** [b]	435	224	51·5%	(46·8%, 56·2%)	1,268	203	16·0%	(14·1%, 18·1%)	35·5%	(30·4%, 40·6%)
**Alberta** [b]	76	37	48·7%	(37·8%, 59·7%)	276	37	13·4%	(9·9%, 17·9%)	35·3%	(23·3%, 47·2%)
**Atlantic Canada** [b]$	70	25	35·7%	(25·5%, 47·4%)	260	50	19·2%	(14·9%, 24·5%)	16·5%	(4·3%, 28·7%)
**British Columbia** [b]$	140	55	39·3%	(31·6%, 47·6%)	425	70	16·5%	(13·2%, 20·3%)	22·8%	(14·0%, 31·6%)
**Ontario** [b]	630	292	46·3%	(42·5%, 50·3%)	2,469	340	13·8%	(12·5%, 15·2%)	32·6%	(28·5%, 36·7%)
**Saskatchewan-Manitoba** [b] $	40	25	62·5%	(47·0%, 75·8%)	185	30	16·2%	(11·6%, 22·2%)	46·3%	(30·4%, 62·2%)
**New South Wales** [n]	530	211	39·8%	(35·7%, 44·0%)	1,440	185	12·8%	(11·2%, 14·7%)	27·0%	(22·5%, 31·5%)
**Victoria** [n]	315	146	46·3%	(40·9%, 51·9%)	1,261	145	11·5%	(9·9%, 13·4%)	34·9%	(29·1%, 40·6%)
**New Zealand** [b]	440	166	37·7%	(33·3%, 42·3%)	756	93	12·3%	(10·1%, 14·8%)	25·4%	(20·3%, 30·5%)
Stomach cancer										
**Denmark** [n]	719	273	38·0%	(34·5%, 41·6%)	1,512	178	11·8%	(10·2%, 13·5%)	26·2%	(22·3%, 30·1%)
**Norway** [b]	943	323	34·3%	(31·3%, 37·3%)	1,414	132	9·3%	(7·9%, 11·0%)	24·9%	(21·5%, 28·3%)
**England** [n]	6,224	3,297	53·0%	(51·7%, 54·2%)	13,589	2,372	17·5%	(16·8%, 18·1%)	35·5%	(34·1%, 36·9%)
**Northern Ireland** [n]	293	157	53·6%	(47·9%, 59·2%)	697	119	17·1%	(14·5%, 20·0%)	36·5%	(30·2%, 42·9%)
**Scotland** [b]	913	425	46·5%	(43·3%, 49·8%)	1,424	221	15·5%	(13·7%, 17·5%)	31·0%	(27·3%, 34·8%)
**Wales** [b]	648	317	48·9%	(45·1%, 52·8%)	1,008	185	18·4%	(16·1%, 20·9%)	30·6%	(26·0%, 35·1%)
**Alberta** [b]	161	69	42·9%	(35·5%, 50·6%)	323	56	17·3%	(13·6%, 21·8%)	25·5%	(16·8%, 34·2%)
**Atlantic Canada** [b]$	180	85	47·2%	(40·1%, 54·5%)	400	70	17·5%	(14·1%, 21·5%)	29·7%	(21·5%, 37·9%)
**British Columbia** [b]$	315	120	38·1%	(32·9%, 43·6%)	450	75	16·7%	(13·5%, 20·4%)	21·4%	(15·1%, 27·8%)
**Ontario** [b]	1,738	678	39·0%	(36·7%, 41·3%)	4,727	592	12·5%	(11·6%, 13·5%)	26·5%	(24·0%, 29·0%)
**Saskatchewan-Manitoba** [b] $	150	60	40·0%	(32·5%, 48·0%)	285	40	14·0%	(10·5%, 18·5%)	26·0%	(17·1%, 34·8%)
**New South Wales** [n]	969	322	33·2%	(30·3%, 36·3%)	2,210	217	9·8%	(8·6%, 11·1%)	23·4%	(20·2%, 26·6%)
**Victoria** [n]	616	232	37·7%	(33·9%, 41·6%)	1,981	191	9·6%	(8·4%, 11·0%)	28·0%	(24·0%, 32·1%)
**New Zealand** [b]	726	295	40·6%	(37·1%, 44·2%)	794	99	12·5%	(10·3%, 14·9%)	28·2%	(23·9%, 32·4%)
Colon cancer										
**Denmark** [n]	3,655	781	21·4%	(20·1%, 22·7%)	9,273	456	4·9%	(4·5%, 5·4%)	16·5%	(15·1%, 17·9%)
**Norway** [b]	5,354	1,091	20·4%	(19·3%, 21·5%)	9,587	474	4·9%	(4·5%, 5·4%)	15·4%	(14·3%, 16·6%)
**England** [n]	25,859	8,789	34·0%	(33·4%, 34·6%)	62,360	5,084	8·2%	(7·9%, 8·4%)	25·8%	(25·2%, 26·5%)
**Northern Ireland** [n]	914	274	30·0%	(27·1%, 33·0%)	2,932	212	7·2%	(6·3%, 8·2%)	22·7%	(19·6%, 25·9%)
**Scotland** [b]	3,366	996	29·6%	(28·1%, 31·2%)	6,234	457	7·3%	(6·7%, 8·0%)	22·3%	(20·6%, 23·9%)
**Wales** [b]	2,102	684	32·5%	(30·6%, 34·6%)	4,040	316	7·8%	(7·0%, 8·7%)	24·7%	(22·6%, 26·9%)
**Alberta** [b]	747	175	23·4%	(20·5%, 26·6%)	1,633	103	6·3%	(5·2%, 7·6%)	17·1%	(13·9%, 20·4%)
**Atlantic Canada** [b]$	850	155	18·2%	(15·8%, 21·0%)	2,205	120	5·4%	(4·6%, 6·5%)	12·8%	(10·0%, 15·6%)
**British Columbia** [b]$	1,360	235	17·3%	(15·4%, 19·4%)	3,185	145	4·6%	(3·9%, 5·3%)	12·7%	(10·6%, 14·9%)
**Ontario** [b]	6,653	1,186	17·8%	(16·9%, 18·8%)	17,828	816	4·6%	(4·3%, 4·9%)	13·2%	(12·3%, 14·2%)
**Saskatchewan-Manitoba** [b] $	635	115	18·1%	(15·3%, 21·3%)	1,645	85	5·2%	(4·2%, 6·3%)	12·9%	(9·8%, 16·1%)

**New South Wales** [n]	4,499	783	17·4%	(16·3%, 18·5%)	11,817	444	3·8%	(3·4%, 4·1%)	13·6%	(12·5%, 14·8%)
**Victoria** [n]	2,689	465	17·3%	(15·9%, 18·8%)	9,058	374	4·1%	(3·7%, 4·6%)	13·2%	(11·7%, 14·7%)
**New Zealand** [b]	3,480	731	21·0%	(19·7%, 22·4%)	6,022	273	4·5%	(4·0%, 5·1%)	16·5%	(15·0%, 17·9%)
Rectal cancer										
**Denmark** [n]	811	144	17·8%	(15·3%, 20·5%)	5,194	167	3·2%	(2·8%, 3·7%)	14·5%	(11·9%, 17·2%)
**Norway** [b]	888	169	19·0%	(16·6%, 21·7%)	4,724	118	2·5%	(2·1%, 3·0%)	16·5%	(13·9%, 19·2%)
**England** [n]	3,628	1,287	35·5%	(33·9%, 37·0%)	30,277	1,349	4·5%	(4·2%, 4·7%)	31·0%	(29·4%, 32·6%)
**Northern Ireland** [n]	125	47	37·6%	(29·6%, 46·3%)	1,251	50	4·0%	(3·0%, 5·2%)	33·6%	(25·0%, 42·2%)
**Scotland** [b]	499	139	27·9%	(24·1%, 31·9%)	3,036	95	3·1%	(2·6%, 3·8%)	24·7%	(20·7%, 28·7%)
**Wales** [b]	355	127	35·8%	(31·0%, 40·9%)	2,222	102	4·6%	(3·8%, 5·5%)	31·2%	(26·1%, 36·2%)
**Alberta** [b]	136	28	20·6%	(14·6%, 28·1%)	817	30	3·7%	(2·6%, 5·2%)	16·9%	(10·0%, 23·8%)
**Atlantic Canada** [b]$	125	40	32·0%	(24·5%, 40·6%)	935	45	4·8%	(3·6%, 6·4%)	27·2%	(18·9%, 35·5%)
**British Columbia** [b]$	220	50	22·7%	(17·7%, 28·7%)	1,430	40	2·8%	(2·1%, 3·8%)	19·9%	(14·3%, 25·5%)
**Ontario** [b]	860	179	20·8%	(18·2%, 23·7%)	6,985	181	2·6%	(2·2%, 3·0%)	18·2%	(15·5%, 21·0%)
**Saskatchewan-Manitoba** [b] $	120	35	29·2%	(21·8%, 37·8%)	795	35	4·4%	(3·2%, 6·1%)	24·8%	(16·5%, 33·0%)
**New South Wales** [n]	660	107	16·2%	(13·6%, 19·2%)	4,799	108	2·3%	(1·9%, 2·7%)	14·0%	(11·1%, 16·8%)
**Victoria** [n]	403	75	18·6%	(15·1%, 22·7%)	4,035	94	2·3%	(1·9%, 2·8%)	16·3%	(12·5%, 20·1%)
**New Zealand** [b]	643	105	16·3%	(13·7%, 19·4%)	2,604	77	3·0%	(2·4%, 3·7%)	13·4%	(10·4%, 16·3%)
Liver cancer										
**Denmark** [n]	668	373	55·8%	(52·0%, 59·6%)	873	162	18·6%	(16·1%, 21·3%)	37·3%	(32·7%, 41·8%)
**Norway** [b]	597	329	55·1%	(51·1%, 59·1%)	584	127	21·7%	(18·6%, 25·3%)	33·4%	(28·2%, 38·6%)
**England** [n]	7,497	4,591	61·2%	(60·1%, 62·3%)	10,202	2,341	22·9%	(22·1%, 23·8%)	38·3%	(36·9%, 39·7%)
**Northern Ireland** [n]	215	153	71·2%	(64·8%, 76·8%)	367	93	25·3%	(21·2%, 30·0%)	45·8%	(38·3%, 53·3%)
**Scotland** [b]	1,111	580	52·2%	(49·3%, 55·1%)	1,229	197	16·0%	(14·1%, 18·2%)	36·2%	(32·6%, 39·8%)
**Wales** [b]	583	342	58·7%	(54·6%, 62·6%)	572	123	21·5%	(18·3%, 25·1%)	37·2%	(31·9%, 42·4%)
**Alberta** [b]	152	86	56·6%	(48·6%, 64·2%)	338	63	18·6%	(14·8%, 23·1%)	37·9%	(29·0%, 46·8%)
**Atlantic Canada** [b]$	115	75	65·2%	(56·1%, 73·3%)	210	60	28·6%	(22·9%, 35·0%)	36·6%	(26·0%, 47·3%)
**British Columbia** [b]$	345	210	60·9%	(55·6%, 65·9%)	535	125	23·4%	(20·0%, 27·1%)	37·5%	(31·2%, 43·8%)
**Ontario** [b]	1,167	493	42·2%	(39·4%, 45·1%)	2,962	392	13·2%	(12·1%, 14·5%)	29·0%	(25·9%, 32·1%)
**Saskatchewan-Manitoba** [b] $	105	55	52·4%	(42·9%, 61·7%)	190	55	28·9%	(23·0%, 35·8%)	23·4%	(11·9%, 35·0%)
**New South Wales** [n]	1,161	584	50·3%	(47·4%, 53·2%)	1,994	280	14·0%	(12·6%, 15·6%)	36·3%	(33·0%, 39·5%)
**Victoria** [n]	742	315	42·5%	(38·9%, 46·0%)	1,581	231	14·6%	(13·0%, 16·4%)	27·8%	(23·9%, 31·8%)
**New Zealand** [b]	618	310	50·2%	(46·2%, 54·1%)	625	78	12·5%	(10·1%, 15·3%)	37·7%	(33·0%, 42·4%)
Pancreatic cancer										
**Denmark** [n]	1,674	907	54·2%	(51·8%, 56·6%)	1,817	481	26·5%	(24·5%, 28·5%)	27·7%	(24·6%, 30·8%)
**Norway** [b]	2,059	1,173	57·0%	(54·8%, 59·1%)	1,656	448	27·1%	(25·0%, 29·2%)	29·9%	(26·9%, 32·9%)
**England** [n]	14,291	9,329	65·3%	(64·5%, 66·1%)	16,151	5,597	34·7%	(33·9%, 35·4%)	30·6%	(29·6%, 31·7%)
**Northern Ireland** [n]	496	367	74·0%	(70·0%, 77·7%)	666	215	32·3%	(28·8%, 35·9%)	41·7%	(36·5%, 47·0%)
**Scotland** [b]	1,809	1,012	55·9%	(53·6%, 58·2%)	1,245	396	31·8%	(29·3%, 34·4%)	24·1%	(20·7%, 27·6%)
**Wales** [b]	1,115	640	57·4%	(54·5%, 60·3%)	850	300	35·3%	(32·2%, 38·6%)	22·1%	(17·8%, 26·4%)
**Alberta** [b]	333	192	57·7%	(52·3%, 62·8%)	483	136	28·2%	(24·3%, 32·3%)	29·5%	(22·8%, 36·2%)
**Atlantic Canada** [b]$	280	165	58·9%	(53·1%, 64·5%)	450	155	34·4%	(30·2%, 38·9%)	24·5%	(17·2%, 31·7%)
**British Columbia** [b]$	550	325	59·1%	(54·9%, 63·1%)	755	240	31·8%	(28·6%, 35·2%)	27·3%	(22·0%, 32·6%)
**Ontario** [b]	2,279	948	41·6%	(39·6%, 43·6%)	4,177	964	23·1%	(21·8%, 24·4%)	18·5%	(16·1%, 20·9%)
**Saskatchewan-Manitoba** [b] $	250	155	62·0%	(55·8%, 67·8%)	405	140	34·6%	(30·1%, 39·3%)	27·4%	(19·8%, 35·0%)
**New South Wales** [n]	2,036	940	46·2%	(44·0%, 48·3%)	2,462	539	21·9%	(20·3%, 23·6%)	24·3%	(21·6%, 27·0%)

**Victoria** [n]	1,083	481	44·4%	(41·5%, 47·4%)	2,089	460	22·0%	(20·3%, 23·8%)	22·4%	(18·9%, 25·8%)
**New Zealand** [b]	1,317	659	50·0%	(47·3%, 52·7%)	865	245	28·3%	(25·4%, 31·4%)	21·7%	(17·7%, 25·8%)
Lung cancer										
**Denmark** [n]	5,818	2,486	42·7%	(41·5%, 44·0%)	10,185	1,558	15·3%	(14·6%, 16·0%)	27·4%	(26·0%, 28·9%)
**Norway** [b]	5,679	2,625	46·2%	(44·9%, 47·5%)	8,723	1,134	13·0%	(12·3%, 13·7%)	33·2%	(31·7%, 34·7%)
**England** [n]	46,951	29,788	63·4%	(63·0%, 63·9%)	88,445	19,443	22·0%	(21·7%, 22·3%)	41·5%	(40·9%, 42·0%)
**Northern Ireland** [n]	1,843	1,224	66·4%	(64·2%, 68·5%)	3,909	841	21·5%	(20·3%, 22·8%)	44·9%	(42·4%, 47·4%)
**Scotland** [b]	7,863	4,133	52·6%	(51·5%, 53·7%)	10,799	2,179	20·2%	(19·4%, 20·9%)	32·4%	(31·0%, 33·7%)
**Wales** [b]	3,882	2,223	57·3%	(55·7%, 58·8%)	5,483	1,072	19·6%	(18·5%, 20·6%)	37·7%	(35·8%, 39·6%)
**Alberta** [b]	1,169	596	51·0%	(48·1%, 53·8%)	2,347	421	17·9%	(16·4%, 19·5%)	33·0%	(29·8%, 36·3%)
**Atlantic Canada** [b]$	1,165	655	56·2%	(53·4%, 59·0%)	3,305	635	19·2%	(17·9%, 20·6%)	37·0%	(33·9%, 40·2%)
**British Columbia** [b]$	2,015	1,140	56·6%	(54·4%, 58·7%)	4,005	740	18·5%	(17·3%, 19·7%)	38·1%	(35·6%, 40·6%)
**Ontario** [b]	9,640	4,559	47·3%	(46·3%, 48·3%)	25,452	3,841	15·1%	(14·7%, 15·5%)	32·2%	(31·1%, 33·3%)
**Saskatchewan-Manitoba** [b] $	985	535	54·3%	(51·2%, 57·4%)	2,215	455	20·5%	(18·9%, 22·3%)	33·8%	(30·2%, 37·3%)
**New South Wales** [n]	5,941	2,585	43·5%	(42·3%, 44·8%)	10,393	1,421	13·7%	(13·0%, 14·3%)	29·8%	(28·4%, 31·3%)
**Victoria** [n]	2,954	1,339	45·3%	(43·5%, 47·1%)	7,666	1,085	14·2%	(13·4%, 15·0%)	31·2%	(29·2%, 33·1%)
**New Zealand** [b]	4,330	2,053	47·4%	(45·9%, 48·9%)	4,151	574	13·8%	(12·8%, 14·9%)	33·6%	(31·8%, 35·4%)
Ovarian cancer										
**Denmark** [n]	393	102	26·0%	(21·9%, 30·5%)	1,515	75	5·0%	(4·0%, 6·2%)	21·0%	(16·5%, 25·5%)
**Norway** [b]	911	209	22·9%	(20·3%, 25·8%)	1,696	90	5·3%	(4·3%, 6·5%)	17·6%	(14·7%, 20·6%)
**England** [n]	7,120	2,614	36·7%	(35·6%, 37·8%)	13,326	1,131	8·5%	(8·0%, 9·0%)	28·2%	(27·0%, 29·4%)
**Northern Ireland** [n]	243	97	39·9%	(34·0%, 46·2%)	578	67	11·6%	(9·2%, 14·5%)	28·3%	(21·6%, 35·0%)
**Scotland** [b]	909	286	31·5%	(28·5%, 34·6%)	1,214	120	9·9%	(8·3%, 11·7%)	21·6%	(18·1%, 25·0%)
**Wales** [b]	643	176	27·4%	(24·1%, 30·9%)	933	73	7·8%	(6·3%, 9·7%)	19·5%	(15·7%, 23·4%)
**Alberta** [b]	103	25	24·3%	(17·0%, 33·4%)	346	23	6·6%	(4·5%, 9·8%)	17·6%	(8·9%, 26·3%)
**Atlantic Canada** [b]$	115	45	39·1%	(30·7%, 48·3%)	305	30	9·8%	(7·0%, 13·7%)	29·3%	(19·8%, 38·8%)
**British Columbia** [b]$	250	75	30·0%	(24·7%, 35·9%)	500	20	4·0%	(2·6%, 6·1%)	26·0%	(20·1%, 31·9%)
**Ontario** [b]	1,164	331	28·4%	(25·9%, 31·1%)	3,716	188	5·1%	(4·4%, 5·8%)	23·4%	(20·7%, 26·1%)
**Saskatchewan-Manitoba** [b] $	105	35	33·3%	(25·0%, 42·8%)	250	10	4·0%	(2·2%, 7·2%)	29·3%	(20·0%, 38·7%)
**New South Wales** [n]	649	159	24·5%	(21·3%, 28·0%)	1,638	66	4·0%	(3·2%, 5·1%)	20·5%	(17·0%, 23·9%)
**Victoria** [n]	363	87	24·0%	(19·9%, 28·6%)	1,376	59	4·3%	(3·3%, 5·5%)	19·7%	(15·2%, 24·2%)
**New Zealand** [b]	684	151	22·1%	(19·1%, 25·3%)	739	35	4·7%	(3·4%, 6·5%)	17·3%	(13·9%, 20·8%)

EP: Emergency Presentation; CI: Confidence interval, 3m: 3-month

$ To comply with requirements for minimising residual disclosure risk, all actual counts for these jurisdictions were randomly rounded to a higher or lower multiple of 5 within cells. Sample sizes used in different aspects of the analysis may vary minimally for this reason.

C. 12-month mortality.

	EP				Non-EP				Difference, EP - non-EP	
Jurisdiction	**Patients**	**Deaths (12m)**	**Mortality %**	**(95% CI)**	**Patients**	**Deaths (12m)**	**Mortality %**	**(95% CI)**	**%-point**	**(95% CI)**
Oesophageal cancer										
**Denmark** [n]	461	353	76·6%	(72·5%, 80·2%)	1,435	666	46·4%	(43·8%, 49·0%)	30·2%	(25·5%, 34·8%)
**Norway** [b]	435	320	73·6%	(69·2%, 77·5%)	897	385	42·9%	(39·7%, 46·2%)	30·6%	(25·4%, 35·9%)
**England** [n]	5,364	4,499	83·9%	(82·9%, 84·8%)	21,910	10,859	49·6%	(48·9%, 50·2%)	34·3%	(33·1%, 35·5%)
**Northern Ireland** [n]	193	159	82·4%	(76·4%, 87·1%)	779	362	46·5%	(43·0%, 50·0%)	35·9%	(29·5%, 42·3%)
**Scotland** [b]	851	696	81·8%	(79·1%, 84·2%)	2,527	1,289	51·0%	(49·1%, 53·0%)	30·8%	(27·5%, 34·0%)
**Wales** [b]	435	349	80·2%	(76·2%, 83·7%)	1,268	647	51·0%	(48·3%, 53·8%)	29·2%	(24·6%, 33·8%)
**Alberta** [b]	76	57	75·0%	(64·2%, 83·4%)	276	141	51·1%	(45·2%, 56·9%)	23·9%	(12·5%, 35·3%)
**Atlantic Canada** [b]$	75	50	66·7%	(55·4%, 76·3%)	255	130	51·0%	(44·9%, 57·1%)	15·7%	(3·4%, 28·0%)
**British Columbia** [b]$	150	115	76·7%	(69·3%, 82·7%)	430	220	51·2%	(46·4%, 55·9%)	25·5%	(17·2%, 33·8%)
**Ontario** [b]	630	491	77·9%	(74·5%, 81·0%)	2,469	1,219	49·4%	(47·4%, 51·3%)	28·6%	(24·8%, 32·4%)
**Saskatchewan-Manitoba** [b] $	40	30	75·0%	(59·8%, 85·8%)	180	105	58·3%	(51·0%, 65·3%)	16·7%	(1·4%, 31·9%)
**New South Wales** [n]	530	394	74·3%	(70·5%, 77·9%)	1,440	659	45·8%	(43·2%, 48·3%)	28·6%	(24·1%, 33·1%)
**Victoria** [n]	315	238	75·6%	(70·5%, 80·0%)	1,261	545	43·2%	(40·5%, 46·0%)	32·3%	(26·9%, 37·8%)
**New Zealand** [b]	440	344	78·2%	(74·1%, 81·8%)	756	389	51·5%	(47·9%, 55·0%)	26·7%	(21·5%, 32·0%)
Stomach cancer										
**Denmark** [n]	719	461	64·1%	(60·5%, 67·5%)	1,512	563	37·2%	(34·8%, 39·7%)	26·9%	(22·6%, 31·2%)
**Norway** [b]	943	614	65·1%	(62·0%, 68·1%)	1,414	480	33·9%	(31·5%, 36·5%)	31·2%	(27·2%, 35·1%)
**England** [n]	6,224	4,886	78·5%	(77·5%, 79·5%)	13,589	6,519	48·0%	(47·1%, 48·8%)	30·5%	(29·2%, 31·9%)
**Northern Ireland** [n]	293	245	83·6%	(78·9%, 87·4%)	697	338	48·5%	(44·8%, 52·2%)	35·1%	(29·5%, 40·8%)
**Scotland** [b]	913	682	74·7%	(71·8%, 77·4%)	1,424	716	50·3%	(47·7%, 52·9%)	24·4%	(20·6%, 28·3%)
**Wales** [b]	648	512	79·0%	(75·7%, 82·0%)	1,008	517	51·3%	(48·2%, 54·4%)	27·7%	(23·3%, 32·1%)
**Alberta** [b]	161	103	64·0%	(56·3%, 71·0%)	323	149	46·1%	(40·8%, 51·6%)	17·8%	(8·7%, 27·0%)
**Atlantic Canada** [b]$	175	120	68·6%	(61·4%, 75·0%)	395	170	43·0%	(38·2%, 48·0%)	25·5%	(17·1%, 34·0%)
**British Columbia** [b]$	315	210	66·7%	(61·3%, 71·6%)	445	185	41·6%	(37·1%, 46·2%)	25·1%	(18·2%, 32·0%)
**Ontario** [b]	1,738	1,143	65·8%	(63·5%, 68·0%)	4,727	1,653	35·0%	(33·6%, 36·3%)	30·8%	(28·2%, 33·4%)
**Saskatchewan-Manitoba** [b] $	140	90	64·3%	(56·1%, 71·7%)	295	125	42·4%	(36·9%, 48·1%)	21·9%	(12·2%, 31·6%)
**New South Wales** [n]	969	615	63·5%	(60·4%, 66·4%)	2,210	698	31·6%	(29·7%, 33·6%)	31·9%	(28·3%, 35·5%)
**Victoria** [n]	616	407	66·1%	(62·2%, 69·7%)	1,981	698	35·2%	(33·2%, 37·4%)	30·8%	(26·5%, 35·1%)
**New Zealand** [b]	726	496	68·3%	(64·8%, 71·6%)	794	325	40·9%	(37·6%, 44·4%)	27·4%	(22·6%, 32·2%)
Colon cancer										
**Denmark** [n]	3,655	1,292	35·3%	(33·8%, 36·9%)	9,273	1,109	12·0%	(11·3%, 12·6%)	23·4%	(21·7%, 25·1%)
**Norway** [b]	5,354	1,953	36·5%	(35·2%, 37·8%)	9,587	1,163	12·1%	(11·5%, 12·8%)	24·3%	(22·9%, 25·8%)
**England** [n]	25,859	13,412	51·9%	(51·3%, 52·5%)	62,360	12,563	20·1%	(19·8%, 20·5%)	31·7%	(31·0%, 32·4%)
**Northern Ireland** [n]	914	455	49·8%	(46·5%, 53·0%)	2,932	506	17·3%	(15·9%, 18·7%)	32·5%	(29·0%, 36·0%)
**Scotland** [b]	3,366	1,706	50·7%	(49·0%, 52·4%)	6,234	1,242	19·9%	(19·0%, 20·9%)	30·8%	(28·8%, 32·7%)
**Wales** [b]	2,102	1,070	50·9%	(48·8%, 53·0%)	4,040	850	21·0%	(19·8%, 22·3%)	29·9%	(27·4%, 32·3%)
**Alberta** [b]	747	277	37·1%	(33·7%, 40·6%)	1,633	237	14·5%	(12·9%, 16·3%)	22·6%	(18·7%, 26·4%)
**Atlantic Canada** [b]$	855	255	29·8%	(26·9%, 33·0%)	2,205	270	12·2%	(10·9%, 13·7%)	17·6%	(14·2%, 20·9%)
**British Columbia** [b]$	1,360	380	27·9%	(25·6%, 30·4%)	3,190	335	10·5%	(9·5%, 11·6%)	17·4%	(14·8%, 20·1%)
**Ontario** [b]	6,653	2,197	33·0%	(31·9%, 34·2%)	17,828	1,970	11·1%	(10·6%, 11·5%)	22·0%	(20·8%, 23·2%)
**Saskatchewan-Manitoba** [b] $	645	190	29·5%	(26·1%, 33·1%)	1,655	195	11·8%	(10·3%, 13·4%)	17·7%	(13·8%, 21·5%)

**New South Wales** [n]	4,499	1,479	32·9%	(31·5%, 34·3%)	11,817	1,235	10·5%	(9·9%, 11·0%)	22·4%	(20·9%, 23·9%)
**Victoria** [n]	2,689	853	31·7%	(30·0%, 33·5%)	9,058	1,000	11·0%	(10·4%, 11·7%)	20·7%	(18·8%, 22·6%)
**New Zealand** [b]	3,480	1,316	37·8%	(36·2%, 39·4%)	6,022	820	13·6%	(12·8%, 14·5%)	24·2%	(22·4%, 26·0%)
Rectal cancer										
**Denmark** [n]	811	285	35·1%	(31·9%, 38·5%)	5,194	505	9·7%	(8·9%, 10·6%)	25·4%	(22·0%, 28·8%)
**Norway** [b]	888	324	36·5%	(33·4%, 39·7%)	4,724	428	9·1%	(8·3%, 9·9%)	27·4%	(24·2%, 30·7%)
**England** [n]	3,628	2,106	58·0%	(56·4%, 59·6%)	30,277	4,135	13·7%	(13·3%, 14·0%)	44·4%	(42·7%, 46·0%)
**Northern Ireland** [n]	125	74	59·2%	(50·4%, 67·4%)	1,251	151	12·1%	(10·4%, 14·0%)	47·1%	(38·3%, 55·9%)
**Scotland** [b]	499	244	48·9%	(44·5%, 53·3%)	3,036	404	13·3%	(12·1%, 14·6%)	35·6%	(31·0%, 40·1%)
**Wales** [b]	355	201	56·6%	(51·4%, 61·7%)	2,222	331	14·9%	(13·5%, 16·4%)	41·7%	(36·4%, 47·1%)
**Alberta** [b]	136	55	40·4%	(32·6%, 48·8%)	817	87	10·6%	(8·7%, 13·0%)	29·8%	(21·3%, 38·3%)
**Atlantic Canada** [b]$	125	65	52·0%	(43·3%, 60·6%)	935	115	12·3%	(10·3%, 14·6%)	39·7%	(30·7%, 48·7%)
**British Columbia** [b]$	225	90	40·0%	(33·8%, 46·5%)	1,435	155	10·8%	(9·3%, 12·5%)	29·2%	(22·6%, 35·8%)
**Ontario** [b]	860	353	41·0%	(37·8%, 44·4%)	6,985	663	9·5%	(8·8%, 10·2%)	31·6%	(28·2%, 34·9%)
**Saskatchewan-Manitoba** [b] $	125	55	44·0%	(35·6%, 52·8%)	795	95	11·9%	(9·9%, 14·4%)	32·1%	(23·1%, 41·0%)
**New South Wales** [n]	660	258	39·1%	(35·4%, 42·9%)	4,799	387	8·1%	(7·3%, 8·9%)	31·0%	(27·2%, 34·8%)
**Victoria** [n]	403	144	35·7%	(31·2%, 40·5%)	4,035	324	8·0%	(7·2%, 8·9%)	27·7%	(22·9%, 32·5%)
**New Zealand** [b]	643	228	35·5%	(31·9%, 39·2%)	2,604	275	10·6%	(9·4%, 11·8%)	24·9%	(21·0%, 28·8%)
Liver cancer										
**Denmark** [n]	668	540	80·8%	(77·7%, 83·6%)	873	418	47·9%	(44·6%, 51·2%)	33·0%	(28·5%, 37·4%)
**Norway** [b]	597	450	75·4%	(71·8%, 78·7%)	584	254	43·5%	(39·5%, 47·5%)	31·9%	(26·6%, 37·2%)
**England** [n]	7,497	6,309	84·2%	(83·3%, 85·0%)	10,202	5,332	52·3%	(51·3%, 53·2%)	31·9%	(30·6%, 33·2%)
**Northern Ireland** [n]	215	188	87·4%	(82·3%, 91·2%)	367	195	53·1%	(48·0%, 58·2%)	34·3%	(27·5%, 41·1%)
**Scotland** [b]	1,111	892	80·3%	(77·8%, 82·5%)	1,229	540	43·9%	(41·2%, 46·7%)	36·3%	(32·7%, 40·0%)
**Wales** [b]	583	489	83·9%	(80·7%, 86·6%)	572	302	52·8%	(48·7%, 56·9%)	31·1%	(26·0%, 36·1%)
**Alberta** [b]	152	121	79·6%	(72·5%, 85·2%)	338	129	38·2%	(33·1%, 43·5%)	41·4%	(33·2%, 49·7%)
**Atlantic Canada** [b]$	120	100	83·3%	(75·7%, 88·9%)	220	115	52·3%	(45·7%, 58·8%)	31·1%	(21·7%, 40·4%)
**British Columbia** [b]$	340	290	85·3%	(81·1%, 88·7%)	530	280	52·8%	(48·6%, 57·0%)	32·5%	(26·8%, 38·1%)
**Ontario** [b]	1,167	838	71·8%	(69·2%, 74·3%)	2,962	1,126	38·0%	(36·3%, 39·8%)	33·8%	(30·7%, 36·9%)
**Saskatchewan-Manitoba** [b] $	110	85	77·3%	(68·6%, 84·1%)	190	105	55·3%	(48·2%, 62·2%)	22·0%	(11·5%, 32·6%)
**New South Wales** [n]	1,161	875	75·4%	(72·8%, 77·8%)	1,994	755	37·9%	(35·8%, 40·0%)	37·5%	(34·2%, 40·8%)
**Victoria** [n]	742	531	71·6%	(68·2%, 74·7%)	1,581	566	35·8%	(33·5%, 38·2%)	35·8%	(31·7%, 39·8%)
**New Zealand** [b]	618	467	75·6%	(72·0%, 78·8%)	625	254	40·6%	(36·9%, 44·5%)	34·9%	(29·8%, 40·1%)
Pancreatic cancer										
**Denmark** [n]	1,674	1,361	81·3%	(79·4%, 83·1%)	1,817	1,097	60·4%	(58·1%, 62·6%)	20·9%	(18·0%, 23·9%)
**Norway** [b]	2,059	1,730	84·0%	(82·4%, 85·5%)	1,656	938	56·6%	(54·2%, 59·0%)	27·4%	(24·5%, 30·2%)
**England** [n]	14,291	12,682	88·7%	(88·2%, 89·2%)	16,151	11,100	68·7%	(68·0%, 69·4%)	20·0%	(19·1%, 20·9%)
**Northern Ireland** [n]	496	463	93·3%	(90·8%, 95·2%)	666	451	67·7%	(64·1%, 71·2%)	25·6%	(21·5%, 29·8%)
**Scotland** [b]	1,809	1,508	83·4%	(81·6%, 85·0%)	1,245	865	69·5%	(66·9%, 72·0%)	13·9%	(10·8%, 17·0%)
**Wales** [b]	1,115	926	83·0%	(80·7%, 85·1%)	850	576	67·8%	(64·5%, 70·8%)	15·3%	(11·4%, 19·1%)
**Alberta** [b]	333	283	85·0%	(80·8%, 88·4%)	483	311	64·4%	(60·0%, 68·5%)	20·6%	(14·9%, 26·3%)
**Atlantic Canada** [b]$	280	245	87·5%	(83·1%, 90·9%)	450	325	72·2%	(67·9%, 76·2%)	15·3%	(9·6%, 20·9%)
**British Columbia** [b]$	540	465	86·1%	(82·9%, 88·8%)	755	515	68·2%	(64·8%, 71·4%)	17·9%	(13·5%, 22·3%)
**Ontario** [b]	2,279	1,687	74·0%	(72·2%, 75·8%)	4,177	2,402	57·5%	(56·0%, 59·0%)	16·5%	(14·2%, 18·9%)
**Saskatchewan-Manitoba** [b] $	255	215	84·3%	(79·3%, 88·3%)	415	285	68·7%	(64·1%, 72·9%)	15·6%	(9·3%, 22·0%)
**New South Wales** [n]	2,036	1,570	77·1%	(75·2%, 78·9%)	2,462	1,395	56·7%	(54·7%, 58·6%)	20·5%	(17·8%, 23·1%)

**Victoria** [n]	1,083	827	76·4%	(73·7%, 78·8%)	2,089	1,132	54·2%	(52·0%, 56·3%)	22·2%	(18·9%, 25·5%)
**New Zealand** [b]	1,317	1,096	83·2%	(81·1%, 85·1%)	865	598	69·1%	(66·0%, 72·1%)	14·1%	(10·4%, 17·8%)
Lung cancer										
**Denmark** [n]	5,818	4,119	70·8%	(69·6%, 72·0%)	10,185	4,428	43·5%	(42·5%, 44·4%)	27·3%	(25·8%, 28·8%)
**Norway** [b]	5,679	4,382	77·2%	(76·1%, 78·2%)	8,723	3,383	38·8%	(37·8%, 39·8%)	38·4%	(36·9%, 39·9%)
**England** [n]	46,951	40,499	86·3%	(85·9%, 86·6%)	88,445	46,349	52·4%	(52·1%, 52·7%)	33·9%	(33·4%, 34·3%)
**Northern Ireland** [n]	1,843	1,659	90·0%	(88·6%, 91·3%)	3,909	2,172	55·6%	(54·0%, 57·1%)	34·5%	(32·4%, 36·5%)
**Scotland** [b]	7,863	6,330	80·5%	(79·6%, 81·4%)	10,799	5,594	51·8%	(50·9%, 52·7%)	28·7%	(27·4%, 30·0%)
**Wales** [b]	3,882	3,211	82·7%	(81·5%, 83·9%)	5,483	2,885	52·6%	(51·3%, 53·9%)	30·1%	(28·3%, 31·9%)
**Alberta** [b]	1,169	907	77·6%	(75·1%, 79·9%)	2,347	1,055	45·0%	(42·9%, 47·0%)	32·6%	(29·5%, 35·8%)
**Atlantic Canada** [b]$	1,155	945	81·8%	(79·5%, 83·9%)	3,305	1,550	46·9%	(45·2%, 48·6%)	34·9%	(32·1%, 37·7%)
**British Columbia** [b]$	2,010	1,650	82·1%	(80·4%, 83·7%)	4,005	1,795	44·8%	(43·3%, 46·4%)	37·3%	(35·0%, 39·5%)
**Ontario** [b]	9,640	7,356	76·3%	(75·4%, 77·1%)	25,452	10,617	41·7%	(41·1%, 42·3%)	34·6%	(33·6%, 35·6%)
**Saskatchewan-Manitoba** [b] $	985	810	82·2%	(79·7%, 84·5%)	2,205	1,015	46·0%	(44·0%, 48·1%)	36·2%	(33·0%, 39·4%)
**New South Wales** [n]	5,941	4,374	73·6%	(72·5%, 74·7%)	10,393	4,307	41·4%	(40·5%, 42·4%)	32·2%	(30·7%, 33·6%)
**Victoria** [n]	2,954	2,192	74·2%	(72·6%, 75·8%)	7,666	3,128	40·8%	(39·7%, 41·9%)	33·4%	(31·5%, 35·3%)
**New Zealand** [b]	4,330	3,341	77·2%	(75·9%, 78·4%)	4,151	1,868	45·0%	(43·5%, 46·5%)	32·2%	(30·2%, 34·1%)
Ovarian cancer										
**Denmark** [n]	393	186	47·3%	(42·4%, 52·3%)	1,515	227	15·0%	(13·3%, 16·9%)	32·3%	(27·1%, 37·6%)
**Norway** [b]	911	334	36·7%	(33·6%, 39·8%)	1,696	191	11·3%	(9·8%, 12·9%)	25·4%	(21·9%, 28·9%)
**England** [n]	7,120	3,868	54·3%	(53·2%, 55·5%)	13,326	2,580	19·4%	(18·7%, 20·0%)	35·0%	(33·6%, 36·3%)
**Northern Ireland** [n]	243	137	56·4%	(50·1%, 62·5%)	578	153	26·5%	(23·0%, 30·2%)	29·9%	(22·7%, 37·1%)
**Scotland** [b]	909	449	49·4%	(46·2%, 52·6%)	1,214	260	21·4%	(19·2%, 23·8%)	28·0%	(24·0%, 32·0%)
**Wales** [b]	643	259	40·3%	(36·6%, 44·1%)	933	145	15·5%	(13·4%, 18·0%)	24·7%	(20·3%, 29·2%)
**Alberta** [b]	103	48	46·6%	(37·3%, 56·2%)	346	69	19·9%	(16·1%, 24·5%)	26·7%	(16·1%, 37·2%)
**Atlantic Canada** [b]$	120	75	62·5%	(53·6%, 70·6%)	300	70	23·3%	(18·9%, 28·4%)	39·2%	(29·3%, 49·1%)
**British Columbia** [b]$	255	120	47·1%	(41·0%, 53·2%)	505	70	13·9%	(11·1%, 17·1%)	33·2%	(26·4%, 40·0%)
**Ontario** [b]	1,164	558	47·9%	(45·1%, 50·8%)	3,716	547	14·7%	(13·6%, 15·9%)	33·2%	(30·1%, 36·3%)
**Saskatchewan-Manitoba** [b] $	100	45	45·0%	(35·6%, 54·8%)	250	30	12·0%	(8·5%, 16·6%)	33·0%	(22·5%, 43·5%)
**New South Wales** [n]	649	271	41·8%	(38·0%, 45·6%)	1,638	227	13·9%	(12·3%, 15·6%)	27·9%	(23·8%, 32·0%)
**Victoria** [n]	363	136	37·5%	(32·6%, 42·6%)	1,376	164	11·9%	(10·3%, 13·7%)	25·5%	(20·3%, 30·8%)
**New Zealand** [b]	684	284	41·5%	(37·9%, 45·3%)	739	124	16·8%	(14·3%, 19·6%)	24·7%	(20·2%, 29·3%)

EP: Emergency Presentation; CI: Confidence interval, 12m: 12-month

$ To comply with requirements for minimising residual disclosure risk, all actual counts for these jurisdictions were randomly rounded to a higher or lower multiple of 5 within cells. Sample sizes used in different aspects of the analysis may vary minimally for this reason.

Supplementary Material 14. ICBP Module 9 Emergency Presentations Working Group co-authors

Brooke Filsinger, MSc; Ontario study team.
Katharina Forster, MD, PhD; Ontario study team.
Leon May, MPH; Wales study team.
David S Morrison, MD; Scotland study team.
A. Ffion Thomas, MPH; Wales study team.
Janet L Warlow, MSc; Wales study team.
Hui You, MAppStat; New South Wales study team.
